# Transcriptomics reveal the molecular underpinnings of chemosensory proteins in *Chlorops oryzae*

**DOI:** 10.1186/s12864-018-5315-4

**Published:** 2018-12-07

**Authors:** Lin Qiu, Shunjie Tao, Hualiang He, Wenbing Ding, Youzhi Li

**Affiliations:** 1 Hunan Provincial Key Laboratory for Biology and Control of Plant Diseases and Insect Pests, College of Plant Protection, Hunan Agricultural University, Changsha, 410128 China; 2 Hunan Provincial Engineering & Technology Research Center for Biopesticide and Formulation Processing, Changsha, 410128 China

**Keywords:** *Chlorops oryzae*, Transcriptome, Chemosensory genes, Olfactory receptors, Expression profiling

## Abstract

**Background:**

Chemosensory proteins are a family of insect-specific chemical sensors that sense specific chemical cues and regulate insect behavior. Chemosensory proteins have been identified and analyzed in many insect species, such as *Drosophila melanogaster*, *Bactrocera dorsalis* and *Calliphora stygia*. This research has revealed that these proteins play a crucial role in insect orientation, predation and oviposition. However, little is known about the chemosensory proteins of *Chlorops oryzae*, a major pest of rice crops throughout Asia.

**Results:**

Comparative transcription analysis of the genes of *Chlorops oryzae* larvae, pupae and adults identified a total of 104 chemosensory genes, including 25 odorant receptors (ORs), 26 odorant-binding proteins (OBPs), 19 ionotropic receptors (IRs), 23 gustatory receptors (GRs) and 11 sensory neuron membrane proteins (SNMPs). The sequences of these candidate chemosensory genes were confirmed and used to construct phylogenetic trees. Quantitative real-time PCR (qRT-PCR) confirmed that the expression of candidate *OR* genes in different developmental stages was consistent with the fragments per kilobase per million fragments (FPKM) values of differentially expressed genes (DEGs).

**Conclusions:**

The identification of chemosensory genes in *C. oryzae* provides a foundation for the investigation of the function of chemosensory proteins in this species, which, in turn, could allow the development of new, improved methods of controlling this pest.

**Electronic supplementary material:**

The online version of this article (10.1186/s12864-018-5315-4) contains supplementary material, which is available to authorized users.

## Background

Olfactory and gustatory systems play crucial roles in insect orientation, oviposition, host-identification, mate choice and predator avoidance [1–14]. Chemoreception is mediated by odor and taste receptors which are responsible for identifying a diverse array of chemicals [15–17].

*Chlorops oryzae* (Diptera) cause significant economic damage to rice crops throughout Asia. Newly hatched larvae primarily burrow into the stem of rice plants and feed on the growing tips of developing leaves [18]. Most recent studies have focused on the ecology and physiology of this species [18–20], and there is consequently relatively little information on its genetics. In this paper we present the results of genetic and phylogenetic analyses of putative chemosensory genes in *C. oryzae*.

The mechanism responsible for discriminating chemical cues in lepidopteran larvae has been well established [21–26]. In short, hydrophobic volatile molecules are solubilized and ferried from the external environment to sensory neurons where the chemical signals they carry are converted into electric signals [27]. In insects the process of chemoreception involves olfactory receptors (ORs), gustatory receptors (GRs) and ionotropic receptors (IRs). In addition, odorant binding proteins (OBPs), sensory neuron membrane proteins (SNMPs) and odorant-degrading enzymes (ODEs) also play an important role in regulating host behavior [1, 17, 28, 29].

Insect odor and taste receptor genes were first identified in *Drosophila melanogaster* [30, 31] and subsequent research identified the corresponding genes in other Dipteran species, including *Anopheles gambiae*, *Musca domestica*, *Bactrocera dorsalis*, *Calliphora stygia*, *Glossina morsitans morsitans*, *Mayetiola destructor*, *Episyrphus balteatus* and *Eupeodes corollae* [31–36]. ORs and GRs were first regarded as G-protein-coupled receptors (GPCRs) that share a common 7-transmembrane protein. However, subsequent research suggests that these two chemosensory receptors are not homologous with mammalian *OR* genes [37]. Insect GRs have been classified into sweet, bitter, and carbon dioxide receptors [38–42]. More recently, a new class of ionotropic receptor, a variant sub-family of ionotropic glutamate chemosensory receptor (iGluR), has been identified in *Drosophila* [43]. Insect OBPs are small hydrophilic proteins that ferry hydrophobic chemical cues to ORs across the sensilla lymph [44–46]. In the lepidoptera, OBPs are usually divided into general odorant-binding proteins (GOBPs) and pheromone-binding proteins (PBPs). GOBPs and PBPs are involved in recognizing and transporting host plant odorants and pheromones [47, 48]. SNMPs are the transmembrane domain-containing proteins thought to be involved in pheromone and general odorant reception [49, 50].

The transcriptome approach has been a recent advance in investigating the mechanisms underlying chemosensory proteins in various insect taxa [51–54]. We used this approach to identify candidate chemosensory genes (25 *ORs*, 26 *OBPs*, 19 *IRs*, 23 *GRs* and 11 *SNMPs*) in *C. oryzae* larvae, pupae and adults. We then constructed phylogenetic trees to infer the putative functions of each gene, and used quantitative real-time RT-PCR (qPCR) to confirm the expression patterns of *OR* genes in each developmental stage. The identification of putative chemosensory genes is an essential first step for both fully understanding the molecular basis of an insects’ chemosensory system, and developing better pest management tools.

## Results

### Analysis of the *C. oryzae* transcriptome

An Illumina HiSeq platform and Trinity assembly was used to sequence *C. oryzae* larvae, pupae and adult transcriptomes. We obtained 50.64 million (Larvae-1), 50.90 million (Larvae-2) and 44.41 million (Larvae-3) raw-reads from larvae, 55.06 million (Pupae-1), 55.73million (Pupae-2) and 50.59 million (Pupae-3) raw-reads from pupae, and 57.64 million (Adult-1), 62.21 million (Adult-2) and 56.50 million (Adult-3) raw-reads from adults (Additional file 1: Table S1). Filtering obtained 49.97 million (Larvae-1), 50.31 million (Larvae-2), 43.86 million (Larvae-3), 54.36 million (Pupae-1), 54.86 million (Pupae-2), 49.35 million (Pupae-3), 56.12 million (Adult-1), 61.06 million (Adult-2) and 54.82 million (Adult-3), clean-reads. The final transcript dataset contained 201, 810 unigenes with a mean length of 835 bp and N50 length of 1, 243 bp.

### Homology and gene ontology (GO) annotation

A total of 68, 745 (34.1%) unigenes showed significant similarity to known proteins in the NCBI non-redundant protein database when the cut-off of E-value was set to 10^− 5^. E-value distributions suggested that the assembled sequences had 65.3% homology (<1e ^− 30^) with proteins in the Nr database. However, homology fell to 34.7% when E-values were between 1e^− 30^ and 1e^− 5^ (Additional file 2: Figure S1a). Similarity distributions showed that 79.3% of sequences had more than 60% similarity, and 20.7% of sequences had 18–59% similarity, to sequences in the Nr database (Additional file 2: Figure S1b). Species with the highest proportion of similar genes were *M. domestica* (17.9%) followed by *Ceratitis copitata* (15.8%), *Bactrocera cucurbitae* (14.8%), *B. dorsalis* (13.7%) and *D. melanogaster* (3.4%). (Additional file 2: Figure S1c).

Gene ontology (GO) analysis was used to categorize annotated genes into functional groups. Most genes were categorized into the “Biological Process”, “Cellular Process”, “Metabolic Process” and “Single-organism Process”, categories and “Cell” and “Cell Part” comprised the highest proportion of the “Cellular Component” category (Additional file 2: Figure S1d). “Binding” and “Catalytic Activity” were the most common subcategories in the “Molecular Function” category (Additional file 2: Figure S1d).

### Candidate odorant receptors

**Fig. 1:**
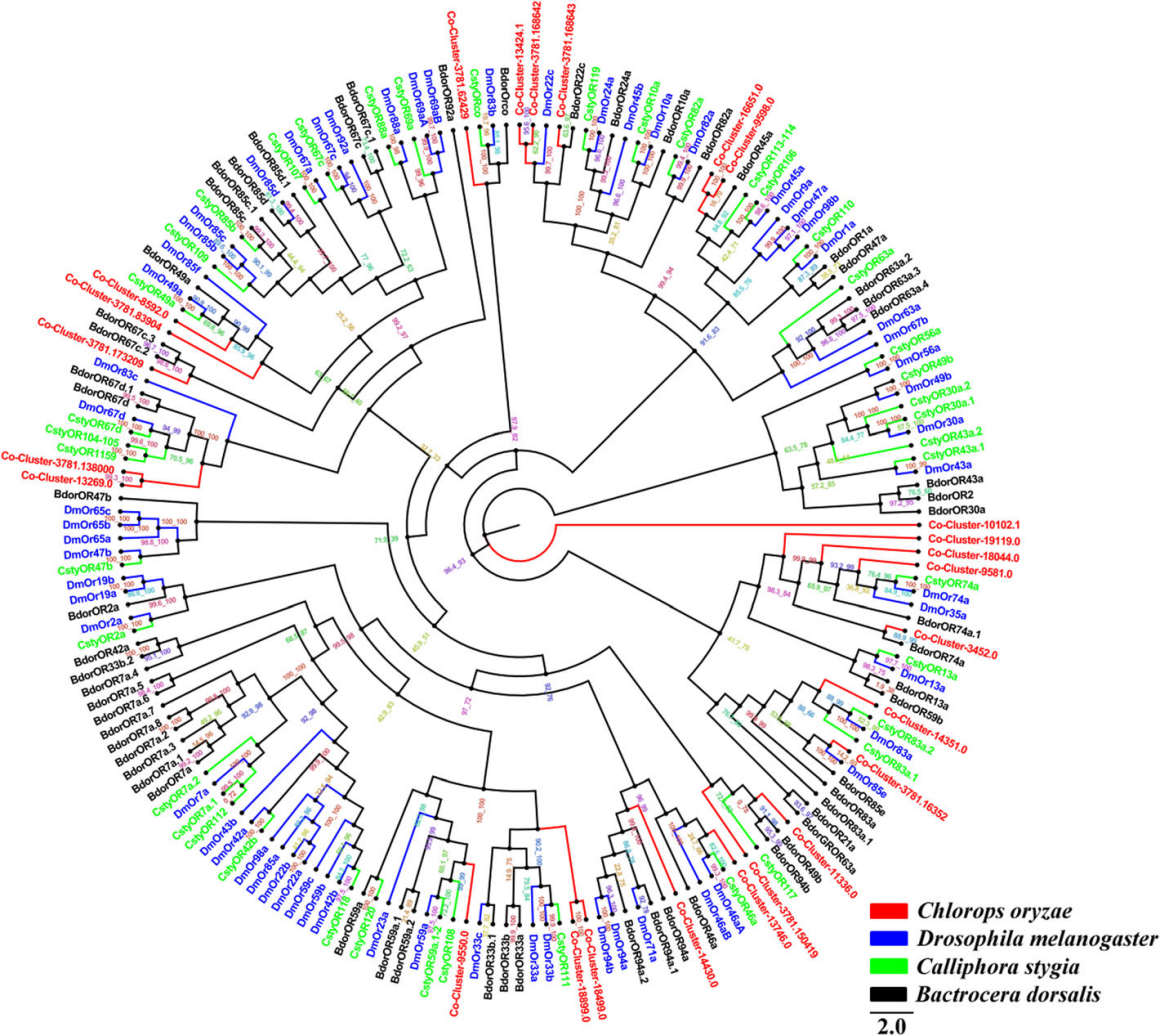
Phylogenetic tree of relationships between *Chlorops oryzae* odorant receptors (ORs) and those of other species; *B. dorsalis* (*Bdor*, black), *C. stygia* (*Csty*, green) and *D. melanogaster* (*Dm*, blue) (Additional file 4). Bootstrap values after 1000 replications

We identified 25 candidate ORs in larval, pupal and adult transcriptomes and constructed a phylogenetic tree of the similarity between these and ORs from three other Dipteran species; *D. melanogaster*, *Calliphora stygia* and *B. dorsalis* (Fig. 1). This indicated that the highly-conserved *C. oryzae* co-receptor, Orco (Cluster-3781.62429), shares 88.1, 87.7, and 85.8% identity with Orco in *C. stygia*, *B. dorsalis* and *D. melanogaster* OR83b, respectively. Cluster-3781.168642 and Cluster-13,424.1 belong to the same cluster and share 68.3% identity based on amino acid sequences. Similarly, 94.2% homology was found between Cluster-16,651.0 and Cluster-9598.0, and 73.4% between Cluster-18,499.0 and Cluster-18,899.0, which were clustered in different groups. Interestingly, even though Cluster-3781.138000 and Cluster-13,269.0 were placed on the same branch they only share 44.0% identity. The remaining *C. oryzae* ORs were placed in different clusters, which is consistent with their highly divergent amino acid sequences. Cluster-10,102.1 was placed on a separate branch of the phylogenetic tree (Fig. 1).

### Candidate gustatory receptors

**Fig. 2:**
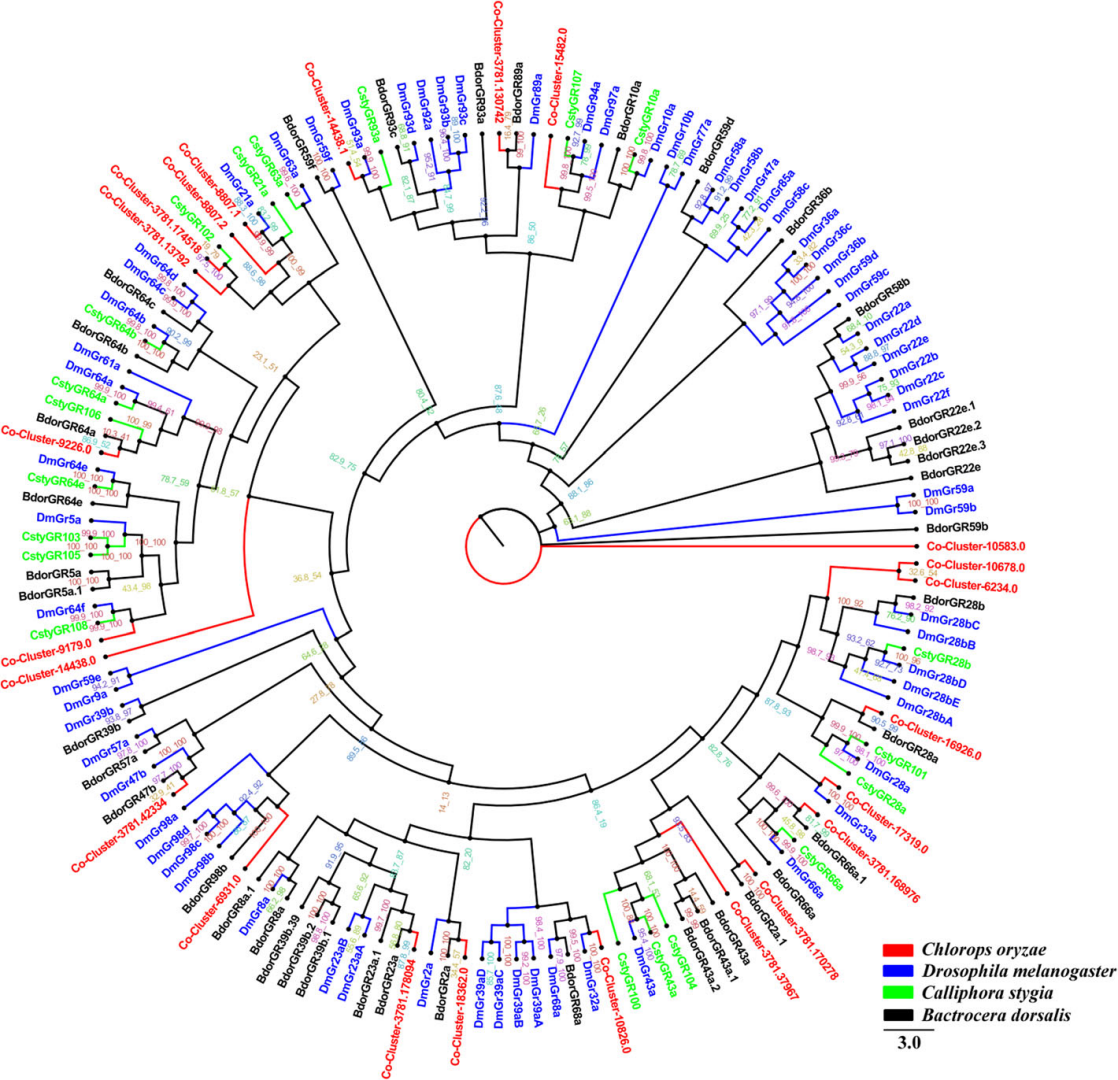
Phylogenetic tree of relationships between *Chlorops oryzae* gustatory receptors (GRs) and those from other species; *B. dorsalis* (*Bdor*, black), *C. stygia* (*Csty*, green) and *D. melanogaster* (*Dm*, blue) (Additional file 5). Bootstrap values after 1000 replications

We identified 23 candidate gustatory receptors (GR) from *C. oryzae* transcriptomes and constructed a phylogenetic tree of the relationships between these and 68 *D. melanogaster* GRs, 20 *C. stygia* GRs and 40 *B. dorsalis* GRs (Fig. 2). Cluster-10,678.0 and Cluster-6234.0 were placed on a single branch and share 12.7% identity. Cluster-8807.1 was classified with a *D. melanogaster* GR (Gr21a) (72.7% identity). *D. melanogaster* Gr21a is a CO_2_ receptor in *Drosophila* [55]. Cluster-3781.37967 and Cluster-3781.170278 were grouped with another *D. melanogaster* CO_2_ receptor (Gr43a) (Fig. 2).

### Candidate ionotropic receptors

**Fig. 3:**
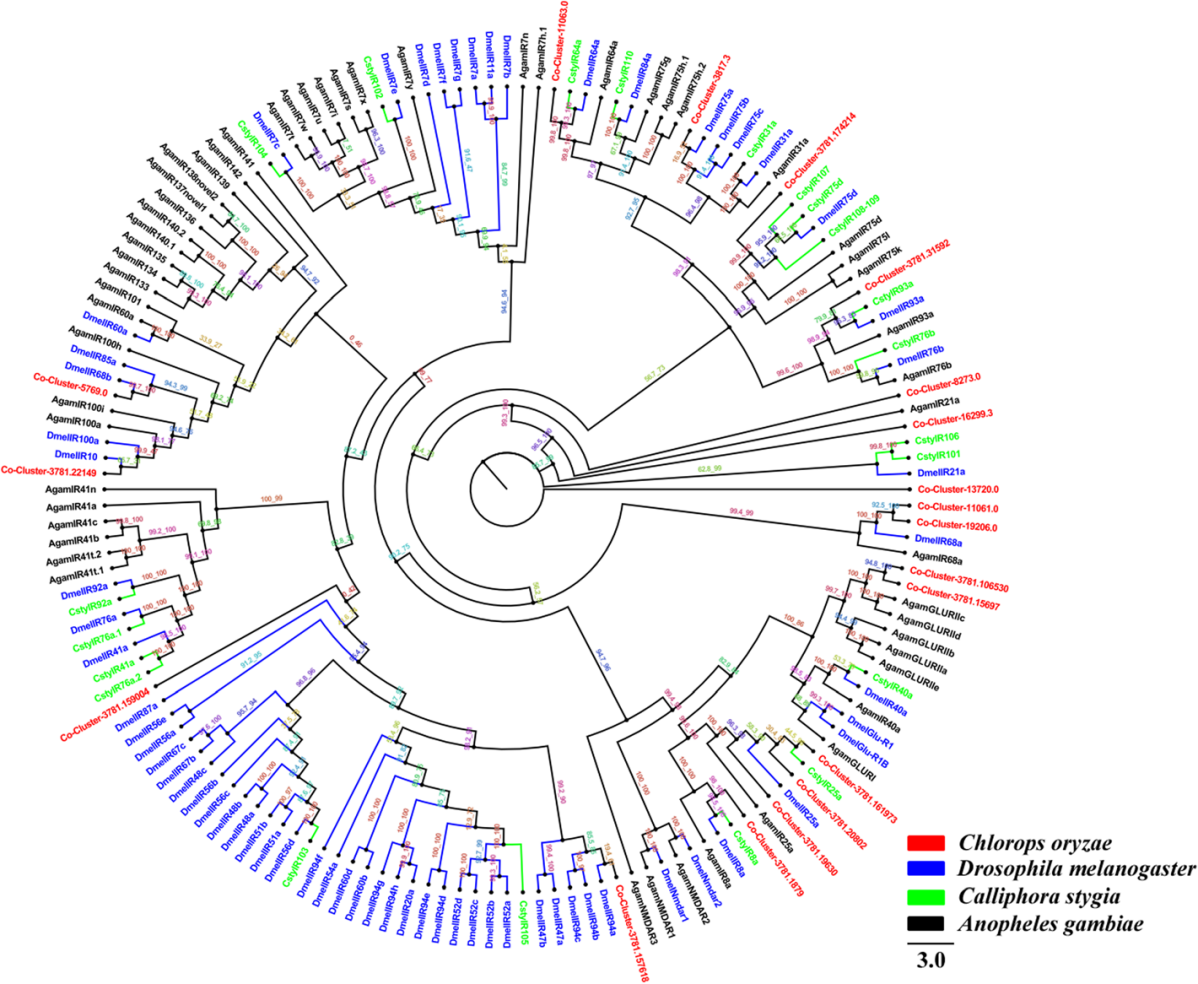
Phylogenetic tree of relationships between *Chlorops oryzae* ionotropic receptors (IRs) and those of other species; *A. gambiae* (*Agam*, black), *C. stygia* (*Csty*, green) and *D. melanogaster* (*Dmel*, blue) (Additional file 6). Bootstrap values after 1000 replications

We identified 19 candidate ionotropic receptor transcripts in *C. oryzae*. To distinguish putative IRs from iGluRs, all *C. oryzae* IRs were aligned with those from *D. melanogaster*, *C. stygia* and *A. gambiae* and a phylogenetic tree constructed of the resultant relationships. This placed Cluster-3781.106530 and Cluster-3781.15697 (49.9% identity) on the same branch as the *A. gambiae* iGluRs (*Agam*GLURIIa, *Agam*GLURIIb, *Agam*GLURIIc, *Agam*GLURIId and *Agam*GLURIIe). Cluster-11,061.0 and Cluster-19,206.0 (97.1% identity) were classified in single a group (Fig. 3). Cluster-3781.159004 and Cluster-3781.157618 were classified with the *D. melanogaster* ionotropic receptor superfamily and *Co*-Cluster-3781.174214 were classified with *Csty*IR107*, Csty*IR75d*, Dmel*IR75d and *Csty*IR108–109. Cluster-8273.0 and Cluster-16,299.3 was classified separately from the IRs (iGluRs) of the other species. The remaining *C. oryzae* IRs were clustered on different branches, however, no *C. oryzae* IRs were placed on the IR7 branch (e.g. *Dmel*IR7a, d, e, f, g; *Agam*IR7i, s, t, u, w, x). IRs on that branch may play various roles in insect olfaction.

### Candidate odorant binding proteins

**Fig. 4:**
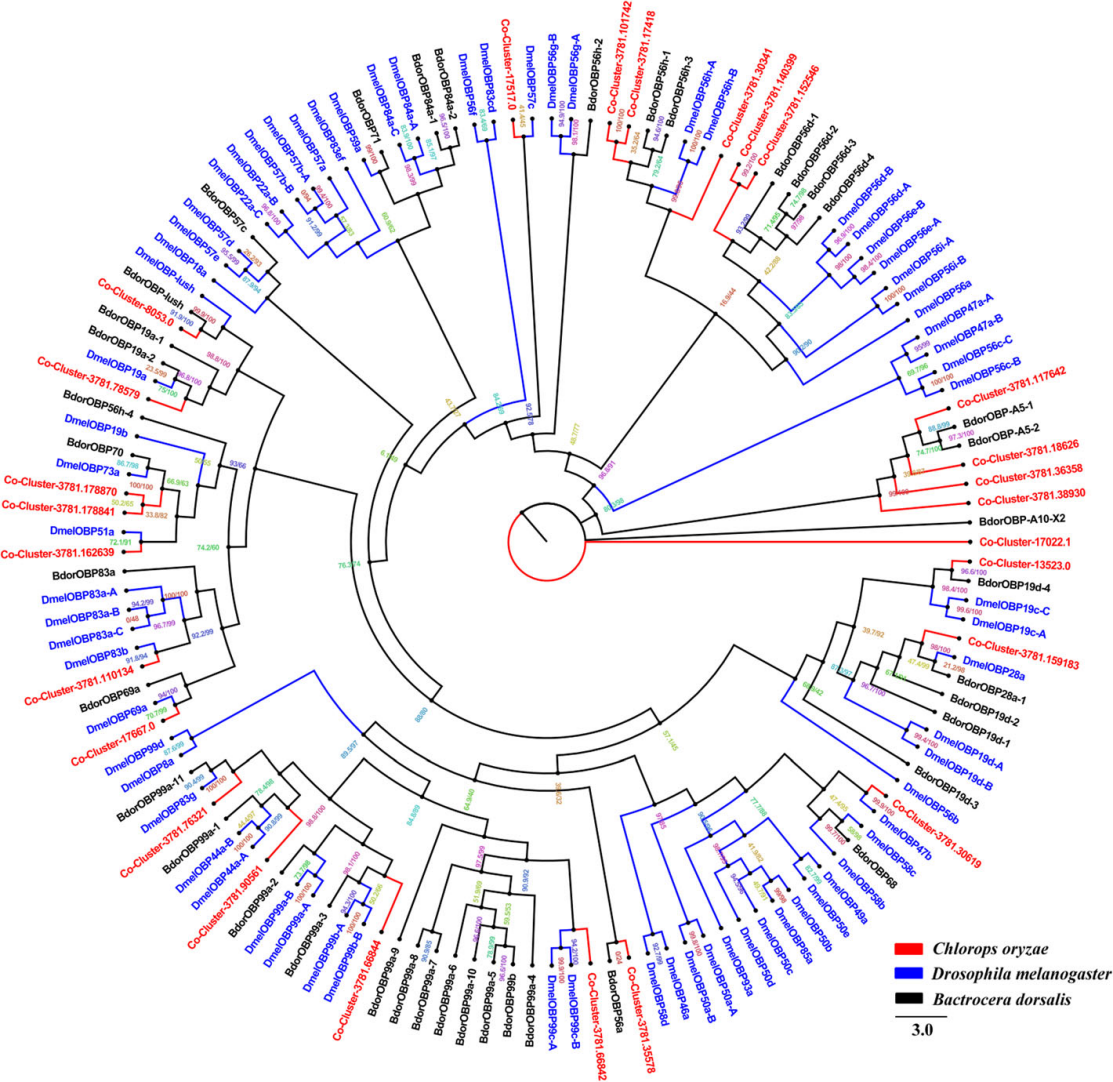
Phylogenetic tree of relationships between *Chlorops oryzae* odorant binding proteins (OBPs) and those of other species; *B. dorsalis* (*Bdor*, black) and *D. melanogaster* (*Dmel*, blue) (Additional file 7). Bootstrap values after 1000 replications

We identified 26 OBP transcripts from *C. oryzae* larvae, pupae and adults and constructed a phylogenetic tree of the relationships between these and OBPs from *D. melanogaster* and *B. dorsalis.* Cluster-3781.101742 and Cluster-3781.17418 were placed in a single group (47.3% identity), Cluster-3781.140399 and Cluster-3781.152546, which share 43.6% identity, were placed on the same branch, as were Cluster-3781.178870 and Cluster-3781.178841 (86.3% identify) (Fig. 4). Four *Co*-OBPs (Cluster-3781.117642, Cluster-3781.18626, Cluster-3781.36358 and Cluster-3781.38930) were clustered with *Bdor*OBP-A5–1 and *Bdor*OBP-A5–2. We found the orthologue of the *Bdor*OBP-lush Cluster-8053.0 in *C. oryzae*, these two OBPs were 56.2% identical at the amino acid level. Cluster-17,022.1 clustered separately with a large group of OBPs which suggests that it may have a novel function in odor recognition. The remaining *Co*-OBPs were placed in different clusters with those of the other species (Fig. 4).

### Candidate SNMPs

**Fig. 5:**
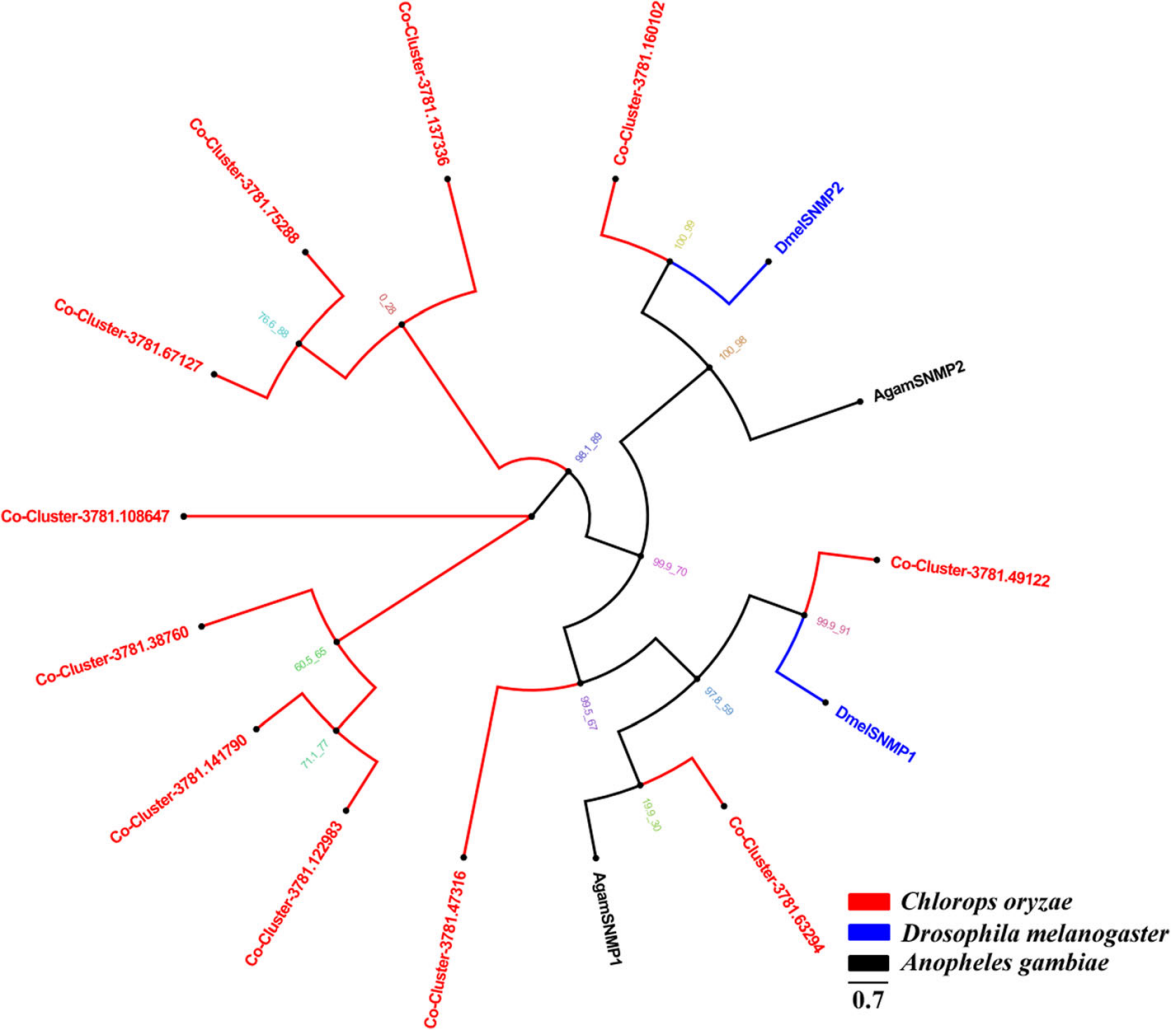
Phylogenetic tree of relationships between *Chlorops oryzae* sensory neuron membrane proteins (SNMPs) and those of other species; *A. gambiae* (*Agam*, black) and *D. melanogaster* (*Dmel*, blue) (Additional file 8). Bootstrap values after 1000 replications

Eleven *C. oryzae* SNMP transcripts were identified and aligned with those of two Dipteran species, *D. melanogaster* and *A. gambiae*. The resultant phylogenetic tree suggests that Cluster-3781.63294, Cluster-3781.49122, Cluster-3781.47316 and Cluster-3781.160102 are similar to *Dmel*SNMP1–2, *Agam*SNMP1–2 (Fig. 5). The remaining SNMPs were placed within a large SNMP superfamily.

### Differentially expressed genes (DEGs)

**Fig. 6:**
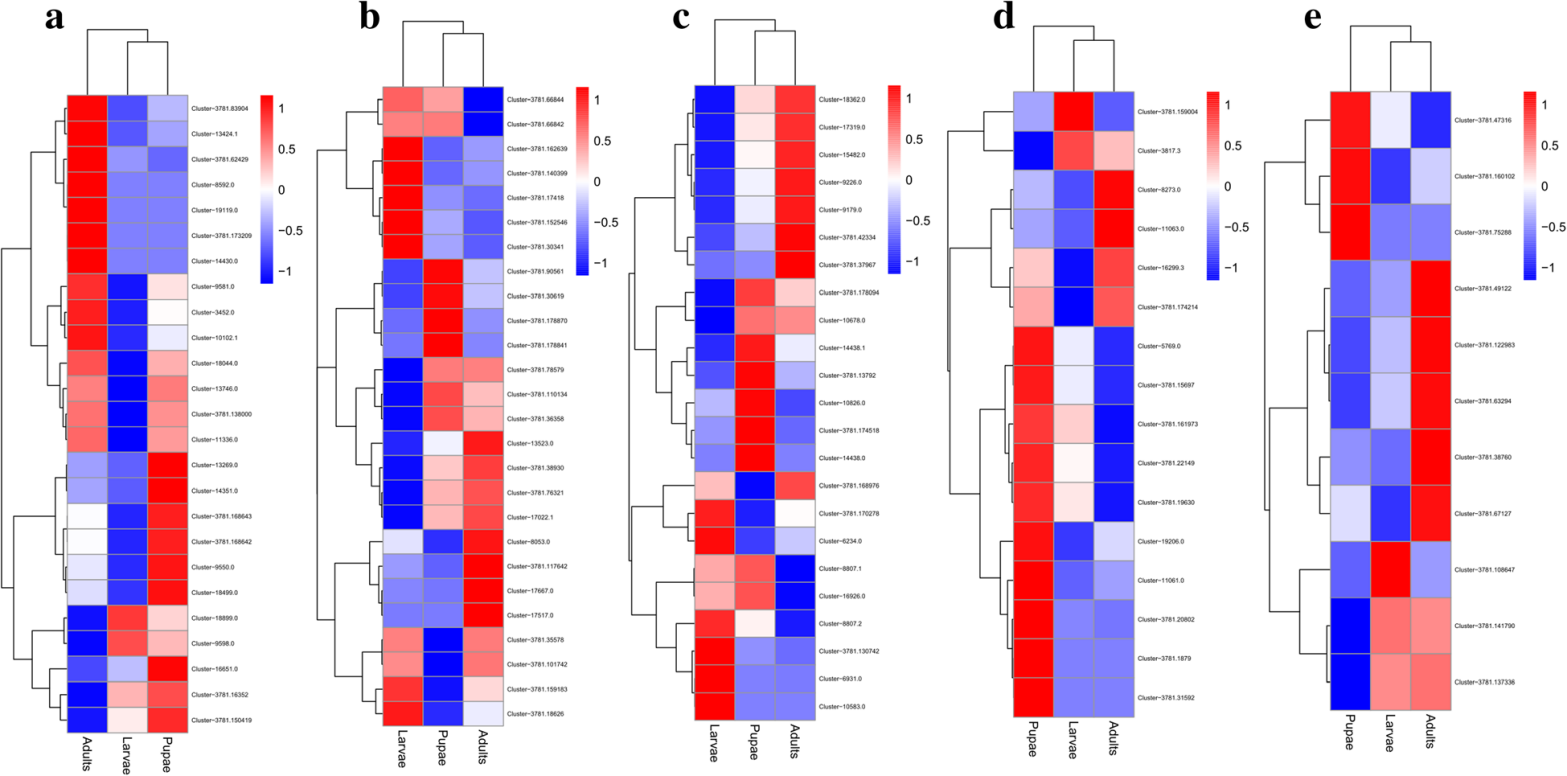
Expression profiles of chemosensory genes in *Chlorops oryzae* larvae, pupae and adults. **a**: *odorant receptors* (*ORs*); **b**: *odorant binding proteins* (*OBPs*); **c**: *gustatory receptors* (*GRs*); **d**: *ionotropic receptors* (*IRs*) and **e**: *sensory neuron membrane proteins* (*SNMPs*)

The expression levels of chemosensory genes in larvae, pupae and adults were estimated as fragments per kilobase per million fragments (FPKM) values and the results shown in a heatmap (Fig. 6). Of the 25 *ORs* identified, 13 were more highly expressed in adults, including *Orco* (Cluster-3781.62429), and 10 were more highly expressed in pupae. Two *ORs* were more highly expressed in pupae compared to both larvae and adults (Fig. 6a). Eight *OBPs* were more highly expressed in adults and 7 were more highly expressed in pupae (Fig. 6b). Eight *GRs* were more highly expressed in adults, 8 were more highly expressed in pupae, and 6 were more highly expressed in larvae (Fig. 6c). Most IR genes were more highly expressed in pupae (Fig. 6d). Five *SNMPs* were more highly expressed in adults and 3 were more highly expressed in pupae (Fig. 6e).

### Specific expression profiles of candidate *OR* genes in different developmental stages

**Fig. 7:**
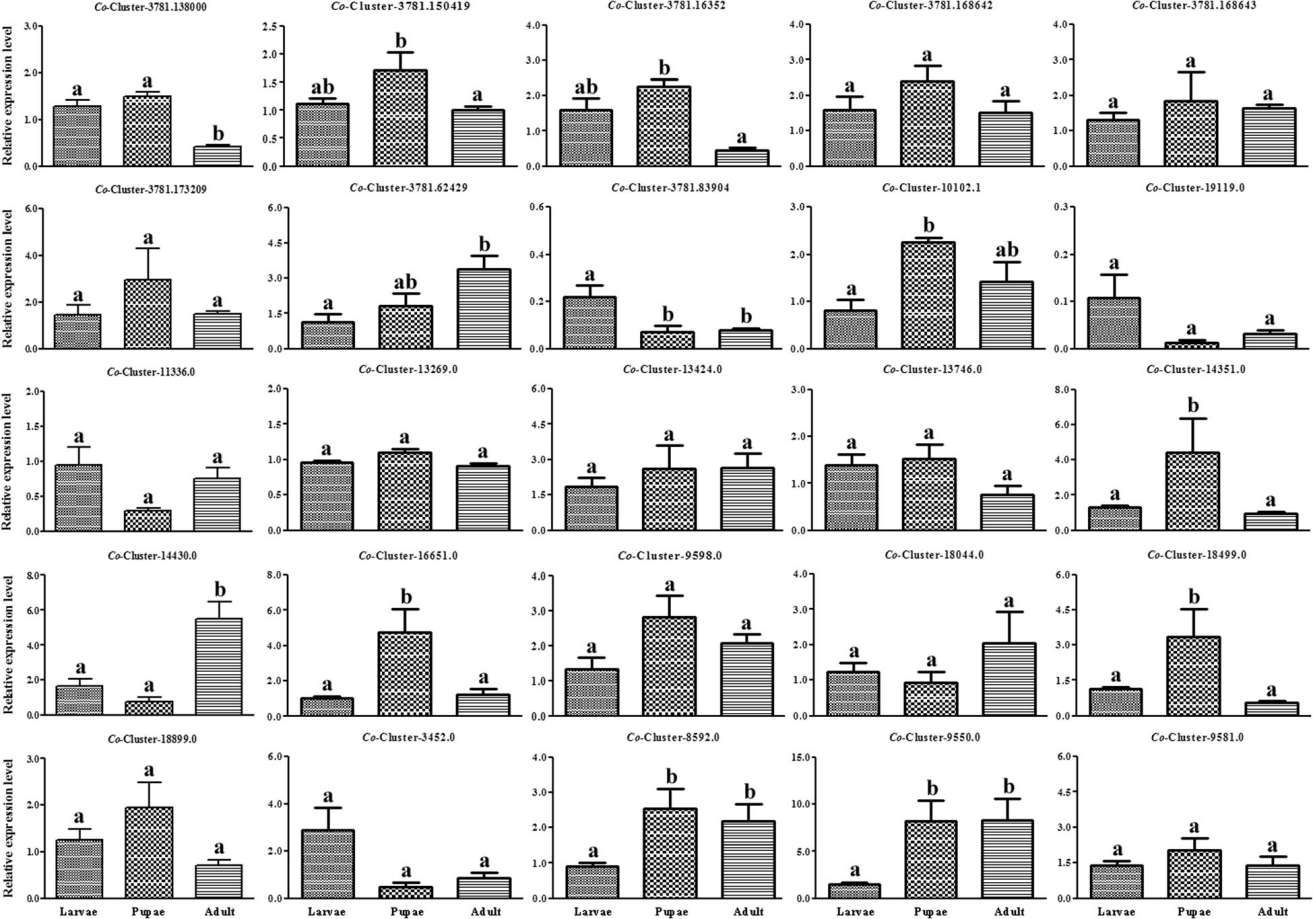
Expression profiles of chemosensory genes as determined by Quantitative Real-time PCR (qRT-PCR) in *Chlorops oryzae* larvae, pupae and adults. Relative amounts of chemosensory genes were normalized to the expression of *GAPDH*. Bars with different letters indicate *P*-values < 0.05 (ANOVA)

We used qRT-PCR to measure the expression levels of candidate *ORs* in larvae, pupae and adults to confirm that the DEGs identified by comparative transcriptomic analysis are differentially expressed in these different developmental stages. We detected all 25 candidate *ORs* in the three developmental stages. The *Orco* gene was highly expressed in adults, as were Cluster-14,430.0, Cluster-8592.0 and Cluster-9550.0 (Fig. 7). Cluster-3781.150419, Cluster-3781.16352, Cluster-14,351.0, Cluster-16,651.0 and Cluster-18,499.0, were more highly expressed in pupae compared to larvae and adults. These results are consistent with the results of the transcriptome analysis.

## Discussion

*C. oryzae* is one of the most important insect pests of rice crops. In the majority of insect species, chemosensory proteins in the olfactory recognition system play a key role in foraging, orientation, mating and oviposition. To better understand how insects perceive olfactory chemical cues, we first identified candidate chemosensory proteins in transcriptomes of *C. oryzae* larvae, pupae and adults, then used qRT-PCR to investigate the expression profiles of *Co-ORs* in these three different developmental stages. Our results provide new evidence of the molecular basis of olfactory proteins in chemosensory reception in *C. oryzae* that may help develop better methods of controlling this pest.

We used next generation sequencing technology to sequence transcriptomes from *C. oryzae* larvae, pupae and adults. De novo assembly of transcripts was performed using the Trinity method and a total of 68, 745 unigenes were obtained from our sequence assembly, 34.1% of which showed similarities to known proteins in the NCBI non-redundant protein database. This percentage is less than that found for other Dipteran species [35, 54, 56].

We identified 104 candidate chemosensory genes in *C. oryzae*. *ORs*, which connect binding proteins and olfactory sensory neurons to transduce olfactory signals, are the best known group of insect chemoreceptors. We identified 25 *C. oryzae* ORs, less than those identified in *C. stygia*, *G. morsitans morsitans*, *D. melanogaster*, *M. domestica* and *A. gambiae* [32, 34–36]. The sequencing methods, or depth, used in these studies may have allowed the detection of genes with lower expression levels [57]. This suggests that different sample preparation and deep sequencing will be required to obtain more functional ORs from *C. oryzae*. Our phylogenetic analysis showed that Cluster-3781.138000 and Cluster-13,269 are part of the *Dmor*67d superfamily. Previous studies have found that OR67d is involved in the perception of the sex pheromone cis-vaccenyl acetate (cVA) [58–60]. Additional functional characterization of the candidate proteins we identified will provide further information on the pheromone reception mechanism in *C. oryzae*.

Our qRT-PCR results confirm that all *OR* genes had different expression profiles in each development stage. Different olfactory organs may express different *OR* genes in different developmental stages. For example, the maxillary palp in larvae plays the same vital role in host detection as the proboscis in adults [61–63].

OBPs also play a vital role or carrying odorants through the hemolymph to olfactory receptor neurons, and transducing the resultant signals to downstream effector molecules in the olfactory system [44]. We identified 26 OBPs in *C. oryzae*, fewer than have been found in other Dipteran species. This may reflect physiological and evolutionary differences between *C. oryzae* and the other Dipteran species [54, 64].

The function of OBPs in the Diptera is relatively well understood [65–69]. For example, *Dmel*OBP-LUSH is involved in recognition of a *D. melanogaster* aggregation pheromone [65]. We found that the *C. oryzae* Cluster-8053.0 clustered with *Dmel*OBP-LUSH and *Bdor*OBP-LUSH, however, because sequence specific attributes may affect OBP function and thereby influence behavior, additional research is required to confirm the function of OBPs in *C. oryzae*.

We identified 23 *C. oryzae* GRs, more than those found in the antennae of *E. balteatus* and *E. corollae* [54]. However, the number of GRs we found in *C. oryzae* is fewer than those reported in other Dipteran species, such as *D. melanogaster* [35]. GRs are known to function as taste and contact receptors [70], so it is not surprising that Cluster-9226.0 is homologous to the *D. melanogaster* sugar receptors *Dm*Gr61a and *Dm*Gr64a [71, 72]. Functional analysis indicates that GRs are involved in host-specific pollination behavior in some insects [73, 74]. Similar functional analysis will be required to confirm the function of candidate GRs in *C. oryzae*.

IRs are conserved in the Diptera where they play a key role in the synaptic ligand gated ion channels involved in chemosensation. We identified 19 IRs in *C. oryzae*, fewer than have been reported in other Dipteran species [35, 54, 75]. In general, IRs function as chemoreceptors [43, 75] and are expressed in the peripheral and internal gustatory neurons associated with taste and food assessment [75], Further studies of *Co*-IRs are required to reveal their physiological and ecological function. We also identified 11 *C. oryzae* SNMP transcripts. SNMPs are conserved throughout holometabolous insects and play important roles in pheromone detection [49, 50, 76–80]. Additional research is needed to verify how the putative SNMP proteins we identified mediate the behavior of *C. oryzae*.

## Conclusions

The 104 candidate *C. oryzae* chemosensory proteins we identified comprise the first comprehensive list of chemosensory proteins in this important agricultural pest. Phylogenetic trees based on the sequence similarity of these putative proteins with similar proteins in other Dipteran species shed light on the molecular basis of olfactory and other behaviors in *C. oryzae* and provide a foundation for developing improved methods of controlling this pest.

## Methods

### Insect rearing and sample collection

*C. oryzae* larvae were collected in Hanshou County, Hunan province, China, in 2017, maintained in a laboratory and reared on fresh rice stems until pupation. Lab conditions were 28 ± 1 °C, > 80% relative humidity, and a photoperiod of 16:8 (L:D) h. Samples of individual insects were collected from 2- to 5-day-old larvae, pupae and adults, respectively. All samples were immediately frozen in liquid nitrogen and stored at − 80 °C until required.

### cDNA library construction and transcriptome analysis

Total RNA of 30 larvae, 30 pupae and 30 adults were individually extracted using TRIzol reagent (Invitrogen, Carlsbad, CA, USA) according to the manufacturer’s instructions. RNA integrity was verified with gel electrophoresis and concentration was measured with a Qubit® RNA Assay Kit in Qubit® 2.0 Flurometer (Life Technologies, CA, USA). 1.5 μg of RNA per sample was used to construct the cDNA (Complementary DNA) library. Sequencing libraries were sequenced on an Illumina Hiseq platform and paired-end reads generated.

All RNAseq data were pre-processed through in-house perl scripts. Briefly, clean data (clean reads) were obtained by removing reads containing adapters, ploy-N and low-quality reads, from the raw data. The Trinity assembly was conducted based on the left.fq and right.fq using Trinity [81] with the min_kmer_cov set to 2 by default and all other parameters set to the default values. The raw sequence data has been uploaded to the National Center for Biotechnology Information (NCBI), under the accession number of SRR7528441 (*C. oryzae* Larvae-1), SRR7528446 (*C. oryzae* Larvae-2), SRR7528467 (*C. oryzae* Larvae-3), SRR7529086 (*C. oryzae* Pupae-1), SRR7529100 (*C. oryzae* Pupae-2), SRR7533623 (*C. oryzae* Pupae-3), SRR7534236 (*C. oryzae* Adult-1), SRR7534658 (Adult-2) and SRR7534603 (Adult-3).

The function of unigenes was inferred by aligning them against Nr (NCBI non-redundant protein sequences), Nt (NCBI non-redundant nucleotide sequences), Pfam (Protein family), KOG/COG (Clusters of Orthologous Groups of proteins), Swiss-Prot (A manually annotated and reviewed protein sequence database), KO (KEGG Ortholog database) and GO (Gene Ontology).

### Differential gene expression

The differential expression of genes in larvae, pupae and adults was measured using the Fragments Per Kilobase of transcripts per Million mapped reads (FPKM) method [82], Differential expression in two conditions/groups (genes and samples) was measured using the DESeq R package (1.10.1). DESeq provides statistical routines for determining differential digital gene expression using a model based on the negative binomial distribution. The resulting *P*-values were adjusted using Benjamini and Hochberg’s approach to control false discovery rate. Genes with an adjusted P-value < 0.05 were considered to be differentially expressed.

### Identification of chemosensory genes

Chemosensory receptor genes were verified by manually checking the amino acid sequences of all identified candidate receptors in BLASTX against the NCBI non-redundant protein database (e-value<1e-5) based on the identity and similarity to orthologous genes from other insect species. The Open reading frame (ORF) of candidate chemosensory genes was predicted by ORF finder tool (https://www.ncbi.nlm.nih.gov/orffinder/).

### Sequencing and phylogenetic analysis

Amino acid sequence alignment was performed using the ClustalW method [83]. Phylogenetic trees of *C. oryzae* chemosensory genes were constructed in IQ-TREE using the best-fitting substitution-model with Maximum-likelihood [84]. Branch support was assessed by bootstrapping with 1000 replicates. OR sequences were obtained from *D. melanogaster*, *C. stygia* and *B. dorsalis*. The GR data set contained GR sequences identified in other Diptera, including *D. melanogaster*, *C. stygia* and *B. dorsalis*. The OBP data set contained OBP sequences from *D. melanogaster* and *B. dorsalis*. The IR data set contained IR sequences from *D. melanogaster*, *C. stygia* and *A. gambiae*. For the SNMP data set, we selected SNMP sequences from *D. melanogaster* and *A. gambiae*.

### Quantitative real-time PCR

We used qRT-PCR with three replicates for each treatment to verify the expression of candidate *C. oryzae* chemosensory genes. cDNA was synthesized from total RNA using a PrimeScript RT reagent kit with gDNA eraser (perfect real time) (Takara, Dalian, China) according to the manufacturer’s instructions. qRT-PCR primers were designed using the National Center for Biotechnology Information’s profile server (https://www.ncbi.nlm.nih.gov/tools/primer-blast/) (Additional file 3: Table S2). The *C. oryzae glyceraldehyde-phosphate dehydrogenase* (*GAPDH*) gene was used as the internal reference. A SYBR® Premix Ex Taq™ (TaKaRa, Dalian, China) and a Bio-rad Detection iQ2 System were used for PCR reactions as follows: 95 °C for 30 s, 40 cycles at 95 °C for 10 s, 59 °C for 30 s. Melting curve analysis was performed from 55 °C to 95 °C to determine the specificity of qPCR primers. To determine the efficiency of the qPCR primers, a standard curve (cDNA concentration vs. Ct) was produced with a 5-fold dilution series of 3rd instar larvae cDNA corresponding to one microgram total RNA. qRT-PCR efficiencies were then calculated according to the equation: E = (10^[− 1/slope]^ − 1)*100 [85, 86]. The 2^−ΔΔCt^ method was used to analyze gene expression profiles [85]. Means and variances of treatments were analyzed with a one-way ANOVA implemented in the SPSS program for windows (SPSS, Chicago, IL, USA).

## Additional files

Additional file 1: **Table S1.** Summary of the transcriptome sequencing data from the *C. oryzae* samples. (DOCX 15 kb)

Additional file 2: **Figure S1.** Results of BLASTx matches of *Chlorops oryzae* transcriptome unigenes and Gene ontology classification. a: E-values, b: gene identity, c: insect species in which homologous genes were matched. d: Gene ontology classifications of *C. oryzae* unigenes. (TIF 192 kb)

Additional file 3: **Table S2.** Primers of candidate *ORs* in *C. oryzae* used for qRT-PCR. (DOCX 16 kb)

Additional file 4: Protein sequences of ORs used to construct phylogenetic tree. (DOCX 52 kb)

Additional file 5: Protein sequences of GRs used to construct phylogenetic tree. (DOCX 49 kb)

Additional file 6: Protein sequences of IRs used to construct phylogenetic tree. (DOCX 72 kb)

Additional file 7: Protein sequences of OBPs used to construct phylogenetic tree. (DOCX 28 kb)

Additional file 8: Protein sequences of SNMPs used to construct phylogenetic tree. (DOCX 17 kb)

## Abbreviations

cDNA: Complementary DNA
DEGs: Differentially expressed genes
FPKM: Fragments per kilobase per million fragments
GO: Gene Ontology
GR: Gustatory receptor
IR: Ionotropic receptor
OBP: Odorant-binding protein
OR: Odorant receptor
ORF: Open reading frame
qRT-PCR: quantitative real-time polymerase chain reaction
SNMP: Sensory neuron membrane protein
