# Supplementary material for: Transcriptomics reveal the molecular underpinnings of chemosensory proteins in Chlorops oryzae
Source: BMC Genomics. 2018 Dec 7;19:890. doi: 10.1186/s12864-018-5315-4 (PMC6286535; doi:10.1186/s12864-018-5315-4)
Supplement: Supplementary file 1 — Table S1. Summary of the transcriptome sequencing data from the C. oryzae samples. (DOCX 15 kb) [file 12864_2018_5315_MOESM1_ESM.docx]

| **Sample** | **Raw Reads (million)** | **Clean reads (million)** |
| --- | --- | --- |
| *Co*-Larvae-1 | 50.64 | 49.97 |
| *Co*-Larvae-2 | 50.90 | 50.31 |
| *Co*-Larvae-3 | 44.41 | 43.86 |
| *Co*-Pupae-1 | 55.06 | 54.36 |
| *Co*-Pupae-2 | 55.73 | 54.86 |
| *Co*-Pupae-3 | 50.59 | 49.35 |
| *Co*-Adult-1 | 57.64 | 56.12 |
| *Co*-Adult-2 | 62.21 | 61.06 |
| *Co*-Adult-3 | 56.50 | 54.82 |

Table S1: Summary of the transcriptome sequencing data from *Chlorops oryzae* samples.
