# Supplementary figures and images for: Transcriptomics reveal the molecular underpinnings of chemosensory proteins in Chlorops oryzae
Source: BMC Genomics. 2018 Dec 7;19:890. doi: 10.1186/s12864-018-5315-4 (PMC6286535; doi:10.1186/s12864-018-5315-4)

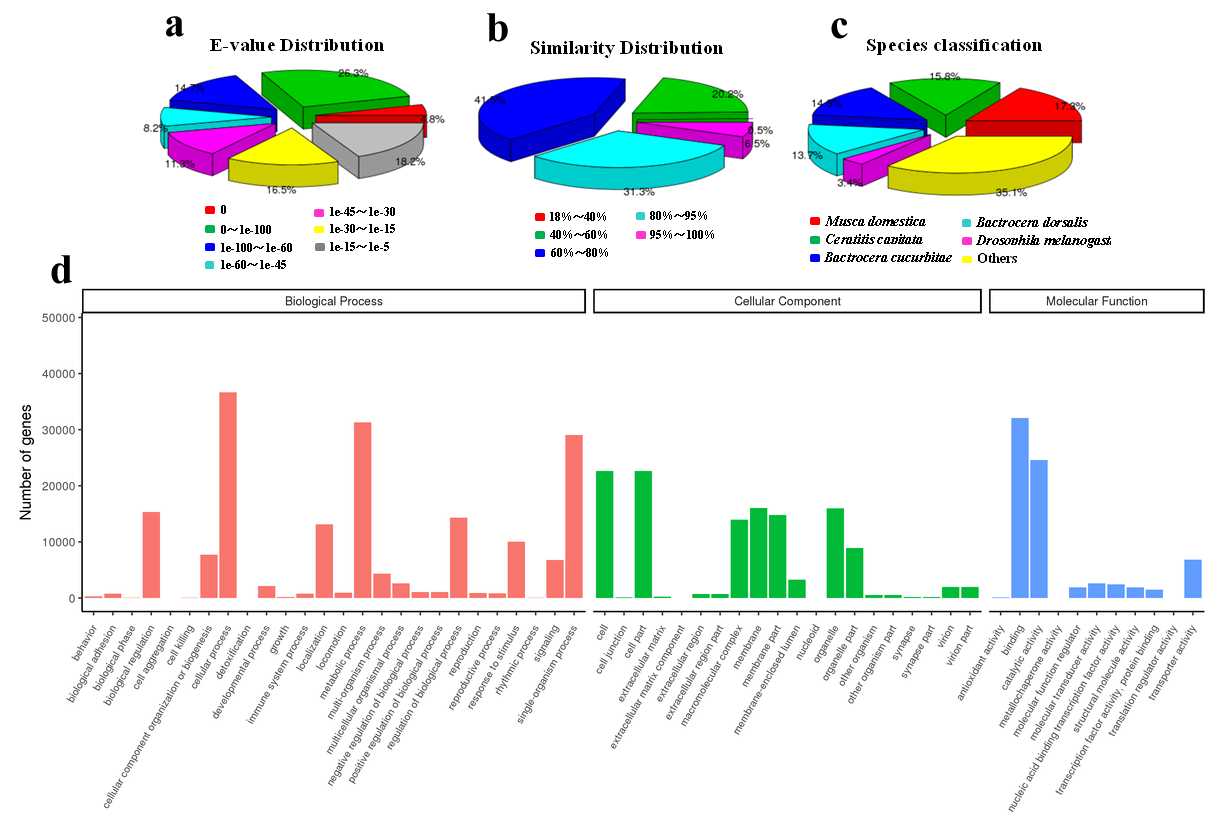

Supplement: Supplementary file 2 — Figure S1. Results of BLASTx matches of Chlorops oryzae transcriptome unigenes and Gene ontology classification. a: E-values, b: gene identity, c: insect species in which homologous genes were matched. d: Gene ontology classifications of C. oryzae unigenes. (TIF 192 kb) [file 12864_2018_5315_MOESM2_ESM.tif]
