# Supplementary material for: Transcriptomics reveal the molecular underpinnings of chemosensory proteins in Chlorops oryzae
Source: BMC Genomics. 2018 Dec 7;19:890. doi: 10.1186/s12864-018-5315-4 (PMC6286535; doi:10.1186/s12864-018-5315-4)
Supplement: Supplementary file 3 — Table S2. Primers of candidate ORs in C. oryzae used for qRT-PCR. (DOCX 16 kb) [file 12864_2018_5315_MOESM3_ESM.docx]

Table S2: Specific primers used in qRT-PCR.

| Genes | Forward primers (5’-3’) | Reverse primers (5’-3’) |
| --- | --- | --- |
| *Co-Cluster-8592.0* | GGTGTACGCTCGGCTACGAA | TGGATGGCGATGGTCGAAACT |
| *Co-Cluster-3781.83904* | ACATTGCCATGGCGGCTTAC | AGCCTGCTTCTGGGAACGTA |
| *Co-Cluster-3781.173209* | GCAAGTGGCAGAGGCAACATT | TTCGAGCGCAAATGTCAGCG |
| *Co-Cluster-3781.138000* | CTTTGTGCCATGCGTCGGAG | CCTGGACGCCAAGCGTAGTA |
| *Co-Cluster-9550.0* | GAGATTGTTGCGATGCCGGG | GCATCGGCCGCGATATGATG |
| *Co-Cluster-18899.0* | CTTGCTGGACACACGCGACT | TCTTCGGCACCTTGCACGAG |
| *Co-Cluster-18499.0* | TGGCGTCTCATCGGCTTCAA | TGCGTTTTTCGGATCGGGGA |
| *Co-Cluster-3781.16352* | GCGCTCACAATGGTCATCCG | CCGTTAACGTGAATGCGCGA |
| *Co-Cluster-3452.0* | CATCAGCTGACGTTGCAGGA | ATCGCGACGGGATTTTGGGT |
| *Co-Cluster-9581.0* | CTTTGCCGCAGCGACTTCAA | TGTGGCTCCGTACAATCCCA |
| *Co-Cluster-18044.0* | AGGCAGCGCCTTGAAGAATTT | GCCGAACACTCGCGAAAATG |
| *Co-Cluster-19119.0* | ACGAGCCAGCAGTTTGAAGG | GCCGATCGTATTCATGGGCCT |
| *Co-Cluster-3781.62429* | TCGGCCTCGTTGCCGATTTA | GATGAGCCGCCAGTGAATGC |
| *Co-Cluster-3781.150419* | CGTCGCGCTGGGAAAACATT | AGACGTGCTTGGGCTCAGTT |
| *Co-Cluster-16651.0* | GGACTACAGCCGCTACAGGG | GCATCCTGCCAGTGCTTTCG |
| *Co-Cluster-3781.168642* | AATGAGGCAAGGGACGGTGT | TCAGGCGGTAGGTGCAACAT |
| *Co-Cluster-3781.168643* | GACACATACGCCCACAAGCA | TGCTTGGTGAATGGAGCACA |
| *Co-Cluster-13269* | CTGCGGACCCTGTTTCTCGT | TCGTGTTTCTCGGTCGCAGT |
| *Co-Cluster-14430.0* | ATCGGGTTGGACCGATCTGC | GCTTTCAGCATGAGTGGCCG |
| *Co-Cluster-13746.0* | CATCGTCATTGCCCGTGATCG | TTCGATGGCTTGCTTCGTGA |
| *Co-Cluster-11336* | GCCACAATCTGCACAGCGAA | CCGGGTCTATATGCCGCACT |
| *Co-Cluster-14351.0* | CTACGCTTGGGCCGTACTCA | GGGGCCGATCGTGTTCGATA |
| *Co-Cluster-10102.1* | GGACGCCGAGGTAAGTCGAA | ACGTTCATAGCTGCCAGGGA |
| *Co-Cluster-9598.0* | GGACAATTGCCTTTGCCCAGT | CGCGAAGCCTTCCCATTTGA |
| *Co-Cluster-13424.1* | ACCTCCCCCAGCTATCAGCA | CCGGTAAATTGGTCGGCTGC |
| *Co-GAPDH* | ACATCCCCGAGAGGCAAGATG | TGGCAGAGAGAATAGCAGCC |
