# Supplementary material for: Transcriptomics reveal the molecular underpinnings of chemosensory proteins in Chlorops oryzae
Source: BMC Genomics. 2018 Dec 7;19:890. doi: 10.1186/s12864-018-5315-4 (PMC6286535; doi:10.1186/s12864-018-5315-4)
Supplement: Supplementary file 4 — Protein sequences of ORs used to construct phylogenetic tree. (DOCX 52 kb) [file 12864_2018_5315_MOESM4_ESM.docx]

>Co-Cluster-10102.1

MRYVPQIEYLSVTGIRVFMGEILLCSICSAKKFFKAKGRIIAEIIIIIMTNIEDEDFESLKIYNTYTSHCRRFSIGSLLSCVVISTFFIIDSLLGPNRDLIFQTYIPGFDIQQSPLYETLFCAQMSVVFFGISTLSPFINLLVAWLFLGISFSRSIQRKIRKIDDVDESLAVDKLKSCIEYHIRLIRYYEKLELNISAIAFVQFLFFSLQLCIVLVWSVSADSLMKKCKTLSFAIFFLMTTGPLYWLANEFADESVKIGESVYGINWIDKDRKFHMNVLFMLKRCQKPLQIRYGSMFVMSMATFQKLLNTCYTIFTFLQTMRSE

>Co-Cluster-19119.0

MGLAYYESNWESVIQVSNNRKENIELMKLISIAIELNQKPIVFMGLNFFHISLASAITILQTAGSYFTCLYTFY

>Co-Cluster-11336.0

MRYFWKFPDNLFATDFKNGVSGSAVLNVWLARLSGVPIFAFKEENFWSKVIILLYGATITILVICLYLCFELYDLVLSWPNLDKMIQNGCLSLTHLTGAYKILNTVSRSNDLFQIFGKFKFLTRTYVKTTKQREAYLRGDRQNIISLIIYASLVAFSGILAIALIYINPENADGKKFPFRVYMPHWIPYAIRINYMGFSLLFFAVQIVAIDYFYISMINQIRFH

>Co-Cluster-13269.0

MTEKKEQGKLPSEEYVNLFGILRTLAKPLGLDLLDDDYHMNALTFLVLSLIVTYPVLMFLTIWRLFQNNWMIVLETSCFSVAVIQAAVKFFVAIFKQKSIYSMYHMLLRIFKVYEKQGPQYHDALMKTNETLRKAVKIMAVTCLIPGISIIVAPFLMLLIDMNNRPLMIPLLFPFIDIDVTSGYYATLGGQAFIMIVTSTGLFVSDLAVLLLLMQFNTFADILGVKIMEMNDTLDDKKKNIHETNIYASLVDIIKWQHDYLWFVDMCNDCYYWIVVACVTTSGLGIVLTLFIIIAGDWPGAYPCLILGFHTLYAYCFMGTRIEICNDEFIASIYETNWYNMDAKEQKIVLFILMNTQKPKNIEIAGVLPLSVATG

>Co-Cluster-13424.1

MLKFLSASVPISRSFFLIPRFAGKLVGCWPQSQRSWYDLVAYCISGLLMLIAGGGEILYGFVHREDLQNALEAFCPGITKAVCVLKLIWFLMYSQSFYETVFKLKTMLTDESTIGKSKEIQRLAAQASLLSFLLLFFGCATTTFFCLSPLITASYGVFTGSNFDLELPFKILLPSLFNRMPSYPLTYVGLTLSAIITPLTFSTVDGFFVCACMYINTIFRNLQQDIRELFSDLKGCDQANFSQSLRFHRELPAIVVRHNAIIDLCSEFTRQFTVIIMMHFLSAAIVLCLSNLNLMLQFGTIDMFIYLFYGFAALTQLFLYCIGGDQVSESSLQVAISLYDIEWYKCDARTKKMILMMLRRSQKAKTVAVPFFTPSLSTFSSILSTAGSYITLLQTFL

>Co-Cluster-13746.0

MYTPYDLECKWIFILTYTYQFLSISMACFVNIAFDTLCPSLLIFIKSQLEILADRLRNISAITGNDDEKVAVELKSAIIFYGDILKIFNDIEKLIRTPISVQLMCSIL

>Co-Cluster-14351.0

MFPLNLKDYFPYKWRLIGIFIDLVFEIFLFFTLLHIPVLYACTLYFFYDDGDLMFSVNCIMQVIIYAWAVLMKVYFRRISPGLLNDLMESINNEYRTRSAPGLTYVTMEEANVLANKWIRRFVFSCFSGTIFWSILPIAYNDRSLPLPCWYPFDYKEPIVYETMYFLQIVGQLQVAAAFSSSSGLHMILAVLLSGQFDVLCCSLKNIIYTTYINMGEGNIKCLREPEIREEYLNTEPNEFYCAKERMEDYVRTIQPQLALSSLNPLSFSQASRISFKKCVLHHRSLLGMLKKMEDFYNFIWLLKCGEVIMLLCLLAFVAVKSTTMNSSFARILSLAQYLLLVIVELLIICYFGEMIYQNSQRCGEALMRSPWYMHMRELRQDFIIFLMNSRRAFKLSAGKMYALDITRFRAVITTAFSFFTILQNMDQ

>Co-Cluster-16651.0

MPAMATYVLSLDNPVTLCLNYTWNVFVTYIALFMFGGIDALFSWGIYNIAAQFRIVQLKMEQLAKQEDKANLRKSIIDCIAYHQQTLELAERFDQVFRTSAFAQFMTSSIPLCFGAFQMGRLREISQILYLMTNMVVILLQLAIYSFGGQYLQNQCDLVATTAFESINWENWSISDRKIFLFTLTRAQKPCKITAIFFDVNMRSFVTVLQSASSYVALLRNLNY

>Co-Cluster-9598.0

MPAMATYVLSLDNPVNICLNYMWNLVVTYNVGFMFGGIDSLFSWAIYNIAAQFRIVQLKMEQLAKQEDKANLRKSIIDCVAYHQQTLELAERFDEVFRTIAFAQFMTSSIPLCFGAFQMGRLREISQILYLMTNMVVILLQLAIYSFGGQYLQNQCDLVATTAFESINWENWSISDRKIFLFTLTRAQKPCKITAIFFDVNMRSFVTVLQSASSYVALLRNLNY

>Co-Cluster-18044.0

MFLCVLGFKVFTNPSESYIFICWSMAKITEMITIGLIGSQLFQTSNELSSMYYHSKWELIIERSPNTRENVRLMKLIFTAMEMSHRPILLTGLKFFDVSLNSVVKILQGAASYFTCLISLR

>Co-Cluster-18499.0

MKMVNEIDSNQVLQRYGLFWRLIGFNYKNPSVFIRAYKLLFIIVVTIYYPIHLIIGLVNIPDPKNAFSNLIMNVAVCSCSMKHFFYWPQLERMKDITRLLTELDRNVRTPVDREFHEKNMRITTDRLLKLFIVAYGFVGLSSILMWAMGEHPSLVFPAYFPFDWKSSTLSYLAAVIFQIVGILLQILMNIANDTYVPLILCLLAGHTRLLANRLARIGYDESKSEKDHYADLIECAEDYQCLMRFFFQTQETISWNMILQSVTNGVNFCVAVIFLVFYADTIFEYGYAVIFLIACSVEFYPGCYYGSSVEKNFHDLTY

>Co-Cluster-18899.0

MFAELNTNRILKRYYMLWHCIGFFYENPSVYIQAYRILFTLIVTIYYPIHLIIGLLHIPDSKDAFRNLMINVAASACSIKHFFYWPQLERMKDITRILLQLDENVLAKEDREFHDKNMSIPTDRLLKLFIATYGFVGLSSVLMWAMAEQPSLVFPAYFPFDWKSSTIAHCCAVIFQIVFFMLQITMNIANDTYVPLALCLLAGHTRLLSNRLSRIGYDESKTEKQHYADLVQGAEDYQRLMRFFYQLQETISWNMVLQSVTNGVNFCVTVIFMVFYAESFFEYGFAIGLLVACSVEFYPGCYYGTLVESNFHQLTYAMFSCNWPDRSPEFKRNLRVLSEFSMREVVMYAKYLVKINLDTYLNTMR

>Co-Cluster-3452.0

MTLISLYAAPIIGFVNGQQLLPFRMIFHFDYRPIPIYIVVLLIAIATGFWVASTMVAEANIMTLLILNLNGRYLILQRQLMSLYERCSINGTVPGNLVIKDFRQGLVGILQQNRQLNQFANQIQQQYSFRIFILMALSAIMLCVLCFKFVTVG

>Co-Cluster-3781.138000

MRCDCIVSMIAISFVAVAVAIAGAVKFAAAISRRETIYKLYGNLLDIYEKHEIIDQYYSEALMETNGRVRRAMKIMCVTCFVPCVGVMMSPFLILLIDSNNRPLMIPLLIPFVDTSTNFGYYTTLGVQGTVVLIGTIGLYAADVIVLVFFMQSYIFADIFHLKIKDFNVELLDET

>Co-Cluster-3781.150419

MKSVRFQAPKTNYDAFTEGPKKYLGYILLNMEPKRSLARRLLHFVALVNCFTFLIAQGNFAVKGCGDLLDRYMAIIFVNYYCVALGKHFTMCIYRLKLRNIFEEFKQLSPSTSQVRQYGLQVYYRNYKFVERFIYNFFVYICLLYSIHPILKSFFMLWHDGEFGFVSILPIWPLIPGYVAYALYCPFIWFSSWIIGFSIVSADVSLLSCISQLCAHFEFLAQRIMELQPEKEESLKLLNEIIKDHQKIVKKIVLLFPLLDSPEKSIPFLHHRL

>Co-Cluster-3781.16352

MSFVLIVLGQFDVLYCSLKNLNPHAKLLSGLNIETLHEMQQDLPSEETKELNQYLVLSEHLTDLSVFREMPVKRNSFKMEYPAALNASLIKCIEMYAFLMKAYERLENLFNPICFVISMQVMVQLCFLIFAAVTRESSGLGMFNQVQYFFLTLCNLAMYTFSGELLRQHSLRCGEAFWRSQWSSDEVMLIRRNLLIFLTNSNRAFTLTAAKLFNVDILFFRKAIAQAFSFFTLLRKITAKNEQL

>Co-Cluster-3781.168642

MHFLSASMVLCLSILNFMLNFGSLNMITFLFYSIAALTQLCLYCIGGNYVNESSQAVGATLYDTEWYKCDVPTRKMILMILRRAQMGKAIAVPFFTPSLASFALIMRTAGSYITLLQTFL

>Co-Cluster-3781.168643

MPLYPVTYAALFLSGTMTVFTFSTVDGYFLCGCLYMSAMFRMLQEDIHDTFSELKEYANDFIKKRKITKKAGRKCYLSFRFFVDCLVNGAHAFKDIFV

>Co-Cluster-3781.173209

MLPLPLARAQRSTHLNAFKFFVISMESFKTLLSTSYQFFALINTQLET

>Co-Cluster-3781.62429

MSLEKMSASDLQSKGKYVGLVADLMPNIRIMKYSGLFMHAFTGGSSFVKKVYSSIHLVLIVMQFAFILVNMALNAEEVNELSGNTITVLFFTHCITKFIYFAVNQKSFYRTLNIWNQVNSHPLFAESDARFHAIALAKMRKLFFLVMLTTVASAISWTTITFFGESIKFAFDKETNQTITVDIPRLPIKSFYPFNAGAGIFYIIAFALQFYYVLFSMVHSNLCDVMFCSWLIFACEQLQHLKGIMKPLMELSASLDTYRPNSAALFRTLSGNSKSELINNEENEQTTDLELNGIYSTKADWGAQFRAATTLQTFAGMNGANPNGLTKKQEMMVRSAIKYWVERHKHVVRLVAAVGDTYGAALLLHMLTSTIMLTLLAYQATKITGINVYALTVIGYLGYALAQVFHFCIFGNRLIEESSSVMEAAYSCHWYDGSEEAKTFVQIVCQQCQKAMIISGAKFFTVSLDLFASVLGAVVTYFMVLVQLK

>Co-Cluster-3781.83904

MAAYMHNWYNASPEYKKLLHILMLRSQKQAQLLAVGLEPLSMDTFKRFMGTVYQVSALLQTMLDLKF

>Co-Cluster-8592.0

MEYSFDHRHPFTYIMVYVFETVNAHYLIISNQGTDLWIVPFIIQLRMHLDRTVHDLLELKFKVENHTDNQRKLAKIIQKHQRLMNVHSELNSSFETCMAFNIIATVGTLCTVGFRISAIGIIKDSLYLWYLLSAVMRFYILCYHGQKLAEASGNVAFAVYNCQWYNASQKCKKMLMILMIRASQPAELTVIGGPPISLDTFKIVMTVSYRIFTIINSMME

>Co-Cluster-9550.0

MQTIISRPMLLQFLVSATNTCVAIGSFLFYAESTASRIYYFFYSFGILLQI

>Co-Cluster-9581.0

MIFPFDHRPLHIYYMIIFTNLWMGLYGATFILGEMNMISHILLHLSERFRLLIEDFGGSVECLLSEINADSIAREYQAEVKRIVQRNLDLYDFAAKV

>Co-Cluster-14430.0

MYVNIGTAETLDDVSNGLYISLTETGLLVKVFNGMRKYRKIVAFLQLFESYSEGLPADEAEMEIARKSHKPFKQFGNVYISGAGIVLAMSFSTPFLQNSYELPFPCWMPFDYKNERNFYYAYIFVCIGITTTAITNSTMDLFQAYLLLQMSIYYRTLGSRLRNFGNKNKGDEITLKKHFDFHTHVMRLTYECESFVSLPVMVQIFLRAFVICFAAYRLQNVNFIESPSEYIKLLTFTIVMAMQIYLPCNYGNELTMSYNDLTYAVYESGWTDLPLGTRKKLFIFMEFLKRPLMLKAGRIFNIGLNVFSQTMNNAYRLYALLLNFEN

>CstyOR120

MAFEENPESVGSLFRTHWIVWKCLGQVPDPRYPKLFKVYAVLLNVGFGLGYPLHLLLGQLGLQTLEEVLLNLTISVPVAVCALKFFNIWRNLRKVRHLEKMFNTLNTRINQRDEWIYYRKVTIPNALKVLHLFYFICVGTALASELTLLIMGFAYEWRLMYPAYFPFDPYATTGGYVVAHTFQIIGLLVQLAENLVSDTYGGMCLALLAGHAHLLGKRVAIIGYDNQKTEMDTGRELANCIVDHNMLFDCHSILGEIIGIGMFAQIISASLIMGIVVIYMVFYVGNAFEYVYYSIYLFGCAMEVFPTCYYATNFEFEFDKLTFMLFSCNWMDQNQSFKKSLMISIEQSLKTRSFRVGGMFRINLQIFFATCKGAYSVLALALKFK

>CstyOR119

MLPQFLTAAYPMERHYFLLPKFALSLIGFYPEQERTFLIRLWSFFNFFILSYGCFAEAYYGIYYIRINIVTALDALCPVASSILSLLKMCCIWWYREELKFLIQRVRVLTEEQRSERKLNYKKKSYTLVTRLTTLLLFCGFCTSTSYSVRHLLDNMLRKAHGKEWIYETPFKMMFPDPLLRLPLYPFIYMLVHWHGYITVVCFVGADGFFLGFCLYLTVLLQSLRDDVTDLLHIKNILNIPTKKEEERIVKQMERLVDRHNEIAELTERLSGVMVEITLGHFVTSSLIIGTSVIDMLLFSGVGIIVYLVYTCAVGTEIYLYCLGGTCIMEACSDLAHSTFSSHWYGHNVRIQKMTLLMIARAQRVLTIKIPFFSPSLETLTSILRFTGSLIALAKSVL

>CstyOR118

MLFRLIRQAPLTEKVASRDGSIYLYRAMKFIGWIPPKEGLLRYVYFSWTLMTFAWCTTFLPLGFLGSYITQIKLFSPGEFLTSLQVCINAYGSSIKVIIVYSQLWRLIKARDLLDKLDVRCTSLEEREKIHRVVARCNHVFLIFTIIYCSYGVSTYLSSVLSGRPPYQLYNPFLDWHNGTRNLWIVSTLEYLIMAGAVLQDQLSDTYSLVYGLILRTHLELLNGRISKLRTNPEMTEDENYEELVNCVLDHKLILEYCALIRPVISGTIFVQFLLIGVVLGIALINLFFFSDAWTGLASAVFIVAILLQTFPFCYICNLVVDDCEDLAHAIFQINWVGSGSRYQSTLFYFLHNVQQPIVFIAGGIFPISMSSNISVAKFAFSVITIVRQMNIADKFKTD

>CstyOR117

MTFFTQCRVVSATTYKRILPDESQAHCEMERLRELTQRIRPSDVDEGRIGSIELNVWLAQLTGLPLSGLKPETKAESIRILVVSGVVLPLLFCYVVLEIYDLVLNWDNVDIMTQNVVMTLTHVGYWFKVLNTFYYYEDIRRIVFTLKHLTRTCVLSPGQRETFHQVEVENKVVCLFYFCLVVFSSTLAMVMLLIVPDNLAGKRFPYRVHMPHFLPPIVQYLYMGLSIIWISCGIPTIDNVNMLFMNQICMHLKILNMAFDVLQRQVDPNIWMVSIVKYHSVLINLRQRLEQIYRLPVLFQFVSSLLVVAMTAFQAIVGDGSGSSVLIYFLFGGVMCQIFLYCWFGNEVFEQSKTLSTSAFGCNWHEFDAQFKRTLLIFMINADRPFLFTAGGFMGLTLTSFANILGKSYSIVTVLRHMYGRAH

>CstyOR1159

MARPTIIKSPSQRFKKFLDVIKLFAKTCGANIFAEDYRINALTCLITILVNCFMLFNFYTIYVSVAKDNYHIVLQNLCVVGTAIQGFSKLINAIVYQDLTRFTCSEIEFMYRTFETEEQHYVDVLNTSLKLLKRIIFTILKIYGILTIAILISPMIIQMITNERLFILNVFIPGVDVDTTVGFIIIQTFNAACTTFSGFGNFAADTACFMLGAHTPLMKDIIKCKLIDLDEVLRKHPKDRSRTEPLVKDIIQWHQRYIIFTEKNTSNFFWMIFIQVASSVMGIISNMVCMFLGGWPVAPLYLLSSFVILFCYCSLGNLVELSNEDMCDNIYECKWYELTVPEQKMILIMLRESQKPNNLSVGGVASLSMNTGLQLTKSIYSVAMLLNNSLN

>CstyOR113-114

MAQIQLPADMDDSYFSIQRRALEIVGFDPSTQRLHMRRPLWAGLLILSLVSHNWPMIVYGLQDLSDLTRLTDNLAVFMQGSLCTLKFLAFIVKRRRIGALVHRLHGLNQEACASPLQREKILRENRLDMYVSRAFRNAAYAVTVASMIAPMLNGLIAYLTEGVFRPTTPMEFNFWLDERQARFYWPIYAWGVLGVAAAVWLAIVADTLFSWLVHNVVAQFQLLKLLLADKERQQAADSDSHLAECIRRHRLALELARELSAIFAEIVFVQYMLSYLQLCMLAFRFTRSGWSSQVPFRAAFLVTVFIQLSSYCYGGEYLKQQSSGVALAVYSGCDWSQMPPARRRLWQMMIMRAQRPAKVFGYMFDVDLPLLLWVTRTTGSFLALLRTFER

>CstyOR111

MDLKPRVIRSEDIYRTYWLYWHLLGLESNFFLNRLLDLVITVFVTIWYPIHLILGLFMDRSLGDICKGLPITAACFFASFKFICFRIKLSEIKQIEILFKELDQRASSQEECDFFNQNTRREANFIWKSFIVAYGLSNISAIASVLFAGGHKLLYPAWFPYDVQASELIFWLSVTYQIAGVSLAILQNLANDSYPPMTFCMVAGHVRLLAMRLSRIGQGPEETKYSTGKQLIESIEDHRKLMKIVELLRSTMNISQLGQFISSGVNISITLVNILFFADNNFAVTYYGVYFLSMVLELFPCCYYGTLISVEMNQLTYAIYSSNWMSMDRSYSRILLIFMQLTLAEVKIKAGGMIGIGMNAFFATVRLAYSFFTLAMSMR

>CstyOR110

MSKLIEVFLGNLWTQRFTFARMGLDLQPDKKGKVLRSPLLFSIMCLTTGFELCTVCAFMVQHRNQIVLCSEALMHGLQMVSSLLKMAIFLAKSHDLMALIQQILAPFAGEDLGDTEWRSQNRRGQLMAAVYFMMCAGTSVSFLLMPVALTMLKYHSTGEFAPVSSFRVLLPYDVTQPHVYAMDCCLMVFVLSFFCCSTTGVDTLYGWCALGLSSQYRRLGQQLKGMRSCFNPHRSDYGLSGLFVEHARLLKLVQQFNLCFMEIAFVEVVIICVLYCSVICQYIMPHTNQNFAFLGFFSMVVTTQLCIYLFGAEQVRLEAERFSRLLYEVIPWQNLPPQHRKLFLFPLERAQRETVLGAYFFELGRPLLVWIFRTAGSFTTLMNALYAKYETH

>CstyOR112

MAVSTRVATKQEVPESRRAFRNLFNCFYALGMQAPDGSRPTTSSTWRRIYACFSVVMYVWQLLLVPTFFVISYRYMGGMEITQVLTSAQVAIDAVILPAKIVALAWNLPLLRRAEHHLAALDARCREQEEFQLILDAVRFCNYLVWFYQICYAIYSSSTFVCAFLLGQPPYALYLPGLDWQRSQMQFCIQAWIEFLIMNWTCLHQASDDVYAVIYLYVVRIQVQLLARRVEKLGTDDSGQVEIYHDERRQEEHCAELQRCIVDHQTMLQLLDCISPVISRTIFVQFLITAAIMGTTMINIFIFANTNTKIASIIYLLAVTLQTAPCCYQATSLMLDNERLALAIFQCQWLGQSARFRKMLLYYLHRAQQPITLTAMKLFPINLATYFSIAKFSFSLYTLIKGMNLGERFNRTN

>CstyOR108

MLYERFAEVGFDDAEDPEDQLEACITDHKRLLELFQTVETFISVSMLIQFAVTGVNVCISVAGVIFLFNDPMTKAYYFFYAAGIILELFPICYYGTDIQICFGNLHYAAFRCNWIKQNRSFKKKLMLFVERSLKGSTPMAGGMLRIHIDTFFSTLKFAYSMFTILIQMRK

>CstyOR109

MDNFLKYANFFYKAVGIEPYARRTQSSGDALRKSIVYWANVINLSVIVFGEVVYVGIAFASNRPVEAIMVMSYIGFVAVGMSKMIIVWLKKPDLSKIMDELHELYPRGEVKQREYKLERYLRSCSRISLTFSTLYSVLIWTYNLFSIMQFLIYELWLQSRVVGQTLPYLMYTPWNWEGNWTYYVMLFCQNFAGYTSAAGQISTDLLLVAVATQVVMHFDYLARSIEGHELAGNWETDSRFLSDSVQYHERLFSLASGLNDIFGVPLLLNFMISSFVICFVGFQMTVGVPPDLMVKLVLFLVSSLCQILLICYYGQLIADSSSGISWAAYKQNWNYADVRYRRALAFIIARAQKPAYLRATIFMRITMGTMTDLLQISYKFFALLRTMYAK

>CstyOR107

MKYLLSMTLLLAIGLQQIDAHGMMLSPPSRSSRWRYDGSAPQNWNDNELFCGGLYRCRGAKESVSVVEDFLMLATKYFYSIGVVPYESDEKPRFGLHLYLGFHVANLLFVWFTMMVFVVNSVRDNEDFLKISMVVGYITFGNVGVLKILVVQLQKRKLTSLVQNLKSLFPQPNKGTHEDFDVEHYLRFSKLISKYFGRLYVAMIVINSASSITQYAIQRWWLHSANVELTLPYVPLAPWNWRGSWTSWPTYLLQSTAAYTCTCGCLSADLMMFAVVMQVIMHFDRLAKALREFDKRCSNGAEEDLNELRSLIVYHNQVLRLTSKMNDIFGVPLLLNFLNSSMLICNVGFQLTIGISLEYIGRQVLIILSALVEVYLICSLSQMLINASKNVSLAVYDMDWLEYDTKFRKMLVLVVMRAQKPVSLNAKAFLSTVSMGTMTTFMQVSYKFFCAIRMMYQ

>CstyOR106

MDSSYFAVQRRALEIVGFNPSTPQLSLKHPLWAGILVLSLVSHNWPMAVYALQDLSDLTRLTDNFAVFMQGSQSTFKFLVMVAKRRRIGSLIHRLHKLNQAESATPKHLEKIERENQLDRYVSRSFRNAAYGVICASAIAPMLLGLWGYVETGVFTPTTPMEFNFWLDERNPHFYWPIYVWGVLGVAAAAWLAIATDTLFSWLIHNVVIQYQLLELVLEEKDLSGGDSRLTECIRRHRLALDLAKELSSIFAEIVFVKYMLSYLQLCMLAFRFSRSGWSAQVPFRATFLVAIIIQLSSYCYGGEYLKQQSLGIAQAVYGNSNWPKMTPKNRRLWQMVIMRAQRPAKIFGFMFDVDLPLLLWVIRTAGSFLALLRTFERSPT

>CstyOR104-105

MARPTIIKSPSQRFKKFLDVIKLFAKTCGANIFAEDYRINALTCLITILVNCFMLFNFYTIYVSVAKDNYHIVLQNLCVVGTAIQGFSKLINAIVYQDLTRFTCSEIEFMYRTFETEEQHYVDVLNTSLKLLKRIIFTILKIYGILTIAILISPMIIQMITNERLFILNVFIPGVDVDTTVGFIIIQTFNAACTTFSGFGNFAADTACFMLGAHTPLMKDIIKCKLIDLDEVLRKHPKDRSRTEPLVKDIIQWHQRYIIFTEKNTSNFFWMIFIQVASSVMGIISNMVCMFLGGWPVAPLYLLSSFVILFCYCSLGNLVELSNEDMCDNIYECKWYELTVPEQKMILIMLRESQKPNNLSVGGVASLSMNTGLQLTKSIYSVAMLLNNSLN

>CstyOR88a

MDAVDEPIKIEQFLQFQKYQQVFHFLHFQFVRNADGRLINKSELVFRAGFLLDVWFFGINLFDILRSIQLGETSHQNLPVLSISIYFTIRGIMMFIKRHDIVDFLNVLDREFPKDLVSQRVLNVPQVFERYHRRHGYVGLYANYALPGFCLTPVVTYILTYEDRNAPILLDHQLLGGWLPYDLRQNHLVYPLVWLYDVYCMLVGVTFFTSFDTLFNTMQAQVIMYLDCFCRQLEALNAVDSYQAMDERQFHEHICGLIRRHQQLNLICDKFNDIFKLAILITDLVGATSICFHLYLITENQDPLMIIKYILPTLALVVFTFEICLRGTQLEEASSRLNEALYNQNWYMGSKMYRKLILIWIKYAQCTRKLTAYGLVEINMKHFSDIMQLAYRLFTFLKSRQ

>CstyOR85b

MCENIESFEAFLRIPSFFYRSVGVDLWNTNGGSIQRFIFYFGFLNVNLWLLSELIFAIITVSENFIQATMTLSYAGFVLVGSIKMYFMWRKKTEMTQFLKLMDEIFPRTAEQQKMMNLRRHLRQSTIVMSGFALIFMILIWTYNLYPFMQRQIYDCWLDTRSINKTLPYESYIPWNWHNHWSFYLYYVLQSIAGYHSAAGQIASDLVLCAMATQMIMHYEYVSHKIRSRYRGERKCDMRWLCETIAYHSNLLSLSDVMNDVLGVPLLVNFMTSSFVICFVGFQMTMDAEPDYMVKLFLFLFSSLAQIYLICHYGQLLIDASINVAAAVYDQDWFDLNVRYQHMLVLVVARAQKPAMLKATNFVRISRGTLTDIMQISYKFFTLIRTMYSD

>CstyOR83a.2

EKGEINPFKRQDLFVFVRQTMCIAAMYPFRYYFKVSGILFGLITFLDVIYEIFNYFVSVHIAALFMCTIYLNYGKGDLDFFVNCLIQTIIYSWMIAMKLYFRRFRPALLDEILKYINEKYEPRSAVGFSYVNMDGSYRMSRLWIKTYVYCCYIGTIFWLALPIAYRDKSLPLACWYPFDYTQPVVYEVVFFLQAVGQIQVAATFASSSGLHMVFCILMSGQYDVLFCSLKNVLATSYVRMGASLAEMRQLEAEQSVSDAEPSQYSYSFELQTPLEQLLQENPKSEVSPDFSRAFRDSFIRCIYHHRYIVSVLKKMEKFYSPIWFLKIGEVTFLMCLVAFVSTKSTAANSFMRMVSLGQYLILVLYELFIICYFAEIVYQNSQRCGEALWRSPWQRHSREVRSDYMFFMLNSRRQFQLTAGKITNLNIERFRGTITTAFSFLTLLQKMDARG

>CstyOR83a.1

MIEIFSIYKDITSEMLLITRKDTMKERNPPLKIKGDSERRDLFVFVRYTMCIAAMYPFGYSLQGSSFLGLLVRVLDWFYEIFNYFVSVHILGLYICTIYINYGQGDLDFFVNCMIQTIIYLWTIAMKLYFRRFRPKMLDEIMSIINENYHTRSALGFSYVTMSGAHHVSKLWIKTYVYCCYIGTIFWLVLPIAYRDKSLPLACWYPFDYTQPIVYETVFFLQAIGQIQVAASFASSSGLHMVFCVLLSGQYDVLFCSLKNVLATSYIHMGGNMAELRQLQSEQSIADAEPNQYAYSHEEQTPLEQLLQHPPQDESSRDFLKAFKRSFRHCIDHHRYIVEVLKKMERFYSPIWFVKIGEVTFLMCLVAFVSTKSTTANSFMRMVSLGQYLLLVLYELFIICYFADVVYQNSQRSGEALWRSPWQRHLREIRSDYMFFMLNARKQFQLTAGKITNLNVDRFRGVS

>CstyOR82a

MAHIFKLQEYCLRAMGHSHEPDSSGSGSGSGGGALSLKHVVSLLFAASAQYPLIHNVVYHRNNMELATAGMSVLLTNLLTLFKIATFLVYKQDFWRMIQSFRQMHQQSSRNIRDGDGYGYVAEANKLASLLGRAYCTSCAATGLYFMVSPILKIIANSWRGTTYVRELPMPLRLPFNYVDSPGYEIGFVYILLVTIVVVSYASAVDGLFISFAINLRAHFQTLQRDIRTTDFSQSEHEFQKALKDLVDYHIRLLTLSKRLRVMYMPIVFGQFFITSLQVGVIIYQLVTNMDSIMALLVYLSFLGSIMLQLFIYCYGGEIIKVESHRVDVAVQLSNWYQAAPKLRRSLAFIILRSQRDLLIKAGFYEASLGNFLAICRAALSFITLIKSIE

>CstyOR74a

MPFHRYRPRLPGGELAPMPWPVSLYRILNHVAWPLEAESELWTVLLDRVMIFLGFLVFCEHNEVDFHYLIANRQDMDNLLTGMPTYLILVELQIRCFQLAWHKDRFRALLQRFYAEIYVSEVMEPLLFARIQRQMLATRVNSTVYLLTLFNFFLVPVTNVIYHRREMLYKQVYPFDNTQLHFFIPLLVLNFWVGFIITSMLFGELNVMGELMMHLNARYVQLGQDLRRSAQRLLKRSSSLNVATGYRLILTHILRRNAALRDFGQRVEDEFTLRIFVMFAFSAGLLCALFFKAFTNPWANVAYIMWFLAKFMELLALGMLGSTLLKTTDELGMMYYTANWEQVIHQSDNVGENVKLMKLVTLAIQLNSRPFFITGLNYFRVSLTAVLKIIQGAFSYFTFLNSMR

>CstyOR69a

MRLKDFMHYPDIGCRLAMMRCYEWFGSEMPRVHQTLAQRLWFRFGALNLIYQNLGMVVYLFMAESQYDKLSTIVAQISETCSVMGLTLVGACNMWMLLQYRSDIECMLADLQQLYPNRGIQSRVYRIEYYFEKSTRLMRYTAIFFISAYGYYNALPVVQLLYELLAESQHVRYQYQSNTWYPWQLLAAPDSSISFIAAYLCQALSSLVGVAFIMISQFLLCFFITQMRLHFDALANGLYYLDARQPGANEHLKVLISYHSRLLCIADKINDIFNFTFLINFTTSTIAICLMAFAMVMISLASTFKYSVGLLSFLVFTLFICYNGTELTSASDKLLPAAFYNNWYDGDLVYRKMLLFFMMRSCESRVLRAYKFTPVSMATYMAMLKFSYQLFTFVRAMI

>CstyOR67d

MSEGPFERYCKINRAIRFCVGLCGNDVIAEDYRMWWLTYAVIGAILFFFGCTGYTVYVGVVLDGDLTVILQAFALVGSAVQGLAKLLVTARMAAVVRQIQATYEAIYREYARRGGDYGRCLERRIKTTWHMLMSFMWVYVVLVGGLIAYPFFHLILHHKKLLVMQFRVPWIDESTDGGYLVLISIHVMLLSMGGFGNFGGDMFLFLFISNVPTLKDIFSAKLREFNEVAVRRQDYQRMRTLLWDLLAWHQQYVSILRDTERIYRIVLFVQLSTNCVSILCTISCIFIGAWPAAPIYLVYSFIVMYSFCGLGTIVETSNEDFSKEIYANCLWYELPVKEQRLVILMLAKSQHEISLTAADVMPLSMSTALQLTKGIYSFSMMLITYLGYES

>CstyOR67c

MSKSLVDTPRTFEDMMRMPVLFYRSIGEDIYAHRSRNPLKSLFLRIYLYAGFINFNLLVIGELVFFYKSLQDFETIRLAIAVAPCIGFSLVADFKQAAMVMHRKLLIELLNELEDMHPKTLEKQRDYKMDQFERTMKRVINIFTFLCLAYTTTFSFYPAIKAAVKYNFLGYEIFDRNFGFLIWFPFDATRNNLIYWITYWDIAHGAYLAGIAFLCADLLLVVVITQLCMHFNYISMRLESHPCKRDGKEADKENIEFLIQMIRYHTKCLNLCEHVNSLYSFSLLLNFLMASMQICFIAFQVTESTLEVILIYCIFLMTSMVQVFLVCYYGDELIAASLRVGDSAYNQKWFQCSKTYCKMLKLLIMRSQRPAAIKPPTFPPISLVTYMKVISMSYQFFALLRTTYKDN

>CstyOR63a

MSQMLKALLLEKQLENNKMLNIFYRISFMTGVNVKYQATFKDPVKLWNGFLIIVSLIGLSAQYCLVWNNRSEPFAESADAICTANQAWISILKLIYLLFVQHEFYDLLHTAINGSLLHDLGIFDLAINSKQYLLKEIKTILNDSWLHIKHQLNFFTFSCMMACGFYMFSCIFANYYFTHIQPQNFTLQLPMPALFPMWHDYGMTWPYYPIQYFITGVENSICGMCAVCFDGIFIVIVVHCSALLEILHKLLEHVCDEEIPQSERVKYLLCCARLHERTFRYYEKINGMYRTPSLAQCVLSMLVLCVVMFMANVGLEEDITLFVKMLCFLCAVGLQIGIYCYNGQKIITQSEISPVAWYSSTWYNESEQFKYIVNMMVLRTNRTLYMQVSGFTTMSLMTLASIVQTSGSYFLLLKNLSGMD

>CstyOR59a.1-2

MTDGTVDSLVFFRSHWTAWRILGLAHHRTKSWRNLYLLYSLVMNVLVTLCYPLHLGMNLFRNGSLTEDILNLTTFATCTACSVKCLIYGYNIRQVMEMERLLRLLDSRVVGQKQLNIYSQVKVQLRNVLYIFIGIYMPCALFAELMFIFKEERGLMYPAWFPFDWLNSTRYYYMANLYQIGGITFQLIQNYVSDCFPAVALCLISSHVKMLYKRFEEVGEDSEKDAEKELEACITDHKNLLELFRRVEAFVSLPMLIQFTVTALNVCIGIAALVFFVTEPMARMYLIFYSMAMPLQIFPTCYFGTDNEYWFGKLHYAAFSCNWHPQNKSFKRKMMLFVEQALKKSTAVAGGMMRIHLDTFFSTLKGAYSLFTIIIRMRK

>CstyOR56a

MPQSGTVKMYKVKELQLTPDSFDNPIFRIHLRCFRWYGYVASMEQRHPWLSLVRCTIFTSSIWLSCALMLLRQFLARGYENLNDGATSWATAVQYFTVSIATLNAYMQRERVVLMLRTAHADLQQLMLEADDQELDLLRTTQRYVRTITFLLWVPSVVAGFMAWSDCIYRTIFLPQTVFNTAAVLRGEAQPILLFKLFPFGELYDNFFIGYLGPWYALSLGITTIPLWHTFITCLMKYVTLKLQILNKRVLAMDIQRLKPELLPQQLTVNELIHWQLQLCKSFVQEQLRIRAFVWQIEQLIRVPVMADFIIFSILICFLFFALTVGVPSKMDYSFMFIYLFVMAAILWVYHWHATLIIECQNELCFAFYAGDWYNFGLPVQRMLLFMMMHAQRPLKMRALLVELNLRTFLDIMRGAYSYFNLLRSSHLY

>CstyOR49b

MFEDIQLIYMNIKILRFWALLYDKNLRRYVCIGLASFHIFTQIVYMMSTNEGLTGIIRNSYMLVLWINTVLRAYLLLADHDRYLALIQKLTEAYYDLLNLNDSYISEILDQVNKVGKLMARGNLFFGMLTSMGFGLYPLSSSERVLPFGSKIPGLNEYESPYYEMWYIFQMLITPMGCCMYIPYTSLIVGLIMFGIVRCKALQHRLRQVALKHPYGDRDPRELREEIIACIRYQQSIIEYMDHINELTTMMFLFELMAFSALLCALLFMLIIVSGTSQLIIVCMYINMILAQILALYWYANELREQNLAVATAAYETEWFTFDVPLRKNILFMMMRAQRPAAILLGNIRPITLELFQNLLNTTYTFFTVLKRVYG

>CstyOR47b

MSEADYKSNVRLVSGFFDEFLSVLKKKSRPPRLVLHYHRACLCLLFIYPNKKMTENSVYRQCNLVILANLTFFLITVMSAIHESKNVIDMGEDFVWIIGISLILTKIFYIYLRADGIDSVIEDFDYYGKLRPHNNDEEILRWQRLCYLAESGIFINCFVLLTLFNLAICVQPLIGGGDLPFHVMYPNNWHRRQIHPKVWSFIYIWQSITSHHNLMSILQIDLLGIHTFLQTALNLKILCLEMRKLGKFGKLNDDQFHQEFSVLVKFHQHVISTVQKNNRVFYGSFITQMIASFALISMSTFETMAAAGDPKVAAKFVLFMVITFIQLSYWCLAGTLVYTQSMEVAQAAFDIQDWHTKSVSIQRDIMFVIQRSQKPLMYVAQPFMPFTLTTYTMILKQCYRILALLRESM

>CstyOR49a

MSEVEKQQKKKMQEKQREYQDFTFLANIMFKTLGYDFLDSARPSWQTGLLRCYFFVCIASSSYEAFFVALECLQVESVAGSPSKIMRRALHFFYMLSAAVKFVTLMIYRKRLRTLILSLKELYPADESLRREYEVNKYYLPRSTRYVFYSYYCFMAVMAIGPLPQSFMMYFLKGHFPFLRTFPTQLCFRSDTPVGYAVAYFMDLTYSQFVVNVSVGADLWMMCVSSQICMHFGYLAKKLAAYLPSREREREDCEFLASLVQKHQLILRLHKEVNQIFGILLASNLFTTASLLCCIGFYTVVEGRSEEGMSYMIIFVVVSAQFYMVSSFGQQLIDLSSSISMAAYSQYWYDGSLRYKKDLLLIMARAQRPAEISAKGIIIISLDTFKILMTITYRFFAAIRQTVGK

>CstyOR43a.2

MEILDCPLLSVNVRVWRFWSFVLVHNWRRYISIIPVTLLNVFMFADLYRAWGNIEEVIINAYFAVLYFNAVLRTLILVYNRDKYERFLAGAAGVYEEICALNDDVITKLVTTYTKRARFLSISNLALGAFISGCFVIYPLFTGQRGLPYGMFIPGVNNFDSPQYEIIYLTQLVLTFPGCCMYIPYTSFFTSSTLFGLVQIKTLQHQLRNFRSENLHETTSTLNRKLQKLIEDHKRIIRYVQDLNDLVTYICLIEFLSFGLMLCALLFLLNIISVMAQIVIVGAYIFMILTQIFAFYWHSNEVREESMAIAEASYSGPWLNVDNAIKKKLLLITIRAQRPLEITVGNVYPMTLEMFQSLLNASYSYFTLLRRVYN

>CstyOR46a

MSKGVEIFYKGQKAFLNILSLWPQIERRWRIIHQVNYVHVIVFWVLLFDLLLVLHVMANLSYMSEVVKAIFILATSAGHTTKLLSIKANNVQMEELFRRLDNEEFRPRGANEELIFAAACERSRKLRDFYGALSFAALSMILIPQFALDWSHLPLKTYNPLGENTGSPAYWLLYCYQCLALSVSCITNIGFDSLCSSLFIFLKCQLDILAVRLDKIGRLITTSGGTVEQQLKENIRYHMTIVELSKTVERLLCKPISVQIFCSVLVLTANFYAIAVLSDERLELFKYVTYQACMLIQIFILCYYAGEVTQRSLDLPHELYKTSWVDWDYRSRRIALLFMQRLHSTLRIRTLNPSLGFDLMLFSSIVNCSYSYFALLKRVNS

>CstyOR43a.1

MEQNAKDIALVAINVEIWRRVAVLYPTPGNNWRKYAFMLPVCLMNLMQFFFLLRMWGDLPAFILNLFFFSAIFNALMRTLLVMFKRLEFEQFLDELATLYRQIEASSDECSHRLLAAAVHEARRLAIFNLTASFLDVSGAVIFAMFLEQRTHPFGVSLPGLDMQRTPIYQIFYVLQVPTPVVLSMMYMPFVSLFAAFALFGKAMLQILAHKLTRIEQLQDEEQRYQMLTACIRIHITVAGYVRKLTTLVTYIVGVEAIIFGSIICSLLFCLNIITSRTQMISIVMYILTMLYVLFTYYNRANDLVIECSRVTQAAYNVPWMECSLRFRQTLLIFLMQTQRPLVIKVGNVYPMTLAMFQSLLNASYSYFTMLRGVTNK

>CstyOR30a.2

MELSSMDTVEMPIFGSTLKLMKFWSYLFVHNWRRYVAMAPYIIINCTQYVDIYLSTESLDFIIRNVYLAVLFTNTVVRGVLLCVQRFSYERFINILKGFYIELLKSDDPAISHLVGETTRLSLFISRINLLMGCCTCIGFVTYPIFGSERVLPYGMYLPTIDEYKYASPYYEIFFVIQAIMAPMGCCMYIPYTNMIVTFTLFAILMCRVLQHKLRSLEKLGNEQVRGEIIWCIKYQLKLAGFVDSMNALNTHLHLVEFLCFGAMLCVLLFSLIIAQTIAQTVIVIAYMVMIFANSVVLYYVANELYFQSFDIAIAAYESNWMDFDVDTQKTLKFLIMRSQKPLAILVGGTYPMNLKMLQSLLNAIYSFFTLLRRVYG

>CstyOR30a.1

MELKSMDPVEMPIFGSTLKLMKFWSYLFVHNWRRYVAMTPYIIINCTQYVDIYLSTESLDFIIRNVYLAVLFTNTVVRGVLLCVQRFSYERFINILKSFYIELLQSDDPIINILVKETTRLSVLISRINLLMGCCTCIGFVTYPIFGSERVLPYGMYLPTIDEYKYASPYYEIFFVIQAIMAPMGCCMYIPYTNMVVTFTLFAILMCRVLQHKLRSLEKLKNEQVRGEIIWCIKYQLKLSGFVDSMNALNTHLHLVEFLCFGAMLCVLLFSLIIAQTIAQTVIVIAYMVMIFANSVVLYYVANELYFQSFDIAIAAYESNWMDFDVDTQKTLKFLIMRSQKPLAILVGGTYPMNLKMLQSLLNAIYSFFTLLRRVYG

>CstyOR42b

MYLRKLFPALFTQPEDSPVRSRDGTLYLLRCVFLMGVRKPPAKFFVAYVLWSFALNFFSTFYQPIGFLTGYISHLSEFSPGEFLTSLQVAFNAWSCSTKVLIVWVLVKRFDEANAILDEMDRRITEPEERLQIHRAVSLSNRIFFFFMAVYMIYATNTFLSAIFIGRPPYQNYYPFLDWRSSTLHLALQAGLEYFAMAGACFQDVCVDCYPVNFVLVLRAHMPIFEKRLRRLGTSPAESQQQRYERLVECIQDHKVILRFVDCLRPVISGTIFVQFLVVGLVLGFTLINIVLFANMGSAIAALSFMAAVLLETTPFCILCNYLTEDCNKLGDALFQSNWIDGEKRYKNALMYFLQKLQQPITFMAMDVFPISVGTNISVTKFSFSVFTLVKQMNIAEKLAKSEMEE

>CstyOR10a

MWSFHRLLRRDQPLRSYFFAVPRLSLDIMGYWPMGDDLPVRAIVHFVILSIGVVTELHAGFVFLQNAKITLALETLCPAGTSAVTLLKMLLMLRYRRDLANVWMQLQRMLFDAGLNRPEQKAIIHENSVLAARINFWPLSAGFFTCTTYNLKPLLIALILYLQDPDQELPWNTPFNMTMPKVLLAAPFFPLTYAFIAYTGYVTIFMFGGCDGFYFEFCVHISSLFQSLQEETRAIFRPFEEYLMLTPAQCARLELQLRGIIIRQNSVFELTSFFRKRYTVITLAHFVSAALVIGFSICNLLTVGNNSLGALLYVAYTVAALSQLLVYCYGGTLVAESSVELSRVAASCPWSLCAPRQRRVILLLILRSQRAPTMAVPFFSPSLNTFASILQTSGSIIALAKSFQ

>CstyOR7a.2

MGQERNLFQKSKPKHVWESRQAFGNLFNCFYALGMQAPDGSYPTRSRVWKRVYRCFAAIMYLWQLLMVPTFFVISYKYTEGMEITQVLTSLQVAIDAVILPAKILALACGLPLLRRGELHLAELDARCVRPEEHRRIAATVRFCNRLVWFYQIAYAIYSASTFVCSFLLGQPPYSLYLPGLDWRRNRRQFCAQAWIEFIIMNWTCLHQATDDVYAVIYLYVLRTHVQLLSERMHHLGKTIQDWDNQPGQKQVQEQQQQENDCDELQRCIQDHQTVLKFLNCISPVISRTIFVQFLITAAIMGTTMINIFIFANTNTKIASTIYLMAVTLQTSPCCYQATSLIDDCQNLSLAIFQCRWIGQNPKFRKLLLIYLHRSQEPMKLTAMKLLPINLATNLSIAKFSFSLYTLIKKMNLGERLNNGN

>CstyOR13a

MFNPQPLRDNGFRIPMQCIWLKLNGCWPLETISQKLRPASLTRKNIYGLAYTLWAWYVIISVGITISFQTAFLVNNFGDIIMITESCCTTLMGALNFVRLMHLRLNQRKFYEIIQQFVVDIWIPNDSNVKVSNECRKWMNTYRVMSVLLSCLILMYCILPLVELFWIVGIDASTKPFPYKMLFPFNPYNNWIPYSLTYIFTAYAGICVVTTLFAEDSIFGFFVTYTCGQFRLLHERIDVLMDSTNVQMCPKSNIKQFHLKQIRELHSIAHHHNKIICFAKLLEDFFNPILLVNLTISALLICMVGFQLVTGKNMFIGDYIKFVVYISSAISQLYILCGNGDTLIQHSTITAYHLYNCGWEGTSKIPYNKEFRTSLEFMILCSQRPVRITAFKFSTLSLQSFAAILSTSMSYFTLLRSLYF

>CstyOR2a

MEELQLDTHRAVRYHWRVWELTGLMQPDGISRLWYLAYSLALNASVTILFPLSLMARLLFTHNMQNLCENLTITITDIVANLKFLNVFLVRRQLHKIRSLLKHLDERARQVNHPEELAALNEAVTTAQKGFQYFARIFTFGTILSCVRVAISSKRQLLYPAWFGVDWENSWRAYVICYGYQLFGLIVQAVQNCASDSYPPAYLCLLTGHMRALELRVRRIGYGTRRLHSAHGELLDCIRDLMLVHRLKGIIQRILSVACMAQFACSAAVQCTVAMHFLYVVDDNDLSAMILSIVFFVAVTLEVFIICYFGERMRTQSEALCDGFYACNWVDQRPIFKRNLIFTLARTQKPSLIYAGGYIPLSLETFEQLMRFTYSAFTLLLRAK

>CstyORco

MQSNLQPTKYVGLVADLMPNIKLMKYSGLFMHAFTGGSPLLKKVYSSIHLVLILAQFMFILVNMALNADEVNELSGNTITALFFTHCVTKFIYLAVNQKNFYRTLNIWNQVNTHPLFAESDARYHSIALAKMRKLFFLVMLTTVASAVAWITITFFGESVKFAFDKETNSSITVEIPRLPIKSFYPWDASQGIFYTISFAFQGYYLLFSMVHSNLCDVLFCSWLIFACEQLQHLKGIMKPLMELSASLDTYRPNSAALFRSLSANSKSELIINEEKEPPSDLDMTGIYSTKADWGAQFRAPTTLQTFNGVNGGNPNGLTKKQEMMVRSAIKYWVERHKHVVRLVAAIGDTYGAALLLHMLTSTIKLTLLAYQATKITGVNVYAFTVIGYLGYALAQVFHFCIFGNRLIEESSSVMEAAYSCHWYDGSEEAKTFVQIVCQQCQKAMSISGAKFFTVSLDLFASVLGAVVTYFMVLVQLK

>CstyOR7a.1

MAVSTRVATKQEVPESRRAFRNLFNCFYALGMQAPDGSRPTTSSTWRRIYACFSVVMYVWQLLLVPTFFVISYRYMGGMEITQVLTSAQVAIDAVILPAKIVALAWNLPLLRRAEHHLAALDARCREQEEFQLILDAVRFCNYLVWFYQICYAIYSSSTFVCAFLLGQPPYALYLPGLDWQRSQMQFCIQAWIEFLIMNWTCLHQASDDVYAVIYLYVVRIQVQLLARRVEKLGTDDSGQVEIYHDERRQEEHCAELQRCIVDHQTMLQLLDCISPVISRTIFVQFLITAAIMGTTMINIFIFANTNTKIASIIYLLAVTLQTAPCCYQATSLMLDNERLALAIFQCQWLGQSARFRKMLLYYLHRAQQPITLTAMKLFPINLATYFSIAKFSFSLYTLIKGMNLGERFNRTN

>BdorOR67c

SQRIGDAAYNQNWFDCDRHYKKLLAIIIMRSQQPASIRAPTFPPISFRTYMKVISMSYQFFALLRTTYSGKGN

>BdorOR46a

MGFHQKLLGLFGLPAHYPSLLQYLYKLYFWHVAIFWMLLFDISMWIKIIGNISNLNEIIKVFYLCSMAIAVMAKFVRIRLKNSSYVALFARIHDEDLLPVNVSELEKFTQSSHLSCRVRNSYMYLSLTSLSLIFVTQLISEPGELPLSIYVPISVENFWCYLIAYLFQFIGLSLCCLLNISFDSLSASFFIYLKGQLDILANRLENIGKDLYVDDNIINLQLRDCIQHYVKLRKIAEIMEDLLSIPMSVQMISSVLVLVANFYAMTFLTDPSDYVTFIKFLVYQLCMLSQIFMLCYFANEVSLRSAELSYSLYSSEWTRCSKINRRLMLLMMAQMDVPIRIKTINRCYSFNLPAFTSIINSSYSYYALLKKMKD

>BdorOR74a

MRYLPISYHKPLLPNGLHPPIDWQLYGFFCANGWPLAAHITKTRYIADIMVTIMQFMSEGMVLIGEAVVMHDNLDNISFVCTVLAPNLILFEMMLRAYNIIYRRNSFRTHIEEFYKKIYIQRTWNPELFEKIRRQQLPTKYSTFTYIITLVTYVYVPVSGLIKNERLVPFPINFGFDYTVPWPRYLVFLTMSMWTGFAVVGPLVAEANILAMQILHLNGRYSLLLEDLRNISRKSIAEHEKCKRKDNMLVTQRFRYRLYDIIRRNVELNDFAKSMQEQYSFRVFVMLALSATLLCVLGFLTATLGITAQNIRFVSWIIGKVVELLIFGRLGTTLSTTTDKLSTSYYCCDWEDIILHSTNAEENKKLMKLIALAIHLNSNPFRLTGLNFSVVNYETVVAILRGAGSYFTVIYAYR

>BdorOR59a.2

MSTSPLSVPQQALAAVDTRSFFKLHWTCFKSPCERRYFVELRKEMRRITFGFLSIYAVVAVTAELMFFFRNEHNLLYPAWFPFDWRASDLKFYAAHSYQIVGISYQLLQNFVNDCLPTMALALLSAHIKLLGMRVSKIGYATESSAANEEELLYCIKDQEQLYNMLNVIQNIISLPMFLQFTVTAINICLPVAALLFYVDAPFDRLYFLVYLLSLPLEIFPICYYGTTFQLLFDKLHVEMFCSNW

>BdorOR85e

MDARDLGGMSLLYSPEDKPRIADLFAAQVIFFKATGQIPFNVGCGLGYIYCCFFITQSLHMAVLFMKTSYDMLLSGKLEEITDALTMTIIFWFSVYAASYWLLRWQRLLDFLQRINQYYWHHSLPGLSFVSAHRTFILAKRMTITWTLTCIAGTVLYGLAPVMGVRTLPLKCWYPFDPLQPYVYELVYVLQFTAQIIMGATFGNGSALYVSLVILMCGQFDVLYCSLKNLSYYGRLRARCDVDKLLDLGGTSLLYSPEDKPRIADLFGAHVIFFKATGQIPFNVGYGLGYIYCCFFVAQSVHMAVLFWKTSYDMLLSGKLEEITDALTMTIIFSFSVYAAFYWLLRLKRLLAFLQRINQYYWHHSLPGLSFVSAYRPFILAKRITIVWVLICVASTALYGLPPLIMGVRALPLKCWYPFDPLQPYVYELVYVLQFTAQLIMGATLSNGSALFVSLVILMCGQFDVLYCSLKNLSYYGRLRARCDVDKLRKEQAALPKPSDDELNQYMYCEEHLTNLSTLQHLYTQKPAATLPEALHLAVVQCVQLHRFILDVCKEMEELFNPFCLVKSIQVTLQLCLLVFVGVAGERSIVRILNLAQYVTLTFIELLMFTYFGELLRGHSVRCGEAFWRSQWWTHTAAIRQDILILLANSKRAVRLTAGKFYAMDVERLRSVVTQAFSFLTLLQKLAAKNPK

>BdorOR2a

MTLKINSWDAFKYHWRVWDLSGFRGPQRQSVWYIPYKLYTIAITLLFPIYYPICFTVESFLADNLNDFCEVIYIAMADMTLNIKFLTLFIVRRQLLELRPILKRLDARAKTEEEMNVLQEGIDSAKKCFLIILRLFYSAFVTSQFMVIFSAEARLMYPAWYPFDYQASRTKFWIAYGYQTIGFLVQCTQACSVDTYPQAYMRVLTAHIRALSLRIERIGRQNFSGVSSELMCSKENEMKRNYEELVSCIKDHKTIIELFSTIQKPISGTSMAQFVCTGVAQCTIGVYMLYVGFNISIMLNMAVFFVSVTMETLILCYYGDLFCQECEELSKAIYNCNWTVQSSEFKKVLCFFLFRSQRVNVLMAGNWIPVRLPTFVMVVKSSYSIFTLLSSFK

>BdorOR7a

MFDLIKGRGRTVFASRDAVIYLFNSFRYLGINPPAKYRLPYFMYSAIITFFAVLFSPVIFNVGWLRDRNKLSVMEILTCVQASLNVMAVPLKCITLAMAQKRLRGIEPMVTELDERFPTLEDKAKIKKCAVTGNRLVFGFAVSYFMYETLTVVSALVGGHAPLSLWIPNVDWHRSTWEYWLQVSFDAAVLFFLLYHQVLNDSYPAVYIYIIRTQVQLLTSRVEKLGYDEQKSVDENYQELLECIVIHQKILKIVKIVESVVSITVFTQFLVAAAILGVTMINIFIFADLTTKIASVTYFFCVLLQTSPTCYHASYLLDDCDQLRIAIFQCNWIAQNKRFNNLLIYFLHRSQDSMPFFALKLVPINLATNLSIAKFSFTLFTFIQEMGLGENLK

>BdorOR7a.1

MFDLVKGRGRSVFASRDAVIYLFNTFRFVGLNPPPHCRLLYYFYGSIITLFVVLLSPLIFNVGWIRDRNILSVMEILNCVQAALNVIGVPIKSITLALSLGRLRSVEPLLSKLDARYTEPEDLAKIRACAITGNRIVFGYIISYMMYETLTVVTALLGGHAPLTLYIPFVDWHRSAWEYWLQSSFDGAMLFFLLFHQILNDSYPAVYIYIIRTQVQLLANRVRRLGTGNKSQEQTYHELQDCIITHQEILRLVSVVEPIISLTLFVQFFIAAAILGTTMINIFIFADFATRIASGAYMFCVLLQTFPTCFYATHLQSDCEQLSMSIFHCNWLSQGKRFNTMLLYFLHRSQADIPLFALKLVPINLSTNVSIAKFSFTLYTFIQKMGVGKNLK

>BdorOR59a

MAFEDVKEIFCTHWTIWKWMGQVNHPKYTKLYKAYSILVNVVFSLGYPVHLVIGLSQEKTIQGSLLNLTISLPSVICVLKFYNTWRNFDKVRHLEQMYNTLYARLDHPEDLAYYRKVTAPNAIRVVSAFKVICVGMAVTAELTQLYVGFVYGWRLMYPGYFPFDPHGSTAGYVTAHIFQFIGLLTQISQNLMSDTYGAVCLALLAGHTHLLGQRLARIGYDKDKTREQHNQDFVDFIVDHNMLLNCQRTLVDIIGMGLFALIISTSLLLAIVIIYPMFFVDNALEYAYYVFFMFGALMEVFPTCYYATHFEYEFEGLTYKIFSCNWVDQNRSFKKNLIVCLEQSLKARYVFVGGMFRINMQIFIAICKGAYSVFTLALNYK

>BdorOR67c.1

MPEARTFSEFIRIPIRFYQTIGEDLYEHRSPHRIRRLLLKSLLYLGFLNFNILVVGEIIYFVKALNSFATVLEATGVAPCIGFSLXSGFQADRLDSAPSNLREHLDQMEESFPKTAIQQVEYKLPQRERVMRRVMAIFALLCLAYTSTFSLYPALKAAVQYWLLGAPVFERNFGFAIWYPYNATEKTWVYWLTYMGQVHGAYLAGVAFLSADLVLVASVTQLCMHFDYISRCLEEFAGRSAKSSAQEDLQYLQALVVKHAKCLELSEHVNSIFSFSLLLNFLTASLTICFIGFQVTASSTEDIVKYIIFLTTMLVQVFVICYYGDELMTSSQRIGDAAYNQNWFDCDRHYKKLLAIIIMRSQKPASIRAPTFPPISFRTYMKVSLNFNQA

>BdorOR67c.2

MMPSFKSSEPAPTVPDFVDIPLFQIKFMGAKLFKWTPDEPRGKLQITLLGTFCVFATFNFTSMLLFVINDELATSLDITEFILFWGFALNAMMKGGTMVCFRRDIEFVLKGLVARHPKTEEEREAFQLVPYFRTINASNKYLSIWHLSITSIFALHPMVSSLLRYIWRDDTNESYDFTFPFMMAYYYDTNQPLTYAVSYFIQCCGAFYMSLLFLSGDLLLISMVQLVNMHFGYLIYKIESFQPTGTDADMRTLGPLLEYHNEILDYAERIDSTFSLATFLNYVGSCLVLCLIGLQIVLGSEALSVIKFIGFLVSTIVQVFFVSYFGNNLKDLSTGISDAFYNHPWYDANYKYMRMLVLPIARSQRYAHLTAFKFFEISMDSFKSLCTTSYQFFTLLRTSMEEEDS

>BdorOR74a.1

MLYRPRLKNGKLIPLSWPVAAYRLLNHICWPLRDDASYLLRLYDRFFWAFGFFIFMQHNDAELRYIIVNNNNLDEMLICGPTYLVLVEIHLRAFQLGLKKEPFKRFLQKFYAEIYIDESSHPKLYANIQKSLRPIWFYSLLYFSTLSSYVIMPVVNYLNNVKAPLYKMYYPFDITPNPIYVAIVLSNIWVGFTVITMVSGEDNVLSEVMLHLNGRFLLLQQKLRHDAERLLHVVDDRNIADGLRRRIIEAIEENVRLYKFAEDFEREFSFRIFVNLSFSAGLLCVLGFKVYTVSRLS

>BdorOR24a

MFLKFLSQSYPTEENVFLIPRFALRIAGFYPGDGNSRRIQAWLIFNFVVLVYGSYAEFMFGIHYLSIDVVRALDALCPVASSIMSVVKLAFLWWHREELERLIKRVTELIATQNSRLKLADKRRYFTIATRLSASVLFFGTTTSTLYTIRAGIVNYLSHLRGEEIPYETPFKMIFPKPLISMPIFPLTFIFSHWHGYITVAGFAGTDGLFLCFCMYIGTLLKALQYDTKDLLSDVGCGERKHSSEAEIMESLKMIIARHNEIIDLVKRFSAVMSGITLGHFVTSSAIIGTCVVDMLLFSDYGVLVYLVHTMAVSTELFLYCLGGTVVIECSSQLATAVYDSNWYTHTVDVQRMVLLIIIRAQRSLVLKVPFFAPSLPALTSILRFTGSLIALAKSVI

>BdorOR67c.3

MTPIFKSSEFVPTVPDFVHIPFFLIKFLGVKLFKWTPDEPITKQQITILGLFTVFSIFNFTSMLLYVVYEDLETLLDITEFVLFWGFTLNALMKGISMVCFRREIESILKGLIAKHPKTAEERAAYQLVPYFRTINISNKYLSIWHLSITSIFVVHPLIASIHGYISREDKNESFDFTLPFMMTYFYDINQPLAYAVSYFLQCCGAFHVSLLFLSGDLLLISMVHLVNMHFGYLIYKIESFQPTGTDADMKVLGPLMVYHNEMLNYAERIDNTFGLATLLNYVGSCLVLCLIGLQIAMGSEAVIVIKFIGFLVSTIVQVFFVSYFGNNLKDLSTGISDAFYNHPWYDANYKYMRMLVLPIARAQRYARLTAFKFFEISMDSFKSLCTTSYQFYTLLRTSIEEDGV

>BdorOR49b

MAAFLDKLKEVYFRVFPSEAGKGQIGSIEFNIWLSQMSGVPLAPWFVPLQRTRLVNGLLLAYDPDMAGQIFPYRVAMPAWLPFFLPVVYIGVTDFMFAVQIVTVDYLNISMMNLLRCHLNIIKSTFDDLILDECHVRRDMKRIRDPNARMADIVEHHCILKSVRDDVEHIFRLSILLQFFTSLVISAVTGFQATMNSSNSNSEIIIYFYCFCIFTQLFGYCWFGNEVNEQNKTLAAHGYGSSWYHFDQRFRKSLAIFLLNAQQPFNFTGGGFVDLSLPSFTNVLSKAYSFIAVLRQMYER

>BdorOR94a

MELHEHDNLSGGRRVIKILKLLGLWHYGGVMRTPYLLYSGLLHSVFTIPYTIMMCMDVVQASDLEKFTNTMYMTLTELGLVAKLVNVWSYSKLLVDFFTAFTHDKLYQLQDAEERQSWQRTQKNYSRVAFLYFTMSLSTLATAFVGVLYSEDYELPFPYAPPFDWRTPRGYWYAYCYELLAMPITCLSNCAFDMIQCYMLLQLSLCFKVISGRLERMGTLQECSSTRGFSEVMFHRDFVDIVRLHARTKLLSQQCQTYISFPFLIQIISSSFVLCFSAYRLQKVPILENPSQFLTLVQANLIMVLQIFIPCYCGNNIIEYSSGLNNATYNAEWFRCSPEMRKYLVIYMEMLQRPVRVRAGDFFDISLTIFTKTMNNTYSLIALLLNMNK

>BdorOR94b

MSLLEEGPVVSLLLRPTLLKPSVAMSSMVSFAACLITICSGVRSSSSSIAAVGQLCITPETETQRLVMDKLEELSSRIFPSDASKGKIGSIQYNVWLAQLFGVPVVGLKAESTRLRIALGIYGLLMTLVVTFFYTGFEVYDMILCWPNLDSLTQNICLSLTHVAGVFKVINIIYRLDEVAFVVRRIEYAAKTYVISKSQLVAFYRGEFENKIPLTIYASLVGFTGVLGLIYLFYNPIGVAGQIFPYRVKLPEWMPFGIQLAYMGMSVLVFALQIVAIDYLNVTMINQIRFQLKILNLAFEELKLDCVNARELEEVNKRLHTIVEHHCLLHDLRNDVEDIFRLPVLLQFFTSLIIFAMTGFQAIVKVENSNGAALIYCYCGCIFCELFVYCWFGNEVSEQSKTLSASGYGSHWYAFDQRFKKSLLIFMCNSQTPFVFTAGGFMSLSLPSFTGILSKS

>BdorOR63a.2

MTLENKEELYKRNYNSIKVLFRVSYTLGVNLTAPDKFKDSLKVIQIILIASSLLSLFAHWWYLKRHIDNIPLIAEAVFTALQIGMAAIKLIYFFFTHRTFYRLLDQTLTHEIIRKIEILTDFPIDRQLRQEIDDIMNRVWLNTRRLFLFYFCCCVGIIANYFFTAFFVNLYHQLKQTPDYEFFLPVPALYPFWEKKGMTFPYYPIQMYMTGAALYVSGLGAVSFEGVFMVLCQHAVALVKVHNLLVQHATSPQIPAERRLEYLRYLIITYRRVSKYLQEIKTIFKHISLVQFLLSLIVMGFVLFEISYGLEASIVIFIRMIMYISASISQITIYCYHGQALTSACEEIPLAYYNCDWYGENKTFKNLILMMIMRTNKEFNMEVSWFTLMNLTTLISLLRASGSYFLLLQNLQED

>BdorOR63a.3

MMSESVEEIYKRNYNSIKVLIGVSFSLGVNLTAPSKIKDALKLFNVIWVVASLLSLYAHWSYFIRHIDNIPLLAETVCTALQTLISAVKMVYYLFTQRTFYRLLEQTLTHEIIRKIEIFERDFPINRQLKQEVDDIMNGVWRSARRQLLFYFCCCVGIVCNYFFGALFVNLYHQLKQTPDYEHILPFPALYPIWEDQGMTFPYYPLQMYLSGSAVYIAGMCAVSFEGVFIVLCQHAVGLVKVHNLLVLRSTSRLIPAERRIEYLRYTIITYQRINIFAQQIQTIFKHVSLSQFVLSLIVFGFVLFEMSFGLESSIVIVIRMIMYFAAGGTQIILYCYNGQQLTSVSEEIPLAFYNCNWYEESGKFKQLLRMMIMRTNRPFNLEVSSFTLMNLATLIALFRMSGSYFLLLRNLQEK

>BdorOR59a.1

MKRPSVVDSRQFFRTHWRMWLLLGCVREPVRYQLLYRLYRSVVNALIILFYPGTVLIALYNSGNVNDLLQTLPICAAALACSAKYISYYRRLNLVRQVEQIFNALDEQIVLEEDRAFFAGIHRGTNLILNTLRGICVFLFAITVMAFADSLENRDLGFAMELPFDWRASTAAYVGAVALELLLLACDLVQSLANDSFPAVALCVLTNHTRLLGARLSRIGHTGKDAQANIRELQQCIIDHQRLYSYYKYERKRLKIVLTSSCVPLTSELAMSQPVNSNAFFKIHWLGFRICGGDLTVCKYRLVYLPYALMVTALVTFCYPLHLALALFRNGSLAGNIKNLAVCVTCIACSLKFLIYTRKLRIMREIEQTFAELDARVSSEEELKYFALMRTSVRNVVAVFVCAYAAVGVTAELAFLLSKERSLLYPAWFPFDWRASTRNFYVANVYQIVGICYQIFQNFIDDTFPPITCCLLSGHIKLLGIRVSRIGYDGVHLADNERELVRCIKDQKNLYKLFDLLQEVMSWPMFIQFTVTAFNICVAMVVMLFYVDTPFERLYYLVYFISMPLQIFPICYYGSSLQFLFGQLQYEVFRCNWPDQTRHFKKQMILFTERALKTTTALAGGMVKIHLDTFFATVKGAYSLFAVIMKVK

>BdorOR63a.4

MYITGCANYIAGMSAVSFEGVFIVLCQHAVGLVKVHNLLVIRSTSPLIPAERRVEYLRYTILTYQRIYNYVQQIQKSFKQVSLSQFVLSLIIFGIILFEMSFGLKSSIFVVIRMIFYILASGTQISLYCINGQHLTTVSEEIPLALYSCNWYEESGKFKQLLRMMIMRTNRHFNLEVSWFTLMNLATLIAFFRMSGSYFLLLRNLQEK

>BdorOR67d

MTIKHIRPTASFAKLVKTVRFISSLVGADVSTVNYQVNIITIIVIICIIMYFIFTATTVASVFSENWTYLLEASCMLGSVLQGITKLISGISRTNEVSGMRLELEELYRVYETKGESYCKVMNACCERVWQLIKMVGLIYGAAIVGNLLLTSFMLFFTNQKIYIMHFFIPGVDVETSFGYLLTTALHSLCFLAGCFGLFGGDLFFLIYLGQPELFRDILILKVHELNEAAAQKDNKTESLLISIIEWHQYYTDYNERCNEIFYYIITMQILTSGVSIVFTMYIILMGDWPGAYLYILIALSSLYLYCIIGTNIQTCNETFFEELYNINWYELDVKERKLMILVLMKSQNPSEIKIGGVLPLSVQTALQITKTIYGIFTMMLGFLDEEQ

>BdorOR67d.1

MTTTKVRPTESFGKIIKFFHLISSLVGADVADENYRVNIITITLIICIVAYFIFTGTTVASVFSENWTYLLEASCMVGSVLQGITKLISAFAFAKEILGIRIELENLYREYEVKGDDYAEALNKSCERVWQVIKMVGQVYFVAGGGIILITIVLIFASNEKVFLMHFMIPGIDVDTQVGYLMTLTLHTMCFLFGAFGLFAGDLFFLLFLGQPMLFLDLLVLKVKSLNEAAAENSSNAERLLIEIIEWHQYYTDYNLRCNRIFYYINSMQIVTSGISIICTLYIILLGDWPGAYLYILVAFGGLYLYCIMGTKIQTCNTAFCEELWNINFYDLEVKNQKMIIPILMKAQNPSEIKVGGFLPLSVQTALQITKTIYGIFTMMLRFLEESQ

>BdorOrco

MQPSKYVGLVADLMPNIRLMKYSGLFMHNFTGGSGLFKKIYSSVHLVLVLVQFLLILVNLALNAEEVNELSGNTITVLFFTHSITKFIYLAVSQKNFYRTLNIWNQVNSHPLFAESDARYHGIALAKMRKLFTLVMLTTVASAVAWTTITFFGESVKFAFEKETNSTITVEIPRLPIKSFYPWNAGTGMFYIISFAFQCYYLLFSMVHANLCDVLFCSWLIFACEQLQHLKGIMKPLMELSASLDTYRPNSAALFRSLSANSKSELINNEEKEPTDLDISGVYSSKADWGAQFRAPSTLQTFNGMNGTNPNGLTRKQEMMVRSAIKYWVERHKHVVRLVAAIGDTYGGALLLHMLTSTIMLTLLAYQATKITGVNVYAFTTIGYLGYALAQVFHFCIFGNRLIEESSSVMEAAYSCHWYDGSEEAKTFVQIVCQQCQKAMSISGAKFFTVSLDLFASVLGAVVTYFMVLVQLK

>BdorOR83a.1

MPNEEINEYYVTDELPFDLDCLPHVPNPAETARPRSFREAFNYALRPCVEHHIFILDVLRELQRLFNFIWLVKTFVVTFFFCISAFNIVKLSEGKTFLKLFSIGHYLFLGLSELFMTCYASEIIYIGSQRCGEALLRSPWHLHLREIRADYLLFLTTTQHAFEFTAGKIYPLRLEKFRAIITTSFSVFTLLRNMDKGE

>BdorOR21a

MAYWAIATRKGQSPPMKITPVLNPNQREFLEDELLYREKLEILAENNTISTDLFVRKFEDIDDPVLLDKHDSFYHTTKSLLVLFQIMGVMPIHRNPQKPGMPRTGYSWTSKQVFWAVCVFSMQTTIVVMVLRERVNTFLNDSDRRFDEAIYNVIFISLLFTNFLLPVASWRHGPQVAIFKNMWTNYQLKFLKVTGSPIVFPNLYPLTWSLCFFSWGVSIAINLSQYYLQPDFKLWYTFAYYPIIAMLNGFCSLWYINCTAFGTASRALSASLELTLMSDKPAKKLTEYRHLWVDLSHMMQQLGRAYSNMYGMYCLVVFFTTIIATYGSLSEIMDHGATYKEVGLFVIVFYCMSLLYIICNEAHYATQSVGLDFQTKLLNVDLTAVDSATQKEVEMFLMAITKNPPIMNLDGYANINRELITSNISFMATYLVVLLQFKITEQRNYSLKQSRAELLA

>BdorOR83a

MSSNEEKQKPDISATVHDGCSTCRMQRRDMFRCIRWHLWFSAMYRLPLERYFPARLRFLAVTLDWTYELFLYSTLLHIDILFICTIYLNQDKGDLELIVNCMIQTVIYTWAIVAKVFFKRIQPKRVKELMRYLNEECRTRSAAGFTYVTFKESVDLSNMWTTVFLICCYAGVTFWLFVPIFNQDRSLPLACWYPIDYKVPVVYEFIYFLQTVGQLQIAAAFGCTSAFYVLIAVIFSGQFDILNCSLKNILATTYIILRKPKSELILLREEQSIADYELNQYYIAKEYRTDFDCIPHFFEKETPKPENFYEGFKIALRPCIAHHRYVLYGLKMLEDLYSNLSFLKYLEVTLLVCLVAFVWVKSTAANSFLRLLSLSQYLLLALWEMFMICYMGEIIFLCSKRCDEALQRSPWHLHSGEIKQDTLFFILNAQRPFRLTGGKMYNLNLKMFRTILTTSFSILTILQNMDLRQPQPK

>BdorOR94a.1

MAINKLANFRTLEPVLTFLGLWEGGDSSWFKRQYRYYQLFMHTTITFTFACLMILEFIYSESLDYAIDVLKYMLVEMAIISKVLNAWYYEQQTAELVNELANSAIFELRTSAEEQMWQKSQKNFRKLTMIYMGTGLNSAFCALLAAALMGAKELPYALWLPYDWRDTYFWGIYCYECIAMPFTCLCNITIDLFQAYLLLHLTLCFRVISMRLERLEDAGKEDAITTELLNNIKMHQRVKELALKCEQVISIALLSQIMLTFLILCFIIYNMQNVKTENDIAQFSENPAHFLAMLQYALIISMEMFLPCYYGNELTVESEKLGFHLYSCDWTAMSAVNRRLIYVYMESLKKPVVLCAGRFFEIGIPIFSKAMNNAYSVLALLLNVNDDEQH

>BdorOR13a

MLFNPKPSKDPKNFRFPLQCIWLKLNGSWPLKPKVTGEFEKYFRLLYTTWAWYVVAMVGITIGFQSAFLLKSFGNIMVTTENGCTTFMGVLNFVRLLHLRLHQRDFQQLLAQFVKDIWITSSSHPTVERACARNMRVFQVISVLQSSLITMYCILPLVELYMLTLNVEPDVLDSMPKPFPYKMLFPYDANHGWRYALTYLFTAWAGVCVVTTLFAEDSLFGFFVSYTCGQFRILHTQIDNIIPDSYAATRAGPGTEAVFQRECIRRLDKIANKHCVLFNFVSRMEEFFSPILLVNFLISSVLICMVGFQLVTGQNMFIGDYVKFLVYILSSLSQLFVLCWNGDNIIQNSLEMANHLYACNWESSVKVAADEETKESFPIVSYSTSAAFRKNLQFMIMRSQRQTCITAMKFSILSLNSFSGLISSSMSYFALLQSFYENEEN

>BdorOR7a.2

MGYVHTLTTEPITIQLSLLQTAANVLGVPLKTISVAILRTHLRKVEPIFDRLDERYQSVAGREQIKDCVMTSSRIFASVFHIVFILTVQTAMDLFPAVYIRTLRTHFNLLTERVSHLGENPEFTDEDNFDELVDCIVTHQELLEAKNIVSSVCSITLFVQFVIAAIALCITLLNFFVFADTVQRVVTLLYYFGVIMQITPTCYQASMMEVDSAKLPDAIFHCNWLAMDKRSRKLIIYFIHRAQEDITFVALKLFNINLTTNLSIVKFGFSLYTFMNNMGFGQNLKELLE

>BdorOR10a

MNFRFLSRTFPLRDYYFYVPKLCLGALGFWPLDTSAPNASNVWAWVNLIILTIGVFTEIHAGCTVLKTDLELALDTLCPAGTSAVTLLKMALIYYYRKDLAWVLKRMRDLVYERDVSINTVKKHIVRAHAVMAARLNFIPFVMGFITCTSYNLKPLLMTLILYMQGQEPMWKLPFNMTMPSFLLHAPYFPLTYIFTAYTGYITIFMYGGCDAFYFEFCSNTAALLELLQNDLKSEYLTFSDQLSLTTEESTVLEWRLVQFIKRHNDIIELTRFFCKCYTVITLAHFVSAGLVIGASIFDLMTFTGFGIVIYIGYTIAVLGQLFIYCYGGSMVAESSVQLATVAFGCDWHACNPRLRRYVLMIIMRSQRAISMSVPFFAPSLITFTSILQTSGSIIALASSFK

>BdorOR33a

MAVLAALSTSNILYPAYVIVDWQNSTWKYLAVFIFQFYGLNMQIVQNLTNDVYGPMILCMLSGHIHLLSRRISRIGHESAAEGDRNYEELVLCIDDYKVLMNTTRQVERIISPSYMVQFTAVGINVVIGLLYLLFFADNLFAYCYYIFHIISIMTEIFPCCYYGSMVQAEFYALSYAIFRSNWISQSRTFRRAAVTFTELSLRDVTVSNLDQRVQNEEDKLYYMKNMRARCSFMINFFTVSYFSVSCMAVLTALTTANILYPAYVIVDWQNSTWKYLAVLVFQTYGLKMQIVQNLTNDVYGPMILCMLSGHIHLLSRRISRIGHERETEGDRNYEELVLCIDEYKVLMNTTRQVERIISPSYMVQFTAVGINVVIGLLYLLFFADNLFAYCYYIFHILAIMIEIFPCCYYGSMVHLEFHALSYAIFRSNWTSQSRTFRRAAVTLTELTLRDVIVSAGGMITLDLDSFFKTCKMGYSIFTVLQSMK

>BdorOR33b

MLPKINTKAIFVRLFFTWRLLGITGWPFNRYLRIVYDFWVNFAVTFGFTGHVIVGFFLSTNKDEFFNSLVISVACINSVMKHYILRYFKQEVWELNEIISQLDDRVRIKEDYDYYKRYIERPCKFMMRFFFSSYSSVSLTALMNGLATGDLLYPGYLPLPWRTSSSAYAACVIYQFYGVSMEIVKNLGNDLYGPLIYCMLSGHVHLLANRVSRVAHDNPENVEDNYKELCECIEDHKMLMNIKTKVERINSAVCMVQFFGVGVSLCIGLIYLLFFADNLFAYIYYSVHSMAIMTELFPCCYFGNMLECEYYDLSYAIFRSNWTTQPRPFRRNVVNFTELTLKEVNMYAGGMFRINLDTFFATCKMGYSFFTVVQSMK

>BdorOR33b.1

MGKRFFIFKSDVDARADSVACFDIFWMCWKLMGIAVNSKKWYITLYDISVNIFVNIYYPIHLTIGLFLVPTVADVLKNLTINITDVACSTKHFLFRCKLPKIREIQRLLKELDERVVAPDERNYFNTGIRNVVRRIMLIFCASYAADVVASAIEVLTKKERELRYPAWFPFDWSANRYTYYAAVLYQTVGVSLQITQNLAHDTFAPVSLCVMAGQVRLLGTRVSKVGYDMSKTLIEHERDLNECIEDHKKLLKIFDLLQDVFWYTQLVQFSSVGLNICLTAVLMLLFVDNLFTYIYYTAYFCSMAVELLPACYYGSKMQEEFQNLPYAIFKCNWIGQRKSFQQNLRIFTELSKKQFTPTAGGIINIHLTSFMATCKMAYSLYTVLMNMK

>BdorOR82a

MPEDLFRIQRNCLRVMGHQDIFDNNEASSSDEQKSKSKRQRRCFRHWQALKYVLLLLFMVSAQLPMMNYIIYHIDDLALATACLSIVFTNVLTVIKTSTFLTYKREFKSLMAEFESMYDELQEAGAKQCLVTVNVGAKRFVKLYFGACTSTGLYFTINPLVSMIWAKFQAKPIPLELPMPMRFPFDFESTPGYEFAYIYTVFITIVVVMHATSVDGLFVSFTTNLRGHFQALQYFIETNTFDKSEALLQRELGIYVQYHVRLLGLAQSVQRVFKPIIFGQFLMTSLQVCVIIYQLVMNMGVIMEMVVYCTFLSSILLQLLIYCYGAEFLKTESSAVSTAIQMSQWYNLPPRHRHVLRLMMLRSQREIIISAGFYEASLANFMSILKAAMSYITFIQSIE

>BdorOR7a.3

MFELITGRGIRNAASKDAFIYFFKGCTIVGISPPKYAGPLYYMWSFLVNTICIVIGPITATVGFVIKYMQNIITTVQFLSGLQASLNLIGLPVKCLTVTSALNRLRGMEPTLAALDARYTRPEDMALIRKAAVMGNRLVFFFGTSYLMYMLFTVIPPLINGKAPLSVWIPFYDEHQSTMHFFGQIVYDLFLMGFVLFHQVLYDSYGSVYIYVISTHLQLLVRRVGRLGTDATKSKDDNLNELVDCVVTHQQILELLATIEPIISKTIFTQFLIISSILCVTMVNMFFFADRSTQIASTLYFLCVLLQTSPCCYFATELKADSEKLPLAIFHCNWPEQDRRFRKVILYFMHHAQLSIELMAMQLFPINVATNISTLHTAVR

>BdorOR7a.4

MSVQPQQQPQQQQLDSAHAFRYLWLNWRLIGMHPTRRHRLPYYIYSACINISLGLFLPATMIAKLFFIENLSQLIGLLYLGVTLTMATAKQWSLWLHRPKLLAVNYYLAKLDVRCMRHAVDRQHIRTAIRICHLYYAGYMFVYELSSSGFAYIGFSLRQLVYDGWFPQLYADAAQNLTVTLIYQNFAVMTFFVLQNVNNDMYPQCYLAVMIGHLRALTARISRIGKDGVLSAEENISELINCIEDHKNLLGYFACIRPVISRTIFMQFGITAFVLCLTAVNYVAFERDAAQMLIAATYIFAVLIEALPCCWYVNSLMEECAQLTTALYNCQWYDQNRRFRKMLIIFMQRSQRTMVLMAGDLVPITLQTFLNIIKFSFSMYTILKG

>BdorOR33b.2

MTNKHKPLHATTTLDTTEAFKYIWSCWRLFGMHRDLYERRLNWIYLILLNLYCGVIYPMLYICSFFTPMDLSQKLANISVAVPIIYTFGKHVVIVYYIREDLPKALAQLKALDRLAESRPEDRAYMQKMVKNCHLVFFVSFVSFWFALLSYGVLEIFRHKLPFEGWVPFDWTRSEAAYVGACAIQLIGLGIETTTAICCDTYAVTYLILLVAHLRVLNGRIERVGSAGATSDAESYRELVACVEYHKECMSYYNSLRPTLSGIYFIQFLSTGLGLSMPAIAFVGGNFSFSHVIKFLIIFGAIIIEVAPCCWFMDEVLVEMRRLTNAMFSCRWYDQNLKFRKALIIFMQRSQIAQPILAGNLIPVSLETFTNIIKFAFSLFTLLNQLNS

>BdorOR42a

MTNQPYSATRLDSSDALRYIWLFWRITGIHPTENSDLIEILTNLSVTVPLIYSSTKHFVVAYHIRSELPKAALHLEALDRRVELEPTACADLKRLVQRCRKIYLVALAGIAVCLVLYALVGISRHKLPFEGWLPFDWEHSLNAYILACAFQLFCLSVQCICALCNDIYPIVYLLLLVAHLRILNARIARIGGVCEEQRSEVVNYQQLTACVRDHWECLKCFSPAIAATIFIQFISTALALCTAAVAFVNADSIGEQLIKFLPYILVVLCESAPCCWLMDEAALEMFKLTNALFSCCWYEQNLPFRRSLIIFMQRSQKIEQILAGNIVPISLLTFIKIIQFAFSLFTLLNQFKK

>BdorOR59b

MQRFSDFIYGRVESDCETNKPFKVLLAFYGLIGLKAKPHGFLPTLHMVFFCIAYAYTPFLAIVGFLRFQKTATVTESLSALQAFINAIFAAAKSVAVLVNFKRFQSVEPIMKSLDERYKTPQERQQITDCVADCTRLYAAMGFIYYLYGLISILTALIIHKQPFGGWYPFLDWISNPTVHFYSCVAFETWYLYFLLTAQYLHDVYPTLYMRTIRAHMQLLRERIGRIGVDPEKSVDENNKELIDCIATHQQILQ

>BdorOR7a.6

MMANVPSSTVQLEASIAALSRAESTDKPAVRTVQATNYLFKGFRVLGIYMPERRKWLYSLYSLIPNTLVTLWLPLSFVFSYATMSTEDLVPSSLLTSIQVAINVIGCSVKIVVMAFLLPKLRTANVYMDRLDVRCRVEEEIAELRKIVQQGNRFVVLFAMSYWSYASSTFLGSVVFGRPPYALYNPIIDWRKSKLEFITASLMEFALMDVACFQQVVDDSYAVIYVCILRTHMRILLMRLKRLATSAETNLEENLEELKLCIIDHKNLLGLYDVVAPIISVTIFIQFMITASILSATLINIFIFADQLSAQIACCFYILAVVVEIFPLCYFAQCLMDDSERLSQQIFHSNWIAQDVRFRKMLVFFMQRTQRVMELNAGKIFPITLGSFLNIAKFSFSLYMLIKKMGIRERLGLE

>BdorOR92a

MNAIERNTNFTRFTAGPVRYFKFLGILLQQPEMPHSKYQRLLTVVTIALMFLHQIGYILEPGRTFAEQSAAAGLLNYTTVSGGKILFLVYNRRLLLSNHCQLAALYPSAAVERHYKLEHYLRIYAHVQTLLYNFFKYILIVYITYPIVQSFYDLWSSGVYSYIMPTLFWYPVPLEQSLFVYIVYLLFACFCSFCAGLIILSADLCLFSSVSQLMLHLDLLAQRIKELQPAEEGSLSALKAIIEYHQKILTIAKDVNSIFAPSILFSLASSSFILCFSAYQLLDDVSFIFALKVFLLLGYEMKQVVITCYYGDKLMDSSANLFTAVYAHNWTDGSPVYKRLVLFMLVRTYRPIALKVAGISDVSLITLKQVLSTAYQIFTVLKTT

>BdorOR7a.5

MKFVLEKLRATQILTQRLDKRCRASDEVEELRQMVRFGKKVVIFYLTIFLCYSASTFLASVSSGYPPYSLYFPFLKWRRSRTEFIIASLLEFIIMDFACLQQTVNDGYPVIYINMLRCHMKILQFRVEKLGTNPMLTQVEHLSELKLCIKDHQLLIELYDTIAPIISITLFIQFALSAVCIGTALINIVIFANEFQTQVACSFFILAVLIEIYPACYFSQCLINESDKLADVIFHSNWIEQSPEYRKLIIFFLQRSQRPMFLTAGKLFPVTLSSFVSIAKFSFSLYTFIEKMNLKERFGIE

>BdorOR85c

MSNIIRFEAFLRIPSFFYRSVGVDLWNTNGGPLQNAVFYISLFNVNVWLLSELIFAVLMLTKNFIQATMTLSYAGFVLVGSIKMYFMWRKKAEMTRFLQLMNTIFPRTETQQKKMNLRSHLRQCTIVMTVFAMIFMILIWTYNLYPYMQRQIYDCWLHMRSINKTLPYESYIPWNWHDHWTFYLYYTLQSIAGYHSASGQIASDLVLCAMATQIIMHYEYVAQRITEYQPQALRAPRHQVKESESYRKDMEFLCDIIAYHANILSLSDIMNEVLGVPLLVNFMTSSFVICFVGFQMTMDAEPDYMVKLFFFLFSSLIQIYLICHYGQQLIDASSNVSRAVYNHDWIHSHVHYQRMLVLVTARAQKPAMLKATSFVRISRGTLTDIMQISYKFFTLVRTMYSN

>BdorOR85c.1

MSTIIKFEKFLELASFFCYNIGIKLWGPNDGFWLNFWLYLTSINLFLTVFAECIYIIMTIRSDFIVAIMTLSYVSFIVVAYVKWYYLYNYQTERNAFFQRLDALFPHTKSEQESIKLSEYFRLNKLATRGYTITFMVVIWIYNLYTISQRFIYTQLLHVHIERVLPYQAMYPWDWRDNWTYYVIYVTQGFAGFHATCAQIAYDLLLCILSIQLIMHYDHISRSLEEYQTKFAEVHGIDINNGLPPLMCAAVELRAVKEDIKFISNIVSYHNELLSLSMSLNKLFGMPLFVNFFTSSAIICFLSFQMSVTREVDLLMKLAVFLFFSVMQVYLICHFGQLLSDASTNVASAAYFQDWSYADVRFQKMTILVAQRAQEAAALKATNFITISLDTMTVIMQISYKFFTVLRTMYAD

>BdorOR85d

MSDQIIHFESFNTLANIFYTSIGLDAYQKAGQRTNNIRRQLLSIFFIITIANMNITLLSELLYIFMAFAKNNNFVEATMLSSFVGFVIVGDFKIYSIWRQRARITAMMQALHALYPRTLAEQIKYEVQRALQRYQRFAYAFVLLHELLVWSYNLFPLLNYFIYEVWLAARVVGKTLPYNCWTPFDWHVNDWRYYPMYLTQIAAGQACLSGQLANDLLLSAVAVQLIMHYRQLARRIELHVAGGGGGSGSKWRTAATNVCREQDLRFLRSVIAYHQQILNLSQALNDVFGISLFISFASTALIICFVLFQITIGANIDAIIMLAFFLFCSLVQIFLICYYAQQILEASEYISYAVYNHNWFDSDLRYRKMLIYIMARAQKPSKLQATALVIVSMPTMTDLLQLSYKGFAVIRTMYAREPKNFTK

>BdorOR85d.1

MIEFGAFMSTANFWYSFNGIVAYDDIYRQPGDAPKQKSFAARFTTPLRQIYSLIGLVNLIWVLIIEASFVVVNFIENSDFLQAARNFTFMGFVIVSILKILSNLRQRSRISILMRKLYEIYPKQSTDQPPYELQSHLSHYRRIGFMHAFTHAFTVGTYNFLPMINYLFLAPLLQHTDVVRELPYYCWVPFEWRDNWLYYPLYVSQVCASLTGLGGYLASDLLFCAATVQLIIHFRKLARDIEAYQAGCSCATADVCTQQAQRDLDFLSAAVYYHSHTLALCQLINEIFGLPVLINFISTSFVICFLAFQFSIGVPLDSMVALVSYMICCLVQFYMICSYGQELITTSENIGHAVYNHNWLVADIRYKKMLIMIIRRAQKPAILKATTFVNISMGTLTDLLQLSYKFFALIRTMYAR

>BdorOR47b

MSAPRSSCGPYLPNSRAIAINRFGQANKISGSGYLAANVQCGVLLGLWPAKRAAENQLYYFYNLLIMVLFSFFMATIICDLYEASSDFVLLGEDLVVVLGIILDSDIIYTSELLAMKTYPKVLQTYEKRGNNAMSLQPLLSQQTLPFRCKFPFGLNDPDEHPIAFVCVYFFQCFCTLYMLVAIVVMDSLGGNSFNQTTLNLRILCENIRHLGIVAAGASSSTSEAVAWRELREAVEFHQKIIGLMNRINQTFYWNYVSQMGASTFMICLTAFEALLAQDKPMVAMKFQTYMFSAFMQLLYWCWMGNRTYYDSMEVATAAYEIRAWYEHSPLLQRQLMFIIKRAQKPLEFRAKPLFGFTFASFTSILSTSYSYFALLRTMSD

>BdorOR22c

MRRLLGPQVPIERSFFRIPRFSARVAGFWPQSTNRHRSWLTALRFYVNTFAVAVGGFGEVSYGFVYLHDLFSALEAFCPGITKVISLLKMTIFFGRHKRWQHVINSMHQLLLLDTSAEKRRIVESLASFGSALSFVLLLSGSLTNTFFNILPLLKMGYYKWQSLEVELLLPFNVILPEMFVNWPYYPATYLVLTLSGAMTVFTFSAVDGFFLCACVYTSALFRMLQHDIRNAFAELQELEHSTLAQNMRIQHRLAVLVERHNKIIDLCSDFASEFSLIILMHFLSASLVLCFSILDLLLNSSSVGVLTYIFYSIAALTQLILYCIGGTYVSESSLKVAEVIYDTDWYKCDVRTRRMLLLMICRAQKAKTIQVPFFTPSLPAFRSIVSTAGSYITLLKTFI

>BdorOR7a.7

MSKILLVRSATVYKSRDALTYLFNVFTFMGTNPLENRSQRYYRLYHFYSFTVNFICCLFCPLSFHIGYIKLRHVLTNSQLLAAIQNAVQVSGIPIKILVITWYMKRLRHAFEILDELDVNYTRREDLAKIRECVRRCKKIVLIFCFPYYSFELTTIALGVAQNRAPLAAWVPFLDGQRAAWEYWTIVLWDAFVMFFLLCHQLGSDTYPPIFINIIRTHVQLLIARVNRLGRTGALTADEHYEELLGCIRTHVQIVSIAKIVAPVISVTLFTQFATTATTLLNWLGNVEYPENIISLAFFSCQLLQILPCCSSASQLIADCERLPDAIFHCNWVDQDRRFRRAMLFFLQRAQNPIRFSCLKLFNVKLETSVAIGKFAFSLYTLIEETKVGTDTEN

>BdorOR43a

MCKTWYEDIGETTMNFHRLLLITHCLIRFCIIVKKKNKFERFFQCIEQWHREIERNDDPQMVGILQEITKRTQLLSKMTIYVAAGGTLAAIVYPLSFDERINVKIFLQLGLIGRSTQTKQILLAFVPVATYLGQIINLYKTWGGVIGETGMNFYMLAHITHCLVRLLMVVRNNERFMCFLQSTDRWYEDIELNSDAEVVLMLQDVTTHTHKLTRIGFYTITIGALFSYIYPFSFEERTYAAYDTPWYEGNMELRKCVQIMIARSHKPLEIKSSGLYPMTLENFQAILRISYSYFSML

>BdorOR2

MLTDDDILAVLEASDAPDFSNDGEERDKNYEPSDSTDSEGEEEADIEGNSLEYSYNSTMTSYENLPLYLMNVKVFVKMGLIDSSGWIKRFLYGLILITSFVGQMINVSKTWSEDIGDTSMNFYCLLLVTHCLIRFSIVVKKAEKFERFFQCIKQWYTDIEREGDPQMVGTIQEITRKTQKLSKVTIYVAALATLAAFCYPLSFDERKHMIEVQYLFFDILQTPYYELFYLMEVVLVTPTILVLYLPFTNILLISLMFGELALKDLCVKLRNIRSENEETMLQEFKECIEYHGKVVDFNIYMIGITYYYANNLATESLEVANAIYDTPWYRGNLEMRKSVITMIARCQKPLQKPKDFERFFQCVEQWYRDIERNGDPQMVGTLKEITKRTQLLSKMTIYVAAGGTIAAFFYPLSFDRRKHMITVQYPFVDALQTPFFEFLFLLQVLCLAPIILVLTLPFTNIYLISLMFGELVLKDLCVKLRNIRSENEETMLQEFKKCIAYHQKIIALCDDLQDLLSMDGFFHVALFGMMLCMLLFFLSMIHDLRLILTILTFVSYTTYMLFTTYYYANNLATESLEVANAAYDTPWYRGNLEMRKCVITMIARCQKPLQMKAGGLYPMTMETFQAILRVSYSYFSLLQGLSQQ

>BdorOR94a.2

MWAQHIIKLELKMTPSAAEQQERIGVARVLMHFLQILGAWPILPEHHHQNASSTQCRTWLARNYRYLLHLPLTFTYNTLMWVEALTRWERADHILYISITEVGMMALTLNFWRLDQRAYHFMHELCYSDHLALRNQAERQWWRAKQRSFTRIAVCYIGGGAGVLCTAFGATLLVNGYSLPYDYWLPFEWHNAQNYWYAYGYELVAMSLTCIANVTMDMMLCYYLFHVALLYKLIGMRLMALQHLSERLAVQQLINIIELHKRVKRLTAQCEVLVSLPILVQIVLSVFILCLSAYRLQSMQINENPGQFFAMLQFASVLTLQIFLPCYFANEITINSDALTTCVYNSNWEEFSPPTRKLMNLYMELMKRPEQIKAGNFFLVGLPVFTKTMNNAYSLLALLLNMSK

>BdorOR30a

MVMISTFLVFWNENIAKAAYNCNWENRNKEFRKYIPLIIITSQRPLQLTAGGLKPINMEFFLTIVRCTYSFFTVLFTMTTEGDS

>BdorOR49a

MDFVQFFWFPNALYRIVGYDFQQLPRAHWRKALMKAFLIFTTISGICTRIYMLFQLRELILSGDILNSFRLGVYISYAIDSNVKFFVFLLNAKRLRVIYQSLSNEYPMTSMEQKLYQVDKYSFKRARIMIVSYLSVTNSILIGPMLQSIFMYIIDLFRYGYAAAAFSYLHPTPMSYNFNYCTPHYYILIYISEYLNGHFCTTTNLGTDLYVCTFAGQFCMQLEYLGSSLEAYEPSMDNSKADCKFLMEWIRKHQLMLDLCSELNEVFGTTLLFKLISNCAVFCIIVVQLKLEGFGFGFLNFLSFFFVTVAQFFMVCQYGQKLITISENLALCAYKNRWYNGSQTYKTLLFNIIARAQKPARLTAKGFQPISLATFQIVMTMTYRVFAVLQRALD

>BdorOR45a

MTTRSARVIKIYFLTLQIIALATILIPIAVYSWQHIQEIVEVTNAMAPFMQATISLWKIWRVIYRRKEMAQMAENIYLISTRASAKELTHLIQENNRERLMNTAYYYSVLNTGMLALAAPVLVSFIQYLRLGEFSYIVVLKATYPIEYARPLNYFLIWLWTAVAIYGVIYGSVSVDSLYSWYIHNLVGNFKILQSKLVTAESASELSERRELIYYCIAYHQRIIAMTEQLNIIYQPIVFVQFSLNALQICFLAYQIGSGVVDTVDLPFLFLFMISVGIQLMIYCYGGQHLQNESVNVSKSIYQTINSSSWPNELRKVLLISMMRAQKPSKLTGIFFDVDLPLFLWVWRTAGSYVTLLRSVDQKTM

>BdorOR1a

MENFADVDKIVAAMTINVQLFTTSGKNFIFLARRKRFLRLNEALERLALKGNKYERELWNATNRPVLPITTAYSISCQLTVNICLLLPIFKLLFYYIWYNEVVLTLPLPGIFPYDYTLPFYYILTTILTVLLVQLCANTITVVDGLFGWFVYNISAHLQIMRLKLEQLLQLHVDDPNFHLTSASLPICFLAYQLSYLSDPANVPFICLLLSSIVLQLMIYCFGGEKVQNECDELSQNIYLLIPWHKLPPKHCRLLLIPFIRSQRVLVLTGYFFTANRSLLVWIFRTAGSFTAMLFALKEKEV

>BdorOR47a

MYEYLRIQQFSFRVIGINLWAQRDQRIASAPCRYYSWTLATAIITLFMGFYIYTSEQDKAIQVLTVFLQGVLSVFKSGMFVAKGRRFIKLIRSLDMLAAEANVKEGKEWKHENDWQQRIARVYYSCCMSTGTLYCAVPAIILLYSQCFNGHATFILPFDAAFPFDTAHPFFYPISYIWCISFIIYAVHAIAAMDSLFCWFIFNISAHFRALQRAVETVGAAMTGAEDYASLHGRITRTLHYHRRIIELSAEFDELYAPIVFIEISVSYLKLCFSAYNLINLAAAAANTFQTSPSVSQATDYLKSLNLLNISCVAASECIGAAKRNLQQVAEPIKLNANARHLAITGQSAVLACE

>BdorGROR63a

MFNSYNRRKKHDTVFLNVKPTFNGQGNGLRKYSTGLLDKEDNPFYDVNSSSGSRASVGTITTLNENFRANIFYNNIAPIQWFLHMLGVLPITRREPGKAKFRINSIAFGYSFAFFTLLSVFVTYVAKNRISIVTSLSGPFEEAVIAYLFLVNILPLILIPILWWEARKIAKLWNDWDDFEILYYQISGHSMPLNLRRKTTMIAVVLPILSILSVVITHITMADFQIIQVIPYCILDNLSAMLGAWWFIICESLSMTANILGERFQRALRHIGPAAMVADYRALWLRLSKLTRDTGNATCYTFTFINLYLFFIITLSVYGLMSQLSEGFGIKDIGLAITAIWNVFLLFYICDKAHYASFNVRTNFQKKLLMVELNWMNSDAQTEINMFIRATEMNPSNINCGGFFDVNRNLFKGLLTTMVTYLVVLLQFQISIPTDTGRHMNVSVAELATDMMLESAEDELTTTSTTSTTTTTTTKMPPPARGRKG

>BdorOR7a.8

MGYLHTLETEPITIQLGILQAIFNILGLPMKAIVITILLTHLRSAELIFSRLDARYQSIASREQIKNCVIISTRLLSSVIFVFHFYGSATYLQALLTNGYPLNTWLPFTDYIPQPTIRYWAHFIFEVFHLIFLLTVQATMDAFPAVYIRNLRTHLNLLTERVSHLGENAELTEEENFEELVDCIVTHQEFLEAKNIVESVCSITLFIQFVIVAVALCVSMLNFFVFADRQQQVVTVTYYLGVMLQIMPTCYQASMIEADSAKLPDAIFHCNWLAMDKRCRKLIIYFIHRAQEDITFVALKLFNINLTTNLSIVKFAFSLYTWMSNMGFGQNLKDLLE

>DmOr1a

MSKLIEVFLGNLWTQRFTFARMGLDLQPDKKGNVLRSPLLYCIMCLTTSFELCTVCAFMVQNRNQIVLCSEALMHGLQMVSSLLKMAIFLAKSHDLVDLIQQIQSPFTEEDLVGTEWRSQNQRGQLMAAIYFMMCAGTSVSFLLMPVALTMLKYHSTGEFAPVSSFRVLLPYDVTQPHVYAMDCCLMVFVLSFFCCSTTGVDTLYGWCALGVSLQYRRLGQQLKRIPSCFNPSRSDFGLSGIFVEHARLLKIVQHFNYSFMEIAFVEVVIICGLYCSVICQYIMPHTNQNFAFLGFFSLVVTTQLCIYLFGAEQVRLEAERFSRLLYEVIPWQNLPPKHRKLFLFPIERAQRETVLGAYFFELGRPLLVWIFRTAGSFTTLMNALYAKYETH

>DmOr2a

MEKQEDFKLNTHSAVYYHWRVWELTGLMRPPGVSSLLYVVYSITVNLVVTVLFPLSLLARLLFTTNMAGLCENLTITITDIVANLKFANVYMVRKQLHEIRSLLRLMDARARLVGDPEEISALRKEVNIAQGTFRTFASIFVFGTTLSCVRVVVRPDRELLYPAWFGVDWMHSTRNYVLINIYQLFGLIVQAIQNCASDSYPPAFLCLLTGHMRALELRVRRIGCRTEKSNKGQTYEAWREEVYQELIECIRDLARVHRLREIIQRVLSVPCMAQFVCSAAVQCTVAMHFLYVADDHDHTAMIISIVFFSAVTLEVFVICYFGDRMRTQSEALCDAFYDCNWIEQLPKFKRELLFTLARTQRPSLIYAGNYIALSLETFEQVMRFTYSVFTLLLRAK

>DmOr7a

MAVSTRVATKQEVPESRRAFRNLFNCFYALGMQAPDGSRPTTSSTWQRIYACFSVVMYVWQLLLVPTFFVISYRYMGGMEITQVLTSAQVAIDAVILPAKIVALAWNLPLLRRAEHHLAALDARCREQEEFQLILDAVRFCNYLVWFYQICYAIYSSSTFVCAFLLGQPPYALYLPGLDWQRSQMQFCIQAWIEFLIMNWTCLHQASDDVYAVIYLYVVRIQVQLLARRVEKLGTDDSGQVEIYPDERRQEEHCAELQRCIVDHQTMLQLLDCISPVISRTIFVQFLITAAIMGTTMINIFIFANTNTKIASIIYLLAVTLQTAPCCYQATSLMLDNERLALAIFQCQWLGQSARFRKMLLYYLHRAQQPITLTAMKLFPINLATYFSIAKFSFSLYTLIKGMNLGERFNRTN

>DmOr9a

MSDKVKGKKQEEKDQSLRVQILVYRCMGIDLWSPTMANDRPWLTFVTMGPLFLFMVPMFLAAHEYITQVSLLSDTLGSTFASMLTLVKFLLFCYHRKEFVGLIYHIRAILAKEIEVWPDAREIIEVENQSDQMLSLTYTRCFGLAGIFAALKPFVGIILSSIRGDEIHLELPHNGVYPYDLQVVMFYVPTYLWNVMASYSAVTMALCVDSLLFFFTYNVCAIFKIAKHRMIHLPAVGGKEELEGLVQVLLLHQKGLQIADHIADKYRPLIFLQFFLSALQICFIGFQVADLFPNPQSLYFIAFVGSLLIALFIYSKCGENIKSASLDFGNGLYETNWTDFSPPTKRALLIAAMRAQRPCQMKGYFFEASMATFSTIVRSAVSYIMMLRSFNA

>DmOr10a

MSEWLRFLKRDQQLDVYFFAVPRLSLDIMGYWPGKTGDTWPWRSLIHFAILAIGVATELHAGMCFLDRQQITLALETLCPAGTSAVTLLKMFLMLRFRQDLSIMWNRLRGLLFDPNWERPEQRDIRLKHSAMAARINFWPLSAGFFTCTTYNLKPILIAMILYLQNRYEDFVWFTPFNMTMPKVLLNYPFFPLTYIFIAYTGYVTIFMFGGCDGFYFEFCAHLSALFEVLQAEIESMFRPYTDHLELSPVQLYILEQKMRSVIIRHNAIIDLTRFFRDRYTIITLAHFVSAAMVIGFSMVNLLTLGNNGLGAMLYVAYTVAALSQLLVYCYGGTLVAESSTGLCRAMFSCPWQLFKPKQRRLVQLLILRSQRPVSMAVPFFSPSLATFAAILQTSGSIIALVKSFQ

>DmOr13a

MFYSYPYKALSFPIQCVWLKLNGSWPLTESSRPWRSQSLLATAYIVWAWYVIASVGITISYQTAFLLNNLSDIIITTENCCTTFMGVLNFVRLIHLRLNQRKFRQLIENFSYEIWIPNSSKNNVAAECRRRMVTFSIMTSLLACLIIMYCVLPLVEIFFGPAFDAQNKPFPYKMIFPYDAQSSWIRYVMTYIFTSYAGICVVTTLFAEDTILGFFITYTCGQFHLLHQRIAGLFAGSNAELAESIQLERLKRIVEKHNNIISFAKRLEDFFNPILLANLMISSVLICMVGFQIVTGKNMFIGDYVKFIIYISSALSQLYVLCENGDALIKQSTLTAQILYECQWEGSDRIEIQSFTPTTKRIRNQIWFMILCSQQPVRITAFKFSTLSLQSFTAILSTSISYFTLLRSVYFDDEKKLD

>DmOr19a

MDISKVDSTRALVNHWRIFRIMGIHPPGKRTFWGRHYTAYSMVWNVTFHICIWVSFSVNLLQSNSLETFCESLCVTMPHTLYMLKLINVRRMRGQMISSHWLLRLLDKRLGCDDERQIIMAGIERAEFIFRTIFRGLACTVVLGIIYISASSEPTLMYPTWIPWNWRDSTSAYLATAMLHTTALMANATLVLNLSSYPGTYLILVSVHTKALALRVSKLGYGAPLPAVRMQAILVGYIHDHQIILRLFKSLERSLSMTCFLQFFSTACAQCTICYFLLFGNVGIMRFMNMLFLLVILTTETLLLCYTAELPCKEGESLLTAVYSCNWLSQSVNFRRLLLLMLARCQIPMILVSGVIVPISMKTFTVMIKGAYTMLTLLNEIRKTSLE

>DmOr19b

MDISKVDSTRALVNHWRIFRIMGIHPPGKRTFWGRHYTAYSMVWNVTFHICIWVSFSVNLLQSNSLETFCESLCVTMPHTLYMLKLINVRRMRGEMISSHWLLRLLDKRLGCADERQIIMAGIERAEFIFRTIFRGLACTVVLGIIYISASSEPTLMYPTWIPWNWKDSTSAYLATAMLHTTALMANATLVLNLSSYPGTYLILVSVHTKALALRVSKLGYGAPLPAVRMQAILVGYIHDHQIILRLFKSLERSLSMTCFLQFFSTACAQCTICYFLLFGNVGIMRFMNMLFLLVILTTETLLLCYTAELPCKEGESLLTAVYSCNWLSQSVNFRRLLLLMLARCQIPMILVSGVIVPISMKTFTVMIKGAYTMLTLLNEIRKTSLE

>DmOr22a

MLSKFFPHIKEKPLSERVKSRDAFIYLDRVMWSFGWTEPENKRWILPYKLWLAFVNIVMLILLPISISIEYLHRFKTFSAGEFLSSLEIGVNMYGSSFKCAFTLIGFKKRQEAKVLLDQLDKRCLSDKERSTVHRYVAMGNFFDILYHIFYSTFVVMNFPYFLLERRHAWRMYFPYIDSDEQFYISSIAECFLMTEAIYMDLCTDVCPLISMLMARCHISLLKQRLRNLRSKPGRTEDEYLEELTECIRDHRLLLDYVDALRPVFSGTIFVQFLLIGTVLGLSMINLMFFSTFWTGVATCLFMFDVSMETFPFCYLCNMIIDDCQEMSNCLFQSDWTSADRRYKSTLVYFLHNLQQPITLTAGGVFPISMQTNLAMVKLAFSVVTVIKQFNLAERFQ

>DmOr22b

MLSQFFPHIKEKPLSERVKSRDAFVYLDRVMWSFGWTVPENKRWDLHYKLWSTFVTLLIFILLPISVSVEYIQRFKTFSAGEFLSSIQIGVNMYGSSFKSYLTMMGYKKRQEAKMSLDELDKRCVCDEERTIVHRHVALGNFCYIFYHIAYTSFLISNFLSFIMKRIHAWRMYFPYVDPEKQFYISSIAEVILRGWAVFMDLCTDVCPLISMVIARCHITLLKQRLRNLRSEPGRTEDEYLKELADCVRDHRLILDYVDALRSVFSGTIFVQFLLIGIVLGLSMINIMFFSTLSTGVAVVLFMSCVSMQTFPFCYLCNMIMDDCQEMADSLFQSDWTSADRRYKSTLVYFLHNLQQPIILTAGGVFPISMQTNLNMVKLAFTVVTIVKQFNLAEKFQ

>DmOr22c

MTDSGQPAIADHFYRIPRISGLIVGLWPQRIRGGGGRPWHAHLLFVFAFAMVVVGAVGEVSYGCVHLDNLVVALEAFCPGTTKAVCVLKLWVFFRSNRRWAELVQRLRAILWESRRQEAQRMLVGLATTANRLSLLLLSSGTATNAAFTLQPLIMGLYRWIVQLPGQTELPFNIILPSFAVQPGVFPLTYVLLTASGACTVFAFSFVDGFFICSCLYICGAFRLVQQDIRRIFADLHGDSVDVFTEEMNAEVRHRLAQVVERHNAIIDFCTDLTRQFTVIVLMHFLSAAFVLCSTILDIMLNTSSLSGLTYICYIIAALTQLFLYCFGGNHVSESSAAVADVLYDMEWYKCDARTRKVILMILRRSQRAKTIAVPFFTPSLPALRSILSTAGSYITLLKTFL

>DmOr23a

MKLSETLKIDYFRVQLNAWRICGALDLSEGRYWSWSMLLCILVYLPTPMLLRGVYSFEDPVENNFSLSLTVTSLSNLMKFCMYVAQLTKMVEVQSLIGQLDARVSGESQSERHRNMTEHLLRMSKLFQITYAVVFIIAAVPFVFETELSLPMPMWFPFDWKNSMVAYIGALVFQEIGYVFQIMQCFAADSFPPLVLYLISEQCQLLILRISEIGYGYKTLEENEQDLVNCIRDQNALYRLLDVTKSLVSYPMMVQFMVIGINIAITLFVLIFYVETLYDRIYYLCFLLGITVQTYPLCYYGTMVQESFAELHYAVFCSNWVDQSASYRGHMLILAERTKRMQLLLAGNLVPIHLSTYVACWKGAYSFFTLMADRDGLGS

>DmOr24a

MERHYFMVPKFALSLIGFYPEQKRTVLVKLWSFFNFFILTYGCYAEAYYGIHYIPINIATALDALCPVASSILSLVKMVAIWWYQDELRSLIERVRFLTEQQKSKRKLGYKKRFYTLATQLTFLLLCCGFCTSTSYSVRHLIDNILRRTHGKDWIYETPFKMMFPDLLLRLPLYPITYILVHWHGYITVVCFVGADGFFLGFCLYFTVLLLCLQDDVCDLLEVENIEKSPSEAEEARIVREMEKLVDRHNEVAELTERLSGVMVEITLAHFVTSSLIIGTSVVDILLFSGLGIIVYVVYTCAVGVEIFLYCLGGSHIMEACSNLARSTFSSHWYGHSVRVQKMTLLMVARAQRVLTIKIPFFSPSLETLTSILRFTGSLIALAKSVI

>DmOr30a

MELKSMDPVEMPIFGSTLKLMKFWSYLFVHNWRRYVAMTPYIIINCTQYVDIYLSTESLDFIIRNVYLAVLFTNTVVRGVLLCVQRFSYERFINILKSFYIELLQSDDPIINILVKETTRLSVLISRINLLMGCCTCIGFVTYPIFGSERVLPYGMYLPTIDEYKYASPYYEIFFVIQAIMAPMGCCMYIPYTNMVVTFTLFAILMCRVLQHKLRSLEKLKNEQVRGEIIWCIKYQLKLSGFVDSMNALNTHLHLVEFLCFGAMLCVLLFSLIIAQTIAQTVIVIAYMVMIFANSVVLYYVANELYFQSFDIAIAAYESNWMDFDVDTQKTLKFLIMRSQKPLAILVGGTYPMNLKMLQSLLNAIYSFFTLLRRVYG

>DmOr33a

MDSRRKVRSENLYKTYWLYWRLLGVEGDYPFRRLVDFTITSFITILFPVHLILGMYKKPQIQVFRSLHFTSECLFCSYKFFCFRWKLKEIKTIEGLLQDLDSRVESEEERNYFNQNPSRVARMLSKSYLVAAISAIITATVAGLFSTGRNLMYLGWFPYDFQATAAIYWISFSYQAIGSSLLILENLANDSYPPITFCVVSGHVRLLIMRLSRIGHDVKLSSSENTRKLIEGIQDHRKLMKIIRLLRSTLHLSQLGQFLSSGINISITLINILFFAENNFAMLYYAVFFAAMLIELFPSCYYGILMTMEFDKLPYAIFSSNWLKMDKRYNRSLIILMQLTLVPVNIKAGGIVGIDMSAFFATVRMAYSFYTLALSFRV

>DmOr33b

MDLKPRVIRSEDIYRTYWLYWHLLGLESNFFLNRLLDLVITIFVTIWYPIHLILGLFMERSLGDVCKGLPITAACFFASFKFICFRFKLSEIKEIEILFKELDQRALSREECEFFNQNTRREANFIWKSFIVAYGLSNISAIASVLFGGGHKLLYPAWFPYDVQATELIFWLSVTYQIAGVSLAILQNLANDSYPPMTFCVVAGHVRLLAMRLSRIGQGPEETIYLTGKQLIESIEDHRKLMKIVELLRSTMNISQLGQFISSGVNISITLVNILFFADNNFAITYYGVYFLSMVLELFPCCYYGTLISVEMNQLTYAIYSSNWMSMNRSYSRILLIFMQLTLAEVQIKAGGMIGIGMNAFFATVRLAYSFFTLAMSLR

>DmOr33c

MVIIDSLSFYRPFWICMRLLVPTFFKDSSRPVQLYVVLLHILVTLWFPLHLLLHLLLLPSTAEFFKNLTMSLTCVACSLKHVAHLYHLPQIVEIESLIEQLDTFIASEQEHRYYRDHVHCHARRFTRCLYISFGMIYALFLFGVFVQVISGNWELLYPAYFPFDLESNRFLGAVALGYQVFSMLVEGFQGLGNDTYTPLTLCLLAGHVHLWSIRMGQLGYFDDETVVNHQRLLDYIEQHKLLVRFHNLVSRTISEVQLVQLGGCGATLCIIVSYMLFFVGDTISLVYYLVFFGVVCVQLFPSCYFASEVAEELERLPYAIFSSRWYDQSRDHRFDLLIFTQLTLGNRGWIIKAGGLIELNLNAFFATLKMAYSLFAVVVRAKGI

>DmOr35a

MVRYVPRFADGQKVKLAWPLAVFRLNHIFWPLDPSTGKWGRYLDKVLAVAMSLVFMQHNDAELRYLRFEASNRNLDAFLTGMPTYLILVEAQFRSLHILLHFEKLQKFLEIFYANIYIDPRKEPEMFRKVDGKMIINRLVSAMYGAVISLYLIAPVFSIINQSKDFLYSMIFPFDSDPLYIFVPLLLTNVWVGIVIDTMMFGETNLLCELIVHLNGSYMLLKRDLQLAIEKILVARDRPHMAKQLKVLITKTLRKNVALNQFGQQLEAQYTVRVFIMFAFAAGLLCALSFKAYTNPMANYIYAIWFGAKTVELLSLGQIGSDLAFTTDSLSTMYYLTHWEQILQYSTNPSENLRLLKLINLAIEMNSKPFYVTGLKYFRVSLQAGLKILQASFSYFTFLTSMQRRQMSN

>DmOr42a

MDLRRWFPTLYTQSKDSPVRSRDATLYLLRCVFLMGVRKPPAKFFVAYVLWSFALNFCSTFYQPIGFLTGYISHLSEFSPGEFLTSLQVAFNAWSCSTKVLIVWALVKRFDEANNLLDEMDRRITDPGERLQIHRAVSLSNRIFFFFMAVYMVYATNTFLSAIFIGRPPYQNYYPFLDWRSSTLHLALQAGLEYFAMAGACFQDVCVDCYPVNFVLVLRAHMSIFAERLRRLGTYPYESQEQKYERLVQCIQDHKVILRFVDCLRPVISGTIFVQFLVVGLVLGFTLINIVLFANLGSAIAALSFMAAVLLETTPFCILCNYLTEDCYKLADALFQSNWIDEEKRYQKTLMYFLQKLQQPITFMAMNVFPISVGTNISVTKFSFSVFTLVKQMNISEKLAKSEMEE

>DmOr42b

MVFELIRPAPLTEQKRSRDGCIYLYRAMKFIGWLPPKQGVLRYVYLTWTLMTFVWCTTYLPLGFLGSYMTQIKSFSPGEFLTSLQVCINAYGSSVKVAITYSMLWRLIKAKNILDQLDLRCTAMEEREKIHLVVARSNHAFLIFTFVYCGYAGSTYLSSVLSGRPPWQLYNPFIDWHDGTLKLWVASTLEYMVMSGAVLQDQLSDSYPLIYTLILRAHLDMLRERIRRLRSDENLSEAESYEELVKCVMDHKLILRYCAIIKPVIQGTIFTQFLLIGLVLGFTLINVFFFSDIWTGIASFMFVITILLQTFPFCYTCNLIMEDCESLTHAIFQSNWVDASRRYKTTLLYFLQNVQQPIVFIAGGIFQISMSSNISVAKFAFSVITITKQMNIADKFKTD

>DmOr43a

MTIEDIGLVGINVRMWRHLAVLYPTPGSSWRKFAFVLPVTAMNLMQFVYLLRMWGDLPAFILNMFFFSAIFNALMRTWLVIIKRRQFEEFLGQLATLFHSILDSTDEWGRGILRRAEREARNLAILNLSASFLDIVGALVSPLFREERAHPFGLALPGVSMTSSPVYEVIYLAQLPTPLLLSMMYMPFVSLFAGLAIFGKAMLQILVHRLGQIGGEEQSEEERFQRLASCIAYHTQVMRYVWQLNKLVANIVAVEAIIFGSIICSLLFCLNIITSPTQVISIVMYILTMLYVLFTYYNRANEICLENNRVAEAVYNVPWYEAGTRFRKTLLIFLMQTQHPMEIRVGNVYPMTLAMFQSLLNASYSYFTMLRGVTGK

>DmOr43b

MFGHFKLVYPAPISEPIQSRDSNAYMMETLRNSGLNLKNDFGIGRKIWRVFSFTYNMVILPVSFPINYVIHLAEFPPELLLQSLQLCLNTWCFALKFFTLIVYTHRLELANKHFDELDKYCVKPAEKRKVRDMVATITRLYLTFVVVYVLYATSTLLDGLLHHRVPYNTYYPFINWRVDRTQMYIQSFLEYFTVGYAIYVATATDSYPVIYVAALRTHILLLKDRIIYLGDPSNEGSSDPSYMFKSLVDCIKAHRTMLNFCDAIQPIISGTIFAQFIICGSILGIIMINMVLFADQSTRFGIVIYVMAVLLQTFPLCFYCNAIVDDCKELAHALFHSAWWVQDKRYQRTVIQFLQKLQQPMTFTAMNIFNINLATNINVAKFAFTVYAIASGMNLDQKLSIKE

>DmOr45a

MDASYFAVQRRALEIVGFDPSTPQLSLKHPIWAGILILSLISHNWPMVVYALQDLSDLTRLTDNFAVFMQGSQSTFKFLVMMAKRRRIGSLIHRLHKLNQAASATPNHLEKIERENQLDRYVARSFRNAAYGVICASAIAPMLLGLWGYVETGVFTPTTPMEFNFWLDERKPHFYWPIYVWGVLGVAAAAWLAIATDTLFSWLTHNVVIQFQLLELVLEEKDLNGGDSRLTGFVSRHRIALDLAKELSSIFGEIVFVKYMLSYLQLCMLAFRFSRSGWSAQVPFRATFLVAIIIQLSSYCYGGEYIKQQSLAIAQAVYGQINWPEMTPKKRRLWQMVIMRAQRPAKIFGFMFVVDLPLLLWVIRTAGSFLAMLRTFER

>DmOr45b

MYPRFLSRNYPLAKHLFFVTRYSFGLLGLRFGKEQSWLHLLWLVFNFVNLAHCCQAEFVFGWSHLRTSPVDAMDAFCPLACSFTTLFKLGWMWWRRQEVADLMDRIRLLIGEQEKREDSRRKVAQRSYYLMVTRCGMLVFTLGSITTGAFVLRSLWEMWVRRHQEFKFDMPFRMLFHDFAHRMPWFPVFYLYSTWSGQVTVYAFAGTDGFFFGFTLYMAFLLQALRYDIQDALKPIRDPSLRESKICCQRLADIVDRHNEIEKIVKEFSGIMAAPTFVHFVSASLVIATSVIDILLYSGYNIIRYVVYTFTVSSAIFLYCYGGTEMSTESLSLGEAAYSSAWYTWDRETRRRVFLIILRAQRPITVRVPFFAPSLPVFTSVIKFTGSIVALAKTIL

>DmOr46aA

MSKGVEIFYKGQKAFLNILSLWPQIERRWRIIHQVNYVHVIVFWVLLFDLLLVLHVMANLSYMSEVVKAIFILATSAGHTTKLLSIKANNVQMEELFRRLDNEEFRPRGANEELIFAAACERSRKLRDFYGALSFAALSMILIPQFALDWSHLPLKTYNPLGENTGSPAYWLLYCYQCLALSVSCITNIGFDSLCSSLFIFLKCQLDILAVRLDKIGRLITTSGGTVEQQLKENIRYHMTIVELSKTVERLLCKPISVQIFCSVLVLTANFYAIAVLSDERLELFKYVTYQACMLIQIFILCYYAGEVTQRSLDLPHELYKTSWVDWDYRSRRIALLFMQRLHSTLRIRTLNPSLGFDLMLFSSIVNCSYSYFALLKRVNS

>DmOr46aB

MVTEDFYKYQVWYFQILGVWQLPTWAADHQRRFQSMRFGFILVILFIMLLLFSFEMLNNISQVREILKVFFMFATEISCMAKLLHLKLKSRKLAGLVDAMLSPEFGVKSEQEMQMLELDRVAVVRMRNSYGIMSLGAASLILIVPCFDNFGELPLAMLEVCSIEGWICYWSQYLFHSICLLPTCVLNITYDSVAYSLLCFLKVQLQMLVLRLEKLGPVIEPQDNEKIAMELRECAAYYNRIVRFKDLVELFIKGPGSVQLMCSVLVLVSNLYDMSTMSIANGDAIFMLKTCIYQLVMLWQIFIICYASNEVTVQSSRLCHSIYSSQWTGWNRANRRIVLLMMQRFNSPMLLSTFNPTFAFSLEAFGSIVNCSYSYFALLKRVNS

>DmOr47a

MDSFLQVQKSTIALLGFDLFSENREMWKRPYRAMNVFSIAAIFPFILAAVLHNWKNVLLLADAMVALLITILGLFKFSMILYLRRDFKRLIDKFRLLMSNEAEQGEEYAEILNAANKQDQRMCTLFRTCFLLAWALNSVLPLVRMGLSYWLAGHAEPELPFPCLFPWNIHIIRNYVLSFIWSAFASTGVVLPAVSLDTIFCSFTSNLCAFFKIAQYKVVRFKGGSLKESQATLNKVFALYQTSLDMCNDLNQCYQPIICAQFFISSLQLCMLGYLFSITFAQTEGVYYASFIATIIIQAYIYCYCGENLKTESASFEWAIYDSPWHESLGAGGASTSICRSLLISMMRAHRGFRITGYFFEANMEAFSSIVRTAMSYITMLRSFS

>DmOr47b

MNDSGYQSNLSLLRVFLDEFRSVLRQESPGLIPRLAFYYVRAFLSLLCQYPNKKLASLPLYRWINLFIMCNVMTIFWTMFVALPESKNVIEMGDDLVWISGMALVFTKIFYMHLRCDEIDELISDFEYYNRELRPHNIDEEVLGWQRLCYVIESGLYINCFCLVNFFSAAIFLQPLLGEGKLPFHSVYPFQWHRLDLHPYTFWFLYIWQSLTSQHNLMSILMVDMVGISTFLQTALNLKLLCIEIRKLGDMEVSDKRFHEEFCRVVRFHQHIIKLVGKANRAFNGAFNAQLMASFSLISISTFETMAAAAVDPKMAAKFVLLMLVAFIQLSLWCVSGTLVYTQSVEVAQAAFDINDWHTKSPGIQRDISFVILRAQKPLMYVAEPFLPFTLGTYMLVLKNCYRLLALMQESM

>DmOr49a

MEKLRSYEDFIFMANMMFKTLGYDLFHTPKPWWRYLLVRGYFVLCTISNFYEASMVTTRIIEWESLAGSPSKIMRQGLHFFYMLSSQLKFITFMINRKRLLQLSHRLKELYPHKEQNQRKYEVNKYYLSCSTRNVLYVYYFVMVVMALEPLVQSCIMYLIGFGKADFTYKRIFPTRLTFDSEKPLGYVLAYVIDFTYSQFIVNVSLGTDLWMMCVSSQISMHLGYLANMLASIRPSPETEQQDCDFLASIIKRHQLMIRLQKDVNYVFGLLLASNLFTTSCLLCCMAYYTVVEGFNWEGISYMMLFASVAAQFYVVSSHGQMLIDLSTNLAKAAFESKWYEGSLRYKKEILILMAQAQRPLEISARGVIIISLDTFKILMTITYRFFAVIRQTVEK

>DmOr49b

MFEDIQLIYMNIKILRFWALLYDKNLRRYVCIGLASFHIFTQIVYMMSTNEGLTGIIRNSYMLVLWINTVLRAYLLLADHDRYLALIQKLTEAYYDLLNLNDSYISEILDQVNKVGKLMARGNLFFGMLTSMGFGLYPLSSSERVLPFGSKIPGLNEYESPYYEMWYIFQMLITPMGCCMYIPYTSLIVGLIMFGIVRCKALQHRLRQVALKHPYGDRDPRELREEIIACIRYQQSIIEYMDHINELTTMMFLFELMAFSALLCALLFMLIIVSGTSQLIIVCMYINMILAQILALYWYANELREQNLAVATAAYETEWFTFDVPLRKNILFMMMRAQRPAAILLGNIRPITLELFQNLLNTTYTFFTVLKRVYG

>DmOr56a

MFKVKDLLLSPTTFEDPIFGTHLRYFQWYGYVASKDQNRPLLSLIRCTILTASIWLSCALMLARVFRGYENLNDGATSYATAVQYFAVSIAMFNAYVQRDKVISLLRVAHSDIQNLMHEADNREMELLVATQAYTRTITLLIWIPSVIAGLMAYSDCIYRSLFLPKSVFNVPAVRRGEEHPILLFQLFPFGELCDNFVVGYLGPWYALGLGITAIPLWHTFITCLMKYVNLKLQILNKRVEEMDITRLNSKLVIGRLTASELTFWQMQLFKEFVKEQLRIRKFVQELQYLICVPVMADFIIFSVLICFLFFALTVGVPSKMDYFFMFIYLFVMAGILWIYHWHATLIVECHDELSLAYFSCGWYNFEMPLQKMLVFMMMHAQRPMKMRALLVDLNLRTFIDIGRGAYSYFNLLRSSHLY

>DmOr59a

MAEVRVDSLEFFKSHWTAWRYLGVAHFRVENWKNLYVFYSIVSNLLVTLCYPVHLGISLFRNRTITEDILNLTTFATCTACSVKCLLYAYNIKDVLEMERLLRLLDERVVGPEQRSIYGQVRVQLRNVLYVFIGIYMPCALFAELSFLFKEERGLMYPAWFPFDWLHSTRNYYIANAYQIVGISFQLLQNYVSDCFPAVVLCLISSHIKMLYNRFEEVGLDPARDAEKDLEACITDHKHILELFRRIEAFISLPMLIQFTVTALNVCIGLAALVFFVSEPMARMYFIFYSLAMPLQIFPSCFFGTDNEYWFGRLHYAAFSCNWHTQNRSFKRKMMLFVEQSLKKSTAVAGGMMRIHLDTFFSTLKGAYSLFTIIIRMRK

>DmOr59b

MAVFKLIKPAPLTEKVQSRQGNIYLYRAMWLIGWIPPKEGVLRYVYLFWTCVPFAFGVFYLPVGFIISYVQEFKNFTPGEFLTSLQVCINVYGASVKSTITYLFLWRLRKTEILLDSLDKRLANDSDRERIHNMVARCNYAFLIYSFIYCGYAGSTFLSYALSGRPPWSVYNPFIDWRDGMGSLWIQAIFEYITMSFAVLQDQLSDTYPLMFTIMFRAHMEVLKDHVRSLRMDPERSEADNYQDLVNCVLDHKTILKCCDMIRPMISRTIFVQFALIGSVLGLTLVNVFFFSNFWKGVASLLFVITILLQTFPFCYTCNMLIDDAQDLSNEIFQSNWVDAEPRYKATLVLFMHHVQQPIIFIAGGIFPISMNSNITVAKFAFSIITIVRQMNLAEQFQ

>DmOr59c

MTKFFFKRLQTAPLDQEVSSLDASDYYYRIAFFLGWTPPKGALLRWIYSLWTLTTMWLGIVYLPLGLSLTYVKHFDRFTPTEFLTSLQVDINCIGNVIKSCVTYSQMWRFRRMNELISSLDKRCVTTTQRRIFHKMVARVNLIVILFLSTYLGFCFLTLFTSVFAGKAPWQLYNPLVDWRKGHWQLWIASILEYCVVSIGTMQELMSDTYAIVFISLFRCHLAILRDRIANLRQDPKLSEMEHYEQMVACIQDHRTIIQCSQIIRPILSITIFAQFMLVGIDLGLAAISILFFPNTIWTIMANVSFIVAICTESFPCCMLCEHLIEDSVHVSNALFHSNWITADRSYKSAVLYFLHRAQQPIQFTAGSIFPISVQSNIAVAKFAFTIITIVNQMNLGEKFFSDRSNGDINP

>DmOr63a

MYSPEEAAELKRRNYRSIREMIRLSYTVGFNLLDPSRCGQVLRIWTIVLSVSSLASLYGHWQMLARYIHDIPRIGETAGTALQFLTSIAKMWYFLFAHRQIYELLRKARCHELLQKCELFERMSDLPVIKEIRQQVESTMNRYWASTRRQILIYLYSCICITTNYFINSFVINLYRYFTKPKGSYDIMLPLPSLYPAWEHKGLEFPYYHIQMYLETCSLYICGMCAVSFDGVFIVLCLHSVGLMRSLNQMVEQATSELVPPDRRVEYLRCCIYQYQRVANFATEVNNCFRHITFTQFLLSLFNWGLALFQMSVGLGNNSSITMIRMTMYLVAAGYQIVVYCYNGQRFATASEEIANAFYQVRWYGESREFRHLIRMMLMRTNRGFRLDVSWFMQMSLPTLMAMVRTSGQYFLLLQNVNQK

>DmOr65a

MTELRSERKNGNWDRLFGPFFESWAVFKAPQAKSRHIIAYWTRDQLKALGFYMNSEQRRLPRIVAWQYFVSIQLATALASLFYGISESIGDIVNLGRDLVFIITIIFICFRLVFFAQYAGELDVIIDALEDIYHWSIKGPATKEVQETKRLHFLLFMALIITWFSFLILFMLIKISTPFWIESQTLPFHVSWPFQLHDPSKHPIAYIIIFVSQSTTMLYFLIWLGVVENMGVSLFFELTSALRVLCIELRNLQELCLGDEDMLYRELCRMTKFHQQIILLTDRCNHIFNGAFIMQMLINFLLVSLSLFEVLAAKKNPQVAVEYMIIMLMTLGHLSFWSKFGDMFSKESEQVALAVYEAYDPNVGSKSIHRQFCFFIQRAQKPLIMKASPFPPFNLENYMFILKQCYSILTILANTLE

>DmOr65b

MEASHSSIYYWREQMKAMALFTTTEERLLPYRSKWHTLVYIQMVIFFASMSFGLTESMGDHVQMGRDLAFILGAFFIIFKTYYFCWYGDELDQVISDLDALHPWAQKGPNPVEYQTGKRWYFVMAFFLATSWSFFLCILLLLLITSPMWVHQQNLPFHAAFPFQWHEKSLHPISHAIIYLFQSYFAVYCLTWLLCIEGLSICIYAEITFGIEVLCLELRQIHRHNYGLQELRMETNRLVKLHQKIVEILDRTNDVFHGTLIMQMGVNFSLVSLSVLEAVEARKDPKVVAQFAVLMLLALGHLSMWSYCGDQLSQKSLQISEAAYEAYDPTKGSKDVYRDLCVIIRRGQDPLIMRASPFPSFNLINYSAILNQCYGILTFLLKTLD

>DmOr65c

MESSYSAVYYWREQMKAMFLYTTSKERQMPYRSSWHTLVIIQATVCFLTMCYGVTESLGDKVQMGRDIAFIIGFFYIAFKIYYFQWYGDELDEVVEALETFHPWAQKGPGAVDYRTAKRWYFTLAFFLASSWLVFLCIFILLLITSPLWVHQQILPLHAAFPFQWHEKSIHPISHAFIYLFQTWNVMYFLTWLVCIEGLSVSIYVEITFAIEVLCLELRHLHQRCHGYEQLRLETNRLVQFHQKIVHILDHTNKVFHGTLIMQMGVNFFLVSLSVLEAMEARKDPKVVAQFAVLMLLALGHLSMWSYFGDLLSQKSLTISEAAYEAYDPIKGSKDVYRDLCLIIRRGQEPLIMRASPFPSFNFINYSAILNQCYGILTFLLKTLD

>DmOr67a

MDNVAEMPEEKYVEVDDFLRLAVKFYNTLGIDPYETGRKRTIWFQIYFALNMFNMVFSFYAEVATLVDRLRDNENFLESCILLSYVSFVVMGLSKIGAVMKKKPKMTALVRQLETCFPSPSAKVQEEYAVKSWLKRCHIYTKGFGGLFMIMYFAHALIPLFIYFIQRVLLHYPDAKQIMPFYQLEPWEFRDSWLFYPSYFHQSSAGYTATCGSIAGDLMIFAVVLQVIMHYERLAKVLREFKIQAHNAPNGAKEDIRKLQSLVANHIDILRLTDLMNEVFGIPLLLNFIASALLVCLVGVQLTIALSPEYFCKQMLFLISVLLEVYLLCSFSQRLIDASENVGHAAYDMDWLGSDKRFKKILIFISMRSQKPVCLKATVVLDLSMPTMSIFLGMSYKFFCAVRTMYQ

>DmOr67b

MQDQLDHELERIDKLPKLGLLWVEYSAYALGVNIAPRKRSSKYCRLTRILVLIVNLSIIYSLVAFIMENYMISFETYVEAVLLTFQLSVGVVKMFHFQNKVESCSQLVFSTETGEVLKSLGLFQLDLPRKKELLSSVSLILLNNWMIIDRQVMFFFKIVCMPVLYYCVRPYFQYIFDCYIKDKDTCEMTLTYPAIVPYLQLGNYEFPSYVIRFFLLQSGPLWCFFAVFGFNSLFVVLTRYESGLIKVLRFLVQNSTSDILVPKDQRVKYLQCCVRLFARISSHHNQIENLFKYIILVQCSVSSILICMLLYKISTVLEVGWVWMGMIMVYFVTIALEITLYNVSAQKVESQSELLFHDWYNCSWYNESREFKFMIKMMLLFSRRTFVLSVGGFTSLSHKFLVQVFRLSANFFLLLRNMNNK

>DmOr67c

METAKDNTARTFMELMRVPVQFYRTIGEDIYAHRSTNPLKSLLFKIYLYAGFINFNLLVIGELVFFYNSIQDFETIRLAIAVAPCIGFSLVADFKQAAMIRGKKTLIMLLDDLENMHPKTLAKQMEYKLPDFEKTMKRVINIFTFLCLAYTTTFSFYPAIKASVKFNFLGYDTFDRNFGFLIWFPFDATRNNLIYWIMYWDIAHGAYLAGIAFLCADLLLVVVITQICMHFNYISMRLEDHPCNSNEDKENIEFLIGIIRYHDKCLKLCEHVNDLYSFSLLLNFLMASMQICFIAFQVTESTVEVIIIYCIFLMTSMVQVFMVCYYGDTLIAASLKVGDAAYNQKWFQCSKSYCTMLKLLIMRSQKPASIRPPTFPPISLVTYMKVISMSYQFFALLRTTYSNN

>DmOr67d

MLKMAKVEPVERYCKVIRMIRFCVGFCGNDVADPNFRMWWLTYAVMAAIAFFFACTGYTIYVGVVINGDLTIILQALAMVGSAVQGLTKLLVTANNASHMREVQNTYEDIYREYGSKGDEYAKCLEKRIRITWTLLIGFMLVYIILLGLVITFPIFYLLILHQKVLVMQFLIPFLDHTTDGGHLILTAAHVILITFGGFGNYGGDMYLFLFVTHVPLIKDIFCVKLTEFNELVMKRNDFPKVRAMLCDLLVWHQLYTRMLQTTKKIYSIVLFVQLSTTCVGLLCTISCIFMKAWPAAPLYLLYAAITLYTFCGLGTLVENSNEDFLSVIYTNCLWYELPVKEEKLIIMMLAKAQNEVVLTAADMAPLSMNTALQLTKGIYSFSMMLMNYLG

>DmOr69aA

MQLHDHMKYIDLGCKMACIPRYQWKGRPTERQFYASEQRIVFLLGTICQIFQITGVLIYWYCNGRLATETGTFVAQLSEMCSSFCLTFVGFCNVYAISTNRNQIETLLEELHQIYPRYRKNHYRCQHYFDMAMTIMRIEFLFYMILYVYYNSAPLWVLLWEHLHEEYDLSFKTQTNTWFPWKVHGSALGFGMAVLSITVGSFVGVGFSIVTQNLICLLTFQLKLHYDGISSQLVSLDCRRPGAHKELSILIAHHSRILQLGDQVNDIMNFVFGSSLVGATIAICMSSVSIMLLDLASAFKYASGLVAFVLYNFVICYMGTEVTLASGKVLPAAFYNNWYEGDLVYRRMLLILMMRATKPYMWKTYKLAPVSITTYMATLKFSYQMFTCVRSLK

>DmOr69aB

MQLEDFMRYPDLVCQAAQLPRYTWNGRRSLEVKRNLAKRIIFWLGAVNLVYHNIGCVMYGYFGDGRTKDPIAYLAELASVASMLGFTIVGTLNLWKMLSLKTHFENLLNEFEELFQLIKHRAYRIHHYQEKYTRHIRNTFIFHTSAVVYYNSLPILLMIREHFSNSQQLGYRIQSNTWYPWQVQGSIPGFFAAVACQIFSCQTNMCVNMFIQFLINFFGIQLEIHFDGLARQLETIDARNPHAKDQLKYLIVYHTKLLNLADRVNRSFNFTFLISLSVSMISNCFLAFSMTMFDFGTSLKHLLGLLLFITYNFSMCRSGTHLILTSGKVLPAAFYNNWYEGDLVYRRMLLILMMRATKPYMWKTYKLAPVSITTYMATLKFSYQMFTCVRSLK

>DmOr71a

MDYDRIRPVRFLTGVLKWWRLWPRKESVSTPDWTNWQAYALHVPFTFLFVLLLWLEAIKSRDIQHTADVLLICLTTTALGGKVINIWKYAHVAQGILSEWSTWDLFELRSKQEVDMWRFEHRRFNRVFMFYCLCSAGVIPFIVIQPLFDIPNRLPFWMWTPFDWQQPVLLWYAFIYQATTIPIACACNVTMDAVNWYLMLHLSLCLRMLGQRLSKLQHDDKDLREKFLELIHLHQRLKQQALSIEIFISKSTFTQILVSSLIICFTIYSMQMSPVLQDLPGFAAMMQYLVAMIMQVMLPTIYGNAVIDSANMLTDSMYNSDWPDMNCRMRRLVLMFMVYLNRPVTLKAGGFFHIGLPLFTKTMNQAYSLLALLLNMNQ

>DmOr74a

MSFHRYRPRLPGGELAPMPWPVSLYRVLNHVAWPLEAESGRWTVFLDRLMIFLGFLVFCEHNEVDFHYLIANRQDMDNMLTGLPTYLILVEMQIRCFQLAWHKDRFRALLQRFYAEIYVSEEMEPHLFASIQRQMLATRVNSTVYLLALLNFFLVPVTNVIYHRREMLYKQVYPFDNTQLHFFIPLLVLNFWVGFIITSMLFGELNVMGELMMHLNARYIQLGQDLRRSAQMLLKKSSSLNVAIAYRLNLTHILRRNAALRDFGQRVEKEFTLRIFVMFAFSAGLLCALFFKAFTNPWGNVAYIVWFLAKFMELLALGMLGSILLKTTDELGMMYYTADWEQVIHQSDNVGENVKLMKLVTLAIQLNSRPFFITGLNYFRVSLTAVLKIIQGAFSYFTFLNSMR

>DmOr82a

MGRLFQLQEYCLRAMGHKDDMDSTDSTALSLKHISSLIFVISAQYPLISYVAYNRNDMEKVTACLSVVFTNMLTVIKISTFLANRKDFWEMIHRFRKMHEQSSHIPRYREGLDYVAEANKLASFLGRAYCVSCGLTGLYFMLGPIVKIGVCRWHGTTCDKELPMPMKFPFNDLESPGYEVCFLYTVLVTVVVVAYASAVDGLFISFAINLRAHFQTLQRQIENWEFPSSEPDTQIRLKSIVEYHVLLLSLSRKLRSIYTPTVMGQFVITSLQVGVIIYQLVTNMDSVMDLLLYASFFGSIMLQLFIYCYGGEIIKAESLQVDTAVRLSNWHLASPKTRTSLSLIILQSQKEVLIRAGFFVASLANFVGICRTALSLITLIKSIE

>DmOr83a

MKSTFKEERIKDDSKRRDLFVFVRQTMCIAAMYPFGYYVNGSGVLAVLVRFCDLTYELFNYFVSVHIAGLYICTIYINYGQGDLDFFVNCLIQTIIYLWTIAMKLYFRRFRPGLLNTILSNINDEYETRSAVGFSFVTMAGSYRMSKLWIKTYVYCCYIGTIFWLALPIAYRDRSLPLACWYPFDYTQPGVYEVVFLLQAMGQIQVAASFASSSGLHMVLCVLISGQYDVLFCSLKNVLASSYVLMGANMTELNQLQAEQSAADVEPGQYAYSVEEETPLQELLKVGSSMDFSSAFRLSFVRCIQHHRYIVAALKKIESFYSPIWFVKIGEVTFLMCLVAFVSTKSTAANSFMRMVSLGQYLLLVLYELFIICYFADIVFQNSQRCGEALWRSPWQRHLKDVRSDYMFFMLNSRRQFQLTAGKISNLNVDRFRGTITTAFSFLTLLQKMDARE

>DmOr83b

MTTSMQPSKYTGLVADLMPNIRAMKYSGLFMHNFTGGSAFMKKVYSSVHLVFLLMQFTFILVNMALNAEEVNELSGNTITTLFFTHCITKFIYLAVNQKNFYRTLNIWNQVNTHPLFAESDARYHSIALAKMRKLFFLVMLTTVASATAWTTITFFGDSVKMVVDHETNSSIPVEIPRLPIKSFYPWNASHGMFYMISFAFQIYYVLFSMIHSNLCDVMFCSWLIFACEQLQHLKGIMKPLMELSASLDTYRPNSAALFRSLSANSKSELIHNEEKDPGTDMDMSGIYSSKADWGAQFRAPSTLQSFGGNGGGGNGLVNGANPNGLTKKQEMMVRSAIKYWVERHKHVVRLVAAIGDTYGAALLLHMLTSTIKLTLLAYQATKINGVNVYAFTVVGYLGYALAQVFHFCIFGNRLIEESSSVMEAAYSCHWYDGSEEAKTFVQIVCQQCQKAMSISGAKFFTVSLDLFASVLGA

VVTYFMVLVQLK

>DmOr83c

MSTSESPSSRFRELSKYINSLTNLLGVDFLSPKLKFNYRTWTTIFAIANYTGFTVFTILNNGGDWRVGLKASLMTGGLFHGLGKFLTCLLKHQDMRRLVLYSQSIYDEYETRGDSYHRTLNSNIDRLLGIMKIIRNGYVFAFCLMELLPLAMLMYDGTRVTAMQYLIPGLPLENNYCYVVTYMIQTVTMLVQGVGFYSGDLFVFLGLTQILTFADMLQVKVKELNDALEQKAEYRALVRVGASIDGAENRQRLLLDVIRWHQLFTDYCRAINALYYELIATQVLSMALAMMLSFCINLSSFHMPSAIFFVVSAYSMSIYCILGTILEFAYDQVYESICNVTWYELSGEQRKLFGFLLRESQYPHNIQILGVMSLSVRTALQIVKLIYSVSMMMMNRA

>DmOr85a

MIFKYIQEPVLGSLFRSRDSLIYLNRSIDQMGWRLPPRTKPYWWLYYIWTLVVIVLVFIFIPYGLIMTGIKEFKNFTTTDLFTYVQVPVNTNASIMKGIIVLFMRRRFSRAQKMMDAMDIRCTKMEEKVQVHRAAALCNRVVVIYHCIYFGYLSMALTGALVIGKTPFCLYNPLVNPDDHFYLATAIESVTMAGIILANLILDVYPIIYVVVLRIHMELLSERIKTLRTDVEKGDDQHYAELVECVKDHKLIVEYGNTLRPMISATMFIQLLSVGLLLGLAAVSMQFYNTVMERVVSGVYTIAILSQTFPFCYVCEQLSSDCESLTNTLFHSKWIGAERRYRTTMLYFIHNVQQSILFTAGGIFPICLNTNIKMAKFAFSVVTIVNEMDLAEKLRRE

>DmOr85b

MEKLMKYASFFYTAVGIRPYTNGEESKMNKLIFHIVFWSNVINLSFVGLFESIYVYSAFMDNKFLEAVTALSYIGFVTVGMSKMFFIRWKKTAITELINELKEIYPNGLIREERYNLPMYLGTCSRISLIYSLLYSVLIWTFNLFCVMEYWVYDKWLNIRVVGKQLPYLMYIPWKWQDNWSYYPLLFSQNFAGYTSAAGQISTDVLLCAVATQLVMHFDFLSNSMERHELSGDWKKDSRFLVDIVRYHERILRLSDAVNDIFGIPLLLNFMVSSFVICFVGFQMTVGVPPDIVVKLFLFLVSSMSQVYLICHYGQLVADASYGFSVATYNQKWYKADVRYKRALVIIIARSQKVTFLKATIFLDITRSTMTDLLQISYKFFALLRTMYTQ

>DmOr85c

MKFMKYAVFFYTSVGIEPYTIDSRSKKASLWSHLLFWANVINLSVIVFGEILYLGVAYSDGKFIDAVTVLSYIGFVIVGMSKMFFIWWKKTDLSDLVKELEHIYPNGKAEEEMYRLDRYLRSCSRISITYALLYSVLIWTFNLFSIMQFLVYEKLLKIRVVGQTLPYLMYFPWNWHENWTYYVLLFCQNFAGHTSASGQISTDLLLCAVATQVVMHFDYLARVVEKQVLDRDWSENSRFLAKTVQYHQRILRLMDVLNDIFGIPLLLNFMVSTFVICFVGFQMTVGVPPDIMIKLFLFLFSSLSQVYLICHYGQLIADASSSLSISAYKQNWQNADIRYRRALVFFIARPQRTTYLKATIFMNITRATMTDLLQVSYKFFALLRTMYIK

>DmOr85d

MLTKKDTQSAKEQEKLKAIPLHSFLKYANVFYLSIGMMAYDHKYSQKWKEVLLHWTFIAQMVNLNTVLISELIYVFLAIGKGSNFLEATMNLSFIGFVIVGDFKIWNISRQRKRLTQVVSRLEELHPQGLAQQEPYNIGHHLSGYSRYSKFYFGMHMVLIWTYNLYWAVYYLVCDFWLGMRQFERMLPYYCWVPWDWSTGYSYYFMYISQNIGGQACLSGQLAADMLMCALVTLVVMHFIRLSAHIESHVAGIGSFQHDLEFLQATVAYHQSLIHLCQDINEIFGVSLLSNFVSSSFIICFVGFQMTIGSKIDNLVMLVLFLFCAMVQVFMIATHAQRLVDASEQIGQAVYNHDWFRADLRYRKMLILIIKRAQQPSRLKATMFLNISLVTVSDLLQLSYKFFALLRTMYVN

>DmOr85e

MASLQFHGNVDADIRYDISLDPARESNLFRLLMGLQLANGTKPSPRLPKWWPKRLEMIGKVLPKAYCSMVIFTSLHLGVLFTKTTLDVLPTGELQAITDALTMTIIYFFTGYGTIYWCLRSRRLLAYMEHMNREYRHHSLAGVTFVSSHAAFRMSRNFTVVWIMSCLLGVISWGVSPLMLGIRMLPLQCWYPFDALGPGTYTAVYATQLFGQIMVGMTFGFGGSLFVTLSLLLLGQFDVLYCSLKNLDAHTKLLGGESVNGLSSLQEELLLGDSKRELNQYVLLQEHPTDLLRLSAGRKCPDQGNAFHNALVECIRLHRFILHCSQELENLFSPYCLVKSLQITFQLCLLVFVGVSGTREVLRIVNQLQYLGLTIFELLMFTYCGELLSRHSIRSGDAFWRGAWWKHAHFIRQDILIFLVNSRRAVHVTAGKFYVMDVNRLRSVITQAFSFLTLLQKLAAKKTESEL

>DmOr85f

MEPVQYSYEDFARLPTTVFWIMGYDMLGVPKTRSRRILYWIYRFLCLASHGVCVGVMVFRMVEAKTIDNVSLIMRYATLVTYIINSDTKFATVLQRSAIQSLNSKLAELYPKTTLDRIYHRVNDHYWTKSFVYLVIIYIGSSIMVVIGPIITSIIAYFTHNVFTYMHCYPYFLYDPEKDPVWIYISIYALEWLHSTQMVISNIGADIWLLYFQVQINLHFRGIIRSLADHKPSVKHDQEDRKFIAKIVDKQVHLVSLQNDLNGIFGKSLLLSLLTTAAVICTVAVYTLIQGPTLEGFTYVIFIGTSVMQVYLVCYYGQQVLDLSGEVAHAVYNHDFHDASIAYKRYLLIIIIRAQQPVELNAMGYLSISLDTFKQLMSVSYRVITMLMQMIQ

>DmOr88a

MKPTEIKKPYRMEEFLRPQMFQEVAQMVHFQWRRNPVDNSMVNASMVPFCLSAFLNVLFFGCNGWDIIGHFWLGHPANQNPPVLSITIYFSIRGLMLYLKRKEIVEFVNDLDRECPRDLVSQLDMQMDETYRNFWQRYRFIRIYSHLGGPMFCVVPLALFLLTHEGKDTPVAQHEQLLGGWLPCGVRKDPNFYLLVWSFDLMCTTCGVSFFVTFDNLFNVMQGHLVMHLGHLARQFSAIDPRQSLTDEKRFFVDLRLLVQRQQLLNGLCRKYNDIFKVAFLVSNFVGAGSLCFYLFMLSETSDVLIIAQYILPTLVLVGFTFEICLRGTQLEKASEGLESSLRSQEWYLGSRRYRKFYLLWTQYCQRTQQLGAFGLIQVNMVHFTEIMQLAYRLFTFLKSH

>DmOr92a

MLFRKRKPKSDDEVITFDELTRFPMTFYKTIGEDLYSDRDPNVIRRYLLRFYLVLGFLNFNAYVVGEIAYFIVHIMSTTTLLEATAVAPCIGFSFMADFKQFGLTVNRKRLVRLLDDLKEIFPLDLEAQRKYNVSFYRKHMNRVMTLFTILCMTYTSSFSFYPAIKSTIKYYLMGSEIFERNYGFHILFPYDAETDLTVYWFSYWGLAHCAYVAGVSYVCVDLLLIATITQLTMHFNFIANDLEAYEGGDHTDEENIKYLHNLVVYHARALDLSEEVNNIFSFLILWNFIAASLVICFAGFQITASNVEDIGVYFIFFSASLVQVFVVCYYGDEMISSSSRIGHSAFNQNWLPCSTKYKRILQFIIARSQKPASIRPPTFPPISFNTFMKVISMSYQFFALLRTTYYG

>DmOr94a

MDKHKDRIESMRLILQVMQLFGLWPWSLKSEEEWTFTGFVKRNYRFLLHLPITFTFIGLMWLEAFISSNLEQAGQVLYMSITEMALVVKILSIWHYRTEAWRLMYELQHAPDYQLHNQEEVDFWRREQRFFKWFFYIYILISLGVVYSGCTGVLFLEGYELPFAYYVPFEWQNERRYWFAYGYDMAGMTLTCISNITLDTLGCYFLFHISLLYRLLGLRLRETKNMKNDTIFGQQLRAIFIMHQRIRSLTLTCQRIVSPYILSQIILSALIICFSGYRLQHVGIRDNPGQFISMLQFVSVMILQIYLPCYYGNEITVYANQLTNEVYHTNWLECRPPIRKLLNAYMEHLKKPVTIRAGNFFAVGLPIFVKTINNAYSFLALLLNVSN

>DmOr94b

MESTNRLSAIQTLLVIQRWIGLLKWENEGEDGVLTWLKRIYPFVLHLPLTFTYIALMWYEAITSSDFEEAGQVLYMSITELALVTKLLNIWYRRHEAASLIHELQHDPAFNLRNSEEIKFWQQNQRNFKRIFYWYIWGSLFVAVMGYISVFFQEDYELPFGYYVPFEWRTRERYFYAWGYNVVAMTLCCLSNILLDTLGCYFMFHIASLFRLLGMRLEALKNAAEEKARPELRRIFQLHTKVRRLTRECEVLVSPYVLSQVVFSAFIICFSAYRLVHMGFKQRPGLFVTTVQFVAVMIVQIFLPCYYGNELTFHANALTNSVFGTNWLEYSVGTRKLLNCYMEFLKRPVKVRAGVFFEIGLPIFVKTINNAYSFFALLLKISK

>DmOr98a

MLFNYLRKPNPTNLLTSPDSFRYFEYGMFCMGWHTPATHKIIYYITSCLIFAWCAVYLPIGIIISFKTDINTFTPNELLTVMQLFFNSVGMPFKVLFFNLYISGFYKAKKLLSEMDKRCTTLKERVEVHQGVVRCNKAYLIYQFIYTAYTISTFLSAALSGKLPWRIYNPFVDFRESRSSFWKAALNETALMLFAVTQTLMSDIYPLLYGLILRVHLKLLRLRVESLCTDSGKSDAENEQDLIKCIKDHNLIIDYAAAIRPAVTRTIFVQFLLIGICLGLSMINLLFFADIWTGLATVAYINGLMVQTFPFCFVCDLLKKDCELLVSAIFHSNWINSSRSYKSSLRYFLKNAQKSIAFTAGSIFPISTGSNIKVAKLAFSVVTFVNQLNIADRLTKN

>DmOr98b

MLTDKFLRLQSALFRLLGLELLHEQDVGHRYPWRSICCILSVASFMPLTIAFGLQNVQNVEQLTDSLCSVLVDLLALCKIGLFLWLYKDFKFLIGQFYCVLQTETHTAVAEMIVTRESRRDQFISAMYAYCFITAGLSACLMSPLSMLISYHEQVNCSRNFHFPVYPWDNMKLSNYIISYFWNVCAALGVALPTVCVDTLFCSLSHNLCALFQIARHKMMHFEGRNTKETHENLKHVFQLYALCLNLGHFLNEYFRPLICQFVAASLHLCVLCYQLSANILQPALLFYAAFTAAVVGQVSIYCFCGSSIHSECQLFGQAIYESSWPHLLQENLQLVSSLKIAMMRSSLGCPIDGYFFEANRETLITIVRTAISYVTLLRSLA
