# Supplementary material for: Transcriptomics reveal the molecular underpinnings of chemosensory proteins in Chlorops oryzae
Source: BMC Genomics. 2018 Dec 7;19:890. doi: 10.1186/s12864-018-5315-4 (PMC6286535; doi:10.1186/s12864-018-5315-4)
Supplement: Supplementary file 5 — Protein sequences of GRs used to construct phylogenetic tree. (DOCX 49 kb) [file 12864_2018_5315_MOESM5_ESM.docx]

>Co-Cluster-10583.0

MKLEQHFLRFITKKPIYSRKIIIMMALNSLNAIYQTCGIFVDGFMISLNVIDFSTIYIYVFCAIIWNLFHFIIISYFILCVHISIRNQWLRDEFLLLAKNECRVSSIGLNRAFQMHSKLLSLSMQANKVINFYILLRLLVFLGSNALIFYSGLLNGNRYSFTSFFIVTNSLIYVDILNMIKNIEMQQSSQEMLIEAFNNTELEKIWNQECEAYALGYAISRSPVNVCGMFLLNSSTAFAIIQTAVMHGIILAQFDISNRLRERE

>Co-Cluster-10678.0

MDILQSNAVLRFLCQIGCLSQWRIVENKKLSMMPSRLLVSYTIVVITISLALLSHTMVEYKNWTQITEKGIGEMVDVIQMFGMRIVHIVSLIEALFRSKEEMKFFVAAREIDRIFERSLGISIDNW

>Co-Cluster-10826.0

MHHPKNLLYSKEFYPPPKQRRRIFNNDFLEEQLKENPVFTDIKTVLIILKATGLMPIFEKVSHYEIAPPTRSNEYYSFFVRGVVHALTIFYLYSLFTPGASQWFYSYRDTDNINQWIEIILSIVAYSSTVFICGRNAKRFLKIINDILKVDQEIRDRYRIDINNKCGFSVKFLAGIGICQIYIMGLKIYGQLGQLTPTSYIILGFYAVFQNGLSTIFIVFAAALLHIITTRFAFLNIILTGYTFKMQQKIRRPMRRLGEFRPMELFPEDALFAFRMHNKLLRIYKTTNDCCSLIFVCYMGYAFYTITTNTYNLFVQISTGRDISVQIIQWCLIWLILHSSLLTLLSRSCGQATCEVCYEF

>Co-Cluster-14438.0

MERCSSRTIATTTTTTTIAATGSTLHGMLRRKFLVIVLLKLAMTFMGYAFELPVLYRIENIATMDLRHFGETLVGVYLWMGSMFVLDCCVLGLLIVALMYEEMGNFLRRMLNRMGALEDIAAISIANRRASSNIKLNLSKHKCMKLLCECADIITDCDV

>Co-Cluster-14438.1

MKKFQKLFQWQIFYYIYYNFMVILLLFHNCIWMYLHSDVVHKLGLLMVVVKITNLGILLLCADYVVQKFQMPELLCLDVVCSDIDDRWCMSVEAFLGRRQVENLDIKVLGFFKLNNEFILVILSAIVSYLFIIIQFGLTDIKLKGA

>Co-Cluster-9179.0

MKFVRKAMKKWQKATSDSVTNRQEHGIMGAHWIAASKDKEVPLNKIALQLVRRDGDVCVNKIAVQKANVFGISRGSMLSKMHDPSVEGATKPPKILAKGTKDDYMNDGSFHEAVGSVLLMAEFFAMMPVKGVTNAHTNQLSFSWTNIRTLYSLTFIVMTVIDLGLSMYKVLNRPITFNNVEPIIFRSSIIVVCTSALNLSRKWPALMQYWYNIERDLPPYSTQTKKSRMAYQIRMVTFVLMMLSLCEHLLSVIAVISYSNRCPITDNPIKNFFLITNDEIFHVFEYSHWLAWLGKFENILSTFVWNYMDVFVMIVSIGLASKFRQLNDNLIKFKGMHMPESFWSERRIQYRNLCILCEKMDSAISVITMISFSNNLYFICVQLLKSLNRMPSFAHALYFYYSLFFLLGRTLAVSLYSSSVNDESRKPLRILRCVPKGSWCPEAKRFAEEISSDFVALSGMKFFYLTRNLVLSVAGTIVTYELVLIQFHEDNGSWKCNGLDDNVTKRSN

>Co-Cluster-15482.0

MQGLTKILENIANDVNYMQSKSQMRLQKPYYRMERFCILADQLDELAIKYIQLIEDTLKFVDLLTAPYLCALVCHLFGVTAGLYAQYHAIADTLIFEEPYDIFKAFSNLIFLSISFIEIILQAQLGNQSIVLAHKTGYILQSINLLHADIRFKQSVEAFSLLVLVKKFKIQPWGLLDIDVTLLHAMLSAVT

>Co-Cluster-16926.0

MGWKFWVNFTKAENIFQSLRPFTYISLIGLAPFSLQSQQEVRTSAISFAAGVLHFLFFVLCFFLSFHEGDSIISYFFQTNITKLGDATLSLTGIIAMLMIFGFANLKRNSLISIMQNNLIVDQNFIRLGMKMDYTKIFWYSCCMSLVMLLFNGVYLFVSHMLLRSAGITPSFVVFTAFALPHINISIMVFKFLCTTHLAKSRFRMLNALLQDILDAHIEELSAAEFSPTHSSVRIHRTFSHRRRGYPVIMSAPRYSITSIIRQNPESALRQSTNVHNLLCDICETIEDYFTYPLLAIISISFLFIIFDDFYMLEVLLNPSRVT

>Co-Cluster-17319.0

MQSNVEKVGPIRRIKMKELSDLSSSEDEDAAANGEEYPKKSEQEQVYGSERHTSESNLPDLFKLHDKILSLSVTTSSEFGSQTVPYMTVCFVISIFAIFLETKVIFAVGGKSQLLDYVAQIYVIWSLIMMIVAYLVLRLCCNANGMSKKSAMMVHEIMQKKPAFMLANDSYYNKMKAFTLQFLHWEGYFQFNGIGLFTLDYTFIFSAVSAATSYLIVLLQFDMTAILKSEGLL

>Co-Cluster-18362.0

MVNNCFGISILISVANDFIAITSSCYWIFISFEKYSSSLSDLIIATSAVSSIPHLLNVLFLAIICEKTVKSTTNIALRLHNIKTDLLNDYHNTLIEQFSMQLLHQKLAFTAAGFFNVDCTLLYTIVGATTTYLIILIQFHMSSK

>Co-Cluster-3781.130742

MKIVQTVSRLYLNFYAIYYMSKLMPILSVLPYVGLTYMDLTNCILSLDIFVIFLCFYTILKRLEEGLENAVSPERVERFLYFERSIWNCMKEFNHVFQIAIFIFLLQRFVHFWCNTYSSCFFYYAFNGSFPIILGFNCFIILVDLYFLIVGAHCCCAQRERVKRSLLHHRMLRDKDVWLIDIFWLKRFEFCILGMFKLNNGFWIFLVAYSANFVVLILQFGMVNLDRKYKI

>Co-Cluster-3781.13792

MDMKQTDQATRVEIDHFIVAIELNPAIVSLKGYVNVNRELITSSIATITIYLLILIQFKLTLDNAKA

>Co-Cluster-3781.168976

MVDRHEKFKTWVLAGGGVTGQVISNKPNEIVDNYNYLGSESFAFEEFSLAGSALNRVVPISNSTNSFGKIESPIRQRIQKLTSTNMSRESDLNNMGKVEEKLNNFCHLHDEVCEIGKTLNEIWSYPILVLMAYGFLIFTAQLYFLYCATQSQSIPSLFRSAKNAIITTIYLSYAAGKCIYLIYLSWHTSLESKRTGICLHKCGVSADDNMVYEIVNHLSLKLLNHSVDFSACGFFTLDMETLYGV

>Co-Cluster-3781.170278

MDFCAKISLWKRLKIYLFQNFESDSFAYVLYPFLCIFKLFGFAPIKLKNGDIFRERGKPQYSTKAWILSILDIILYIVGFGFGVKHLSQTDALIRLHEETIIAYYTSWVQISALFVLGAVAVISSWTHLSDMQCLCRAIFQIDQHLNTLRGMKIDYKNLRQRLFTEFAIAFIVPASLSMVNCIVIQPLPDEFVLTTSCFWFICFAPIMLMTFKEFQFCNLLNILKLKFELVNRKLEGFGGKYRQWRAIDRNLNFGDSPSACGSDVPLTVMITEAASGSNSISASDLFEEKPNVENLEKLLKIYAKLADAVDLLLRIFSLHLVLLTTVSIGVIIIQCYNLFALSVNSLQMQSHQIFFVISWILVQITVIGLNIQVCSVTFKRMSQTGILLHQIQRPSSNSDSEQRFNHVLQMFSLEVLQRKQVFSAAGFFDMDYKLITSVVFAIRKFVSIFFK

>Co-Cluster-3781.174518

MFGGIHDSEFSVRIENSLLREELKTNRGPSDISEQRLKTMDLHAQDREFNRIKRLQLNSSCGDTVEDVDQFYRDHKLLLTLFRALAVMPILRSSPGRITFSWKSAATIYAIVFWCFMTIVVVIIGRERVHILHTTRQFDEYIYAVIFVIYLVPHFWIPFVGWGVAKEVAVYKTSWGTFQLRFYRVTGTSLQFPRLKSLIVFLSIGCLLVAILFLWSLSFLLDGFPLWHTLAYYHIIIMINMNCSLWYINSRAIKAASTSLAACFHRDMGQDCSAIMISKYRFLWLNLSELLQSLGNAYARTYSTYCIFMFVNIAIAVYGALAEIVDHSQNGSYNISYKEVGLIVDAIYCSTLLFIFCNC

>Co-Cluster-3781.178094

MVAAIVTYLVILIQFMFTEKDNLERNITTLASLNISATMKSPTMFNSSSYF

>Co-Cluster-3781.37967

MEISDSTIGIFYLSKAFGLAPLARYRNSKGFIEIRRSVFFTIYTLALILTLVFLTYRGLLFDANSKIPVRMKSATSKVVTALDVSVVVLATVAGALCGILGLKSIRELNARFRKVDDSLHSYKNFKKDRMRSIFMIVIPFVTITMLMALDVWSWFRTAEGMNVEDNKTELNVQWYIPFYGLYFILTGLHITIANTAFGISLRYHRLNVVLRNTFLSDKKKEEPPRPKITTVKALVDPPSALNASLSKYSSDAIQNEGKAKVMIIRAMAENHSSLAKCVAIVSSSYGIAVLFILVSCLLHLVATAYFLFLELLNKKQNGTIWLQVLWILFHIFRLLMLVEPCHLATKESRKSIQIICEMQRTIHDPILAEELKRFWQQLLVLDAEFTANGLCKVDRNILTSFSSAIATYLVILIQFQSTNG

>Co-Cluster-3781.42334

MMSSMIFLFNIMRSRSSRSLISSFMIAMSYTIITFAMGTIAFLHVGLLDMLRLRYRLIRRILGQFNDHGNARTLSVYRLSIYTRRYTQLILEINDVFSVAVGSGIFNDFVMLTSLVYLICQRIFVGYSKAHEYIFIVLFMAPRLYKVATSSMYGKIVENERKRCVHDIAIYGDELKTCPRFQELFQSFLHWKVQHNYKVTIGRVLNCNLNILFMVLNSITNYVLILIQLQFQQTSIENRVKSIHNSDGRVE

>Co-Cluster-6234.0

MYTIVTKNPSEIIQESMEIHELICEAASTANKYFTYQLLTIISIAFLIIVFDAYYVLETLLGKSKRESKFKTVEFVTFFSCQMILYIIVIISIVEGSNRAIKKSEKTGGIVHSLLNRTKDPEIKEKLQQFSMQLVHLKINFTAAGLFNVDRSLYFTISGALTTYLI

>Co-Cluster-6931.0

MLHCSSRGMMPGLHLHAAIQQNQQLLARVWNLVNCIERYFTTPIVALVLYNGLAITHTANWAYTNVYYGQEHFNLYRVFFTIIIFGNMMLPSLLGQSCIDCYNNFGAIIHSMKTTAMDVGLSMRLREYSLQLMQLKMRFTCGGFFDVNLAFFGKLVLTVMTYVVILIQFKMQGHPEDEEEVTNEHSYENTTNAETLLNSTQ

>Co-Cluster-8807.2

MLNCFCSLWYINCNAFGTASLALADSLQETLKTDKPANKLTELRYLWVDLSHMMQQLGSAYSNMYGMYCLVIFFTTIIATYGSLSEIIDHGATYKEVGLFVIVFYCMSLLYIICNEAHYASRKVGLDFQNKLLNVNMTAVDSATQKEVEMFLIAIAKNPPIMNLDNYANINRELITSNVSFMATYLVVLLQFKLTEQRG

>Co-Cluster-8807.1

MSFWATNISMTSSSSKIRPVLNPNQKQFLQDEIRYREKLEILARSDKKNLADFFVRKPEEINDPVLLDKHDSFYHTTKSLLVLFQIMGVMPIYRNPQKPDLPRTGYSFTSKYFIWAVFVYVLQTIIVVFVLRERVRNFINNPEKRFDEAIYNVIFISLLFTNFLLPVASWRHGPQVAIFKNMWTNYQLKFLKVTGSAIVFPNLYPLTW

>Co-Cluster-9226.0

MDLFIMIISKGVAYRFEQIMMRINKIADQDVSEHVFIEIRKHYVKVCELLDNIDEHLSAIILLSCVNNLYFVCYQLLNIFNKLRWPINYVYFWYSLIYLIGRTAFVFLTAASINDESKAALTALRSVSHRSWCVEVERLIFQMTTQTVALSGKKFYFLTRRLLFGMAGTIVTYELVLLQFDEPNRSQGLPPLCS

>BdorGR58b

MLRARLFRFVLKISYYNSLLVGLLPAPLDGKTLEFRVTRLYLVHSAVIHLFCVLATTYAGYYYFSRDFLTNDPILQWTYSLTHVTKNLFMVVLVKEMWCKREDIKSAYVVYRALETRLKAYTEAASGRINWNVEKRNELHRTTIDQRVENLIIFKFCLAYVLVTMNVYSFLSQHPGTDGRYMPITLISFALHTFVITVSGNFFYIYSQLYRQFSQINSQLKTLFEQLHGQMSRRRVGAEFASHINNLAMLHLTGYRLTQRIFRIGELTLAALLLRLFTTNMRAVYGACLLLSQNQTKNLWLQANELVFTAVFFGDTAMMMGMLDAMLTKCNRTGQLLREHAGLVDTCESNSLRKAVN

>BdorGR64a

MKPSQCFSLMPVQGVGQPNPRHVRFSFKSLRVLITLIFLVASSALNLAMMKHLARIGVNAKNLVGVVFFTCVQSSTILFLSLAQRWPRLIRFWTRTEMIFIRKPYETPKRDLSTRVRRAAITIIFLSAVEHLLYLASAVVSQYRRANFCATLQNSTVHFTFEDYTYKNYDYVYELFPNTTLVGSLILVVNFVCTFVWNYMDLFIMMVGKGIAYRFEQMKMRINNLLNKEVPESIFMEIRDHYVKLLELLEYVDDDLSGIILLSCANNLYFVCYQLLNIFNKLRWPINYVYFWFSLLFLIGRTAFVFLTAASINDEAKDALAVLRRVSAKTWCVEVERLIFQMATTTVALSGKKFYFLTRRLLFGMAGTIVTYELVLLQFDEPNRSKGLPHLCA

>BdorGR64b

MSQQLEDQTFHNCVSKGSVVIGNAEKLIGNILKFSFFFCGLNNYWTTLVYTYACTSHNSVVIRLSFMTTGTISLFVVCMLEHVYFWRLAIKWPFIMRAWRRTEEIFLRPPYRIYATYNMKARIYALTCLVMFSAMVEHSFLVFNSFHKSNLERTQCKYNITFWESLYRRERPHLSKVVPFNFWYLPVVEWINLTLAYPRSFTDAFIICVSVGLASRFHQLHLRIESVHNKALPMLFWTEVREHFLELIHLMRLLNDKIAPLILLACSNNMYFICFQLFNSFQNIGVDLVAVTAFWYSLFFAIFRTILTLFMASSVNDYSKRILCTLRSVPSTSWCVEAQRFSEQLAFDLTAWSGCGFFFITRQLILAMAGTIFTYEVMVTDVINKGSIQQVTSYCRPIEYEAENE

>BdorGR64c

MAKGNRVAASSEPSPQNINTMHHALRPFMMISQVLATLPVSGACRKSYAERVHFSWCTLISCLSLIMIAFSLIDVVLSTKVVMEFGLKLYTVGPFSFSIISAVSVSSFLQLARKWPDLIKHMYRCELSVFLQKCYANGESRNFTNNVRKFGAILLFGAAMEHSVYIGTAIFNNDFQIKKCNLTVDFWKNYYMRERLQIFSIFNYHAWLIPLVQWITISTTFAWNYVDIFLSMIFRCFAIRFRQMHWRIKRHAKKQMPNDFWHEVRNDFMSLVDLLRLFDDGLSTLILVSCCNNLYFICVQIFHSFNNRDNFMKEFYFWFSLLFVLVRILTMMLSAGAVHDEAKQIMSTMYEIPTKFWCLELKRLNEIIIHDLFAFSGKSFFFLTRRLIFAMAGTIVVYELVLIDQVEDKDVVTDFCTSRNV

>BdorGR5a.1

MHKSYLNYTFLKQVVRYLKAKQITELENDDGRKYSVKRFLTGQPQQRRKRLQIINGILPAPRKLSKRQTTIAVDEKQELTKELNEQSKHPKVRGIRRGTRADFIHNGSFHEAVGPLLVIAQCFCLMPVRGILAASPKGLSFRWKSFRTWYCILYTLVTIADTGLTINMVVKGVLDVRNIEPLIFHANILLASIGFLRLAAKWPQLMRKWQRVERHMPPFQSWREREALAVRVHKVTFVLITLSLTEHLLSTISAIHFANYCPSRVDPIESYFMTVVSQIFFVFDYSTWLAWFGKILNVLMTFGWSYMDVFLMIIGIGLSALFEQVQRSLERVKGQVMPESFWTRTRLQYRLICDLIEQVDAAVSAITVLSFANNLYFVCIQLLKSMNTMPSVAHFVYFYASLCFLLARTLAVSLYLSEVNDRSREPLKIIKKVPKEGFHPEVDRLAHEIGMDTVALTGLQFFNITRGLVLTVAGTIVTYELVLIQFHEDQNLWNCN

>BdorGR5a

MLNKKLKKNKEVYKLKPRRYWITRGIRRGTRADFCHNGSFHEAVGPVFVIAQCFGLMPVRGILAATAKGLSFSWISFRTCYCLIYTLLTTACTGLTLNMTVRSALEVDSISPLVFHVNALLVSIGFLRLARKWPQLMRKWQRVELLMPPHQSWREREALSVRVHKVTFVLISLSLTEHLLSTISAIHFAIHCATQSDAVESYFTAVSSHIFLVFDYSTWLAWFGKILNVFMTFGWSYMDVFLMIIGIGLSSLFGQVQSGLELVKGQVMPETYWTRTRLQYRLICDLIEQVDSAVSGITMLSFANNLFFVCIQLLRSINKMASISHFIYFYASLSFLLGRTLAVSLYLSEVNERSREPLGVIKHVPKEGYCAEVDRFGHEIAVDNVALTGLQYFNVTRGLILTVAGTIVTYELVLIQFQKEDKLWKCS

>BdorGR64e

MVRVRNLFRRGTKKDYEHSGSFLEAIGPVLLLAQFFALMPVCGILSKTASKVYFSWKSVRTCYAMLVIFCLGPASLCTIAFAFRERFNFDTVEAIVFYVSIFLIAMAFFQLARKWPALMVKWESIESKLPPLKTEMQRAALAHRIKMITLVATMCSMVEHLLSMLGIIYYVNACPTMPGHPIRSFLYTNWSQYFYFFDYTDWAGIFGKVLNVISTFAWNFNDIFVMAVSVALSARFRQLNEHMLRVAKRPTSEKFWIENRINYRNLCKLCEATDDTISLITLLCFSNNLFFICGKILKSLQKKPSFSHTMYFWFSLGFLLMRTLMLSLYSAEINDESKRPLVVFRSVPSVSWCRELKRFSEEVTTDVVALSGMKFFHLTRGLVLTVAGTIVTYELVLIQFHQDSKLAECVSSIRTLSSPGNNTLH

>BdorGR28a

MAWQFLKRLPQSDSILQSLRPLAYISLIGLAPFRLSVSKEVRTSAFSLAAGILHFFFYVLCFFLSLREGDSIISYFFQTSVTRLGDATLSLSGIIAMVMIFSSIFFKRNLLLTIIQNCLVVDGIFLRLGLKLDYRKILMYSFVTSLGLLLFNFVYLLVSYMLLRSAEIWPSFVVFTTFALPHLNISIMVFKFLCTTHLTRTRFRMMNEVLQDILDSCIEERDAVGLSPMHSVIHLHSTNNMASRHAPSVVQPTATPRTRYSVTSLVRRNPEGALKQVSNVHNLLCDICCTIEDYFNYPMLAIIAISFLFILFDDFYILEAVITPSRVDKFEADEFFAFFITQMLWYVVIILLIVEGSSKTIKESNKTAAIVHKILNISEESAVHDRLLRLSLQLSHRRVVFTAAGLFNLDRTLIFTICGAATCYLIILIQFRYNPTHWDRLNTSSGTVLASG

>BdorGR28b

MRKIVNKSYETTIELANTLQQPAAATRLQRIRQYFITQEVFATLQPLFLITYIYGLTPFRIVKRRNGTSEIRASCFGFCNTAAYVTLYGLCFLNSLLNAESVVGYFLRTNISNAGDTLQICNGIVTGIVIYTTALTQRCKMRRIIEVFNELDLNFANIGVRVKYSRIYRYALVLIVAKILIIAIYCAGVYLLLRSAHVKPSLSRGFERRLVILNKSQQHANFWPVNAVQSTRMDEDTNGIEAQEVRATRPQRVSGLRRFFQAQQLYESVQPLFVITFWHGLTPFFIKSDGAGNKKLKESIFGYINTFLHITIYVACYMLTLINDFETVAGYFFNSGVSRFGDTLQIFSGLIGVTIIYITAMLPKQRLEYSLRTVQDIDLMLHKVGVKIIYTKLLHYSYFSILLVVTVDTVYSCGNFMLLKSANLEPSTPLYVVFTLQHTVISIATMMYHGFVKMLEMRLTMLNEVLKKLAHQWDNSIVKPMPKQRSLQCLDSFSMYTIVTNNPCEIIQESMEIHHMICDAASTANKYFTYQLLTIISIAFLLIVFDAYYVLEILLGKSAHEGKFKTAEFVTFFSCQMILYVIAIVSIVEGSNRAIQKSEKTSGIVHSLLNKAKNAELKEKLQQFSLQLLHLKIHFTAAGLFNIDRTLYFTISGALTTYLIILLQFSNSNVPEHPFPSMEENDTTPIRSLVSNLTIGG

>BdorGR39b

MLHEWQPILKWLAIVGLVPFANNGKRELQQWQRIYTFIMLAANWILTAYGIFEQPMDDEVLVSNYVSVMVFLSQSISLSVCLLEGMCTYRRHFAFLQQSQRIVWLFQQRLQTGLCRWSLRRRQRFKYICYSCVAYGSLSISMVVISIKYYYGYFWYALGCILVLRTRCLLAIIYMDYIDFYMTHLNMKLRSVVNSRMRKSRLCLDVNYRMLESFDYLSQLKLVYSEIYKLTAMFDDLFGWSLCALLTVIFLDITVNSYWTFLTLSRVFEFYFLYLTMSTAFPLATVISFACYAGENCKQQIFTAIVTYLVILLQFRWTYPEDYGDVN

>BdorGR39b.1

MEHELRHWLRLCMFFGIYQRQPKPAFAVARGTLTLPTESHGCRCSAHSDKRRLLTPAHCHLCLIALMLCVIYVHGLHRCGGMPTLMLTWVASVILFSLQVLTNLLILMETVRRRAQHAAFLQTLAAIEDALKLRLRVNVQKPALLHDLRCLIGCFALCSLVGLLLFIISTHWLNYIGFFWHGFWSILTMRVRIIQLLLYVRILQHYLECLYVKLREIVAYHVAPEELLLDINYVRLASLDSLLAVKETYTLIYGAFHMLNYFAGWSLFGIVNCYMFDVSCNVYWTLLSLDGYQNRRYYYVAGPVALLPLVAIARRIAFLLNKLKILRTKQPLTSYRLVLQQLSAQIQLQQIEVTAQHFFVLELRLLMTIFSVASTNLVILVQFLCLELELLSLS

>BdorGR39b.2

MEHELRHWLRLCMFFGIYQRQPKPAFAVARGTLTLPTESHGCRCSAHSDKRRLLTPAHCHLCLIALMLCVIYVHGLHRCGGMPTLMLTWVASVILFSLQVLTNLLILMETVRRRAQHAAFLQTLAAIEDALKLRLRVNVQKPALLHDLRCLIGCFALCSLVGLLLFIISTHWLNYIGFFWHGFWSILTMRVRIIQLLLYVRILQHYLECLYVKLREIVAYHVAPEELLLDINYVRLASLDSLLAVKETYTLIYGAFHMLNYFAGWSLFGIVNCYMFDVSCNVYWTLLSLDGYQNRRYYYVAGPVALLPLVAIARRIAFLLNKLKILRTKQPLTSYRLVLQQLSAQIQLQQIEVTAQHFFVLELRLLMTIFSVASTNLVILVQFLCLELELLSLS

>BdorGR39b.39

MEKCLRFWLRGCAVFGIYVIPIKEHYTALHWQRLSRKKGDRQLAWTEEKATRLTYLLQRLYLAMLAVTVCVLYLHGLYAREIEHGFVLTWLVATLVYTSQVLTHLSIFMAALWKREQHESFLQLLQQIEVSLKLRLKCNTRQSALLHSLRLLLFSLILLSVVGICVFTVVSVWLNDIGYYWHAAWTIVTLRVRILQLLIYARILRHYLDCVCVKLRQVVACRTSPASQLLDINYERFESLEFLLAIKENYTLIFKAVQLFNDFAGWSLFGIISSYMLDFTCHVYWSLLGLDGYGSPYTYLVGMPAALPFSVIGAIITDLVSKLTTVRSTPALKRYSCVVYQFSTQLELQRIEVTAQHFFVLDLRLIMSISTAIATNLVIMIQFLKSENLESSGA

>BdorGR43a

MEITEPTLCVYYVSKALALAPFSVRRNSKGVLDIRRSVMFSVYSASLCLLMVFLTYQGLLFDANSQMPVRMKSATSKVVTALDVSVVVFACSAGVGCGLWGYRATRELNTRLRKVDDSLHSFSNFKRDRILAILMLGLPLIAITSILGLDLSTWLRFAIEMRTPTDDTELNVQWYIPFYSLYFILTGLQINFANTAFGLGRRFRRLNVMLRSSFLKDVDSSQKYVPLKPLITTVKVVSQHPLALHQSLSKLTHVPTQDTAKNKVALLRLLEENHESLGKCMRLVSNSHGVAVLFILVSCLLHLVATSYFLFLELLSKKDSGMVWLQVLWIIFHSLRLILVVEPCHLATVESKKTIQIVCEIERKIHDPILTEEVKKFWQQLLVVDVEFSASGLCRVNRTLLTSFSSAICTYLVILIQFQNTNG

>BdorGR43a.1

MKTWVNNLSSDEETPPHHAFNRLRNHHSKPTVELLRLLVDNYESLYKCVEIFSNTFGFAVLCNLISCMLHIVITAHFLIIAFREFSITIRTYCQMMWLMLHIFRLLLVVEACHKATVESKETIQIVSEIKRTTVELSLSAEFQKFWKHLHIYDVRISVLGICDINRTILTAVSSNNYKKNLKYQLSIFKLSQQVKLGIASDTKFTTSTYENSISATQQG

>BdorGR43a.2

MEINESSLSVFYLSKLLALAPISIQQNAKGVIEIKRSIMFSIYAIALGLIMVILCYEGLLFDANSKVPLRLVRTQINSIKIIPDKAMSLHESIDRLNTESLPKDGLGKTRVMLLRSLAENHESLGKCVQIFSSTFGIAVLCILVSCLLHLVATAYFLFLALLNPNVTGYAWGQVLWIFLHILRLLLVVEPCHMATLESKKTIQIVCEIERKMHEPVLVEEIKKFWQQLLVIDVEFSALGLCRINRNILTALSSAIATYLVILIQFQKASG

>BdorGR8a

MTIQVPSVVRLHIRFFQLIGCFDASLHGHAKVQRVTEQRLVTLTVLLLLLFFVTTVNTFLRTAEFLYTANRFGYFNDVLKVCIAQLTVFVIYMETVLGRHALRSFWQRYALLNKITADKFKRSNDNWRAQLQTYRRFLCIFYGITFFDVSIEVVFHIMRPANRNLLLFWSMFTPFIYMAHFRNMQIILHIEIIRHELKKLRHDIGLLAAYTSFARRIVPFAGFERFVRHKLAEKQLVYQRIYEMLYYFQRAFSMSSMAVLLMIYVRVVVDAYFMFLNQSSGWQILENLLLLPAYLEIPALLLTSQKCMNEVKFIAFELHNIRSSVDNSLISIQIQNFSLQILHQKIRIDGLGISALDGKMLVSIVGSITTYMVFFIQFMPKFKNL

>BdorGR8a.1

MRSTNFLAHNIHKIRHDIEDFNISTRLQSFALQMLHQRIIIDGFGFFVLNCDMARDILGSIATYMIFFIQFMPKFKSF

>BdorGR57a

MGWRDFVYRPRSIYQANALLVSMQFLTACNGFWYHRGRYVVNRWTILYTIAFPNVIFLALLLGLYELFNDPIQRERIEAMDQLKLTIYALEVLMTPLNHVLVMYLMLRKVRGHIALYDRLDALDQQLIREFGVNLNYHKLLRKNLIKVAILPMVHYSAVTWTIIHEVPDRVPQACFFILLYLFSTSGPNHTCYLHAGFAQILSVRFRLLQKLLNADFLLTNFPEVRICEVRLQCLVGMVRSFHEIIDDINDVYRAALVAVLLHDFTLVTNILYMLFGHSIGKGDDGIFFAYGAMWLIVPLHKFISTPNYCSMAVEEGKRCLHLVEQIDIWFPNFKSAKRMVDATMHWRLENKIQFTCGFNMIFNRTIIATITAVVFNYLLILIQFRMTQLMGQQIEEQKNALHDWVGDL

>BdorGR66a

MAIRNQRRLPGLFERIDCVDEDLRQLGITVDNRRVQRGIWLMIAFTFFCEFFIFFSSIWFLVDELKWTTMLWIFTSLPTFYNTLDKIWFLGILLGLRDRFDAINAELERIAEELEKRQNQQLKGELYQEQDLVLQTSLPLRTEQIGDIKLERLVRNAFGESLLEPEPIRNYMLNISHENSLYTFKALQERFISLCQLHDSTCRIAKLLNELWSYPILILMAFGFVVVTSQLYFVYCATQPDHTIPLIFRSAKERSISTVFLTYIGGKCVSLMLYSWKTSQAARRAGICLHKCGVAADTNEVYEIVNHLSLKLLNHAINFSACGFFTLDMGTLYAVCGAITSYLIILIQFDMAAQQVRISKELAAANETTAIALVTENYTIGMMESTTPWD

>BdorGR66a.1

MAHQTVQPILVHFGTLFTFCKLLGLYPHDLQAFRGIHTLQSSKIGTAIVVATMLAVVVLYNLLIYFFSSEDHDLKASQSTLTFVIGIFLTYIGLGMMITDQLSALRNQSKTGELYERIRAVDEQLLKENVYVDISKTAKNILLMIVLTVVSELIILISTYITLVDLTDWKSILWLFSCFPTLYNSLDKIWFANTLSALKQRFFVINTALEDMVESHERLKRWTENGGDGGGQIFRRPSIANVSIDPSLEYLYKELTHMEAVKAYNMARNKISPSNVILQDILVLWSYPILVLMAYGFLIFTAQLYFLYCATQGQAIPSLFRSAKSATITTIFLSYTAGKCIYLIYLSWKTSLESKRTGICLHKCGVVADNNLLYEIVNHLSLKLLNHSVDFSACGFFTLDMETLYGVSGGITSYLIILIQFNLAAQQAKDASNAAETNYNTMLTTEAGNTTALMDYFTTTFMPYAQTELY

>BdorGR93a

MSQYNHTELFAYRLLRVLYKYGHFLSVFGWKLQKQKMELRQGKRWIRIIRIIWRIWLSLIFASLIPKMMAPYVRHIGNSFLMFFADVQVTTVTLFSILSFVIHECSERKIFQIINKLVNMYERISTKSGFEQILGRTFVISIILKFLLSAFGLMYEIPLLVEGTGLMSFLAGIYLWLSTIYILDCCFVGFMVIRQMYIAMATYLERMLERMSRIESEEPQQRLSKHQRMKELCXCSESIDDSNNIYSVLYELTKEFHHIFRWQVLYYIYYNFVIILMLMHRFISRYIETGAVDLMAFFSTVFKFCNLAFLIFSTDGVVLKSQLTDLLNLDLVCSDIDARWDESVSLGARFEFPGIHIEKLSMQLADALFYASV

>BdorGR98b

MTRILTIGLNLMQIMVQVVLYTQALTGHQQLRSILTQVAQIESDIRKHYTTVCSLCAIRWRFCLRIGIWWLVVCVFLLQLNYALADHNLAPRFRWINVVFSMLSQIKVVEYCVCVLLIQELLHLVYQQLVHLRRELVLCESVDLRWSLYVELQTNQQILARIWQLLSKVERYFCIPISLVFFYHGFTITQTILWGYTNLEYEDFNLRLCRTAFTIMIIASLFIPCYLSQCCIDEYNRFGTLLHKLTVGIDVNLSMRLQEYSFQLMHQQMLFTCGGLFDINLKNFGTIILTITIYVIIFIQFKLQAVKERKIQQGN

>BdorGR89a

MSNWTQRRRRLCSVAFFFSLRAVLLTAQVMLLAPLLRSKPQGAYRTHAVLTCFALLVLLLLCGATPFLLRIIASTYERVGVQFDAIFLLIAMTSQVSDISIVLISMLSQICQRRALCEFLNQLQDTVQRVRTIHGRNFINARVLLLLWLQLGLTLYDMLTQLVFLAKLAFKIAPWQIVVNLLSLYLQQCRATLQLVIMCCVLLLIACYTQLAECLERFSEDSSLELSTYEDLVWLQMVLHKLTLQLKDVFQLPLFLLVVGEFISVLANLYAQLHYYVTTKAWWFAFVFYCAKISIELYLLIHVVYLCCVLHEKVTNLFLDRDLDFEEPTLAHCRYDLTHTDALWPQPIRFYILGMFELNNEFWLFLVSYSVNFIVIIVQFGFFT

>BdorGR93c

MGAIIYIYGTLMMWYTNDPFYNLITFLQTNLMATGSVVMSICLLRSGKKFVCIINSLFGLFRRVQCLTPHRQVMGVHQLIFLLIIVICVGNSAFGFLLILKYTDWRELAVVTSKLYLETGLVVSLHIASIGYMCIGALYNCMNRYMREDLLPRARCLDHVLRRKKTYRPPRYTRKLIRLTRELNNCARIYNDIYNLATTFHQSIRYQILFALLFQFSLLTTVIYSILYMYTVITAIEWDAVFFCLLIIIEVLIMILSAYSAVQDGIAANKLSLDTVYMGGDTEWNRSVEIFINRMNLYEFKPNVLGFFDISSDILVMFLSASVTYLTYILQNTKLSQKL

>BdorGR59d

MYQTQRSLMLPRIIGYFNVLAIFLGLTRFRIDFKAQRVLQSRHVARFVICVNIAYLLALPVFYFYEAMYTSSQFDENLVFLTESANMFISIVAAALSVLMRGRREGIHLELAETIMALDRRHFRSMYAGQRPVTHRSDHLLYFKMFTLCLQTVVPFYDWLTVSDALDVPAFLQTFYYVYSQSILAMALFMYYHWLWVLRKRFCYLNLTVIDLLRRLQQMPIGGTQQRSVIEATTILIKISQVHALLNRLLSHLNRDYKVQILAVLLKNVIECISFGYYLTLGLSGSMDWEFNVDTLLFLSYAIILFVDLNSIYWISHMITDAQQQMSEIMRRFQLLPSLGEEFDQQCELFVMQLTGQQMDSKLAGMFQMNRERALAFWAYSLTQIIVLVQFDIGLQLQPSTDNAVLNRLDKLFRVNDDEE

>BdorGR36b

MYRITYWLLYGHYCYSLALGTLRFRYDFNSNKVHITPFITYYSAVMNVLALTTLPWTYYTAVCTLLRNERVGRLLLYVFIAAKTLRLLAVALVIVYNWQRRRDILKFIRDINHLRYVYFSRFPVPFEWQRRFEVKIIKNIISGVLVDALRLITVLGVNKNQMNVPFIISNVLFSLISNILYLNMSHYYFALRTITLFGNVLNRQFADIIKAAKSVTHLRDQQLCRKQFKARCLTLSRELDELALVHIQLQSLALRINDILKWQGVGVLLNMFILNISLIHTIYSVLWVGEFTISQYGWVSVLMGVYYLVYTVDMVDFLITTLEHVGVFHKTSALLELHTNLPQMDKEFERSVESFQLQLSINEYHLSLLGLFNFNSECVFSMFASVVVNSILIIQFDYQYFQ

>BdorGR10a

MSFWERHKNNIYKYGHIYATLYGLMVINYIPQRPTNTFTHRLAVIYGHALSVCLILVLPIYFARNISVLTEAGDQRGHLLLLVNFANTLLKYITVVVTYVANFAHYAAIRAVTRVRQQLEDDFNGSLRALPAYDERPRKQFEAMLLFKFGLINAMMAVQVANILYQHFNGAHPVRVHIAVYTFVLWNYTENMADYFYFINSSALKFYQQLNQQLHQVLRENKLLHYFRLRGQRRGTVPHLCGLLCDRLDTLAQRYRQINQLYQDSLTMHQFQILGLVFITLISNLTNSFILFNLFVKHSESGVSPAIVLNAVHAIIFYVDTYIVALVSENINLELRNINQTLRQFSQLAVIDARLQQTVEGFALYVMNNRVEVPICGLFMLDRSLTFVTASTALSYFITLVQFDMNLT

>BdorGR23a.1

MYYRNIFASFARYAYLVMMYLQLLQPVPRLTWLTSICRVVWIAWLIKPGYDVMHELYTETIEKLAYIVGSFLFVFKMLTNIAAYVESMCKTKHCQHLERLEIEVDALLRDYIDNAETLERKIWLKVFYSMLLQFVYDILQLVLAFTCYMSPVFYYVVPMLAIERARYAQISLTIERQNARARSLIALLRLLVKANRPKHKYTSDVWQPYAAWEYESLNFLRLLQGRLCELYQCVGDCYGWSITVLLFTTFFTVVANIFWCIEILSRGFRFGQFMYDALTMLRLSTLAVTLLITAEKTRQHNRQISGLIFKLAKPLGNKAYNDLVSDFSLQCLHQPFMINVKGFFRLNLPLLGDLFALSITYLIILMQFLMNEKINNKMTFKSKLIINATEISTISPNF

>BdorGR23a

MGGLMFNLLFIAQMVTHISILLESFFMDSKRAETTKIHKEFEKRFNKEFGRKHAYSDFSSESCLSSILSVLIVMVNVSFKIYSLYVYQLTLKNTLLVWHTLPNALAVQFRILELILSTTVLNEYAIILCRKLNEMGERNIRRVFGHYLRGLPVSKGCIIRVMPPTLQPSEEADEQKLRVYKKLYGDIYNMFKTINESHGWSLLAFMIMYFIYFTINSYWVIFSLIARLSEHVTLIRNLGFLVVVVTLLWILCWQSQNSQEQSRQIGCIMFKLVKPLGNKCYNDLVTDFSLQTLHQQYIITAKEFFNLNLTLLGSMVASIVTYLVILIQFMFAEKNKANRNEILSSIVNNENSTASVSN

>BdorGR59b

MIMHKPWYQRLYNYATIALGMTSFNYNATSDTYKQQLWMQALALVANLWTILSLPFIFWNAMKRSSTIQWNPMMTNIDYITNIIRSLAVLYIILKRPKIDLVNYEVFCKYRRLRKTYLALLPRVAFIERRMNRLICAKYINVSILVIITAVIFLQGSEFEWTWSNYYFLLMVLQTTTILRLTIFNFFWFLWSICHSLKYVQWHIRHLLQETSDSVRHGKRWRLNMLAGDVHRILEAHWQLSELAWVLMRNYGFLALAVFLDEITSLERQLYCGIIFTFDHRAELHLLVVGTIYFFAMVFNIYLDFLMCDITVKTYYACINQLDSISAMNVQCKALEETCTDFALNAALSKFSINIYGMFDMNHRATLAVLRNAVMHALILIQFDYIVDPAKRQAG

>BdorGR2a

MDILKSLGTLHRCLQLSALSQWMVERKTGKVYLSRLLECYAICTLLIGFAILILDIFSDGDYFRMSENDVGHRLDYIQLMGIRVAHFVSVLEAYLRRHGQNTFVKQVREMDRIFECSLNVDVNNGPLRLKILRRGVVMILIFVSVEALKLLVYFLASGRNFSIYWLFYMLPFLICGVRYFQIFTSIMIVRHRLDKLVTAINELNLLNSKPKQYTADSSNTNIKYISQASTVYEITEYLKHQYDMENTDMKRLLIIRDLYNRLWELTATLNKDFGISILTNVGNDFISITTNCYWMFLQFNTFSASLEKFMQISSNLIWSITHLFNVLMLAMLCERTVQRTTAIAMGLHRIETNIWNDNHNTVIEQFSLQLLHQKLAFSAAGFFDINCSLLYTIAGATTTYLIILIQFHMSDDK

>BdorGR2a.1

MIYTMGFCLGILRLRAVPYKHGDGISRLTSWAQVFTLFGLGLIAWVTAWTHMDEQCRLHALITRIDQHLREIDEIQFDYKSLRRRLFWQLGLQLLIAFPLSMVNCIIILPDEVAFSPFSTCYIFICFMPISVLIFKQFQFYNLMHVLKTKFDLINNKLSKFNRDTRFTSERRHIVTVRKSNSVSEVPLTDVPAASQKADMLNEEPNVDLLQKLLTIYSNVSDGIDLVLRIFGWHLLFLTAVSFGVITVQSYNLFSLISHSLVIPTYHVVFIVSWILVQVMAFAVNILICGKTSRMMDTTISILHKIRLSSNEAPNACVFYQILQIFSMEVLQRKRNFNAAGFFDMDYKLITSIIASATTYLVIIIQFHLTNIPDCHFPK

>BdorGR47b

MKVPKKRVKNLSLYRCFEWIYMILYYTGCLCFQLRGESFQLTKANIIYTNFIQISLIFGFLGSVLLKYMDDESYNAMFNRLSPVFKFILAMECFVSAMTYIAVCIKMQTNRYKHLKLLREFKELDNKXAIDFNYIKWNYHKTMRKFTIFTLIGMTYYFTVSFIYLFKLSNCNCDYVATFVFALCYASITLAPGCIFFLHLGKMDLLRIRYRLIKRLLKQQYVLAVDLKHQHKFEMRISRLIDYCKSYIQLILQVNDVFGIVSGFSLFHDFAMLTNMTFLMCQKATETKTTAKEYIFIFLFMLPRIYKVIIYAVYGYVAQRERRDCTHEVRMCERYLTCSKVTRSKLEAFLHWQMQNTYSFLVGKVTTCNLFLLYATVNNIASSVLILIQLQFQQNSITNRMKNQPMLKDVEFI

>BdorGR59f

MLPTIVHGSSNKRKLQHRSQFSPSTKDTSVDGLETQLYRAVHYFLLLSEIFVSLPYDAHQHLPTDNNNKHSIWLILHIVWCITIYASLIVAIYSESTNINIDLPTIHKPLYFGEYLIYILHIFLIITSSYWGRHKCRRFLHIIAEFDYTLIDFKRQPKYERLTCFLKTHVVLVIFFVFWTATVNYFYSNGIFLNYIRSLVVYLLPNLILSISLIQYYTLLYTIAQRSRRLNEILLGELSQNNSPRFLNEKLQRIRLLYSALQVFTKEVNNSFASSVVLVYIGSFTNLAVNIFLIYKYVDEWNTSAMSLVFYSLVWTIMHIAKMFLILYYNQSIQNQNSSTILIMNEIGGQNTEMEDTVTHFVLQLIINTRTNVVCGVAELNLKFITTLLTAMSTVFIFLLQYDITYEALKLTHNSGNPL

>BdorGR68a

MKIIKDMKYVNFAGQVFGLVPIYEVKRGELRISLRGHYFTYFINILLFMISLAVSWVVLNSDSGFSVFRNLEGSDQTTEALFCLLSCNIIIFVSSTNSRRYCGILQEIGKMDAYLVAKGFTAAYTCYLLTCFLIINAAGLLYTAYYYMDMLSDHFHYHQAILLGIYSLQLLISNLYAICLRVLLGNISKRIDFVNVQLEISTKSDLAVESNWRQMSYHIEMLCKYRYVTDKINKSGGIAMLSYMSVAFYILTKQSYMAFMTVVQPMGFEKRYDILGLSVAWIFAEFVTIAVICSACDAVASEANNTGQILARVYAKGKDYQDIVDKFLSKSIKQEVQFTAYGFFSIDNTTLFKIFSAVTTYLVILIQFKQLEDSKAEE

>BdorGR22e

MCSETTLRVLRQTRQIVADTCISTQFLLSTVHGLFPFKYDSGTRRLTSTKLLNCYWPLVNIAIVLITPYIYFLKPQVNQVRFIYDKPLNKLLAHIHYMLGVCILLVIIIANSYHRKELFKLHNDLVQMQRRQQRWQQKWTVKSNSQIEAFYYNAIIAKSLMSLLQVASNVGGKLTMNKNPSLEYFIYVFFMLVLKNVTLLTVANFHFALLNIYRQLQQVNWNFQEVLRLWSVRAPAVSNIPLEHVTDIAFAAFAGEAKQPNGSRNFGVSAIADLCGQYVQICRLARRVCKHYEWQVLLFLIIILFGNVMSTFYFLVYLGGKVVPKELFSPTLFLQFYLINILDLYCYMLICERSMASSKETGFLLKELTQLKSLPKGLQHEFEMLTIFMAGETVRFRFCGLLEWNFRTGASYMTATILYLIVLVQFDYNNL

>BdorGR22e.1

MRARLRRRFTDLILRATIRSANVLAILPFRYNKTKRRVESSKLLLKYTISINVIILFLLIWTRPTKDRLNVEILQRKPIVALVNYLNFYGTIYTIVLIMWTNWRGRKSLLDFFNTCLIMECECFRIYSELKRECEAFDNYIIWKAVLTLLQNASFINTVYDFRGFSWVAQVLLIIFTYFLLNMLLVTIQLFIICILFQYRCFWVLNRRLQHLVEVELKQSGREHIKAEINMLAGIYMRLLGIFRRCTNMYEQQALLMVAVLTGANILSLFFAKLIWTGKVMEMSVWSICDNLQMVLINVVDFWLTITICELTVNTSRITANLLRNFNDFRRLDKYLERNVEQFSFICCDNQLKFRLCGLIDLNHVTGCRILFTMILYFIYFVQVDYNAT

>BdorGR22e.2

MFLLALLTNIIFHVSTHFFLAVLFTYRFIWVLNRRLASMAGNEVSSSRLRVLSIEIDNIAYIYTRLIRLCAHYTRIHQYQLLLLFGSMTLCNIEVLFYVRLLWSGKVNELNGFNVFAILQIVVVNILDFWLTITICEQALVTSQKTLEILRGFNENPKYLIDVERSLESFAIICSNTKLRFNLCGLFDINHSSGLNFLLTMILYLIYLIQYYHENL

>BdorGR22e.3

MMELDLQQYNKLMALMIRAIVVSHVYTLITIVVINWRKYKSVLHIFNEFAAIERIYLAKHADLARSCSAFDACIIWKGVATLLQNISFIFVIFETTSELSMHAVIVLGFALTMGNVIFLVVLHFYNFVVITYRCLWILQQRLQYLANQSAMPTTLRNVTCEVYEITGIYLRLMKLCKSFGSVYGQQLLTSNFAIMCTNVQSLYHLRIIWSDKVNDLTAWDMFYTSQAVLINIFDFWLTIAACELALGKARDIAQLLRTFNDFEKLDVELEKSLDILATVCRNNTPKFRLCGLVDLNHLAGLKALLTMILYLIYLF

>CstyGR108

MRFLPKLERKFRRWKKLKKPPLLRKLDTLFKSAHKGHRPEDSAKRVQKGNGKDVKNELSYKSREQFMYNGSFHEAVGSVLLTAQCFAMMPVRGVTAKHPSSLRFSWRYVRTCCCLLFMASMLINLSLTIYKVLHGSITFNSIRPLIFKSCIFLVCGKALSLARQWPELMMKWYQIEKDLPQYQTQFEKRRMAQTIRMVMLVGMMLSFAEHLLSIVSAINYAEFCSRTDDPIRNFFELTNDEIFYALNYSAPLALWGKLQNVYSTFIWNYMDILVMIVSIGLASKFRQLNDNLLRFKGLHMAAAYWSERRIQYRNICTLCSTMDDAISFITIISFSNNLYFICVQLLRSLNTMPSVAHTVYFYFSLFFLIGRTLAVSLYAASVHDESRRPLRYLRCVPKESWCPEAMRFAEEITSDMVALSGMKFFNLTRKLVLSVAGTIVTYELVLIQFHEDQDLWTCDQSTYS

>CstyGR107

MGFLGIRLSRQPIHGKGKLVHALLIMLIIMLTFFGLFANRFTLRGRQRFVFSKKILAYAMMVTAIFMSIYVRQIYKDYMNSELNLRDAVKLYSYMNITVATINFFTQMIMSKTAGEMMSTVPLFKTLNELHIESGAIRKSVMLALIKVLGFPLVLEFALVMQQRRNEPDAYWTWTLYKLFPMIISNFLNNCYFGAMIIAKNIVEAVNDRLKMVVDQVNCMQLPINREHYSKYYCIQRYCTLADELDDLAIKYKIICVKSTKYMALMSLSIILSLICHLLGITVGAFNQYYGIAESVIGGKQFDGFGALINLVFLTISLLEIALLTYVSNDILLVIRTTGTSLQEINLQNADWRFRQSVHAFALQINTIKYKIKPMGLLEMDISLMTSVLSAVASFTLILVQSDLSQRFK

>CstyGR106

MASNMEGQLGWKLRHMFAKNPKQVDENSPTKQTDVGNFDFNVIRCEKPTDYANLEIFHRAVYPIMLVGQCFALMPLTGIWNPSPRHVRFRFKSLQMITALVFMAACSVLTLGMLKHLLRIGVTANNFVGQVFFTCVQCACILFIDLARHWPPLIRYWTRQELVFTKPPYEVLKRNLNQRVRQPALIIIAMSMVEHGLYLTSAIVSYQQRIHICSMTHNSTAVASFDDYIKNNYIYVFQVLPYSRFIAVYILLVNGTCTFIWNYMDLFIMMISKALAYRFEQISKRICRLEQENQVPESTFIEIREHYVRMCELLERVDNVLSSIILLSCANNLYFICCQLLNIFNKLRWPINYVYFWYSVLYLVGRTACVFLTAATINDESKTALTVLRRVSSRNWCVEVERLIFQMSTQTVALSGKKFYFLTRRLLFGMAGTIVTYELVLLQFDEPNRRKGLLPLCA

>CstyGR105

MWRMQMRRQRIGRRCQRLRQQLQQHGRAWLEQLQQRRAWRVDCARPRHSHSPDGSFHQAVAPVLVLAQCFSLMPVRGVRAASAQGLYFSRHSWRTWYSLAYICCTSVDTLFTINLAVHGTLDVRSVEPIVFHGSILWGSYHFLQLARRWPALMRRWAHVEQQLPGYENWRQREHLTRRIHSVSLALLTLSLMEHLLSILSAVYHDYCPMRKDPIESYLYADAQQLFYVFPYSNWLGWLGKIQNVMLTFGWNYIDLFVIIVGMGLSELLARLKHQLQHLVERPMPELFWTYVRTRYRSIVELIYDVDEAVSGIMLISFGSNLYFVCLQLLKSINKMPSIVHAVYFYFSLSFLIGRSLSVLLFVSSVHDRAREPLRLLQLVPPGGYHGEVSRLASELSSDNVALSGLRFFSITRKLCLAVAGSIVTYELVLIQFHEDEKSWDCVAQPPLH

>CstyGR103

MWRMQMRRQRIGRRCQRLRQQLQQHGRAWLEQLQQRRAWRVDCARPRHSHSPDGSFHQAVAPVLVLAQCFSLMPVRGVRAASAQGLYFSRHSWRTWYSLAYICCTSVDTLFTINLAVHGTLDVRSVEPIVFHGSILWGSYHFLQLARRWPALMRRWAHVEQQLPGYENWRQREHLTRRIHSVSLALLTLSLMEHLLSILSAVYHDYCPMRKDPIESYLYADAQQLFYVFPYSNWLGWLGKIQNVMLTFGWNYIDLFVIIVGMGLSELLARLKHQLQHLVERPMPELFWTYVRTRYRSIVELIYDVDEAVSGIMLISFGSNLYFVCLQLLKSINKMPSIVHAVYFYFSLSFLIGRSLSVLLFVSSVHDRAREPLRLLQLVPPGGYHGEVSRLASELSSDNVALSGLRFFSITRKLCLAVAGSIVTYELVLIQFHEDEKSWDCVAQPPLH

>CstyGR104

MEISQPSIGIFYVSKVLALAPYATARNSKGQVEIGRSWLFTVYSATLTVVMVFLTYRGLLFDANSEIPVRMKSATSKVVTALDVSVVVMAIVSGVYCGLFSLNDTLELNDRLNKIDSTLNAYNNFRRDRWRALGMAAVSLVAISLLVGLDVGTWMRIAQDMNIAQSDTELNVHWYIPFYSLYFILTGLQVNFANTAYGLGRRFGRLNRMLSSSFLAENNATSAIKTQKVSTVKNISLNRPSMPSALHASLTKLNGETQASESTAKNKGLLLKSLADSHESLGKCVHLLSNSFGIAVLFILVSCLLHLVATAYFLFLELLSKRDNGYLWVQMLWICFHFLRLLMVVEPCHLAARESRKTIQIVCEIERKVHEPILAEAVKKFWQQLLVVDADFSACGLCRVNRTILTSFASAIATYLVILIQFQRTNG

>CstyGR100

MEISETNIGIFYVSKLLALAPYSAKKNSKGQLEITRSWLFSIYSVCLCLIMVFLTYRGLLFDANSNIPVRMKSATSKVVTASDVSVVVLAIVTGVYCGMFGLRATQELNTRLDKIDSTLSPYNNVKKDRWRAYAMATVSLIVIGILLGLDVGTWVRMAQEMNISDEDTELNVQWYIPFYSLYFILTGLHINFANTAYGLGRRYKRLNQMLRTSYLSADKSYGNATNLVKVSTVKSISFKPMMPMALHASLTKLNTETLPNESATKNKSLLIRAMADNHESLGKCVRLLSRFYGIAVLFILVSCLLHLVATAYFLFLEMLNKRDNGYVWVQMLWIIFHFLRLLMVVEPCHLAAREARKTIQIVCEIERKVFEPILVEETKKFWQQLLVDDAEFSASGLCRVNRTILTSFASAIATYLVILIQFQKTNG

>CstyGR101

MAFKLWERFSQADNVFQALRPLTFISLLGLAPFRLNLNPRKEVQTSKFSFFAGIVHFLFFVLCFGISVKEGDSIIGYFFQTNITRFSDGTLRLTGILAMSTIFGFAMFKRQRLVSIIQNNIVVDEIFVRLGMKLDYRRILLSSFLISLGMLLFNVIYLCVSYSLLVSATISPSFVTFTTFALPHINISLMVFKFLCTTDLARSRFSMLNEILQDILDAHIEQLSALELSPMHSVVNHRRYSHRLRNLISTPMKRYSVTSVIRLNPEYAIKQVSNIHNLLCDICQTIEEYFTYPLLGIIAISFLFILFDDFYILEAILNPKRLDVFEADEFFAFFLMQLIWYIVIIVLIVEGSSRTILHSSYTAAIVHKILNITDDPELRDRLFRLSLQLSHRKVLFTAAGLFRLDRTLIFTITGAATCYLIILIQFRFTHHMDDTSSNSTNNLHSIHLGD

>CstyGR102

MRWNGCRYRYKTDQTPQRRVEKNFIRRHCPTKTMVIKESEFDDSLGYALLRRDMGTVWDTAKDERMVNGTMDPELIQRAKERAVRAQLNSADGDTCETHDQFYRDHKLLLVLFRGLAVMPITRSVPGRITFSWRSAASIYAFCFYLVSTVIVLVVGYERIKVFQTTTKFDEYIYGILFVIFLVPHFWIPFVGWGVAKQVAIYKTMWGAFQVRYYRVTGTSLQFPHLKLLIVFLSIGCLVCAIVFLLSLSFLLEGFALWHTSAYYHIITMLNMNSALWYINSRGIRVASSSLSRCFRQDVAIECTAAMISRYRFLWLNLSELLQALGNAYARTYSTYCLFMFVNITVAIYGALSEIIDHGFGFSFKEIGLIVDTVYCSTLLFIFCDCSHNATLQVAQGVQDTLLSINLLKVDQPTQKEIDLFIQAIEMNPAIVSLKGYAEVNRELLTSSIATIAIYLIVLLQFKLSLISQQIPVEIIENVKLLQKQ

>CstyGR93a

MAPTGSENWSRFLLLLVYRVARLLSVLSSTLDREKLQLKRPKSGSSIKLFSIIWRCLVVLIYASVWPALSAHLVESKPESYAELFAAIQAASVSVLAVVSFIIQASGESQFRDVLNRYLSLYERIWSATRLPHLFPTKFVVFFLLKTLVTVVGCFHELYPMLNAKHFTEIVAAVAVIFGIYMWLGTLFVLDVCFLGFLVSGILYEHMANNISVMLERMQIIDSREEKDRMSKYRKMRLLCDYADELDELAMIYSELYAVTIAFRRMLQWQILFCIYYNFINICLMLYQCIFRYLKDDESALISLAMAFVKMANLVLLIMCADYAVGESQKPKVFPLDVVCTDIDQRWDKSVETFLCQLQTQQLEIKVLGFFHLNNEFILVILSAVVTYLFIMIQFGITGGFEASEEIRKQFEAQ

>CstyGR66a

MAQAENAVQPLLRQFHQLFFISKIAGILPQDLEKFRSRNLLERSRTLTFVIGLFLTYIGLIMMASDQLTALRNQGRIGELYERIRLVDERLYKEGCVMDNSTIGRRIRIMLIMTVIFELSILVSTYVKLVDYSQWMSLLWIVSAIPTFINTLDKIWFAVSLYALKERFEAINATLEELVDMHEKHKLWLQGNQEVPPPLDSSQPPQYDSNLEYLYKELGGMDIGFIGKSSVSGSGKNKVAPVAHSMNSFGEAINAASRKPPPPPLATNMVHESELGNAAKVEEKLNNLCQVHDEICEIGKALNELWSYPILSLMAYGFLIFTAQLYFLYCATQFQSIPSLFRSAKNPFITVIALSYTSGKCVYLIYLSWKTSQASKRTGISLHKCGVVADDNLLYEIVNHLSLKLLNHSVDFSACGFFTLDMETLYGVSGGITSYLIILIQFNLAAQQAKEAIQTFNSLNDTAGLVGAATDMDNISSTLHDFVTTTMTPAG

>CstyGR64e

MARTTGNQAKRRRCVARIKFWRRSRVASDVTLGILKYKDPSKPLKRSLAFSIIKSFIRRLKKEDYKYSGTFQEAIKPVLIIAQIFALMPVRGVSSKFAEDLSFAWSSIRTYYALVTILCFGISSGYNVAFVANESFSFDSVETLVFYFSIFLISLSFLQLARKWPALAQEWQLVEAKLPPLKLSKDRKSLAQHIKVITIVATTCSLVEHMLSMMSLDYYVSSCPKWPERPIDSFLYLNLSAVFYFVDYTRFLGIIGKVVNVLSTFAWNFNDIFVMAVSVALASRFRLLNDYMLREAKMPTTANYWMQCRINFRSLCKLCELVDDAISGITLLCFSNNLYFICGKILKSMQTKPSISHTLYFWFSLAYLLGRTLILSLYSSSINDESKRPLSIFRLVPREYWCDELKRFSEEVHMDNVALTGMKFFRLTRGVVISVAGTIVTYELILLQFNHDEKVLGCNEG

>CstyGR64b

MPQGETFHRAVSKVLFISQIYGLLPVSNVRALDVEDIRYRWLSPRIFYSALIIALNICEFGAVLNYVGQVAINFHNSSTLSLYVVCLLEHFFFWRLAIQWPSIMRSWHSVEQLFLRVPYRFYGEYRMKKRIYIVFAVVMLSALAEHCLLLTNSFHLSNMERTQCKNNVTYFESIYRWERPHLYMILPYHFWMLPFLEWINETIAYPRSFTDCFIMCIGIGLAARFHQLYRRIAAVHRKVMPAVFWTEVRQHYLALKRLVRLLDAAIAPLVLLAFGNNMSFICFQLFNSFKNIGVDFMVMLAFWYSLVFAVVRTLLTIFVASSINDFERKIVTALRDVPSRAWSIEVQRFSEHLGNDMTALSGSGFFYLTRSLVLAMGTTIITYELMISDVINQGGIRQKTQYCREF

>CstyGR64a

MKGPTLNLRKTPSKDNGVKQVESLAKPETPPPKFVEDSHLAFNVLASEKLPNYASLDLFHRAVFQLMFLAQCVAIMPLVGIRESNPRRVRFAYKSIPMLVTLIFMAATSILFLSMFTHLIKIGITAKNFVGLVFFGCVLAAYVVFIRLAKKWPAVVRIWTRTEMVFTKPPYEVPKRNLSRRVQLAALAIIGLSLGEHALYQVSAILSYTRRIQMCANITTVPSFNNYMQTNYDYVFQLLPYSPIIAVLVLLINGAFTFVWNYMDLFIMMISKGLSYRFEQITARIRKLEHEEVSESVFIQIREHYVKMCELLEFVDSAMSSLILLSCVNNLYFVCYQLLNVFNKLRWPINYVYFWYSLLYLIGRTAFVFLTAADINEESKRGLGVLRRVSSRSWCVEVERLIFQMTTQTVALSGKKFYFLTRRLLFGMAGTIVTYELVLLQFDEPNRRKGLQPLCA

>CstyGR28b

MSFYFCEIFKPRDAFGAEQTLLLYTYLLGLTPFRLRGQAGERQFHLSKIGYLNAFLQLSFFSYCFLAALIEQQSIVGYFFKSEISQMGDSLQKFIGMTGMSILFLCSSIRVRLLIHIWDRISYIDDRFLNLGVCFNYPAIMRLRLLQIFLINGVQLGYLISSNWMLLGNDVRPIYTAIVAFYVPQIFLLSIVMLFNATLHRLWQHFTVLNQVLKNLAHQWDTRSLKAVNQKQRSLQCLDSFSMYTIVTKDPAEIIQESMEIHHLICEAAATANKYFTYQLLTIISIAFLIIVFDAYYVLETLLGKSKRESKFKTVEFVTFFSCQMILYLIAIISIVEGSNRAIKKSEKTGGIVHSLLNKTKSAEVKEKLQQFSMQLMHLKINFTAAGLFNIDRTLYFTISGALTTYLIILLQFTSNSPNNGYGNGSSCCETFNNMTNHTL

>CstyGR63a

MWNNYNRKKKQDAIFLNVKPVANNISVRKYSNGLLDRLDSGFGNNPKEKRTSRQSISTIDSLNQQFIPNIFFRNVAPIKWFLSMIGAFPMKRSGAGKAKFIFGSIIFAYSVAFFTFLTIYVAYVANNRIIIVTSLSGPFEEAVIAYLFLVNILPIFLIPIMWWETRKICSLINDWDDFEILYYQISGHSVPLNLRRRSRNIAIALPLLSILSVVITHITMADFDLIQVVPYCILDNLTAMLGAWWYLICEALSTTANILAERFQKALRHIGPAAMVADYRALWLRLSKLTRDTGTATCYTFTFLNLYLFFIITLSIYGLMSQLSEGFGIKDIGLAITALWNVCLLFFICDQAHYASFNVRTNFQKKLLMVELNWMNSDAQTEINMFLRATEINPSNINCGGFFDVNRNLFKGLLTTMVTYLVVLLQFQISIPTTDLRSSNRNITVSEDISMSDENSEEMEMDVMTTVMTTLATATTKLQPAKGRKG

>CstyGR43a

LGMAAVSLLAISILVGLDVGTWMRIAQDMNIAQSDTELNVHWYIPFYSLYFILTGLQVNIANTAYGLGRRFGRLNRMLSSSFLAENNATSAIKPQKVSTVKNVSVNRPAMPSALHASLTKLNGETLPSEAAGDKAAARSLILNVELLKLGYFPAKNKGLLLKSLADSHESLGKCVHLLSNSFGIAVLFILVSCLLHLVATAYFLFLELLSKRDNGYLWVQMLWICFHFLRLLMVVEPCHLAARESRKTIQIVCEIERKVHEPILAEAVKKFWQQLLVVDADFSACGLCRVNRTILTSFASAIATYLVILIQFQRTNG

>CstyGR28a

MAFKLWERISQADNVFQALRPLTYISLLGLAPFRLNLNPRKEVQTSTYSFVAGIVHYLFFVLCFVTSGLEGDSIIGYFFQTNITRLGDKTLRLTGILAMSTIFGFTMFKRQRLVSIIQNYIVVDEIFVRLGMKLDYRRILLFSFLISLGMLLFNVIYLCVSYGLLVSATISPSFETFTTFALPHINISLMVFKFLCTTDLAKSRFSMLNEILQDILDAHIEQYNALELSPMHSVVNHKRYSHRLRNFISTPMKRYSVTSIIRLNPEYAIKQVSNIHNLLCDICHTIEEYFTYPLLGIIAISFLFILFDDFYILEAVLNPKRLDVFETDEFFAFFLIQMSWYIVIIILIVESSSRTILEGNQSAAIVHKILNITDDPELRDRLFRLSLQLSHRRVLFTAAGLFRLDRTLIFTITGAATCYLIILVQFRATHHMEDAVGANASQLHFLHD

>CstyGR21a

MAFWASVTTKQPPLKIPPVLNPNQKQFLQDELRFREKLDILARSSAGNLTDYYVRKHETIDDPELLDKHDSFYHTTKSLLVLFQIMGVMPIHRNPPLPNLPRTGFSWRSKQVLWAIFIYCIQTAVVVLVLRERVNNFVLNSDKRFDEAIYNIIFISLLFTNFLLPVASWRHGPQVAIFKNMWTNYQLKFLKVTGTPIVFPNLYSLTWALCICSWVLSIMINLSQYFLQPDFELWYTFAYYPIIAMLNCFCSLWYINCNAFGTASRALLTSLETTLKGDKPADKLTEYRHLWVDLSHMMQQLGRAYSNMYGMYCLVVFFTTIIATYGSFSEIIDHGATYKEVGLFVIVFYCMSLLYIICNEAHYASQKVGLDFQTQLLNVNLTAVDTATQKEVEMFLVAIAKNPPIMNLDGYANINRELITSNVSFMATYLVVLLQFKITEQRGIRSQQAMAAM

>CstyGR10a

MTSPDERKSFWERHEFKFYRYGHVYALIYGQVVIDYVPQRALKRGVKMLLIAYGHLFSMLLIVVLPGYFCYHFRTLTDTLDRRLQLLFYVSFANTAIKYATVIVTYVANTVHFEAINQRCTMQRMHLEDEFKNAPQKPKRPFEFFMYFKFCLINLMMMIQVCGIFAQYGEGGKGSVSQVRVYFAIYAFVLWNYTENMADYCYWINGSVLKYYRQFNLQLGSLRDEMDGLRPGGMLLHHCCELSDRLEELRRRCRDIHDLQRESFRMHQFQLIGLMLSTLINNLTNFYTLFHMLAKQSLEEVSYPVVVGSVYATGFYIDTYIVTLVNEHIKLELEAVALTMRRFAEPREMDERLTREIEHLSLELLNYQPPMLCGLLHLDRRLMYLIAVTAFSYFITLVQFDLYLRKKS

>DmGr2a

MEFGMDTLRALEPLHRACQVCNLWPWRLAPPPDSEGILLRRSRWLELYGWTVLIAATSFTVYGLFQESSVEEKQDSESTISSIGHTVDFIQLVGMRVAHLAALLEALWQRQAQRGFFAELGEIDRLLSKALRVDVEAMRINMRRQTSRRAVWILWGYAVSQLLILGAKLLSRGDRFPIYWISYLLPLLVCGLRYFQIFNATQLVRQRLDVLLVALQQLQLHQKGPAVDTVLEEQEDL

EEAAMDRLIAVRLVYQRVWALVALLNRCYGLSMLMQVGNDFLAITSNCYWMFLNFRQSAASPFDILQIVASGVWSAPHLGNVLVLSLLCDRTAQCASRLALCLHQVSVDLRNESHNALITQFSLQLLHQRLHFSAAGFFNVDCTLLYTIVGATTTYLIILIQFHMSESTIGSDSNGQ

>DmGr5a

MRQLKGRNRCNRAVRHLKVQGKMWLKNLKSGLEQIRESQVRGTRKNFLHDGSFHEAVAPVLAVAQCFCLMPVCGISAPTYRGLSFNRRSWRFWYSSLYLCSTSVDLAFSIRRVAHSVLDVRSVEPIVFHVSILIASWQFLNLAQLWPGLMRHWAAVERRLPGYTCCLQRARPARRLKLVAFVLLVVSLMEHLLSIISVVYYDFCPRRSDPVESYLLGASAQLFEVFPYSNWLAWLGK

IQNVLLTFGWSYMDIFLMMLGMGLSEMLARLNRSLEQQVRQPMPEAYWTWSRTLYRSIVELIREVDDAVSGIMLISFGSNLYFICLQLLKSINTMPSSAHAVYFYFSLLFLLSRSTAVLLFVSAINDQAREPLRLLRLVPLKGYHPEVFRFAAELASDQVALTGLKFFNVTRKLFLAMAGTVATYELVLIQFHEDKKTWDCSPFNLD

>DmGr8a

MSGHLGRVLQFHLRLYQVLGFHGLPLPGDGNPARTRRRLMAWSLFLLISLSALVLACLFSGEEFLYRGDMFGCANDALKYVFAELGVLAIYLETLSSQRHLANFWWLHFKLGGQKTGLVSLRSEFQQFCRYLIFLYAMMAAEVAIHLGLWQFQALTQHMLLFWSTYEPLVWLTYLRNLQFVLHLELLREQLTGLEREMGLLAEYSRFASETGRSFPGFESFLRRRLVQKQRIYSHVY

DMLKCFQGAFNFSILAVLLTINIRIAVDCYFMYYSIYNNVINNDYYLIVPALLEIPAFIYASQSCMVVVPRIAHQLHNIVTDSGCCSCPDLSLQIQNFSLQLLHQPIRIDCLGLTILDCSLLTRMACSVGTYMIYSIQFIPKFSNTYM

>DmGr9a

MSLWLEHFLTGYFQLCGLVCGWSGSRLGRLLSSTFLVLILIELVGEIETYFTEENPDNESVPAYFAKVIMGVNMAYKMIHAWIALSALFECRRFRYLLEELPPVKATSFIYRHLILEIILFACNAFLVLSEYTIRGIYLENLRYAYSLQAVRARYLQMMVLVDRLDGKLEQLHHRVISGSSDYKTLRLDYAHLAKVTRSLSHLFGLSLLLLNVLCLGDWIIVCNVYFMVAYLQVLPA

TLFLFGQVMFVVCPTLIKIWSICAASHRCVSKSKHLQQQLKDLPGQTPVERSQIEGFALQIMQDPIQIDVCGIYHLNLQTLAGMFFFILEALVIFLQFVSLVRT

>DmGr10a

MTSPDERKSFWERHEFKFYRYGHVYALIYGQVVIDYVPQRALKRGVKVLLIAYGHLFSMLLIVVLPGYFCYHFRTLTDTLDRRLQLLFYVSFTNTAIKYATVIVTYVANTVHFEAINQRCTMQRTHLEFEFKNAPQEPKRPFEFFMYFKFCLINLMMMIQVCGIFAQYGEVGKGSVSQVRVHFAIYAFVLWNYTENMADYCYFINGSVLKYYRQFNLQLGSLRDEMDGLRPGGMLLH

HCCELSDRLEELRRRCREIHDLQRESFRMHQFQLIGLMLSTLINNLTNFYTLFHMLAKQSLEEVSYPVVVGSVYATGFYIDTYIVALINEHIKLELEAVALTMRRFAEPREMDERLTREIEHLSLELLNYQPPMLCGLLHLDRRLVYLIAVTAFSYFITLVQFDLYLRKKS

>DmGr10b

MRVGKLCRLALRFWMGLILVLGFSSHYYNPTRRRLVYSRILQTYDWLLMVINLGAFYLYYRYAMTYFLEGMFRRQGFVNQVSTCNVFQQLLMAVTGTWLHFLFERHVCQTYNELSRILKHDLKLKEHSRFYCLAFLAKVYNFFHNFNFALSAIMHWGLRPFNVWDLLANLYFVYNSLARDAILVAYVLLLLNLSEALRLNGQQEHDTYSDLMKQLRRRERLLRIGRRVHRMFAWLVA

IALIYLVFFNTATIYLGYTMFIQKHDALGLRGRGLKMLLTVVSFLVILWDVVLLQVICEKLLAEENKICDCPEDVASSRTTYRQWEMSALRRAITRSSPENNVLGMFRMDMRCAFALISCSLSYGIIIIQIGYIPG

>DmGr21a

MSFWAVSRGLTPPSKVVPMLNPNQRQFLEDEVRYREKLKLMARGDAMEEVYVRKQETVDDPLELDKHDSFYQTTKSLLVLFQIMGVMPIHRNPPEKNLPRTGYSWGSKQVMWAIFIYSCQTTIVVLVLRERVKKFVTSPDKRFDEAIYNVIFISLLFTNFLLPVASWRHGPQVAIFKNMWTNYQYKFFKTTGSPIVFPNLYPLTWSLCVFSWLLSIAINLSQYFLQPDFRLWYTFAY

YPIIAMLNCFCSLWYINCNAFGTASRALSDALQTTIRGEKPAQKLTEYRHLWVDLSHMMQQLGRAYSNMYGMYCLVIFFTTIIATYGSISEIIDHGATYKEVGLFVIVFYCMGLLYIICNEAHYASRKVGLDFQTKLLNINLTAVDAATQKEVEMLLVAINKNPPIMNLDGYANINRELITTNISFMATYLVVLLQFKITEQRRIGQQQA

>DmGr22a

MSQPKRIHRICKGLARFTIRATLYGSWVLGLFPFTFDSRKRRLNRSKWLLAYGLVLNLTLLVLSMLPSTDDHNSVKVEVFQRNPLVKQVEELVEVISLITTLVTHLRTFSRSSELVEILNELLVLDKNHFSKLMLSECHTFNRYVIEKGLVIILEIGSSLVLYFGIPNSKIVVYEAVCIYIVQLEVLMVVMHFHLAVIYIYRYLWIINGQLLDMASRLRRGDSVDPDRIQLLLWLYS

RLLDLNHRLTAIYDIQVTLFMATLFSVNIIVGHVLVICWINITRFSLLVIFLLFPQALIINFWDLWQGIAFCDLAESTGKKTSMILKLFNDMENMDQETERRVAEFTLFCSHRRLKVCHLGLLDINYEMGFRMIITNILYVVFLVQFDYMNLKFKTD

>DmGr22b

MFGSSREIRPYLARQMLKTTLYGSWLLGIFPFTLDSGKRIRQLRRSRCLTLYGLVLNYFLIFTLIRLAFEYRKHKLEAFKRNPVLEMINVVIGIINVLSALIVHFMNFWGSRKVGEICNELLILEYQDFEGLNGRNCPNFNCFVIQKCLTILGQLLSFFTLNFALPGLEFHICLVLLSCLMEFSLNLNIMHYHVGVLLIYRYVWLINEQLKDLVSQLKLNPETDFSRIHQFLSLYKR

LLELNRKLVIAYEYQMTLFIIAQLSGNIVVIYFLIVYGLSMRTYSIFLVAFPNSLLINIWDFWLCIAACDLTEKAGDETAIILKIFSDLEHRDDKLEMSVNEFAWLCSHRKFRFQLCGLFSMNCRMGFKMIITTFLYLVYLVQFDYMNL

>DmGr22c

MFASRSDLQSRLCWIILKATLYSSWFLGVFPYRFDSRNGQLKRSRFLLFYGLILNFFLLLKMVCSGGQKLGIPEAFARNSVLENTHYTTGMLAVFSCVVIHFLNFWGSTRVQDLANELLVLEYQQFASLNETKCPKFNSFVIQKWLSVIGLLLSYLSIAYGLPGNNFSVEMVLINSLVQFSFNCNIMHYYIGVLLIYRYLWLINGQLLEMVTNLKLDCSVDSSRIRKYLSLYRRLLELKGYMVATYEYHMTLVLTTGLASNFLAIYSWIVLDISMNINFIYLLIFPLFLLVNVWNLWLSIAASDLAENAGKSTQTVLKLFADLEVKDIELERSVNEFALLCGHCQFNFHVCGLFTINYKMGFQMIITSFLYLIYMIQFDFMNL

>DmGr22d

MFRPRCGLRQKFVYVILKSILYSSWLLGIFPFKYEPKKRRLRRSMWLIPFGVVISSSLLILMVKQSAEDREHGIMLDVFQRNALLYQISSLMGVVGVVSICTVHLRTLWRSKHLEEIYNGLMLLEAKYFCSNAVECPAFDGYVIQKGVVIVVGLLAPWMVHFGMPDSKLPVLNVLVVSMVKLGTLLLALHYHLGVVIIYRFVWLINRELLSLVCSLRGNHKGSSSRVRFLLKLYNKLVNLYSKLADCYDCQTVLMMAIFLAANIIVCFYMIVYRISLSKMSFFVMLIMFPLAIANNFMDFWLSMKVCDLLQKTGRQTSMILKLFNDIENMDKDLEISISDFALYCSHRRFKFLHCGLFHVNREMGFKMFVASVLYLLYLVQFDYMNL

>DmGr22e

MFRPSGSGYRQKWTGLTLKGALYGSWILGVFPFAYDSWTRTLRRSKWLIAYGFVLNAAFILLVVTNDTESETPLRMEVFHRNALAEQINGIHDIQSLSMVSIMLLRSFWKSGDIERTLNELEDLQHRYFRNYSLEECISFDRFVLYKGFSVVLELVSMLVLELGMSPNYSAQFFIGLGSLCLMLLAVLLGASHFHLAVVFVYRYVWIVNRELLKLVNKMAIGETVESERMDLLLYLY

HRLLDLGQRLASIYDYQMVMVMVSFLIANVLGIYFFIIYSISLNKSLDFKILVFVQALVINMLDFWLNVEICELAERTGRQTSTILKLFNDIENIDEKLERSITDFALFCSHRRLRFHHCGLFYVNYEMGFRMAITSFLYLLFLIQFDYWNL

>DmGr22f

MKMFQPRRGFSCHLAWFMLQTTLYASWLLGLFPFTFDSRRKQLKRSRWLLLYGFVLHSLAMCLAMSSHLASKQRRKYNAFERNPLLEKIYMQFQVTTFFTISVLLLMNVWKSNTVRKIANELLTLEGQVKDLLTLKNCPNFNCFVIKKHVAAIGQFVISIYFCLCQENSYPKILKILCCLPSVGLQLIIMHFHTEIILVYRYVWLVNETLEDSHHLSSSRIHALASLYDRLLKLSEL

VVACNDLQLILMLIIYLIGNTVQIFFLIVLGVSMNKRYIYLVASPQLIINFWDFWLNIVVCDLAGKCGDQTSKVLKLFTDLEHDDEELERSLNEFAWLCTHRKFRFQLCGLFSINHNMGFQMIITSFLYLVYLLQFDFMNL

>DmGr23aA

MKTLECLTRRFLEVIFSVLALVPLPPISQLGWLFLSLAIRCCWIVYFIYLLDVAISFSWVAIENVGNAVGTMLFVGNSVLGFALLLESVLKQKTHSQLEDLRVQTELQLQRLGMFGRSRHAAYLLPLIGVQFTCDLVRLATNFGETVSPVFCISLPLMWLLRYRYVQLVQHVMDLNQRSIHLRRSLLSMASGNDLWQPYGVQECLQLQTLRTTYERIFECYETFSDCYGWGMLGLHLLTSFQFVTNAYWMIMGIYDGGNVRSLIFNGATGIDFGTPIATLFWHGDSGAENGRQIGCLISKLVKPQGSKLYNDLVSEFSLQTLHQRFVVTAKDFFSLNLHLLSSMFAAVVTYLVILIQFMFAERSSTRGSG

>DmGr23aB

MFPPTRVQASSRVVLKIFHFILVAFSLRSRRLSRLVLWLQFLGWLTWFISMWTQSVIYAQTIDCTLDCSLRHILTFFQTVSHAFIVVTSFLDGFRIKQDQLDEPIAFEDSDPWLAFTVLAMLVPTLGVEYLVCSNAPEYAFRIRIYHLKTLPSFLALQVQIISFILEVMKVNIRVRQTKLQLLILARELSCRWPQRKQKPQFSDQQAHRVKDLKRRYNDLHYLFVRINGYFGGSLLT

IIIVHFAIFVSNSYWLFVDIRTRPWRIYAILLNLGFIFNVALQMAAACWHCQQSYNLGRQIGCLISKLVKPQGSKLYNDLVSEFSLQTLHQRFVVTAKDFFSLNLHLLSSMFAAVVTYLVILIQFMFAERSSTRGSG

>DmGr28a

MAFKLWERFSQADNVFQALRPLTFISLLGLAPFRLNLNPRKEVQTSKFSFFAGIVHFLFFVLCFGISVKEGDSIIGYFFQTNITRFSDGTLRLTGILAMSTIFGFAMFKRQRLVSIIQNNIVVDEIFVRLGMKLDYRRILLSSFLISLGMLLFNVIYLCVSYSLLVSATISPSFVTFTTFALPHINISLMVFKFLCTTDLARSRFSMLNEILQDILDAHIEQLSALELSPMHSVVNHRRYSHRLRNLISTPMKRYSVTSVIRLNPEYAIKQVSNIHNLLCDICQTIEEYFTYPLLGIIAISFLFILFDDFYILEAILNPKRLDVFEADEFFAFFLMQLIWYIVIIVLIVEGSSRTILHSSYTAAIVHKILNITDDPELRDRLFRLSLQLSHRKVLFTAAGLFRLDRTLIFTITGAATCYLIILIQFRFTHHMDDTSSNSTNNLHSIHLGD

>DmGr28bA

MIRCGLDIFRGCRGRFRYWLSARDCYDSISLMVAIAFALGITPFLVRRNALGENSLEQSWYGFLNAIFRWLLLAYCYSYINLRNESLIGYFMRNHVSQISTRVHDVGGIIAAVFTFILPLLLRKYFLKSVKNMVQVDTQLERLRSPVNFNTVVGQVVLVILAVVLLDTVLLTTGLVCLAKMEVYASWQLTFIFVYELLAISITICMFCLMTRTVQRRITCLHKVLKNLAHQWDTRSL

KAVNQKQRSLQCLDSFSMYTIVTKDPAEIIQESMEIHHLICEAAATANKYFTYQLLTIISIAFLIIVFDAYYVLETLLGKSKRESKFKTVEFVTFFSCQMILYLIAIISIVEGSNRAIKKSEKTGGIVHSLLNKTKSAEVKEKLQQFSMQLMHLKINFTAAGLFNIDRTLYFTISGALTTYLIILLQFTSNSPNNGYGNGSSCCETFNNMTNHTL

>DmGr28bB

MSALRRVRKYFISSQVYEALRPLFFLTFLYGLTPFHVVRRKMGESYLKMSCFGVFNIFIYICLCGFCYISSLRQGESIVGYFFRTEISTIGDRLQIFNGLIAGAVIYTSAILKRCKLLGTLTILHSLDTNFSNIGVRVKYSRIFRYSLLVLIFKLLILGVYFVGVFRLLVSLDVTPSFCVCMTFFLQHSVVSIAICLFCVIAFSFERRLSIINQVLKNLAHQWDTRSLKAVNQKQRSLQCLDSFSMYTIVTKDPAEIIQESMEIHHLICEAAATANKYFTYQLLTIISIAFLIIVFDAYYVLETLLGKSKRESKFKTVEFVTFFSCQMILYLIAIISIVEGSNRAIKKSEKTGGIVHSLLNKTKSAEVKEKLQQFSMQLMHLKINFTAAGLFNIDRTLYFTISGALTTYLIILLQFTSNSPNNGYGNGSSCCETFNNMTNHTL

>DmGr28bC

MDIEMAKEPVNPTDTPDIEVTPGLCQPLRRRFRRFVTAKQLYECLRPVFHVTYIHGLTSFYISCDTKTGKKAIKKTIFGYINGIMHIAMFVFAYSLTIYNNCESVASYFFRSRITYFGDLMQIVSGFIGVTVIYLTAFVPNHRLERCLQKFHTMDVQLQTVGVKIMYSKVLRFSYMVLISMFLVNVLFTGGTFSVLYSSEVAPTMALHFTFLIQHTVIAIAIALFSCFTYLVEMRLV

MVNKVLKNLAHQWDTRSLKAVNQKQRSLQCLDSFSMYTIVTKDPAEIIQESMEIHHLICEAAATANKYFTYQLLTIISIAFLIIVFDAYYVLETLLGKSKRESKFKTVEFVTFFSCQMILYLIAIISIVEGSNRAIKKSEKTGGIVHSLLNKTKSAEVKEKLQQFSMQLMHLKINFTAAGLFNIDRTLYFTISGALTTYLIILLQFTSNSPNNGYGNGSSCCETFNNMTNHTL

>DmGr28bD

MSFYFCEIFKPRDAFGAEQTLLLYTYLLGLTPFRLRGQAGERQFHLSKIGYLNAFLQLSFFSYCFLAALIEQQSIVGYFFKSEISQMGDSLQKFIGMTGMSILFLCSSIRVRLLIHIWDRISYIDDRFLNLGVCFNYPAIMRLRLLQIFLINGVQLGYLISSNWMLLGNDVRPIYTAIVAFYVPQIFLLSIVMLFNATLHRLWQHFTVLNQVLKNLAHQWDTRSLKAVNQKQRSLQC

LDSFSMYTIVTKDPAEIIQESMEIHHLICEAAATANKYFTYQLLTIISIAFLIIVFDAYYVLETLLGKSKRESKFKTVEFVTFFSCQMILYLIAIISIVEGSNRAIKKSEKTGGIVHSLLNKTKSAEVKEKLQQFSMQLMHLKINFTAAGLFNIDRTLYFTISGALTTYLIILLQFTSNSPNNGYGNGSSCCETFNNMTNHTL

>DmGr28bE

MWLLRRSVGKSGNRPHDVYTCYRLTIFMALCLGIVPYYVSISSEGRGKLTSSYIGYINIIIRMAIYMVNSFYGAVNRDTLMSNFFLTDISNVIDALQKINGMLGIFAILLISLLNRKELLKLLATFDRLETEAFPRVGVAMHQVAANKKMNRLVIILVGSMVAYITCSFLMISLRDTTTFSISAVISFFSPHFIVCAVSFLAGNVMIKLRIYLSALNEVLKNLAHQWDTRSLKAVNQ

KQRSLQCLDSFSMYTIVTKDPAEIIQESMEIHHLICEAAATANKYFTYQLLTIISIAFLIIVFDAYYVLETLLGKSKRESKFKTVEFVTFFSCQMILYLIAIISIVEGSNRAIKKSEKTGGIVHSLLNKTKSAEVKEKLQQFSMQLMHLKINFTAAGLFNIDRTLYFTISGALTTYLIILLQFTSNSPNNGYGNGSSCCETFNNMTNHTL

>DmGr32a

MSPNTWVIEMPTQKTRSHPYPRRISPYRPPVLNRDAFSRDAPPMPARNHDHPVFEDIRTILSVLKASGLMPIYEQVSDYEVGPPTKTNEFYSFFVRGVVHALTIFNVYSLFTPISAQLFFSYRETDNVNQWIELLLCILTYTLTVFVCAHNTTSMLRIMNEILQLDEEVRRQFGANLSQNFGFLVKFLVGITACQAYIIVLKIYAVQGEITPTSYILLAFYGIQNGLTATYIVFASA

LLRIVYIRFHFINQLLNGYTYGQQHRRKEGGARARRQRGDVNPNVNPALMEHFPEDSLFIYRMHNKLLRIYKGINDCCNLILVSFLGYSFYTVTTNCYNLFVQITGKGMVSPNILQWCFAWLCLHVSLLALLSRSCGLTTTEANATSQILARVYAKSKEYQNIIDKFLTKSIKQEVQFTAYGFFAIDNSTLFKIFSAVTTYLVILIQFKQLEDSKVEDPVPEQT

>DmGr33a

MIQIMNWFSMVIGLIPLNRQQSETNFILDYAMMCIVPIFYVACYLLINLSHIIGLCLLDSCNSVCKLSSHLFMHLGAFLYLTITLLSLYRRKEFFQQFDARLNDIDAVIQKCQRVAEMDKVKVTAVKHSVAYHFTWLFLFCVFTFALYYDVRSLYLTFGNLAFIPFMVSSFPYLAGSIIQGEFIYHVSVISQRFEQINMLLEKINQEARHRHAPLTVFDIESEGKKERKTVTPITVM

DGRTTTGFGNENKFAGEMKRQEGQQKNDDDDLDTSNDEDEDDFDYDNATIAENTGNTSEANLPDLFKLHDKILALSVITNGEFGPQCVPYMAACFVVSIFGIFLETKVNFIVGGKSRLLDYMTYLYVIWSFTTMMVAYIVLRLCCNANNHSKQSAMIVHEIMQKKPAFMLSNDLFYNKMKSFTLQFLHWEGFFQFNGVGLFALDYTFIFSTVSAATSYLIVLLQFDMTAILRNEGLMS

>DmGr36a

MFDWVGLLLKVLYYYGQIIGLINFEIDWQRGRVVAAQRGILFAIAINVLICMVLLLQISKKFNLDVYFGRANQLHQYVIIVMVSLRMASGISAILNRWRQRAQLMRLVECVLRLFLKKPHVKQMSRWAILVKFSVGVVSNFLQMAISMESLDRLGFNEFVGMASDFWMSAIINMAISQHYLVILFVRAYYHLLKTEVRQAIHESQMLSEIYPRRAAFMTKCCYLADRIDNIAKLQNQ

LQSIVTQLNQVFGIQGIMVYGGYYIFSVATTYITYSLAINGIEELHLSVRAAALVFSWFLFYYTSAILNLFVMLKLFDDHKEMERILEERTLFTSALDVRLEQSFESIQLQLIRNPLKIEVLDIFTITRSSSAAMIGSIITNSIFLIQYDMEYF

>DmGr36b

MVDWVVLLLKAVHIYCYLIGLSNFEFDCRTGRVFKSRRCTIYAFMANIFILITIIYNFTAHGDTNLLFQSANKLHEYVIIIMSGLKIVAGLITVLNRWLQRGQMMQLVKDVIRLYMINPQLKSMIRWGILLKAFISFAIELLQVTLSVDALDRQGTAEMMGLLVKLCVSFIMNLAISQHFLVILLIRAQYRIMNAKLRMVIEESRRLSFLQLRNGAFMTRCCYLSDQLEDIGEVQSQ

LQSMVGQLDEVFGMQGLMAYSEYYLSIVGTSYMSYSIYKYGPHNLKLSAKTSIIVCILITLFYLDALVNCNNMLRVLDHHKDFLGLLEERTVFASSLDIRLEESFESLQLQLARNPLKINVMGMFPITRGSTAAMCASVIVNSIFLIQFDMEFF

>DmGr36c

MDLESFLLGAVYYYGLFIGLSNFEFDWNTGRVFTKKWSTLYAIALDSCIFALYIYHWTGNTNIVNAIFGRANMLHEYVVAILTGLRIVTGLFTLILRWYQRCKMMDLASKVVRMYVARPQVRRMSRWGILTKFIFGSITDGLQMAMVLSAMGSVDSQFYLGLGLQYWMFVILNMAMMQQHMIMLFVRTQFQLINTELRQVIDEAKDLLLSPRHQGVFMTKCCSLADQIENIARIQSQ

LQTIMNQMEEVFGIQGAMTYGGYYLSSVGTCYLAYSILKHGYENLSMTLSTVILAYSWCFFYYLDGMLNLSVMLHVQDDYWEMLQILGKRTIFVGLDVRLEEAFENLNLQLIRNPLKITVVKLYDVTRSNTMAMFGNLITHSIFLIQYDIEHF

>DmGr39aA

MGTRNRKLLFFLHYQRYLGLTNLDFSKSLHIYWLHGTWSSTAIQIVVVGVFMAALLGALAESLYYMETKSQTGNTFDNAVILTTSVTQLLANLWLRSQQKSQVNLLQRLSQVVELLQFEPYAVPQFRWLYRIWLLVCLIYGAMVTHFGINWLTTMQISRVLTLIGFVYRCVLANFQFTCYTGMVVILKKLLQVQVKQLEHLVSTTTISMAGVAGCLRTHDEILLLGQRELIAVYGGV

ILFLFIYQVMQCILIFYISNLEGFHSSNDLVLIFCWLAPMLFYLILPLVVNDIHNQANKTAKMLTKVPRTGTGLDRMIEKFLLKNLRQKPILTAYGFFALDKSTLFKLFTAIFTYMVILVQFKEMENSTKSINKF

>DmGr39aB

MDFQPGELCAYYRLCRYLGIFCIDYNPTKKKFRLRRSVLCYIVHFALQAYLVGCISVMVTYWRRCFKSELTTTGNHFDRLVMVIALGILVVQNAWLIWLQAPHLRIVRQIEFYRRNHLANVRLLLPKRLLWLIIATNVVYMANFIKTCIFEWLTDASRLFVITSLGFPLRYLVTSFTMGTYFCMVHIVRLVLDWNQSQINAIIDESADLKMTSPNRLRLRVCLEMHDRLMLLCNDEI

SLVYGFIAWLSWMFASLDVTGVIYLTMVIQTKKSIVLKLITNVVWLSPTFMTCAASFMSNRVTIQANKTAKMLTKVPRTGTGLDRMIEKFLLKNLRQKPILTAYGFFALDKSTLFKLFTAIFTYMVILVQFKEMENSTKSINKF

>DmGr39aC

MKRNAFEELRVQLRTLKWLGVLRFTIDFNKCLVRENASEERSAWLYLIGVVGITCSLIVYSTYFPSHFIMGKHNTTGNCYALINIRSCSIVTMLIYTQLYIQRFRFVALLQSILRFNQISGSHREEGRFAFYYYTHLSLLIICMLNYAYGYWTAGVRLTTIPIYLLQYGFSYLFLGQVVVLFACIQQILLSILKYYNQVVLKNIKSSKESREFYYNFCKYNQVIWLSYTEINHCFGL

LLLLVTGLILLITPSGPFYLVSTIFEGRFRQNWQFSLMSFTAILWSLPWIVLLVLAMGRNDVQKEANKTAKMLTKVPRTGTGLDRMIEKFLLKNLRQKPILTAYGFFALDKSTLFKLFTAIFTYMVILVQFKEMENSTKSINKF

>DmGr39aD

MSKVCRDLRIYLRLLHIMGMMCWHFDSDHCQLVATSGSERYAVVYAGCILVSTTAGFIFALLHPSRFHIAIYNQTGNFYEAVIFRSTCVVLFLVYVILYAWRHRYRDLVQHILRLNRRCASSCTNQQFLHNIILYGMLTILCFGNYLHGYTRAGLATLPLALCMLVYIFAFLVLCLLLMFFVSLKQVMTAGLIHYNQQLCQGDLISGLRGRQQILKLCGGELNECFGLLMLPIVALV

LLMAPSGPFFLISTVLEGKFRPDECLIMLLTSSTWDTPWMIMLVLMLRTNGISEEANKTAKMLTKVPRTGTGLDRMIEKFLLKNLRQKPILTAYGFFALDKSTLFKLFTAIFTYMVILVQFKEMENSTKSINKF

>DmGr39b

MLYSFHPYLKYFALLGLVPWSESCAQSKFVQKVYSAILIILNAVHFGISIYFPQSAELFLSLMVNVIVFVARIVCVTVIILQVMVHYDDYFRFCREMKYLGLRLQCELKIHVGRLKWQSYAKILALGIGFLVTVLPSIYVALSGSLLYFWSSLLSILIIRMQFVLVLLNVELLGHHVSLLGIRLQNVLECHLMGANCTLDGNANRLCSLEFLLALKQSHMQLHYLFTHFNDLFGWSI

LGTYVVLFSDSTVNIYWTQQVLVEVYEYKYLYATFSVFVPSFFNILVFCRCGEFCQRQSVLIGSYLRNLSCHPSIGRETSYKDLLMEFILQVEQNVLAINAEGFMSTDNSLLMSILAAKVTYLIVLMQFSSV

>DmGr43a

MEISQPSIGIFYISKVLALAPYATVRNSKGRVEIGRSWLFTVYSATLTVVMVFLTYRGLLFDANSEIPVRMKSATSKVVTALDVSVVVMAIVSGVYCGLFSLNDTLELNDRLNKIDNTLNAYNNFRRDRWRALGMAAVSLLAISILVGLDVGTWMRIAQDMNIAQSDTELNVHWYIPFYSLYFILTGLQVNIANTAYGLGRRFGRLNRMLSSSFLAENNATSAIKPQKVSTVKNVSV

NRPAMPSALHASLTKLNGETLPSEAAAKNKGLLLKSLADSHESLGKCVHLLSNSFGIAVLFILVSCLLHLVATAYFLFLELLSKRDNGYLWVQMLWICFHFLRLLMVVEPCHLAARESRKTIQIVCEIERKVHEPILAEAVKKFWQQLLVVDADFSACGLCRVNRTILTSFASAIATYLVILIQFQRTNG

>DmGr47a

MAFTSSQLCSLLTKFTALNGLNTYYFDTKTNAFRVSSKLKIYCAIHHALCVLALAHMSYSTASNLRVSVTVLTIGGTMACCVKSCWEKAQGIRNLARGLVTMEQKYFAGRPSGLLLKCRYYIKITFGSITLLRIHLIQPIYMRRLLPSQFYLNVGAYWLLYNMLLAAVLGFYFLLWEMCRIQKLINDQMTLILARSGQRNRLKKMQHCLRLYSKLLLLCDQFNSQLGHVAIWVLACK

SWCQITFGYEIFQMVAAPKSIDLTMSMRVFVIFTYIFDAMNLFLGTDISELFSTFRADSQRILRETSRLDRLLSMFALKLALHPKRVVLLNVFTFDRKLTLTLLAKSTLYTICCLQNDYNKLKA

>DmGr47b

MQRDDGFVYCYGNLYSLLLYWGLVTIRVRSPDRGGAFSNRWTVCYALFTRSFMVICFMATVMTKLRDPEMSAAMFGHLSPLVKAIFTWECLSCSVTYIEYCLSLDLQKDRHLKLVARMQEFDRSVLMVFPHVQWNYRRARLKYWYGTVIVGFCFFSFSISLIFDTTRCTCGIPSTLLMAFTYTLLTSSVGLLGFVHIGIMDFIRVRLRLVQQLLHQLYQADDSSEVHERIAYLFEMS

KRCSFLLAELNGVFGFAAAAGIFYDFTIMTCFVYVICQKLLEREPWDPEYVYMLLHVAIHTYKVVITSTYGYLLLREKRNCMHLLSQYSRYFSGQDVARRKTEDFQHWRMHNRQAAMVGSTTLLSVSTIYLVYNGMANYVIILVQLLFQQQQIKDHQLTSGKDVDIVGPMGPITHMD

>DmGr57a

MAVLYFFREPETVFDCAAFICILQFLMGCNGFGIRRSTFRISWASRIYSMSVAIAAFCCLFGSLSVLLAEEDIRERLAKADNLVLSISALELLMSTLVFGVTVISLQVFARRHLGIYQRLAALDARLMSDFGANLNYRKMLRKNIAVLGIVTTIYLMAINSAAVQVASGHRALFLLFALCYTIVTGGPHFTGYVHMTLAEMLGIRFRLLQQLLQPEFLNWRFPQLHVQELRIRQVVS

MIQELHYLIQEINRVYALSLWAAMAHDLAMSTSELYILFGQSVGIGQQNEEENGSCYRMLGYLALVMIPPLYKLLIAPFYCDRTIYEARRCLRLVEKLDDWFPQKSSLRPLVESLMSWRIQAKIQFTSGLDVVLSRKVIGLFTSILVNYLLILIQFAMTQKMGEQIEQQKIALQEWIGF

>DmGr58a

MLLKFMYIYGIGCGLMPAPLKKGQFLLGYKQRWYLIYTACLHGGLLTVLPFTFPHYMYDDSYMSSNPVLKWTFNLTNITRIMAMFSGVLLMWFRRKRILNLGENLILHCLKCKTLDNRSKKYSKLRKRVRNVLFQMLLVANLSILLGALILFRIHSVQRISKTAMIVAHITQFIYVVFMMTGICVILLVLHWQSERLQIALKDLCSFLNHEERNSLTLSENKANRSLGKLAKLFKLF

AENQRLVREVFRTFDLPIALLLLKMFVTNVNLVYHGVQFGNDTIETSSYTRIVGQWVVISHYWSAVLLMNVVDDVTRRSDLKMGDLLREFSHLELVKRDFHLQLELFSDHLRCHPSTYKVCGLFIFNKQTSLAYFFYVLVQVLVLVQFDLKNKVEKRN

>DmGr58b

MLHPKLGRVMNVVYYHSVVFALMSTTLRIRSCRKCLRLEKVSRTYTIYSFFVGIFLFLNLYFMVPRIMEDGYMKYNIVLQWNFFVMLFLRAIAVVSCYGTLWLKRHKIIQLYKYSLIYWKRFGHITRAIVDKKELLDLQESLARIMIRKIILLYSAFLCSTVLQYQLLSVINPQIFLAFCARLTHFLHFLCVKMGFFGVLVLLNHQFLVIHLAINALHGRKARKKWKALRSVAAMHL

KTLRLARRIFDMFDIANATVFINMFMTAINILYHAVQYSNSSIKSNGWGILFGNGLIVFNFWGTMALMEMLDSVVTSCNNTGQQLRQLSDLPKVGPKMQRELDVFTMQLRQNRLVYKICGIVELDKPACLSYIGSILSNVIILMQFDLRRQRQPINDRQYLIHLMKNKTKV

>DmGr58c

MNQYFLLHTYFQVSRLIGLCNLHYDSSNHRFILNHVPTVVYCVILNVVYLLVLPFALFVLTGNIYHCPDAGMFGVVYNVVALTKLLTMLFLMSSVWIQRRRLYKLGNDLMKMLHKFRFNLGNDCRNRCLCKGLLTSSRFVLLTQQLLTRDSVVNCESNSSLRQAMVPYQSAAIVYALIMILLMSYVDMTVYMVEVAGNWLLVNMTQGVREMVQDLEVLPERNGIPREMGLMQILAAW

RKLWRRCRRLDALLKQFVDIFQWQVLFNLLTTYIFSIAVLFRLWIYLEFDKNFHLWKGILYAIIFLTHHVEIVMQFSIFEINRCKWLGLLEDVGNLWDINYSGRQCIKSSGTILSRKLEFSLLYMNRKLQLNPKRVRRLHIVGLFDLSNLTVHNMTRSIITNVLVLCQIAYKKYG

>DmGr59a

MKRIGQAYNVYAVFIGMTSYETMGGKFRQSRITRIYCLLINAIFLTLLPSAFWKSAKLLSTADWMPSYMRVTPYIMCTINYAAIAYTLISRCYRDAMLMDLQRIVLEVNREMLRTGKKMNSLLRRMFFLKTFTLTYSCLSYILAVFIYQWKAQNWSNLCNGLLVNISLTILFVNTFFYFTSLWHIARGYDFVNQQLNEIVACQSMDLERKSKELRGLWALHRNLSYTARRINKHYGP

QMLAMRFDYFIFSIINACIGTIYSTTDQEPSLEKIFGSLIYWVRSFDFFLNDYICDLVSEYQMQPKFFAPESSMSNELSSYLIYESSTRLDLLVCGLYRVNKRKWLQMVGSIVVHSSMLFQFHLVMRGGL

>DmGr59b

MVYWMIKLYFRYSLAIGITSQQFSNRKFFSTLFSRTYALIANIVTLIMLPIVMWQVQLVFQQKKTFPKLILITNNVREAVSFLVILYTVLSRGFRDTAFKEMQPLLLTLFREEKRCGFKGIGGVRRSLRILLFVKFFTLSWLCVTDVLFLLYSTDALIWVNVLRFFFKCNTNNILEMVPMGYFLALWHIARGFDCVNRRLDQIVKSKSTRKHRELQHLWLLHACLTKTALNINKIYA

PQMLASRFDNFVNGVIQAYWGAVFTFDLSTPFFWVVYGSVQYHVRCLDYYLIDNMCDVAVEYHDSAKHSWSEVRWTKEISSYVIYANSTKLQLWSCGLFQANRSMWFAMISSVLYYILVLLQFHLVMRK

>DmGr59c

MVDLVKTILLIAYWYGLAVGVSNFEVDWLTGEAIATRRTTIYAAVHNASLITLLILFNLGNNSLKSEFISARYLHEYFFMLMTAVRISAVLLSLITRWYQRSRFIRIWNQILALVRDRPQVVRGRWYRRSIILKFVFCVLSDSLHTISDVSAQRKRITADLIVKLSLLATLTTIFNMIVCQYYLAMVQVIGLYKILLQDLRCLVRQAECICSIRNRRGGVYSIQCCSLADQLDLIAE

RHYFLKDRLDEMSDLFQIQSLSMSLVYFFSTMGSIYFSVCSILYSSTGFGSTYWGLLLIVLSTASFYMDNWLSVNIGFHIRDQQDELFRVLADRTLFYRELDNRLEAAFENFQLQLASNRHEFYVMGLFKMERGRLIAMLSSVITHTMVLVQWEIQNDES

>DmGr59d

MADLLKLCLRIAYAYGRLTGVINFKIDLKTGQALVTRGATLISVSTHLLIFALLLYQTMRKSVVNVMWKYANSLHEYVFLVIAGFRVVCVFLELVSRWSQRRTFVRLFNSFRRLYQRNPDIIQYCRRSIVSKFFCVTMTETLHIIVTLAMMRNRLSIALALRIWAVLSLTAIINVIITQYYVATACVRGRYALLNKDLQAIVTESQSLVPNGGGVFVTKCCYLADRLERIAKSQSDL

QELVENLSTAYEGEVVCLVITYYLNMLGTSYLLFSISKYGNFGNNLLVIITLCGIVYFVFYVVDCWINAFNVFYLLDAHDKMVKLLNKRTLFQPGLDHRLEMVFENFALNLVRNPLKLHMYGLFEFGRGTSFAVFNSLLTHSLLLIQYDVQNF

>DmGr59e

MDSSYWENLLLTINRFLGVYPSGRVGVLRWLHTLWSLFLLMYIWTGSIVKCLEFTVEIPTIEKLLYLMEFPGNMATIAILVYYAVLNRPLAHGAELQIERIITGLKGKAKRLVYKRHGQRTLHLMATTLVFHGLCVLVDVVNYDFEFWTTWSSNSVYNLPGLMMSLGVLQYAQPVHFLWLVMDQMRMCLKELKLLQRPPQGSTKLDACYESAFAVLVDAGGGSALMIEEMRYTCNLI

EQVHSQFLLRFGLYLVLNLLNSLVSICVELYLIFNFFETPLWEESVLLVYRLLWLAMHGGRIWFILSVNEQILEQKCNLCQLLNELEVCSSRLQRTINRFLLQLQRSIDQPLEACGIVTLDTRSLGGFIGVLMAIVIFLIQIGLGNKSLMGVALNRSNWVYV

>DmGr59f

MRSSATKGAKLKNSPRERLSSFNPQYAERYKELYRTLFWLLLISVLANTAPITILPGCPNRFYRLVHLSWMILWYGLFVLGSYWEFVLVTTQRVSLDRYLNAIESAIYVVHIFSIMLLTWQCRNWAPKLMTNIVTSDLNRAYTIDCNRTKRFIRLQLFLVGIFACLAIFFNIWTHKFVVYRSILSINSYVMPNIISSISFAQYYLLLQGIAWRQRRLTEGLERELTHLHSPRISEVQ

KIRMHHANLIDFTKAVNRTFQYSILLLFVGCFLNFNLVLFLVYQGIENPSMADFTKWVCMLLWLAMHVGKVCSILHFNQSIQNEHSTCLTLLSRVSYARKDIQDTITHFIIQMRTNVRQHVVCGVINLDLKFLTTLLVASADFFIFLLQYDVTYEALSKSVQGNVTRYK

>DmGr61a

MSRTSDDIRKHLKVRRQKQRAILAMRWRCAQGGLEFEQLDTFYGAIRPYLCVAQFFGIMPLSNIRSRDPQDVKFKVRSIGLAVTGLFLLLGGMKTLVGANILFTEGLNAKNIVGLVFLIVGMVNWLNFVGFARSWSHIMLPWSSVDILMLFPPYKRGKRSLRSKVNVLALSVVVLAVGDHMLYYASGYCSYSMHILQCHTNHSRITFGLYLEKEFSDIMFIMPFNIFSMCYGFWLNG

AFTFLWNFMDIFIVMTSIGLAQRFQQFAARVGALEGRHVPEALWYDIRRDHIRLCELASLVEASMSNIVFVSCANNVYVICNQALAIFTKLRHPINYVYFWYSLIFLLARTSLVFMTASKIHDASLLPLRSLYLVPSDGWTQEVQRFADQLTSEFVGLSGYRLFCLTRKSLFGMLATLVTYELMLLQIDAKSHKGLRCA

>DmGr63a

MRPSGEKVVKGHGQGNSGHSLSGMANYYRRKKGDAVFLNAKPLNSANAQAYLYGVRKYSIGLAERLDADYEAPPLDRKKSSDSTASNNPEFKPSVFYRNIDPINWFLRIIGVLPIVRHGPARAKFEMNSASFIYSVVFFVLLACYVGYVANNRIHIVRSLSGPFEEAVIAYLFLVNILPIMIIPILWYEARKIAKLFNDWDDFEVLYYQISGHSLPLKLRQKAVYIAIVLPILSVLS

VVITHVTMSDLNINQVVPYCILDNLTAMLGAWWFLICEAMSITAHLLAERFQKALKHIGPAAMVADYRVLWLRLSKLTRDTGNALCYTFVFMSLYLFFIITLSIYGLMSQLSEGFGIKDIGLTITALWNIGLLFYICDEAHYASVNVRTNFQKKLLMVELNWMNSDAQTEINMFLRATEMNPSTINCGGFFDVNRTLFKGLLTTMVTYLVVLLQFQISIPTDKGDSEGANNITVVDFVMDSLDNDMSLMGASTLSTTTVGTTLPPPIMKLKGRKG

>DmGr64a

MKGPNLNFRKTPSKDNGVKQVESLARPETPPPKFVEDSNLEFNVLASEKLPNYTNLDLFHRAVFPFMFLAQCVAIMPLVGIRESNPRRVRFAYKSIPMFVTLIFMIATSILFLSMFTHLLKIGITAKNFVGLVFFGCVLSAYVVFIRLAKKWPAVVRIWTRTEIPFTKPPYEIPKRNLSRRVQLAALAIIGLSLGEHALYQVSAILSYTRRIQMCANITTVPSFNNYMQTNYDYVFQ

LLPYSPIIAVLILLINGACTFVWNYMDLFIMMISKGLSYRFEQITTRIRKLEHEEVCESVFIQIREHYVKMCELLEFVDSAMSSLILLSCVNNLYFVCYQLLNVFNKLRWPINYIYFWYSLLYLIGRTAFVFLTAADINEESKRGLGVLRRVSSRSWCVEVERLIFQMTTQTVALSGKKFYFLTRRLLFGMAGTIVTYELVLLQFDEPNRRKGLQPLCA

>DmGr64b

MPQGETFHRAVSNVLFISQIYGLLPVSNVRALDVADIRFRWCSPRILYSLLIGILNLSEFGAVINYVIKVTINFHTSSTLSLYIVCLLEHLFFWRLAIQWPRIMRTWHGVEQLFLRVPYRFYGEYRIKRRIYIVFTIVMSSALVEHCLLLGNSFHLSNMERTQCKINVTYFESIYKWERPHLYMILPYHFWMLPILEWVNQTIAYPRSFTDCFIMCIGIGLAARFHQLYRRIAAVHR

KVMPAVFWTEVREHYLALKRLVHLLDAAIAPLVLLAFGNNMSFICFQLFNSFKNIGVDFLVMLAFWYSLGFAVVRTLLTIFVASSINDYERKIVTALRDVPSRAWSIEVQRFSEQLGNDTTALSGSGFFYLTRSLVLAMGTTIITYELMISDVINQGSIRQKTQYCREY

>DmGr64c

MQQSGQKGTRNTLQHAIGPVLVIAQFFGVLPVAGVWPSCRPERVRFRWISLSLLAALILFVFSIVDCALSSKVVFDHGLKIYTIGSLSFSVICIFCFGVFLLLSRRWPYIIRRTAECEQIFLEPEYDCSYGRGYSSRLRLWGVCMLVAALCEHSTYVGSALYNNHLAIVECKLDANFWQNYFQRERQQLFLIMHFTAWWIPFIEWTTLSMTFVWNFVDIFLILICRGMQMRFQQMHW

RIRQHVRQQMPNEFWQRIRCDLLDLSDLLGIYDKELSGLIVLSCAHNMYFVCVQIYHSFQSKGNYADELYFWFCLSYVIIRVLNMMFAASSIPQEAKEISYTLYEIPTEFWCVELRRLNEIFLSDHFALSGKGYFLLTRRLIFAMAATLMVYELVLINQMAGSEVQKSFCEGGVGSSKSIFS

>DmGr64d

MLRSHLSVHGLQMERSVQENTLHYTIGHVLIIARIFGVLPLAGINPNGKPENVRFRWFSPYILFFVVAFTFVIADFMLSTKIVLNDGLQLYTMGSLSFSVICIFCFGSFIKLSRRWPHIIRETALCERIFLKPCYANQEGLNFTRFLRRWALILLVAALCEHLTYVGSAAWSNYVQIRDCNLKVGFVENYFLRERQELFSVFEYRAWMVFFIEWNTMAMTFVWNFGDIFLFLMCRGL

KIRFQQLHWRIRQNLGKPMAKEFWQEIRSDFLDLDSLLKLYDKELSGLILVCCAHNMYFICVQVYHSFQVKGAFMDELYFWFCLLYVISRLMNMMLAASSIPQEIKDISNTLYEVRSSPWCDELGRLSEMLRNETFALSGMGYFYVTRRLIFAMAGALMGYELVLFRQMQGAVVQKSICSRGPGSSMSIFFS

>DmGr64e

MARTTGDPAKRRRCMSRIKFWRRSRVGSEATLGIIKYRVVEKDTKRFKLSLIKAWLLRIRQEDYKYSGSFQEAIKPVLIIAQIFALMPVRKVSSKFAEDLTFTWFSVRSYYALVTILFFGVSSGYMVAFVTSVSFNFDSVETLVFYLSIFLISLSFFQLARKWPEIAQSWQLVEAKLPPLKLPKERRSLAQHINMITIVATTCSLVEHIMSMLSMGYYVNSCPRWPDRPIDSFLYLS

FSSVFYFVDYTRFLGIVGKVVNVLSTFAWNFNDIFVMAVSVALAARFRQLNDYMMREARLPTTVDYWMQCRINFRNLCKLCEEVDDAISTITLLCFSNNLYFICGKILKSMQAKPSIWHALYFWFSLVYLLGRTLILSLYSSSINDESKRPLVIFRLVPREYWCDELKRFSEEVQMDNVALTGMKFFRLTRGVVISVAGTIVTYELILLQFNGEEKVPGCFEN

>DmGr64f

MKILPKLERKLRRLKKRVTRTSLFRKLDLVHERARKKAFQESCETYKNQIENEYEIRNSLPKLSRSDKEAFLSDGSFHQAVGRVLLVAEFFAMMPVKGVTGKHPSDLSFSWRNIRTCFSLLFIASSLANFGLSLFKVLNNPISFNSIKPIIFRGSVLLVLIVALNLARQWPQLMMYWHTVEKDLPQYKTQLTKWKMGHTISMVMLLGMMLSFAEHILSMVSAINYASFCNRTADPIQ

NYFLRTNDEIFFVTSYSTTLALWGKFQNVFSTFIWNYMDLFVMIVSIGLASKFRQLNDDLRNFKGMNMAPSYWSERRIQYRNICILCDKMDDAISLITMVSFSNNLYFICVQLLRSLNTMPSVAHAVYFYFSLIFLIGRTLAVSLYSSSVHDESRLTLRYLRCVPKESWCPEVKRFTEEVISDEVALTGMKFFHLTRKLVLSVAGTIVTYELVLIQFHEDNDLWDCDQSYYS

>DmGr66a

MDNMAQAEDAVQPLLQQFQQLFFISKIAGILPQDLEKFRSRNLLEKSRNGMIYMLSTLILYVVLYNILIYSFGEEDRSLKASQSTLTFVIGLFLTYIGLIMMVSDQLTALRNQGRIGELYERIRLVDERLYKEGCVMDNSTIGRRIRIMLIMTVIFELSILVSTYVKLVDYSQWMSLLWIVSAIPTFINTLDKIWFAVSLYALKERFEAINATLEELVDTHEKHKLWLRGNQEVPPP

LDSSQPPQYDSNLEYLYKELGGMDIGSIGKSSVSGSGKNKVAPVAHSMNSFGEAIDAASRKPPPPPLATNMVHESELGNAAKVEEKLNNLCQVHDEICEIGKALNELWSYPILSLMAYGFLIFTAQLYFLYCATQYQSIPSLFRSAKNPFITVIVLSYTSGKCVYLIYLSWKTSQASKRTGISLHKCGVVADDNLLYEIVNHLSLKLLNHSVDFSACGFFTLDMETLYGVSGGITSYLIILIQFNLAAQQAKEAIQTFNSLNDTAGLVGAATDMDNISSTLRDFVTTTMTPAV

>DmGr68a

MKIYQDIYPISKPSQIFAILPFYSGDVDDGFRFGGLGRWYGRLVALIILIGSLTLGEDVLFASKEYRLVASAQGDTEEINRTIETLLCIISYTMVVLSSVQNASRHFRTLHDIAKIDEYLLANGFRETYSCRNLTILVTSAAGGVLAVAFYYIHYRSGIGAKRQIILLLIYFLQLLYSTLLALYLRTLMMNLAQRIGFLNQKLDTFNLQDCGHMENWRELSNLIEVLCKFRYITENI

NCVAGVSLLFYFGFSFYTVTNQSYLAFATLTAGSLSSKTEVADTIGLSCIWVLAETITMIVICSACDGLASEVNGTAQILARIYGKSKQFQNLIDKFLTKSIKQDLQFTAYGFFSIDNSTLFKIFSAVTTYLVILIQFKQLEDSKVEDISQA

>DmGr77a

MPLPLGDPLALAVSPQLGYIRITAMPRWLQLPGMSALGILYSLTRVFGLMATANWSPRGIKRVRQSLYLRIHGCVMLIFVGCFSPFAFWCIFQRMAFLRQNRILLMIGFNRYVLLLVCAFMTLWIHCFKQAEIIGCLNRLLKCRRRLRRLMHTRKLKDSMDCLATKGHLLEVVVLLSSYLLSMAQPIQILKDDPEVRRNFMYACSLVFVSVCQAILQLSLGMYTMAILFLGHLVRHS

NLLLAKILADAEHIFESSQKAGFWPNRQELYKGQQKWLALELWRLLHVHHQLLKLHRSICSLCAVQAVCFLGFVPLECTIHLFFTYFMKYSKFILRKYGRSFPLNYFAIAFLVGLFTNLLLVILPTYYSERRFNCTREIIKGGGLAFPSRITVKQLRHTMHFYGLYLKNVEHVFAVSACGLFKLNNAILFCIVGAILEYLMILIQFDKVLNK

>DmGr85a

MYSLIEAQLLGGKLVNRVMASLRRIIQRSLGYFCALNGILDFNTDIGTGNLRRYRVLFMYRLLHNFAVISLTLKFLFDFTDHFKYIESSTLITVNFFTYFTLVFFALLSSMGSCYQWQNRILAVLKELKHQRDLSRHMGYRVPRSKQNSIDYLLFALTVLLILRLSIHLATFTLSARMGFNHPCNCFLPECMIFSMNYLLFAILAEITRCWWSLQSGLKMVLLNRQLSTVAFNLWEI

ERLHTRFQCLIDLTSEVCSIFRYVTLAYMARNLWSGIVAGYLLVRFVIGNGLQDVELVYLVFSFITCIQPLMLSLLVNSMTSTTGSLVEVTRDILKISHKKSVNLERSIEWLSLQLTWQHTHVTIFGVFRINRSLAFRSASLILVHVLYMVQSDYISITN

>DmGr89a

MLRFPHVCGLCLLLKYWQILALAPFRTSEPMVARCQRWMTLIAVFRWLLLTSMAPFVLWKSAAMYEATNVRHSMVFKTIALATMTGDVCISLALLGNHLWNRRELANLVNDLARLHRRRRLSWWSTLFLWLKLLLSLYDLLCSVPFLKGAGGRLPWSQLVAYGVQLYFQHVASVYGNGIFGGILLMLECYNQLEREEPTNLARLLQKEYSWLRLIQRFVKLFQLGIFLLVLGSFVNI

MVNIYAFMSYYVSLHGVPLTISNNCLVLAIQLYAVILAAHLCQVRSAKLRKKCLQLEYVPEGLTQEQAMASTPFPVLTPTGNVKFRILGVFILDNSFWLFLVSYAMNFIVVILQTSFEHINHGEI

>DmGr92a

MFEFLHQMSAPKLSTSILRYIFRYAQFIGVIFFCLHTRKDDKTVFIRNWLKWLNVTHRIITFTRFFWVYIASISIKTNRVLQVLHGMRLVLSIPNVAVILCYHIFRGPEIIDLINQFLRLFRQVSDLFKTKTPGFGGRRELILILLNLISFAHEQTYLWFTIRKGFSWRFLIDWWCDFYLVSATNIFIHINSIGYLSLGVLYSELNKYVYTNLRIQLQKLNTSGSKQKIRRVQNRLE

KCISLYREIYHTSIMFHKLFVPLLFLALIYKVLLIALIGFNVAVEFYLNSFIFWILLGKHVLDLFLVTVSVEGAVNQFLNIGMQFGNVGDLSKFQTTLDTLFLHLRLGHFRVSILGLFDVTQMQYLQFLSALLSGLAFIAQYRMQVGNG

>DmGr93a

MFSSSSAMTGKRAESWSRLLLLWLYRCARGLLVLSSSLDRDKLQLKATKQGSRNRFLHILWRCIVVMIYAGLWPMLTSAVIGKRLESYADVLALAQSMSVSILAVISFVIQARGENQFREVLNRYLALYQRICLTTRLRHLFPTKFVVFFLLKLFFTLCGCFHEIIPLFENSHFDDISQMVGTGFGIYMWLGTLCVLDACFLGFLVSGILYEHMANNIIAMLKRMEPIESQDERYRM

TKYRRMQLLCDFADELDECAAIYSELYHVTNSFRRILQWQILFYIYLNFINICLMLYQYILHFLNDDEVVFVSIVMAFVKLANLVLLMMCADYTVRQSEVPKKLPLDIVCSDMDERWDKSVETFLGQLQTQRLEIKVLGFFHLNNEFILLILSAIISYLFILIQFGITGGFEASEDIKNRFD

>DmGr93b

MVYGFTMSGLLVMPRILRCLNVSRISAILLRSCFLYGTFFGVITFRIERKDSQLVAINRRGYLWICLVIRLLASCFYGYSYDAWSGQYEDMYLRAFFGFRLIGCLICSVIILVMQFWFGEELINLVNRFLQLFRRMQSLTNSPKNRFGDRAEFLLMFSKVFSLLFVFMAFRLMLSPWFLLTLVCDLYTSVGTGMITHLCFVGYLSIGVLYRDLNNYVDCQLRAQLRSLNGENNSFRNNPQPTRQAISNLDKCLYLYDEIHQVSRSFQQLFDLPLFLSLAQSLLAMSMVSYHAILRRQYSFNLWGLVIKLLIDVVLLTMSVHSAVNGSRLIRRLSFENFYVTDSQSYHQKLELFLGRLQHQELRVFPLGLFEVSNELTLFFLSAMVTYLVFLVQYGMQSQQI

>DmGr93c

MIERLKKVSLPALSAFILFCSCHYGRILGVICFDIGQRTSDDSLVVRNRHQFKWFCLSCRLISVTAVCCFCAPYVADIEDPYERLLQCFRLSASLICGICIIVVQVCYEKELLRMIISFLRLFRRVRRLSSLKRIGFGGKREFFLLLFKFICLVYELYSEICQLWHLPDSLSLFATLCEIFLEIGSLMIIHIGFVGYLSVAALYSEVNSFARIELRRQLRSLERPVGGPVGRKQLRIVEYRVDECISVYDEIERVGRTFHRLLELPVLIILLGKIFATTILSYEVIIRPELYARKIGMWGLVVKSFADVILLTLAVHEAVSSSRMMRRLSLENFPITDHKAWHMKWEMFLSRLNFFEFRVRPLGLFEVSNEVILLFLSSMITYFTYVVQYGIQTNRL

>DmGr93d

MKATKYSVGILRFMSFYARFLSLVCFRLRKQKDNNVWLEEIWSNRSRWKWISVTLRIVPLCIYAFTYAEWISNRMLITEKFLHSCSLVVSIPCYLSIIHLKICHGPEVTKLVNQYLHIFRLGTLDIRRRSQFGGGRELFLLILSVCCQIHEYVFILVIASRLCGFQHIIWWVSYTYVFIICNSIMCFGFIWHLSLGVLYAELNDNLRFESGFQTAFLRKQQRIRVQKSMALFKEISS

VVTSLQDIFNVHLFLSALLTLLQVLVVWYKMIIDLGFSDFRIWSFSLKNLIQTLLPVLAIQEAANQFKQTRERALDIFLVGKSKHWMKSVEIFVTHLNLSEFRVNLLGLFNVSNELFLIIVSAMFCYLVFVTQCVIVYRRRYVI

>DmGr94a

MDFTSDYAHRRMVKFLTIILIGFMTVFGLLANRYRAGRRERFRFSKANLAFASLWAIAFSLVYGRQIYKEYQEGQINLKDATTLYSYMNITVAVINYVSQMIISDHVAKVLSKVPFFDTLKEFRLDSRSLYISIVLALVKTVAFPLTIEVAFILQQRRQHPEMSLIWTLYRLFPLIISNFLNNCYFGAMVVVKEILYALNRRLEAQLQEVNLLQRKDQLKLYTKYYRMQRFCALADE

LDQLAYRYRLIYVHSGKYLTPMSLSMILSLICHLLGITVGFYSLYYAIADTLIMGKPYDGLGSLINLVFLSISLAEITLLTHLCNHLLVATRRSAVILQEMNLQHADSRYRQAVHGFTLLVTVTKYQIKPLGLYELDMRLISNVFSAVASFLLILVQADLSQRFKMQ

>DmGr97a

MRFLRRQTRRLRSIWQRSLPVRFRRGKLHTQLVTICLYATVFLNILYGVYLGRFSFRRKKFVFSKGLTIYSLFVATFFALFYIWNIYNEISTGQINLRDTIGIYCYMNVCVCLFNYVTQWEKTLQIIRFQNSVPLFKVLDSLDISAMIVWRAFIYGLLKIVFCPLITYITLILYHRRSISESQWTSVTTTKTMLPLIVSNQINNCFFGGLVLANLIFAAVNRKLHGIVKEANMLQSP

VQMNLHKPYYRMRRFCELADLLDELARKYGFTASRSKNYLRFTDWSMVLSMLMNLLGITMGCYNQYLAIADHYINEEPFDLFLAIVLVVFLAVPFLELVMVARISNQTLVETRRTGELLQRFDLQHADARFKQVVNAFWLQVVTINYKLMPLGLLELNTSLVNKVFSSAIGSLLILIQSDLTLRFSLK

>DmGr98a

MEQMSGELHAASLLYMRRLMKCLGMLPFGQNLFSKGFCYVLLFVSLGFSSYWRFSFDYEFDYDFLNDRFSSTIDLSNFVALVLGHAIIVLELLWGNCSKDVDRQLQAIHSQIKLQLGTSNSTDRVRRYCNWIYGSLIIRWLIFIVVTIYSNRALTINATYSELVFLARFSEFTLYCAVILFIYQELIVGGSNVLDELYRTRYEMWSIRRLSLQKLAKLQAIHNSLWQAIRCLECYFQ

LSLITLLMKFFIDTSALPYWLYLSRVEHTRVAVQHYVATVECIKLLEIVVPCYLCTRCDAMQRKFLSMFYTVTTDRRSSQLNAALRSLNLQLSQEKYKFSAGGMVDINTEMLGKFFFGMISYIVICIQFSINFRAKKMSNEQMSQNITSTSAPI

>DmGr98b

MVAQKSRLLARAFPYLDIFSVFALTPPPQSFGHTPHRRLRWYLMTGYVFYATAILATVFIVSYFNIIAIDEEVLEYNVSDFTRVMGNIQKSLYSIMAIANHLNMLINYRRLGGIYKDIADLEMDMDEASQCFGGQRQRFSFRFRMALCVGVWMILMVGSMPRLTMTAMGPFVSTLLKILTEFVMIMQQLKSLEYCVFVLIIYELVLRLRRTLSQLQEEFQDCEQQDMLQALCVALKR

NQLLLGRIWRLEGDVGSYFTPTMLLLFLYNGLTILHMVNWAYINKFLYDSCCQYERFLVCSTLLVNLLLPCLLSQRCINAYNCFPRILHKIRCTSADPNFAMLTRGLREYSLQMEHLKLRFTCGGLFDINLKYFGGLLVTIFGYIIILIQFKVQAIAANRYKKVVN

>DmGr98c

MEMEAKRSRLLTTARPYLQVLSLFGLTPPAEFFTRTLRKRRRFCWMAGYSLYLIAILLMVFYEFHANIVSLHLEIYKFHVEDFSKVMGRTQKFLIVAIATCNQLNILLNYGRLGLIYDEIANLDLGIDKSSKNFCGKSHWWSFRLRLTLSIGLWMVIIIGVIPRLTLGRAGPFFHWVNQVLTQIILIMLQLKGPEYCLFVLLVYELILRTRHVLEQLKDDLEDFDCGARIQELCVTL

KQNQLLIGRIWRLVDEIGAYFRWSMTLLFLYNGLTILHVVNWAIIRSIDPNDCCQLNRLGSITFLSFNLLLTCFFSECCVKTYNSISYILHQIGCLPTAEEFQMLKMGLKEYILQMQHLKLLFTCGGLFDINIKLFGGMLVTLCGYVIIIVQFKIQDFALIGYRQNTSDTS

>DmGr98d

MEANRSRLLAAARPYIQIYSIFGLTPPIQFFTRTLHKRRRGIVILGYACYLISISLMVIYECYANIVALQKDIHKFHAEDSSKVMGNTQKVLVVAMFVWNQLNILLNFRRLARIYDDIADLEIDLNNASSGFVGQRHWWRFRFRLALSVGLWIVLLVGLTPRFTLVALGPYLHWTNKVLTEIILIMLQLKCTEYCVFVLLIYELILRGRHILQQISVELEGNQSRDSVQELCVALKR

NQLLAGRIWGLVNEVSLYFTLSLTLLFLYNELTILQIVNWALIKSVNPNECCQYRRVGTCLLLSINIFLSCLYSEFCIQTYNSISRVLHQMYCLSAAEDYLILKMGLREYSLQMEHLKLIFTCGGLFDINLKFFGGMVVTLFGYIIILVQFKIQFFAQSNFMQNINSTELKAYTA
