# Supplementary material for: Transcriptomics reveal the molecular underpinnings of chemosensory proteins in Chlorops oryzae
Source: BMC Genomics. 2018 Dec 7;19:890. doi: 10.1186/s12864-018-5315-4 (PMC6286535; doi:10.1186/s12864-018-5315-4)
Supplement: Supplementary file 6 — Protein sequences of IRs used to construct phylogenetic tree. (DOCX 72 kb) [file 12864_2018_5315_MOESM6_ESM.docx]

>Co-Cluster-13720.0

MGPFQWPVWVALTLVYLGAVFPIVFTDRLSLSHILGNWGEMENMFWYVFGMFTNSLTFSGKYSWSSSKKLSTRILIGFYWFFTIIITACYTGSIIAFVTLPAFPETIDSVMDLLGLFFRVGTLDKNGWETWFQNSTHWPTAKLYKKMEFVASLEEGIGNVTQSFFWNYAFLGSKAQLECLVQANFSDENISRRSALHLSEECFALFQIGYIYPRDTVYKRKIDSMILLAQQSGLMNKIEQEVRWTMQRSSTGKLLQASSGASLRERIQEERQLTTADTEGMFLLMGIGYALGAIALVSEIVGGITNKCRQIIKRSRKSISSGFSSQRSSAVGTRTEAEQEAYDRHKAERRREAKNAHMNAHDNGVFGVKQFNLTRETLKELYGNYNKQEPNYSVKDGKLILESDVISTDTGSVSRESSTEFLIPPLCVKKKTIVLAEIDNNREVPENKADDDPIDDDIQNTLDGLDQCIARHDVCREASQDNVMFGQLTESDLFGSYIEPTIEVDSERKLDNLAMFKDVEQEDDVESNSENQTK

>Co-Cluster-11061.0

MRQAFLSFCCCALIINVGGANYGDLLANLNGCNDYLHSLRLLLTELALIAKIERCLVVVTDEAHLGIYEGSFFDWSNQPKSAFILHVDDDDDLLAPNYRTVKALKEIRNSQCDLYLITILNGLQVKRFLRFIDRNRVLDMQKRFVFLSDNRLLERDMVYIWSSMVSSIFLEPNRNFKRYTISTIAYPEILNGQFVITQKIKDWKPGKRLREAAIFPEKISNFKGMRLPIAVYPHIPMVQRLPSQSEFEGLEIEIVRSLGVAMNFEPYFYETRDSEIERWGRQLPNGSFTGLLGELVSDKSYLNDLI

>Co-Cluster-19206.0

MRQAFLSFCCCALIINVGGANYGDLLANLNGCNDYLHSLRLLLTELALIAKIERCLVVVTDEAHLGIYEGSFFDWSNQPKSAFILHVDDDDDLLAPNYRTVKALKEIRNSQCDLYLITILNGLQVKRFLRFIDRNRVLDMQKRFVFLSDNRLLERDMVYIWSSMVSSIFLEPNRNFKRYTISTIAYPEILNGQFVITQKIKDWKPGKRLREAAIFPEKISNFKGMRLPIAVYPHIPMVQRLPSQSEFEGLEIEIVRSLGVAMNFEPYFYETRDSEIERWGRQLPNGSFTGLLGELIDRQARIAIGDLRLYKLYYSVIDFTKPHSHECMTFLTPESSQDDSWKTLISPFSGAMWAGVLLSLFAVGTVFYTISF

>Co-Cluster-11063.0

MLAKAAITRTKQRQCRKVPGCVPRIHIDGVVGGHERHSMLVLDAFLFYVGSICQQGLTFSTSFISGRCIVITSLFFAFAIFQFYSASIVSSLLMERPKTIRTLQDLVHSSLEIGIEDILYNRDYFRRTKDPDAIELYNKKIASFDSGTTNITKHYGSKSSAATNWYTPAQGMARLKRGKFAFHVDVATAYKIIADTFTESEICELTEIELFPVQKTVACVQKGSPLRKMITYGLRRVGETGLMDYQRKIWRSPKP

>Co-Cluster-16299.3

MLSQVRLVLLSLVTFLTQGCTAAFDDGICLSNHLIEKHRFNRQIYRDCNEDFSASYVRLPRRVDPTFRGNPKPRSDVLAAKFHVNANANANDDQKHSLVNLINKIAIEYLAKCPPVIYYDHFVEKSDGLFLENLFKTLPITFYHGEINEKYEAINARLKGRIDVQCKSYILFLSDPLMTRKIIGPQVESRVVLVARSSQWKLRDFLSSEKSSNIVNLLVIGESYTADLAEVSQQTIVILKKKKKTKHKDASLIEYFYPQERPYVLYTHKLYTDGLGSNKPVVLTSWINGALSRPHINLFPKKFQHGFAGHRFNVASVNQPPFIFSIKNIVSGGSITINWDGIEYRLLNMIAQKLNFSIDISEPPTRWNTRGYEGPANTICFFFFFS

>Co-Cluster-3781.106530

MCSPIDTVFAKSKWLIFFSFIGLIYENDNPDMEKVFHLAIDEANKEADGYRLHGISVAIEPGNAFDTSKKLCKMLKQNLVAVFGPTSNMAARHAMSICDAKELPFVDTRWDFNTQLPTINLHPHPGQLANLLKDMVLALEWDTFTIIYESGEYLQTVNELLRMYEPDGPTVTVRRYELDLNGNYRNVLRRIRNSDDDSFIVVGSMATLPELLKQAQQVGLMTADYRYIIGNLDLQTSDLEPFQHSDTNITMFRLVSPENEAVLKVAKAVYESEDPYQNVSCPLTVSMALVYDGVQLLAQTFKHVMFRAVPLSCNNDSSWDKGYTLVNYMKSLSIEGLSGEIQFDYQGLRSEFTLEVAELLVSGVQNIGIWRPETGFVENRPAMAAPSIVDQRSLVNQTFIVITAISEPYGMLKDTSKKLKGNEQYEGFGIELIDELSKKLGFLYTFVLEPKNNYGSCSATTGECTGMLKEIIEGRADLGITDLTMTSARESGVDFTIPFMNLGISILFRKPRKEPPKLFSFMSPFSGEVWLALGMAYISVSVTMFLMGRICPTEWDNPYPCIEDPTELENQFSFANCLWFAIGALLQQGSELAPKGYSTRAVASFWSFFTLILVSSYTANLAAFLTVESLSTPIENAEDLAAQKGGVVYGAKINGSTYGFFQGAKYPTYQKMYEFMRNNPQYMTLSNPEGVKRTENENYAFLMESTSIEYTTERHCTLTQVGKLLDEKGYGIAMRKNWEYRDILSKGVLELQEAGVLAQMKTKWWKEKRGGGACTQEKNDSAAKLGIANMGGVFLVWGVGSAVACVVGLLEWLISVYNTSRGNKVPFKNELMDELYFILQCSGNTKPVKYPKSSHRSKSRSSDTSSSKRSSASVDSILMDEGKAQLERTKQVENGK

>Co-Cluster-3781.31592

MDMFHIYNQWLFFILDDKRRNFDAMSITQNLEEGANIAFALNETLLDCVDSINCTIAEISFAFVSSISRIITEEQSIQGKITDEEWEAIRLTKQEKQDQILGYMKDFLKAKSKCSCSRWRMITSISWGKSQEHRRYRPHNDHNEASNRNFEFLDVGYWSPLLGFVTQELLFPHIEHHFRNITMDILTSHNPPWQIISKDSRGSITEHKGITMEILKELSRMLNFTYVLHQPNVIEIGMEPIRNDSTLMGSLTHVIPFEVVEMVQGKNFFLAAVAATIDEPDRKIFNYTVPISVQKYSFLTRRPDEISRIYLFTAPFTLETWACLAAVMLITAPVLYLINRLVPMQHLKTTGLCRINNCCWYIYGALLQQG

>Co-Cluster-3781.15697

MDPFSGEVWMYLGFAYFGVTLSLFILGRLSPTEWDNPYACIEEPEELENQFTFNNAMWFTTGALLQQGSEIGPKALSTRTVATIWWFFTLIIVSSYTANLAAFLTVEKPTSIIDNVNDLAENKGGVRYGAKRNGATRNFFQTSEDPVYHQMDEYMTKNLDLLVDDNQVGVERVLKEDRYAFLMESSSIEYHTVRNCDLEKIGEPLDEKGYGIAMKKEWEYRDRFNNALLALQEQGTLAKLKNKWWNEIGAGVCNTKSGADAESHPLDMKNLGGVYLVLFVGSALSICVGMLIWLVNVFIKAKRHEVPFIDALKEEWSVVIDFKNNEREVRSPASVYSKRNSLMSIDSIETDSEKEAQIEDERTSSV

>Co-Cluster-3781.157618

MLTPLYLKSSKSNVWMSLDLDVWKYLRPIDLKRKYNVVSNFPQSTLA

>Co-Cluster-3781.159004

MANEADTSHLNHRALTVNVFPNQLILSKAYADIVNNFGWRKFTILYYHIDENAPAHLQDLLQLRDVHKELIRVRKFKKKDDFRLLWKNINGERRVVLDCPPDVLVEVLNAAMQFNMMSELNHMFLTNLDTHTSNRGDLKNNNKIMSNITAARVMMDKDQTV

>Co-Cluster-3781.161973

MIIKILNKLKILFPIITFISLSDNVVGQTSQNINVLFINEVDNEPAAKAVEVVQTYLKKSPSYGLSVQIDQVEVNKTDAKALLEMICIKYAEMIGKPQPPHVILDTTKSGIASETVKSFTQALGLPTVSASYGQEGDLRQWRDIEESKQKYLLQVMPPADMIPEVVRSIVRKMNITNAAILYDDTFTMDHKYKSLLQNIQTRHVISRIAKNGPEAL

>Co-Cluster-3781.174214

MYLNMMSHTAYGKPYEWSKDDFELGAPMGTRFNTFQMDNFGWRKPNGSFDGLMGMFQRYELDFAHMAIFMRSDRIPLVDFVGETFRIRAGIIFRQPPLSAVTNIFALPFDKYVWISILILMIITILLFCIEVKYSPHRHNMNFLDCCDFVWGAMCQQGFYVDVGNRSGRIIVLNTFIATLFLFTSFSANIVALLQSPSESITSLAALSQSPLEIGVQDTQYNKIYFTESTDPVTNRLYHKKIAPKGDNIFMRPIQGIKKMQTGLFAYQVELQAGYQIISETFSEPEKCGLKELEPFQLPMISLPTRKNFPYKELFRRQMLWQREVGIMNREELKWIPQKPKCEGGVGGFVSIGLTECRYALAMFGFGMLLSFIIFLWEISMKYFIKFMPQLKKLNEIHVPPAQTAP

>Co-Cluster-3781.1879

MNSKSLCSSINMEESYCLTEFTFQRAVILSVLRSLAMYMKANPEKLQPLRASCIETNATGKELPDLFGKLEFPSFIQFVEPESDGNEAEEEDGDESHPKLSYAVNITMGTYASEEGTGTQLAVWNSGHLDRINATITPAKRFFRVGTTEAIPWSYLLRDEKTNELILDENGNPQWVGYCIDFLIALAEKCNFDYELIEPKKGKFGERNAITGEWDGIVGDLVTGETDFALTALKMYSEREEVIDFL

>Co-Cluster-3781.19630

MKNLTADTVYRIYTVEQAPFIMRDETAPKGYKGYCIDLIDEIAEIVNFDYTIQEVEDGKFGNMDEKGEWNGIVKKLIDKQADIGLGSMSVMAEREIVIDFTVPYYDLVGITIMMLRPSQPSSLFKFLAVLETNVWLCILAAYFFTSFLMWIFDRWSPYSYQNNREKYKDDDEKREFNLKECLWFCMTSLTPQGGGEAPKNLSGRLVAATWW

>Co-Cluster-3781.20802

MWQAMQEAELPSTLAEAVARVRNSTSATGFAFLGDATDIRYLVMTNCDLQNVGEEFSRKPYAIAVQQGSHLKDQFNNAILTLLNKRQLEKLKEKWWKNDEIQAKCDKPEDQSDGISIQNIGGVFIVIFVGIGMACITLIFEYWWYKYRKTPRVIDVIEAQTSSKDGQPNDGVILGQSGKDHDKTVNALRPRFHNYPATLKPRF

>Co-Cluster-3781.22149

MLDLFVKIFLLPLVSYAVVSRAEYMPTFGSPRIGPSESIPIGLISDNNAEDLGKVFDYAIQVANTDLSIPLSGQQKVIEYGDMVDGFIQLCRFLEAGVGAVFGPSSKPTSKHLLNVCDTKDVPFIFPHMIESLEGFNLQPNSLDIARVLRSIIDAYEWKHIVLLYENSEYLSIVMRLLEFYEEYGPVITIIRYDLKLNGNYKSV

>Co-Cluster-3817.3

MTTYRLRYIHILAETGSFHSICLFRTPRNTGGTGSGQAFLDPFTPPVWVVFGTILFLSSVQLWAMFFVEFYRMPKNLDHAPSLLTSCLITLGTACVQGAYVMPRSVGGRATYTVVALATFIMYNYYTSIIVSTLLGSPIKSDIKTVGQIADSALDVGFEPRPWIFSYLNSSQVPA

>Co-Cluster-5769.0

MLYLLFKTQLGEQMGSFVRISSLQEFLIAWILFSYILSTIYFGKLESSFVSPAFESELNSLDELDKLNVPIYAIANVFDVVEYTLEPKYRKLINAHAIRLPLNFSAHSWAATFAKKSRTKKLGFILRDETAKDFLASTYNSKAGRPQFHAVKEFLRSMPRTYIVTPGSPFLEKFQYFVGAFFESGLLEHWLNEDLGNRLWHMANADTFQEEFISDEIGSELGTGIYEAPNFERKHVVLDMHILQGAFYVWLVGIGISMFTFAYEYIPWMRRRRIVAPI

>Co-Cluster-8273.0

MTTAEYETQSRTISKELNDMQQSGTDISTDSTKSNDDRGGVNQGNGKSSVNSDEQQQVQDTSMVIQPLNLRMLQGAFIVLLVGYGMAG

>CstyIR25a

MILPRLKFIHIVLLFLKILSRRYLLVSSQTSQNINVLFINELDNDPASKAIDIVQTYLKKNSNYGLSVQIDKIEANKTDAKALLESICIKYAESIENKQPPHVVFDTTKSGIASETVKSFTQALGLPTVSASYGQEGDLRQWRDMEESKQKYLLQVMPPADIIPEVVRSIVRKMNITNAAILYDNTFVMDHKYKSLLQNIQTRHVITAVAEGDSARADQIERLRNLDINNFFILGSLKTIGQVLESVKPAFFERNFAWHAITQNEGEVSSKRDNATIMFLKPIVYTQNRERLGQLRTTYNLNEEPQIMSVFYFDLALRTFLAVKDMLQSGAWPANMEYLGCDDFQGGNTPERNIDLRQAFVQVTEPASYGDFDLVTQPGKPFNGYSFFKFDMDVNVVQIRGGNSVNSKSIGRWTAGLDSPLVVNDEEAMKNLTADTVYRIFTVVQAPFIMRDETAPKGYKGYCIDLINEIAEIVHFDYTIEEVEDGKFGNMDEKGEWNGIVKKLIDKKADIGLGSMSVMAEREIVIDFTVPYYDLVGITIMMQRPSTPSSLFKFLTVLETNVWLCILAAYFFTSFLMWVFDRWSPYSYQNNREKYKDDDEKREFNLKECLWFCMTSLTPQGGGEAPKNLSGRLVAATWWLFGFIIIASYTANLAAFLTVSRLDTPVESLDDLAKQYKILYAPLNGSSAMVYFERMANIEQMFYEIWKDLSLNDSLSPLERSRLAVWDYPVSDKYTKMWQAMQEAQLPATLDEAVARVRNSTTATGFAFLGDATDIRYLVMTNCDLQVVGEEFSRKPYAIAVQQGSHLKDQFNNAILTLLNKRQLEKLKEKWWKNDEAQSKCDKPEDQSDGISIENIGGVFIVIFVGIGMACITLVFEYWWYKYRKNPRIIDVAEAASTPPGKDVKLAEGIILGQTGKEYEKANAALRPRFNQYPHNFKPRF

>CstyIR8a

MQLLLLTLLLCLNLTGLDGEQELRITFWIEPVQQAEFEADMLIVQKELNTMNLDVRITDAVIILTRSVNQDADFDSLCRILSNVGSSIVIDLTYTLWTDGYRLIQEKGIAYLRLERILRPFLEMFGDFMRQKRANNVAMIFSNQRDMAEAVQQMTEGFPFRTLLMDASQDKNFAERLQSLRPSPNYVAMFARGSAMNGLFEQVQKAGLFRRPTEWHFVFLDTRDRVFKYKRQAEFATRFTLNPRAICRAMQMRDLYCGSGFTLQRALLLDILRGLIGAVQISPSYPEPIYMDCNTSAEVNDRQDANAQDSAEWLEYVHWSNFITYATPLGYSFDDDSLKYEDGDNANTMQSLSFAINISAGYYSSEHEAKTDLARLSSVGEILLLNETISPARRFFRIGTAESIPWSYYRRNKQTGELILDRRGSPIWEGYCIDFIARLAEKLNFDYDIVEPQIGHMGQRNEQGEWDGVVGDLVSGQTDFAIAALKMYSEREEVVDFLPPYYEQTGISIVIRKPVRRTSLFKFMTVLRVEVWLSILAALVGTALMIWFMDKYSPYSSRNNREAYPYACREFTLRESFWFALTSFTPQGGGEAPKAVSGRMLVAAYWLFVVLMLATFTANLAAFLTVERMQTPVQSLEQLARQSRINYTVVHESDTHQYFINMKFAEDTLYRMWKELALNASKDFHKFRIWDYPIKEQYGHILLAINSSMPVRNAREGFTKVNNHENADYAFIHDSAEIKYEITRNCNLSEVGEVFAEQPYAVAVQQGSHLGDELSYAILELQKDRFFEQLKAKYWNQSNVQNCPLSEDQEGITLESLGGVFIATLFGLVLAMITLGGEIIYYKKRRKQLQTSLPITQVKPLDEPERDEQSNPRRAWHVPLPKSKPPAKITPPPSFEAATSKGKKLSKKITLGDAKFKPRHGLQSRRELGAPSGMGYIE

>CstyIR110

MIKLQVKVISWVFIILTGFLCTPQIESINTNFLELAAFEDFLRSQHLQQSLVVRIKGRDGDGDGDEDGDWKIECHQKLLANYRVQYYQPGISHNFEDLMYYGAPRTAVLVLKFEHRLVKQWVYTVASKAGYFNNSLAWFMLGTSRASQLDEAQITEHLGGYKMGIDVDITVALKARDNTSWRLYDVYRIDQQVSSAPLIVERKGEWSPARGYTLMDTFRRSWIIRRRNFRNVTLRGSTAVTEKPSGYDDMAYLANDKQLLQLDPMQRKTYQLFRLVERMYNLSLAISFTDNWGQQLANGRWSGVMGQITNGEADFAVCPIRFVPERQLHVQYSPVLHTQPIHFLFRHPRHSHIRNIFFEPLSSQVWWCVLVLITFSILLLVLLIRHEALQNGNLVETRVAFVWFTMLETYLQQGPATELFRLMSTRLLISASCIFSFMLMQFYGAFIVGSLLSDSPRSIVNLQALFESNLDIGMENISYNFAVFGNTSGQLVRDVYSKKICRSGEHNVITLEQGAERIIKGRFAFHAAIDRLYRLLLEMRMEEHEFCELQEIMFNPPYAAASVMAKGSPWREHLAHAILHLKATGLMQYNDRLWSVPKPDCSLFKASQVEVDLEHFAPALFALVLAMIASALVFLLELMFSRLAPFRPS

>CstyIR107

MVNDQEFQKSTSGPSTRGNSDPFFRQAKTSALFLFTSFSANIVALLQSPSDAIQSLSDLGQSPLEIGVQDTQYNKIYFTESTDPVTKNLYHKKIASKGENIYMRPLLGMEKMRTGLFAYQVELQAGYQIVSDTFSEPEKCGLMELEPFQLPMLAIPTRKNFPYKELIRRQLRWQREVSLVNREERKWIPQKPKCEGGVGGFVSIGITECRYALGIFGCGAAASFGLFLLEFIFKYFKQVYRIIKGYREMQR

>CstyIR108-109

MKEMAASNQKEASSTIEETAETFIWKRTTASTLLNKASTIKETAASALEEEQIEQRRKLFVLLLLLLWPLTASTSMSAPADASIPRGQQEQKQLLKDDSWGNQLPDMLVAYYRQHGVHSLMLVVCRGDIDAARLHALLQHFMAHNIYVQLWTEQCLNDLRHVGNETDTPEAPPPRSFQADNETHWELAFVLPALNYKLGILLLQFNSSCALNLLRWSAATEHNYFTTNRYWLLLTQDLQELQLLEDAEIFLPPDSEVRVLWHATSQPFNATLLDVYKVAAWKPLKRRLVGHRLRNPRHMVHALQHFGSAITYRQNLEGIVFNTAIVIAFPDLFTDIEDLSLRHIDTISKVNHRLMLELAVRLNMSYNTYQTVNYGWRQPNGSFDGLMGRFQRYELDFAQLGIFMRLDRIALCDFVAETYRVRAGIMFRQPPLSAVANIFSMPFQQDVWISILVLLIVTMLVMLLEMFFSPHTHNMSYLDSVNFVFGAMCQQGFYVNVSNRSARIIVFTTFVAAVFLFTSFSANIVALLQSPSDAIQSLGDLGQSPLEIGVQDTQYNKIYFTESTDPVTKSLYHKKIASKGDFVYMRPVLGMEKMRTGLFAYQVELQAGYQIVSDTFSEPEKCGLMELEPFQLPMLAVPTRKNFPYKELFRRQIRWQREISLVNREERKWIPQKPKCESGVGGFVSIGLTECRYAFGIFGCGAALSFGVFLIELIRKHSHGIYTFVRGYKHTLH

>CstyIR106

MWGAWMVIALFSSHWMAGKCLDRQDFPENCVSRRLIKRYQLNEDLYGSCPPRRKRRVDPTFHGNPKPRADLLAAKFHVNAYNFDQTNSLVGLVNKIAEEYLQKCPPIIYYDSFVEKSDGLILENLFKTLPITFYHGEINSRYEAKNTHFTSHIDSNCKSYILFLSDPLMTRKILGPQTESRVVLVSRSTQWKLRDFLASELSSNIVNLLVIGESLMADPLRERPYVLYTHKLYADGLGSNTPVVLTSWIKGALSRPHIDLFPAKFQNGFAGHRFQVSAANQPPFIFRIRTLDSSGIGQLRWDGVEFRLLNMISKRLNFSVDITETPTRAYTRGVVDNIQQQIAQRTIDIGMSGIYLTEERLRDTDMSVGHSRDCAAFITLASKALPKYRAIMGPFQWPVWVALICVYLGGIFPIVFTDRLTLSHLLGNWGEVENMFWYVFGMFTNAFTFTGKYSWSNTQKISTRLLIGAYWLFTIIITSCYTGSIIAFVTLPAFPDTVDSVMDLLGLFFRVGTLDNGGWESWFQNSTHVPTSRLFKKMEFVGTLEEGIGNVTQSFFWNYAFLGSKAQLEYLVQSNFSDENISRRSALHLSEECFALFQIGFLFPRESVYKRKIDSMILLAQQSGLITKINKEVSWAMQRSASGRLLQASSSTSLREIIQEERQLTTADTEGMFLLMALGYFLGATALVSEIVGGITNKCRQIINRNRKSASSSWSSRHSSANGGEERTADEQLAHDERKAARREAAEDAQKMSFGMREFNLTRTTLRELYGSYNRSDPTSSQQPEEHVPTHMHTHTYLEDKDSREALESLERLDEFMDQMGTEEEEPAPSASIVDEVFGPNTNNSTNET

>CstyIR105

MQLNIAFIVVCGLCHWLPCVQLGNPTDPIDLQLQEKLLKILERIRLERNYDTIVIYGQSGGEDCLFHELLPKLQLPTVLLTKGSSLSDWSFSSDSLLLCCDSKAEQEQNTRSLLRLQQARRLVYLESEMQPQWLCEDYFEREQHNVAMMNAQGDLFSCRLFQEINHVQLDINSDSKYNSDSIYVQQFRNMKGAVIRSEPDQLAPRSMLYHDPVTGNIKMGGYVANVVNTFVERVNATLPLRDDLKFGENYGLFDLINRTYHNQLDIATSLLGTFGDENLDYVSYPYYSTSYCFMLPVPAKLPYNQIYTIIVDPPVLGIIMVLFVILSLLLIYSQELSWHQLSLANVILNDRCLRGLLGQSFILSDNPSRHMKAICLILFFAGLMITTMYQAYLQTLYTSPPLEEMLHSFEDFQNSRYKIALWRYEMEKVESISNVSLSTARNIAIFDLYTDFIKIRDTFNASFIYTVSKQRWRTYAEQQKSFKEQAFYYSDDVCFSRFVLFGFPLRRHLPYRQQFEQHILHMHAFGLTQFWISRSFYDMVHFNTTTLKDYSNTRMHGAVFLRDLVYIFEFYAAFHLLACFCFAIECFWARFRRTVAIKNV

>CstyIR104

MLPSAVHNVSLVYALVWAIDNYYGIATSTPLAVVQFPTSRESRRLHNDLIDAALGRSSGTGRIQFLLEDDRVEMTETDTDPPPPSGLTGRPIAIWFLDSLRSYFRLEMYLNQLGSPYKRNGFFLVVYTGLEDQPMESLKIMFRRLLNMYVLNVNVFLQRDETVHLYTYYPYGPHHCQSSLPVYYTAFQDLPAPATGFGLTKPLFPSKLANMHGCELVVATFEHRPYVIIEDDPKTPGGRSIHGIEGLIFRSLAERMNFTIKMVERKDKNRGEILPDGNFTGILKMMVDGEVNLTFVCFMYSKARSDLMLPSISYTSFPIVLVVPSGGSISPMGRLTRPFRYIIWSCILVSLICGFLLIFLLKITAVPRLRNLVLGRRNRLPFMSMWASLLGGLALYNPQRNFARYILVMWLLQTLILRAAYTGQLYILLQDVDVRSPIKSLSEVQAKDYEFRILPALRTVFKDSMPNTNFHVVLSPEESLYRLRDEDDPGIAVPLLQPTINQFDFRSGPKKRHLTVLPDPLMTAPLTFYMRPHSYFKRRIDRLIMAMMSSGIVARYRKMYIDRIKQVGKRRNLEPKPLSIWRLSGIFVCCAGLYMLALIVFILEILTRTHRRLRRALNVINRYAA

>CstyIR103

MVGNYSVSYSYPIGINDWCSMVPYRNQSPAEAYIREALENSAWILLLLSVLYITIAIWWCSPIRPRDFSAAFLQSICTVTYSAPIYIIKTPTHRIRYLYIVLFAMGLISSNMYISKMTSYFTSSPPQRQVNTVQDIIEANLRILVMTYENDHIRSLRNQFPDDFLKQLNSTRMGVIFEHL

>CstyIR101

MWGAWMVIALFSSHWMAGKCLDRQDFPENCVSRRLIKRYQLNEDLYGSCPPRRKRRVDPTFHGNPKPRADLLAAKFHVNAYNFDQTNSLVGLVNKIAEEYLQKCPPIIYYDSFVEKSDGLILENLFKTLPITFYHGEINSRYEAKNTHFTSHIDSNCKSYILFLSDPLMTRKILGPQTESRVVLVSRSTQWKLRDFLASELSSNIVNLLVIGESLMADPLRERPYVLYTHKLYADGLGSNTPVVLTSWIKGALSRPHIDLFPAKFQNGFAGHRFQVSAANQPPFIFRIRTLDSSGIGQLRWDGVEFRLLNMISKRLNFSVDITETPTRAYTRGVVDNIQQQIAQRTIDIGMSGIYLTEERLRDTDMSVGHSRDCAAFITLASKALPKYRAIMGPFQWPVWVALICVYLGGIFPIVFTDRLTLSHLLGNWGEVENMFWYVFGMFTNAFTFTGKYSWSNTQKISTRLLIGAYWLFTIIITSCYTGSIIAFVTLPAFPDTVDSVMDLLGLFFRVGTLDNGGWESWFQNSTHVPTSRLFKKMEFVGTLEEGIGNVTQSFFWNYAFLGSKAQLEYLVQSNFSDENISRRSALHLSEECFALFQIGFLFPRESVYKRKIDSMILLAQQSGLITKINKEVSWAMQRSASGRLLQASSSTSLREIIQEERQLTTADTEGMFLLMALGYFLGATALVSEIVGGITNKCRQIINRNRKSASSSWSSRHSSANGGEERTADEQLAHDERKAARREAAEDAQKMSFGMREFNLTRTTLRELYGSYNRSDPTSSQQPEEHVPTHMHTHTYLEDKDSREALESLERLDEFMDQMGTEEEEPAPSASIVDEVFGPNTNNSTNET

>CstyIR93a

MRPPALVFSVFCGLLLYVPAPVGANDFSSFLSANASLAVVVDYEYMAMHRQNILAHFEKILSDIIRENMKNGGINVRYFTWNAVKLKKDFLAAITIMDCESTWNFYKNTQITSILLIAITDSDCPRLPLNRALMIPMVAQGDEFSQILLDAKVQGIFKWKSVAVFVDQSILEENPMLVKSILHESTINHIPPLSVILYKIDETLRGQQKRVALRQALAQFAPTKHELKRQQFLVISKFHEDIIEIAETLSMFHVNNQWMFFVSEQLHADFDASTVTINLDEGANIAFALNETVSDCVDTLNCTISEVSMAVVTSLSRMILEEQSIYGEISDEEWESIRFTKHEKQDEMLQYMKEYLKANSKCASCAKWRFETAITWGKSQENRKFRMAPTRDTKNRNFEFINIGYWSPLLGFVCHELAFPHIDQHFRNITMDIVTVHNPPWQILTKDSRGAIVEHTGIVMEILKELSRALNFSYYLHEARSPDYEYSLAQSTNESDELMGSMTYRIPYRVVELVQGSGYFMAAVAATIDEPHKKRFNYTQPISIQKYTFILRQPDEVSRIYLFTAPFTLETWGCLAGILLVTAPMLYIVNRLVPLQELQIRGLSTVKNCFWYIYGALLQQGGMYLPRADSGRLVVGFWWLVVIVLVTTYCGNLVAFLTFPKFQPGIDYLNQLFDHKEIKQYGLRNGTFFEKYVHSTTRHDFKRFMERALVYNSSQSENIAAVKQGERINIDWRINLQLIVQQHFEQDKECRFALGKEDFVSEQIGLIVPSSSAYLHLINQHIDRLFRMGFIDRWHDTNLPSMDKCNGKHMQRQIANHKVNMDDMQGCFMVLLFGIIAALLVSCIEFWYYRFLVLNKGQSIAFAN

>CstyIR102

MNISQLWTSYQDRSGEQMNHINEFVARALLHVVHQHIMSVTPSLVLTLCCRNNHTCNFYNEMMSIMFRQWGISPLQIVNVEEGVPWRRIPGRRHFNVIFTDSFAAFAEIRVNEYSLEYNYNEYYFIFLQARDYLHHTEMMAIFQYCWHYQLINCNIQVQKSNGDILIYSYYPFSRENCNDIEPQLINRYNGSSFINPDLFPRKLINLYGCPLRAAIWNEPPFIWLTERENKSENFVSGGYEGNLLLSLAKKMNFTIAIRKPPNIKYRDEAMNMLLHNDADLSLGGFRQTVERTILATSTHNYHQTRQVFGLLDASFELGSFDILLYPYPLEIWLGILGVLGLALFIQLALDRLLHESHQDNWLNLELIFVGMPLLQTPRSSAKRMYCIMLMFYTLLIRTVYQGLLYHLIRTHQLYRLPQTIDELVGQNYTVVVTPTAQETLSGIPRVQHMNHRVIASTSETRPLEFLAEHPEYRRHTVATAEDVFIYHNRRNALKASGHFELVPQELINIQFTMYLPKHSYLVDHINEEIMWMRSVGLLVIWARWELDESYLKNIQSFKILNLTDLYAIFLLVAMGLILSTAVFTLEILSRHWLKLQNLFL

>CstyIR76b

MATGIELLVAAALCLACPPINDTLPADLIQLNEDGSLVTELPIDVAAANVGLDEDAPIETLEVIRNKKEKLRNMREWINGKHLRIATLEDYPLSYTEMLDNGTRVGRGVSFQIIGFLQEKFNFTYDVVVPQDNIIGSTTDLDRSLIEMVNNSLVDLAAAFIPSLSEQRTFVFYSTTTLDEGEWIMVMQRPRESAGGSGLLAPFEFWVWILIFVSLLAVGPIIYMLIIIRNRLTGDSVQQPYSLGHCAWFVYGALMKQGSTLSPIADSTRLLFATWWIFITILTSFYTANLTAFLTLSKFTLPFNTVNDILTKNKHFVSMRGGGVEYAIRTTNESLFMLNRMIQNNYAVFTDASNDTYNLQNYVERNGYVFVRDRPAINILLYRDYLYRKSVSFHDEKIHCPFAMAKEPFLTKKRTFAYPIGSNLSELFDPELLNLVESGIIKHLSARELPSAEICPQDLGSTERQLRNGDLMMTYYIMLTGFATSLVVFTTELLFRYINGRHEANKWARHGIGRTPNGQSVRPSRWLRGLRRLHSGDKQLLGSSTHAQNVTPPPPYQSIFNHGGEHLHGGQQRWRQAGREHGLILGGHHNGAGVRRLINGRDYMVFRNPNGTSQLVPVRSPSAALFQYTYTE

>CstyIR76a.2

MLLADKTWSLLLNIIIERYLHGTTICVLWHDDYEFQPQDLAVEYPSFIDINIATLASSFEDNVVDASTKRQQLKERDLVYDDLLLKLTLSIEMSHCESFLVFGEYIPRFVAAFTQAAMYSIWRSLHNKFIFAHVAQDLEEACCRDFFFQDQPNILFVVRSYANATDFELKTNKYVGPRSERPDQLELLDRYDALDQRFQHNSSLFPDKLRNLQGRELIINGFDYRPYTVIKYGSQSNAHDIGVPDDSELSKVYIDGTETRVVLSFCAQFNCTVQIDTSDAYDWGTIYPNMTGDGSLGMIIEHKVDICIGAMYSWYDDYTYLDLSMYLARSGITCLVPAPLRLASWYLPLQPFQGTLWAAVLFCLGIETLGLVLAHRREQELYANSDPKEGWWRSTKFGLVTTFKLFISQSGNSNARSVTVRVLLFACFLNDIIITSIYGGGLASILTIPSLGEAADTMQRMRSQKLQWAANSQAWVSSIRGSDDPLVHELLSNFHVYTDDQLLDLAQRDRMGFTVERLPFGHFAIGDYLTGQAIERMVIMQEDLYYEYTVAFVPRLWPLLERFNALVYRWHSSGFDKYWEYRVVADNLNVQIQQQVESTMSGGGQEDINIEPVTLAMSNFAGILLVWVLGVTVALMAFVAELMAAKVKRRRIMRF

>CstyIR92a

MLFQPLVMQLSPLLRIIIGQYFASFSSILIVHNNTRTTTLLQLEYLRSLELVLKGFGQSILLQWINVGQLSDLAELEWQVMSAVNSSTIEGFITILPQTNLFLHARYYATRNANVRLKDKRYLFLCEDEHPQELLAMDILQFYPHHLMVRPGTRAVTGPERGPATVPVPAHRHKEGTSVNTESTRDGSRLRDVDVDGDVRDGDGGAGATLPYRDINFELWTQKFVGAFGNLDAVRLGAFLPNETFAGNSRVELYPNKLLDLQQRTLRLGSITYVPYTITNYVPAGEGNEDPIHPHGANRSIAYYGAEANVMKTFCQVHHCHLRVEAYGADNWGSIYENESSDGMLGDIYEQRVELAIGCIYNWYDGITETSYHIARSSVTILGPAPAPLPSWRTNILPFNGATWLLLISTMVICGVFLYVMKFASFRLGYGRYEPEFHHAKKIEQSLLDTFALFIQQPSAPLSFDRFATRFFLATLLCATITLENIYSGQLKSLLTVPFYSAPVDTIEKWAQAGWKWAAPSIVWVHTVESSDLGIEQILARNFEVRDHSFLSNASFSPNYGLGIERLVSGSLSVGNYVPAEALENRIVLHDDLYFDWTRAVSIRGWTLMPELDRHILTCQETGLYVHWELEFVVKYMDKKVQEILLDMVNGPKAKGAPKPLNVSNISGALFVLAFGYTFAFSALLVETAICGLQKIK

>CstyIR64a

MVQFQFNFDSRKMSKPKRINNALDKRDNKYDYIKARPEKCHLHSAMYFWILLLLASSCVGLPADLDLDYGVAEPQRTALLQYSLMVQFALHHKRIPRLTYFTCRKPHSSGSELRGVFAAKNAQLIKSLYQSELFVRIVQLDVLAPNSGGGSSSGAGGGGATRGRGPSGGFSQTPSQAQSNAEWLDSVLRMEALRQIAVVDLACGSLSRRFLELASTKMLYSEKFHWLLIEDLAWQGGRSSSSSGKVQKDGSQDEAEEGPPGQQLQAPGNEELPTIESFMGGMNLYMNTELTLAKRMSEAAHYTLFDVWNPGLNYGGHVNLTEIGSFTPSGGIQLHAWFRGTSTVRRRMDMQHARVRCMVVVTNKNMTGTLMHYLTHTVSGHIDTMNRFNFNLLMAVRDMFNWTFVLSRTTSWGYVKNGRFDGMIGALIRNETDIGGAPIFYWLERHKWIDVAGRSWSSRPCFIFRHPRSTQKDRIVFLQPFTNDVWVLIVGCGVLTVFILWLLTTIEWKLVPHDGSALIKPKGGAPPRHHYQQKSHQDEVQNKEPLNATLDATLRPITAVSAVVDEEKSPEADPQPIDGETLWQRCYQKLNKYLKDRKAKEKKSPERVGLFLESVLFFVGIICQQGLGFSTSFVSGRCIVITSLLFSFCIYQFYSASIVGTLLMEKPKTIKTLSDLVHSSLKVGMEDILYNRDYFLHTKDPVSMELYSKKITSVPTTKENEADEDEPVNAAAPTADPAKSYRDIVHSHETGAHAKENSVSNWLDPETGLRRSLHLGFAFHVDVAAAYKIIAETFSEQDICDLTEVSMFPPQKTVSIMQKNSPMRKVISYGLRRVTETGILTYHFNVWHSRKPPCVKKIETSDLHVDMDTVSSALLILLFSYGITIMILSIEILYCKWQGRIIINFK

>CstyIR76a.1

MENLLVESYYFSTVLSFFAQQFFADSHATCIFWHPAFDFRLETVHPMPLIIMDWHRWANRSDQDVYDYKIKEDEFEGKGIPYNDWTLRLTVAIERSHCETFIAFQEQIPEFARYFYHASIYSIWRSLRNRFMFVYTKEFEDKKDSYLSGYIFQDQPNILVITSQYLNSSTFEIKTNRFVGPRNFNKNPEPVEFYILQRFDAKGTKATWETQSAMSSKMRNLKGREVVIGIFDYKPFMLLDYEKPPLYYDRFMNTTDVTIDGTDIQLMLIFCELYNCTIQVDTSEPYDWGDIYLNASGYGLVGMILDRRNDYGVGGMYLWYEAYEYMDMTHFLGRSGVTCLVPAPNRLISWTLLLRPFQFVLWMCVMLCLLLESLALGITRRWEHSSVAAGNSWISSLRFGCISTLKLFVNQSTNYVTSSYALRTVLVASYMIDIILTTVYSGGLAAILTLPTLEEAADSRQRLFDHKLIWTGTSQAWITTIDERSADPVLLGLMEHYRVYDANLISAFSHTEQMGFVVERLQFGHLGNTELIENDALKRLKLMVDDIYFAFTVAFVPRLWPHLNAYNDFILAWHSSGFDKFWEWKIAAEYMNAHRQNRIVASEKTNLDIGPVKLGIDNFIGLILLWCFGMICSLLTFLGELWRGQG

>CstyIR75d

MKVQVAHWLPLIFFLLVSGTPPRVAGSWRSEYSRQDPDPKTRWGNQLPDMLVAYYRHHGVHSLMLVVCHTDIADFRLWKLWQHFNLNNFYVQVSTESSLRDLQHVDALDEHKDAPPPKSFHANNSTHWETSFLLPALPYKMGILLLEFSSECALNLLRWSAASEHNYLTTNRFWLLLTEDPGDIDLLEDPEIFIPPDSELRVLQYENVGNFSCSLIDLYKVAAWKPLKRTLVGHNIRNSRHVIHALQHFGSAITYRQDLEGIVFNSAIVIAFPDLFTNIEDLSLRHIDTISKVNHRLMLELANRLNMSYNTYQTVNYGWRQPNGSFDGLMGRFQRYELDLAQLAIFMRLDRIALVDFVAETYRVRAGIMFRQPPLSAVANIFAMPFENDVWVSILMLLIITTVVLVLELFFSPHNHDMSYMDTLNFVWGAMCQQGFYVEVRNRSARIIVFTTFVAALFLFTSFSANIVALLQSPSDAIQSLSDLGQSPLEIGVQDTQYNKIYFTESTDPVTKNLYHKKIASKGENIYMRPLLGMEKMRTGLFAYQVELQAGYQIVSDTFSEPEKCGLMELEPFQLPMLAIPTRKNFPYKELIRRQLRWQREVSLVNREERKWIPQKPKCEGGVGGFVSIGITECRYALGIFGCGAAVSFVLFLFEFIFRHFKQVYRIIKGYREVQR

>CstyIR40a

MHYSLLFRLFLMVCLLGPTWAKLSYIPCNESDVAIALSQIINGLQPRQLAILVMPSSRLFGADCVNDDTIQVDEFIQRLHRLYYKSVIFYDTELFFDYIEANLLGAIECVNLIFHEPDELSARIQERRLAHRLSLFIFYWGARQPPNATRVSFEEPMRAVVITRPRRKAFRIYYNQALPTGTSQLRLVNWYDGDNLGLQKTPLLPSAATVYANLEGRVFRVPVFHSPPWFWVSYNNDSNNVGNSSEDPLDEYENLEYNEVNVTGGRDHRLLMLLAQHMNFQFMYIEAPGRTQGSLRAPDDSGESNDSFTGGIGMLQSGLADFFMGDVGLSWERRKAIEFSFFTLADSGAFATHAPRRLNEALAIMRPFKLDIWPYLILTIVFSGPIFYGIIAMPFKWRRRQLAVDVEHLGELCVYMAYIKEITPCVLKITHTRRPQAAPQMPPQLLQRCIWFTLRLFLKQSCHELYHDYRTKFLTIVYWVAATYVLADVYSAQLTSQFARPAHEAPINTLQRLQAAMLRDGYRLYVEKESSSLEMLENGTELFRQLYAQMRQQQPDDHLAFLIDSVESGIKLIADGRENKAVLAGRETLYFNIQQYGAKNFQLSQKLYTRYSAVAVQIGCPFLDSLNDVLMHLFEGGILEKMTTAEYEAQSRKMTKDQSQQSMELAAVENANANANTKSTEAKNQPQHAQDNEMISALNLRMLQGAFIALGVGFFTAALMLLLELFCRRLNLALPLRRCRLRWLRRWRRFKRMARHQTVRIFAPNLG

>CstyIR31a

MNLLISMFILILAAGEGEIIPSMEESVVTNFVKSLVKTKQAIVFSCLFKDFKEISLALMRINQFVSVVNLNQSYSLTSILTRENYARTSVMVNARCSGSSELLFEASENRYFNKTYQWFLWGVDLEVQSLFPLNLNYVGPNAQITYVNETADGYAYWDIHSKGRHLKSNLEINLIATLINDTLNIARDIFHLQSIDFRGQFNGLTLRGASVIDKEDIISNEQIESILSRPTKDAGVAAFIKYHYELLGLLRERFNFTVNFRNSRGWAGRLGNTTFRLGLLGIVMRNEADIAASGAFNRINRFAEFDTIHQSWKFETAFLYRYTSDLDTHGKSGNFLSPFSDRVWLFCLLTLGAFSIIWVLFEIIDYKILRIRVNSQKLEHLNQKSSVICIKTTCIERILQTFGACCQQGLDPNPVDRSVRFLVMTLFLFSLVMYNYYTSSVVGGLLSSSDQGPSTVDEITASPLKISFEDIGYYKVLFRESQNRSITRLIEKKLSSSRSLNELPIFSHIEDAVPYLKAGGFAFHCEVVDAYPVISEYFDANEICDLREVSGLMEVEILNWILHKNSQYTEIFKTAMCNAQEKGFVERILRRRQIKKPACQSLYTVYPVSLSGVLPGFVILICGFGASLLLLCLEKVYAHFGPRKFCGF

>CstyIR41a

MLLADKTWSLLLNIIIERYLHGTTICVLWHDDYEFQPQDLAVEYPSFIDINIATLASSFEDNVVDASTKRQQLKERDLVYDDLLLKLTLSIEMSHCESFLVFGEYIPRFVAAFTQAAMYSIWRSLHNKFIFAHVAQDLEEACCRDFFFQDQPNILFVVRSYANATDFELKTNKYVGPRSERPDQLELLDRYDALDQRFQHNSSLFPDKLRNLQGRELIINGFDYRPYTVIKYGSQSNAHDIGVPDDSELSKVYIDGTETRVVLSFCAQFNCTVQIDTSDAYDWGTIYPNMTGDGSLGMIIEHKVDICIGAMYSWYDDYTYLDLSMYLARSGITCLVPAPLRLASWYLPLQPFQGTLWAAVLFCLGIETLGLVLAHRREQELYANSDPKEGWWRSTKFGLVTTFKLFISQSGNSNARSVTVRVLLFACFLNDIIITSIYGGGLASILTIPSLGEAADTMQRMRSQKLQWAANSQAWVSSIRGSDDPLVHELLSNFHVYTDDQLLDLAQRDRMGFTVERLPFGHFAIGDYLTGQAIERMVIMQEDLYYEYTVAFVPRLWPLLERFNALVYRWHSSGFDKYWEYRVVADNLNVQIQQQVESTMSGGGQEDINIEPVTLAMSNFAGILLVWVLGVTVALMAFVAELMAAKVKRRRIMRF

>DmelGlu-R1

MHSRLKFLAYLHFICASSIFWPEFSSAQQQQQTVSLTEKIPLGAIFEQGTDDVQSAFKYAMLNHNLNVSSRRFELQAYVDVINTADAFKLSRLICNQFSRGVYSMLGAVSPDSFDTLHSYSNTFQMPFVTPWFPEKVLAPSSGLLDFAISMRPDYHQAIIDTIQYYGWQSIIYLYDSHDGLLRLQQIYQELKPGNETFRVQMVKRIANVTMAIEFLHTLEDLGRFSKKRIVLDCPAEMAKEIIVQHVRDIKLGRRTYHYLLSGLVMDNHWPSDVVEFGAINITGFRIVDSNRRAVRDFHDSRKRLEPSGQSQSQNAGGPNSLPAISAQAALMYDAVFVLVEAFNRILRKKPDQFRSNHLQRRSHGGSSSSSATGTNESSALLDCNTSKGWVTPWEQGEKISRVLRKVEIDGLSGEIRFDEDGRRINYTLHVVEMSVNSTLQQVAEWRDDAGLLPLHSHNYASSSRSASASTGDYDRNHTYIVSSLLEEPYLSLKQYTYGESLVGNDRFEGYCKDLADMLAAQLGIKYEIRLVQDGNYGAENQYAPGGWDGMVGELIRKEADIAISAMTITAERERVIDFSKPFMTLGISIMIKKPVKQTPGVFSFLNPLSQEIWISVILSYVGVSFVLYFVTRFPPYEWRIVRRPQADSTAQQPPGIIGGATLSEPQAHVPPVPPNEFTMLNSFWYSLAAFMQQGCDITPPSIAGRIAAAVWWFFTIILISSYTANLAAFLTVERMVAPIKTPEDLTMQTDVNYGTLLYGSTWEFFRRSQIGLHNKMWEYMNANQHHSVHTYDEGIRRVRQSKGKYALLVESPKNEYVNARPPCDTMKVGRNIDTKGFGVATPIGSPLRKRLNEAVLTLKENGELLRIRNKWWFDKTECNLDQETSTPNELSLSNVAGIYYILIGGLLLAVIVAIMEFFCRNKTPQLKSPGSNGSAGGVPGMLASSTYQRDSLSDAIMHSQAKLAMQASSEYDERLVGVELASNVRYQYSM

>DmelGlu-R1B

MRFGLKLSCLWPSFLLWLTWSSGGGGGSGVGVSAQPSLTEKIPLGAIFEQGTDEVQSAFKYAMLNHNLNVSSRRFELQAYVDVINTADAFKLSRLICNQFSRGVYSMLGAVSPDSFDTLHSYSNTFQMPFVTPWFPEKVLTPSSGFLDFALSMRPDYHQAIIDTIQFYGWRKIIYLYDSHDGLLRLQQIYQGLRPGNESFQVELVKRISNVSMAIEFLHTLEQIGRFENKHIVLDCPTEMAKQILIQHVRDLRLGRRTYHYLLSGLVMDDRWESEIIEFGAINITGFRIVDTNRRLVREFYDSWKRLDPQMSVGAGRESISAQAALMYDAVFVLVEAFNKILRKKPDQFRNNVQRRSQTLMVAQAAASTSSDGYNYSASGGGGGNGGAGGGFAGSDSGGSGGMASRALDCNTAKGWVNAWEHGDKISRYLRKVEIEGLTGDIKFNDDGRRVNYTLHVVEMTVNSAMVKVAEWNDDAGLQPLNAKYVRLRPHVEFEKNRTYIVTTVLEEPYIMLKQVAFGEKLHGNNRFEGYCKDLADLLAKELGINYELRLVKDGNYGSEKSSAHGGWDGMVGELVRKEADIAIAAMTITAERERVIDFSKPFMSLGISIMIKKPVKQTPGVFSFMNPLSQEIWVSVIFSYIGVSIVLFFVSRFSPHEWRLVQQQPQQSQSPDPHAHHEQLANQQPPGIIGGAPLPAPPGPPTPGAQTAAGAAALQAALSAGSPGSGGSSSAVVNEFSVWNSFWFSLAAFMQQGCDLSPRSVSGRIAAASWFFFTLILISSYTANLAAFLTVERMVTPINSPEDLAMQTEVQYGTLLHGSTWDFFRRSQIGLHNKMWEYMNSRKHVFVPTYDEGIKRVRNSKGKYALLVESPKNEYVNAREPCDTMKVGRNLDTKGFGIATPLGSALKDPINLAVLTLKENGELIKLRNKWWYEKAECSTHKDGETSHSELSLSNVAGIFYILIGGLLVSVFVAILEYCFRSRDSRSASSGSGMGLGMGLGGGMSGGSLGKANGSMMLGPSSAVPGGMPSSHQRSTLTDTMHAKAKLTIQASRDYDNGRVGYLNCASLQYYPPAQLSATPPDAGDSLHMNAHGQV

>DmelNmdar2

MMPSRVKLKRGTDGPTPTPTPMPTTMRKHTPIATLNTASCQHNSTTSRRKRILTPPSGPISLLLLTVLTLLILDTRSCQGLRLTNGGGSLSKGAAANKEQLNIGLIAPHTNFGKREYLRSINNAVTGLTKTRGAKLTFLKDYSFEQKNIHFDMMSLTPSPTAILSTLCKEFLRVNVSAILYMMNNEQFGHSTASAQYFLQLAGYLGIPVISWNADNSGLERRASQSTLQLQLAPSIEHQSAAMLSILERYKWHQFSVVTSQIAGHDDFVQAVRERVAEMQEHFKFTILNSIVVTRTSDLMELVNSEARVMLLYATQTEAITILRAAEEMKLTGENYVWVVSQSVIEKKDAHSQFPVGMLGVHFDTSSAALMNEISNAIKIYSYGVEAYLTDPANRDRRLTTQSLSCEDEGRGRWDNGEIFFKYLRNVSIEGDLNKPNIEFTADGDLRSAELKIMNLRPSANNKNLVWEEIGVWKSWETQKLDIRDIAWPGNSHAPPQGVPEKFHLKITFLEEAPYINLSPADPVSGKCLMDRGVLCRVAADHEMAADIDVGQAHRNESFYQCCSGFCIDLLEKFAEELGFTYELVRVEDGKWGTLENGKWNGLIADLVNRKTDMVLTSLMINTEREAVVDFSEPFMETGIAIVVAKRTGIISPTAFLEPFDTASWMLVGIVAIQAATFMIFLFEWLSPSGYDMKLYLQNTNVTPYRFSLFRTYWLVWAVLFQAAVHVDSPRGFTSRFMTNVWALFAVVFLAIYTANLAAFMITREEFHEFSGLNDSRLVHPFSHKPSFKFGTIPYSHTDSTIHKYFNVMHNYMRQYNKTSVADGVAAVLNGNLDSFIYDGTVLDYLVAQDEDCRLMTVGSWYAMTGYGLAFSRNSKYVQMFNKRLLEFRANGDLERLRRYWMTGTCRPGKQEHKSSDPLALEQFLSAFLLLMAGILLAALLLLLEHVYFKYIRKRLAKKDGGHCCALISLSMGKSLTFRGAVFEATEILKKHRCNDPICDTHLWKVKHELDMSRLRVRQLEKVMDKHGIKAPQLRLASSSDLLNHHHLKERPPLLGNLSLAASAQDLYRWSYKTEIAEMETVL

>DmelNmdar1

MAMAEFVFCRPLFGLAIVLLVAPIDAAQRHTASDNPSTYNIGGVLSNSDSEEHFSTTIKHLNFDQQYVPRKVTYYDKTIRMDKNPIKTVFNVCDKLIENRVYAVVVSHEQTSGDLSPAAVSYTSGFYSIPVIGISSRDAAFSDKNIHVSFLRTVPPYYHQADVWLEMLSHFAYTKVIIIHSSDTDGRAILGRFQTTSQTYYDDVDVRATVELIVEFEPKLESFTEHLIDMKTAQSRVYLMYASTEDAQVIFRDAGEYNMTGEGHVWIVTEQALFSNNTPDGVLGLQLEHAHSDKGHIRDSVYVLASAIKEMISNETIAEAPKDCGDSAVNWESGKRLFQYLKSRNITGETGQVAFDDNGDRIYAGYDVINIREQQKKHVVGKFSYDSMRAKMRMRINDSEIIWPGKQRRKPEGIMIPTHLRLLTIEEKPFVYVRRMGDDEFRCEPDERPCPLFNNSDATANEFCCRGYCIDLLIELSKRINFTYDLALSPDGQFGHYILRNNTGAMTLRKEWTGLIGELVNERADMIVAPLTINPERAEYIEFSKPFKYQGITILEKKPSRSSTLVSFLQPFSNTLWILVMVSVHVVALVLYLLDRFSPFGRFKLSHSDSNEEKALNLSSAVWFAWGVLLNSGIGEGTPRSFSARVLGMVWAGFAMIIVASYTANLAAFLVLERPKTKLSGINDARLRNTMENLTCATVKGSSVDMYFRRQVELSNMYRTMEANNYATAEQAIQDVKKGKLMAFIWDSSRLEYEASKDCELVTAGELFGRSGYGIGLQKGSPWTDAVTLAILEFHESGFMEKLDKQWIFHGHVQQNCELFEKTPNTLGLKNMAGVFILVGVGIAGGVGLIIIEVIYKKHQVKKQKRLDIARHAADKWRGTIEKRKTIRASLAMQRQYNVGLNSTHAPGTISLAVDKRRYPRLGQRLGPERAWPGDAADVLRIRRPYELGNPGQSPKVMAANQPGMPMPMLGKTRPQQSVLPPRYSPGYTSDVSHLVV

>DmelIR8a

MELPLLVLLLALRFAGSEVLKITFWIEPVQRAEFDTDIAMVLKELDALRLDVKVDDTTLTLTRSEDGLDMQRFCEILSTVGASAVIDLTYSHWEEGYNLVRSLGIGYVRLERIMRPFLDMFGDFMRQKRANNVAMVFMNARDAVEAMQQMLVGYPFRTLIMDASQTDPGQHFLERIRSLRPAPTYIALFARAAAMNGIFEKVQKADLFQRPLEWHFVFLDTRDRVFKYRRQAELCTRFTLNPRAICRSMPMPDLYCGSGFTMQRAMLLNVLRSLINAAQVSPGYPLAIYQDCNATASSSEVSDPLEKDDYNWLDMVHWSNFLAYAPPLPHIQDQFQSPVPGLTFAVNISAGYYSSEHEAKTDLAAWSSVGEMRLLNETISPARRFFRIGTAESIPWSYLRREEGTGELIRDRSGLPIWEGYCIDFIIRLSQKLNFEFEIVAPEVGHMGELNELGEWDGVVGDLVRGETDFAIAALKMYSEREEVIDFLPPYYEQTGISIAIRKPVRRTSLFKFMTVLRLEVWLSIVAALVGTAIMIWFMDKYSPYSSRNNRQAYPYACREFTLRESFWFALTSFTPQGGGEAPKAISGRMLVAAYWLFVVLMLATFTANLAAFLTVERMQTPVQSLEQLARQSRINYTVVKDSDTHQYFVNMKFAEDTLYRMWKELALNASKDFKKFRIWDYPIKEQYGHILLAINSSQPVADAKEGFANVDAHENADYAFIHDSAEIKYEITRNCNLTEVGEVFAEQPYAVAVQQGSHLGDELSYAILELQKDRFFEELKAKYWNQSNLPNCPLSEDQEGITLESLGGVFIATLFGLVLAMMTLGMEVLYYKKKQNALEITQVRPVNDSSGSGGNSSTAPPTATSTTKQAWHIPVLEAEEKPAKVSPPPSFETATFRGKKLPARITLGDGKFKPRHGLYARRNLGASDSHSGYME

>DmelIR25a

MILMNPKTSKILWLLGFLSLLSSFSLEIAAQTTQNINVLFINEVDNEPAAKAVEVVLTYLKKNIRYGLSVQLDSIEANKSDAKVLLEAICNKYATSIEKKQTPHLILDTTKSGIASETVKSFTQALGLPTISASYGQQGDLRQWRDLDEAKQKYLLQVMPPADIIPEAIRSIVIHMNITNAAILYDDSFVMDHKYKSLLQNIQTRHVITAIAKDGKREREEQIEKLRNLDINNFFILGTLQSIRMVLESVKPAYFERNFAWHAITQNEGEISSQRDNATIMFMKPMAYTQYRDRLGLLRTTYNLNEEPQLSSAFYFDLALRSFLTIKEMLQSGAWPKDMEYLNCDDFQGGNTPQRNLDLRDYFTKITEPTSYGTFDLVTQSTQPFNGHSFMKFEMDINVLQIRGGSSVNSKSIGKWISGLNSELIVKDEEQMKNLTADTVYRIFTVVQAPFIMRDETAPKGYKGYCIDLINEIAAIVHFDYTIQEVEDGKFGNMDENGQWNGIVKKLMDKQADIGLGSMSVMAEREIVIDFTVPYYDLVGITIMMQRPSSPSSLFKFLTVLETNVWLCILAAYFFTSFLMWIFDRWSPYSYQNNREKYKDDEEKREFNLKECLWFCMTSLTPQGGGEAPKNLSGRLVAATWWLFGFIIIASYTANLAAFLTVSRLDTPVESLDDLAKQYKILYAPLNGSSAMTYFERMSNIEQMFYEIWKDLSLNDSLTAVERSKLAVWDYPVSDKYTKMWQAMQEAKLPATLDEAVARVRNSTAATGFAFLGDATDIRYLQLTNCDLQVVGEEFSRKPYAIAVQQGSHLKDQFNNAILTLLNKRQLEKLKEKWWKNDEALAKCDKPEDQSDGISIQNIGGVFIVIFVGIGMACITLVFEYWWYRYRKNPRIIDVAEANAERSNAADHPGKLVDGVILGHSGEKFEKSKAALRPRFNQYPATFKPRF

>DmelIR21a

MSYYWVALVLFTAQAFSIEGDRSASYQEKCISRRLINHYQLNKEIFGVGMCDGNNENEFRQKRRIVPTFQGNPRPRGELLASKFHVNSYNFEQTNSLVGLVNKIAQEYLNKCPPVIYYDSFVEKSDGLILENLFKTIPITFYHGEINADYEAKNKRFTSHIDCNCKSYILFLSDPLMTRKILGPQTESRVVLVSRSTQWRLRDFLSSELSSNIVNLLVIGESLMADPMRERPYVLYTHKLYADGLGSNTPVVLTSWIKGALSRPHINLFPSKFQFGFAGHRFQISAANQPPFIFRIRTLDSSGMGQLRWDGVEFRLLTMISKRLNFSIDITETPTRSNTRGVVDTIQEQIIERTVDIGMSGIYITQERLMDSAMSVGHSPDCAAFITLASKALPKYRAIMGPFQWPVWVALICVYLGGIFPIVFTDRLTLSHLMGNWGEVENMFWYVFGMFTNAFSFTGKYSWSNTRKNSTRLLIGAYWLFTIIITSCYTGSIIAFVTLPAFPDTVDSVLDLLGLFFRVGTLNNGGWETWFQNSTHIPTSRLYKKMEFVGSVDEGIGNVTQSFFWNYAFLGSKAQLEYLVQSNFSDENISRRSALHLSEECFALFQIGFLFPRESVYKIKIDSMILLAQQSGLIAKINNEVSWVMQRSSSGRLLQASSSNSLREIIQEERQLTTADTEGMFLLMALGYFLGATALVSEIVGGITNKCRQIIKRSRKSAASSWSSASSGSMLRTNAEQLSHDKRKANRREAAEVAQKMSFGMRELNLTRATLREIYGSYGAPETDHGQLDIVHTEFPNSSAKLNNIEDEESREALESLQRLDEFMDQMDNDGNPSSHTFRIDN

>DmelIR31a

MNLLISMFILILAAGEGEIIPSMEESVVTNFVKSLVKTKQAIVFSCLFKDFKEISLALMRINQFVSVVNLNQSYSLTSILTRENYARTSVMVNARCSGSSELLFEASENRYFNKTYQWFLWGVDLEVQSLFPLNLNYVGPNAQITYVNETADGYAYWDIHSKGRHLKSNLEINLIATLINDTLNIARDIFHLQSIDFRGQFNGLTLRGASVIDKEDIISNEQIESILSRPTKDAGVAAFIKYHYELLGLLRERFNFTVNFRNSRGWAGRLGNTTFRLGLLGIVMRNEADIAASGAFNRINRFAEFDTIHQSWKFETAFLYRYTSDLDTHGKSGNFLSPFSDRVWLFCLLTLGAFSIIWVLFEIIDYKILRIRVNSQKLEHLNQKSSVICIKTTCIERILQTFGACCQQGLDPNPVDRSVRFLVMTLFLFSLVMYNYYTSSVVGGLLSSSDQGPSTVDEITASPLKISFEDIGYYKVLFRESQNRSITRLIEKKLSSSRSLNELPIFSHIEDAVPYLKAGGFAFHCEVVDAYPVISEYFDANEICDLREVSGLMEVEILNWILHKNSQYTEIFKTAMCNAQEKGFVERILRRRQIKKPACQSLYTVYPVSLSGVLPGFVILICKSINKFS

>DmelIR40a

MHKFLALGLLPYLLGLLNSTRLTFIGNDESDTAIALTQIVRGLQQSSLAILALPSLALSDGVCQKERNVYLDDFLQRLHRSNYKSVVFSQTELFFQHIEENLQGANECISLILDEPNQLLNSLHDRHLGHRLSLFIFYWGARWPPSSRVIRFREPLRVVVVTRPRKKAFRIYYNQARPCSDSQLQLVNWYDGDNLGLQRIPLLPTALSVYANFKGRTFRVPVFHSPPWFWVTYCNNSFEEDEEFNSLDSIEKRKVRVTGGRDHRLLMLLSKHMNFRFKYIEAPGRTQGSMRSEDGKDSNDSFTGGIGLLQSGQQADFFLGDVGLSWERRKAIEFSFFTLADSGAFATHAPRRLNEALAIMRPFKQDIWPHLILTIIFSGPIFYGIIALPYIWRRRWANSDVEHLGELYIHMTYLKEITPRLLKLKPRTVLSAHQMPHQLFQKCIWFTLRLFLKQSCNELHNGYRAKFLTIVYWIAATYVLADVYSAQLTSQFARPAREPPINTLQRLQAAMIHDGYRLYVEKESSSLEMLENGTELFRQLYALMRQQVINDPQGFFIDSVEAGIKLIAEGGEDKAVLGGRETLFFNVQQYGSNNFQLSQKLYTRYSAVAVQIGCPFLGSLNNVLMQLFESGILDKMTAAEYAKQYQEVEATRIYKGSVQAKNSEAYSRTESYDSTVISPLNLRMLQGAFIALGVGSLAAAALNNTINVRSLNSRDKFICGGPVKIWYYLVLLLWYYFNRGLVGIYQLWHKTSIRNTGKGMPFLGE

>DmelIR64a

MHWWLLVFLPLSCQGLPEHELLELELDYGLAEPQRTSLLQSSLILQFSQDYKHIPRITYFTCQKPHLQTPNQIPNAAEHRDAFAAKNFQLIKSLYESELFVRIVLLDVLAQSPTSGRPNRPGNGPTGGFSQTPSQAQSNSEWLEGVLRMEALRQIAVVDLACGAVSRRFLELASAKMLYSEKFHWLLIEDFAWHGRTQTAEGSGKRDDGEMEEEEPPGQQIQATDDEDLPSIESFLGGMNLYMNTELTLAKRMSEAAHYTLFDVWNPGLNYGGHVNLTEIGSFTPTEGIQLHTWFRTTSTVRRRMDMQHARVRCMVVVTNKNMTGTLMYYLTHTMSGHIDTMNRFNFNLLMAVRDMFNWTFVLSRTTSWGYVKNGRFDGMIGALIRNETDIGGAPIFYWLERHKWIDVAGRSWSSRPCFIFRHPRSTQKDRIVFLQPFTNDVWILIVGCGVLTVFILWFLTTIEWKLVPHDGSALIKPKGGAPPRHHYQQQQQQEQVEAPVRPITAVSVVVSKEKVEEKQEEYEDSTPIDAGTLWQRCYQKLNKYIKDRKAKQKKAPERVGLFLESVLFFVGIICQQGLGFSTSFVSGRCIVITSLLFSFCIYQFYSASIVGTLLMEKPKTIKTLSDLVHSSLKVGMEDILYNRDYFLHTKDPVSMELYAKKITSVPTTKENEADEDEPVDPNPVSTDPAKSYRDIVHSHETGAHAKDNAASNWLDPETGLLRVKHERFAFHVDVAAAYKIIAETFSEQDICDLTEVSMFPPQKTVSIMQKNSPMRKVISYGLRRVTETGILTYHFNVWHSRKPPCVKKIETSDLHVDMDTVSSALLILLFSYAITLMILGTEILYSKWHNRIQLKWVGAT

>DmelIR75a

MQLVQLANFVLDNLVQSRIGFIVLFHCWQSDESLKFAQQFMKPIHPILVYHQFVQMRGVLNWSHLELSYMGHTQPTLAIYVDIKCDQTQDLLEEASREQIYNQHYHWLLVGNQSKLEFYDLFGLFNISIDADVSYVKEQIQDNNDSVAYAVHDVYNNGKIIGGQLNVTGSHEMSCDPFVCRRTRHLSSLQKRSKYGNREQLTDVVLRVATVVTQRPLTLSDDELIRFLSQENDTHIDSLARFGFHLTLILRDLLHCKMKFIFSDSWSKSDVVGGSVGAVVDQTADLTATPSLATEGRLKYLSAIIETGFFRSVCIFRTPHNAGLRGDVFLQPFSPLVWYLFGGVLSLIGVLLWITFYMECKRMQKRWRLDYLPSLLSTFLISFGAACIQSSSLIPRSAGGRLIYFALFLISFIMYNYYTSVVVSSLLSSPVKSKIKTMRQLAESSLTVGLEPLPFTKSYLNYSRLPEIHLFIKRKIESQTQNPELWLPAEQGVLRVRDNPGYVYVFETSSGYAYVERYFTAQEICDLNEVLFRPEQLFYTHLHRNSTYKELFRLRFLRILETGVYRKQRSYWVHMKLHCVAQNFVITVGMEYVAPLLLMLICADILVVVILLVELAWKRFFTRHLTFHP

>DmelIR75b

MNFSVLESHFKEAQIFVDADVTYVTHDPFSKNFLLYDVYNKGRQLGGELNITADREIFCNKTNCRVERYLSELYTRSALQHRKSFTGLTMRATAVVTALPLNVSIKEIFDFMNSKYRIQLDTYARLGYQARQPLRDMLDCKFKYIFRDRWSDGNATGGMIGDLILDKADLAIAPFIYSFDRALFLQPITKFSVFREICMFRNPRSVSAGLSATEFLQPFSGGVWLTFALLLLLAGCLLWVTFILERRKQWKPSLLTSCLLSFGAGCIQGAWLTPRSMGGRMAFFALMVTSYLMYNYYTSIVVSKLLGQPIKSNIRTLQQLADSNLDVGIEPTVYTRIYVETSEEPDVRDLYRKKVLGSKRSPDKIWIPTEAGVLSVRDQEGFVYITGVATGYEFVRKHFLAHQICELNEIPLRDASHTHTVLAKRSPYAELIKLSELRMLETGVHFKHERSWMETKLHCYQHNHTVAVGLEYAAPLFIILLGAIILCMGILGLEVIWHRHCTLH

>DmelIR75c

MTSWPLYRLIVFNLLEINLSNLMVFHCWSIKEAFPLVEMLNQNGIFSQYIDVQNPDNLANVHKEYLDSDLVSLNADVTYVSREDEERFILHDVYNKGSHLGGKLNITVDQTLQCNRSHCQVKEYLSELHLRPRLQHRMDLSSVTFRLAALVSVLPINSSEEELLEFLNSDRDSHMDSISRIGNRLIMHTQEILGFNVQDAFGGAIGMLTNESAELCTTPFVPSWNRLHYLHPMTEQAQFRAVCMFRTPHNAGIKAAVFLEPFMPSVWFAFAGLLIFAGVLLWMIFHLERHWMQRCLDFIPSLLSSCLISFGAACIQGSYLMPKSAGGRLAFIAVMLTSFLMYNYYTSIVVSTLLGSPVRSNIRTIQQLADSSLDVGFDTVPFTKTYLVSSPRPDIRSLYKQKVESKRDPNSVWLSPEEGVIRVRDQPGFVYTSEASFMYHFVEKHYLPREISDLNEIILRPESAVYGMVHLNSTYRQLLTQLQVRMLETGITSKQSRFFSKTKLHTFSNSFVIQVGMEYAAPLFISLLVAYFLALLILILEICWARYAKKKFSTIIPQNQ

>DmelIR75d

MKVQVAHWLPLIFFLLVSGTPRVAGSWRSEYSRQDPDPKTRWGNQLPDMLVAYYRHHGVHSLMLVVCHTDIADFRLWKLWQHFNLNNFYVQVSTESSLRDLQHVDALDEHKDAPPPKSFHANNSTHWETSFLLPALPYKMGILLLEFSSECALNLLRWSAASEHNYFTTNRFWLLLTEDPGDIDLLEDPEIFIPPDSELRVLHYENVGNFSCSLIDLYKVAAWKPLKRTLVGHNIRNSRHVIHALQHFGSAITYRQDLEGIVFNSAIVIAFPDLFTNIEDLSLRHIDTISKVNHRLMLELANRLNMSYNTYQTVNYGWRQPNGSFDGLMGRFQRYELDLAQLAIFMRLDRIALVDFVAETYRVRAGIMFRQPPLSAVANIFAMPFENDVWVSILMLLIITTVVLVLELFFSPHNHDMSYMDTLNFVWGAMCQQGFYVEVRNRSARIIVFTTFVAALFLFTSFSANIVALLQSPSDAIQSLSDLGQSPLEIGVQDTQYNKIYFTESTDPVTKNLYHKKIASKGENIYMRPLLGMEKMRTGLFAYQVELQAGYQIVSDTFSEPEKCGLMELEPFQLPMLAIPTRKNFPYKELIRRQLRWQREVSLVNREERKWIPQKPKCEGGVGGFVSIGITECRYALGIFGCGAAVSFVLFLFEFIFRHFKQVYRIIKGYREVQR

>DmelIR76a

MENLLVESYYFSTVLSFFAQQFFADSHATCIFWHPAFDFRLETVHPMPLIIMDWHRWANRSDQDVYDYKIKEDEFEGKGIPYNDWTLRLTVAIERSHCETFIAFQEQIPEFARYFYHASIYSIWRSLRNRFMFVYTKEFEDKKDSYLSGYIFQDQPNILVITSQYLNSSTFEIKTNRFVGPRNFNKNPEPVEFYILQRFDAKGTKATWETQSAMSSKMRNLKGREVVIGIFDYKPFMLLDYEKPPLYYDRFMNTTDVTIDGTDIQLMLIFCELYNCTIQVDTSEPYDWGDIYLNASGYGLVGMILDRRNDYGVGGMYLWYEAYEYMDMTHFLGRSGVTCLVPAPNRLISWTLLLRPFQFVLWMCVMLCLLLESLALGITRRWEHSSVAAGNSWISSLRFGCISTLKLFVNQSTNYVTSSYALRTVLVASYMIDIILTTVYSGGLAAILTLPTLEEAADSRQRLFDHKLIWTGTSQAWITTIDERSADPVLLGLMEHYRVYDANLISAFSHTEQMGFVVERLQFGHLGNTELIENDALKRLKLMVDDIYFAFTVAFVPRLWPHLNAYNDFILAWHSSGFDKFWEWKIAAEYMNAHRQNRIVASEKTNLDIGPVKLGIDNFIGLILLWCFGMICSLLTFLGELWRGQG

>DmelIR76b

MATGIELLVAAALCVACPPLNDSPPTNLIQMGENGTLSPVTELPMDVDASEAGFDADAPVETLETINRKKPKLREMLDWIGGKHLRIATLEDFPLSYTEVLENGTRVGHGVSFQIIDFLKKKFNFTYEVVVPQDNIIGSPSDFDRSLIEMVNSSTVDLAAAFIPSLSDQRSFVYYSTTTLDEGEWIMVMQRPRESASGSGLLAPFEFWVWILILVSLLAVGPIIYALIILRNRLTGDGQQTPYSLGHCAWFVYGALMKQGSTLSPIADSTRLLFATWWIFITILTSFYTANLTAFLTLSKFTLPYNTVNDILTKNKHFVSMRGGGVEYAIRTTNESLSMLNRMIQNNYAVFSDETNDTYNLQNYVEKNGYVFVRDRPAINIMLYRDYLYRKTVSFSDEKVHCPFAMAKEPFLKKKRTFAYPIGSNLSQLFDPELLHLVESGIVKHLSKRNLPSAEICPQDLGGTERQLRNGDLMMTYYIMLAGFATALAVFSTELMFRYVNSRQEANKWARHGIGRTPNGQSVAPSRWLRGWRRLNSGHGQLLGASTHGQNVTPPPPYQSIFNGGSHGDPLNRWRRPLANGNALGNGVLLGGDSEGGVRRLINGRDYMVFRNPNGQSQLVPVRSPSAALFQYSYTE

>DmelIR84a

MIKLQVKVISWPLIILTAFLRVLQIESINTNFLELAAFEDFLRSEHLSHVLVVRGDDADGDWKIECHQKLLANYRVQFYRPEMSANFEDLMFYGSPRTAVLVLNSEHVLVRRQVFGVASEAGYFNNSLAWFILGSGRESLPVEQLIDQLLSGYRMGIDADITVALRGPDNASMLFYDVYRISRQANTPLIIEKKGLWTHSGGYQKFGNFKNTWVIRRRNFLNVTLIGSTVLTEKPPGFGDMEYLADDKQLQQLDPMQRKTYQLFQLVERMFNLSLAISLTDKWGELLDNGSWSGVMGQVTSREADFAVCPIRFVLDRQPYVQYSAVLHTQNIHFLFRHPRRSHIKNIFFEPLSNQVWWCVLALVTGSTILLLFHVRLERMLSNMENRFSFVWFTMLETYLQQGPANEIFRLFSTRLLISLSCIFSFMLMQFYGAFIVGSLLSESARSIVNLQALYDSNLAIGMENISYNFPIFTNTSNQLVRDVYVKKICKSGEHNIMSLQQGAERIIQGRFAFHTAIDRMYRLLLELQMDEAEFCDLQEVMFNLPYDSGSVMPKGSPWREHLAHALLHFRATGLLQYNDKKWMVRRPDCSLFKTSQAEVDLEHFAPALFALALAMVASALVFLLELFLHWLPDFRRRLGTMST

>DmelIR92a

MLLQPLVMHLSQLLRIIVGQYFAEFPSILIVYNNSASTTPLQLEYLSALELVLRELSKPIRLQWINVAFLKDLNDLEDQVMGALNSSVTEGFITILSQTHHFIHARYYATRNANVRLKDKRYLFLCEDESPAELLCMDILQFYPHHLMVRPGTETAPTGPTGPHPDPRRGGGASVSTKNKDDGEGGAGNKTTSPYRDINFELWTQKFVGAVGNLDALLLDAFLPNETFANRVELYPNKLLNLQRRSLLVGSITYVPYTITNYVPAGQGDVDPIHPQWPNRSLTFDGAEANVMKTFCQVHNCHLRVEAYGADNWGGIYDNESSDGMLGDIYEQRVEMAIGCIYNWYDGITETSHTIARSSVTILGPAPAPLPSWRTNIMPFNNRAWLVLISTLVICGTFLYFMKYVSYRLRYSGTQVKFHHSRKLEKSMLDIFALFIQQPSAPLSFDRFAPRFFLATILCATITLENIYSGQLKSMLTFPFYSAPVDTIEKWAQSGWKWSAPSIIWVHTVQSSDLETEQILARNFEVHDYSYLSNVSFMPNYGFGIERLSSGSLSVGDYVSTEALENRIVLHDDLYFDYTRAVSIRGWILMPELNKHIRTCQETGLYFHWELEFIDKYMDKKKQEVLMDLANGHKVKGAPQALDVRNIAGALFVLAFGVAFAGCALVAELLIHRMDLSK

>DmelIR93a

MNPGEMRPSACLLLLAGLQLSILVPTEANDFSSFLSANASLAVVVDHEYMTVHGENILAHFEKILSDVIRENLRNGGINVKYFSWNAVRLKKDFLAAITVTDCENTWNFYKNTQETSILLIAITDSDCPRLPLNRALMTVECRINAVVFVDQTILEENALLVKSIVHESITNHITPISLILYEINDSLRGQQKRVALRQALSQFAPKKHEEMRQQFLVISAFHEDIIEIAETLNMFHVGNQWMIFVLDMVARDFDAGTVTINLDEGANIAFALNETDPNCQDSLNCTISEISLALVNAISKITVEEESIYGEISDEEWEAIRFTKQEKQAEILEYMKEFLKTNAKCSSCARWRVETAITWGKSQENRKFRSTPQRDAKNRNFEFINIGYWTPVLGFVCQELAFPHIEHHFRNITMDILTVHNPPWQILTKNSNGVIVEHKGIVMEIVKELSRALNFSYYLHEASAWKEEDSLSTSAGGNESDELVGSMTFRIPYRVVEMVQGNQFFIAAVAATVEDPDQKPFNYTQPISVQKYSFITRKPDEVSRIYLFTAPFTVETWFCLMGIILLTAPTLYAINRLAPLKEMRIVGLSTVKSCFWYIFGALLQQGGMYLPTADSGRLVVGFWWIVVIVLVTTYCGNLVAFLTFPKFQPGVDYLNQLEDHKDIVQYGLRNGTFFERYVQSTTREDFKHYLERAKIYGSAQEEDIEAVKRGERINIDWRINLQLIVQRHFEREKECHFALGRESFVDEQIAMIVPAQSAYLHLVNRHIKSMFRMGFIERWHQMNLPSAGKCNGKSAQRQVTNHKVNMDDMQGCFLVLLLGFTLALLIVCGEFWYRRFRASRKRRQFTN

>DmelIR7a

MFHHLWLLMGLRSLAMGALHPPQPEAMTPLVAAALEILAEQVSPSQSTLAVMDLTQDAEHRDERQEQLMTIILRSVGSEMALRTFQKPPAEVPASFVVFLVNSAQAFNTLGFHFTDIHSTREFNFLILLTHRMSSRAERLQVLRDISRTCVRFHTSNVILLTEKRDGVVLVYAYRLLNMDCDLSVNLELIDIYKNGLFRHGHEARSFNRVLSLSGCPLQVSWYPLPPFVSFIGNSSDPEERAQIWRLTGIDGELIKLLASIFDFRILLEEPCNKCLSPDIKDDCSGCFDQVIISNSSILIGAMSGSHQHRSHFSFTSSYHQSSLVFIMHMSSQFGAVAQLAVPFTVIVWLALVVSSLLLVLVLWMRNRLVCGRSDLASHALQVLTTLMGNPLEARSLPRSSRLRILYAGWLLLVLVLRVVYQGKLFDSFRLPYHKPLPTEISELIRSNYTLINQEYLDYYPRELTVLTRNGSKDRFDYIQGLGKEGKFTTTSLIATMEYYNMMHWSTSRLTHIKEHIFLYQMVIYLRRHSLLKFAFDRKIKQLLSAGIIGYFVREFDACQYRKPFEEDYEVTPIPLDSFCGLYYISLIWLSAAVVAFILELLSQRIVWLRRIFE

>DmelIR7b

MKYWLYILSCCSLVASTMESSSDWDLAEALAQVVANSEMGRFKTLYIYTHTNSQSTGGHLEELLDQVLMIVPNNLQARRLLLQQSMEYKPYVHAVLALVDGLPSLSAIYARIRATQDLSHTLIYMSMPTDAYGEEMQATLRFLWRLSVLNVGVVLRPPGDHILMVSYFPFSALHGCQVISANVVNRYQVGTKRWASQDYFPSKLGNFYGCLLTCATWEDMPYLVWRPDGSGSFVGIEGALLQFMAENLNFTVGLYWMNKEEVLATFDESGRIFDEIFGHHADFSLGGFHFKPSAGSEIPYSQSTYYFMSHIMLVTNLQSAYSAYEKLSFPFTPLLWRAIGLVLILACLLLMLLVRWRHHHELPRNPYYELLVLTMGGNLEDRWVPQRFPSRLVLLTWLFATLVLRSGYQSGMYQLLRQDTQRNPPQTISEVLAQHFTIQLAEVNEARILASLPELRPEQLVYLEGSELQSFPALAQQSGSSARVAILTPYEYFGYFRKVHPMSRRLHLVRERIYTQQLAFYVRRHSHLVGVLNKQIQHAHTHGFLEHWTRQYVSAVDEKDESVARIASTSYSTLDGIDGDPSLSESEEDQQVAPVRQNVLSMRELAALFWLILWANLGAVVVFVLELLLPRIKLRKILRKMKKSTRASATTTSTLSSPSTTKDIPFSCKDGFQDSWPKCSLLVS

>DmelIR7c

MLHSAVHNVSLVYALVWAIDNYYGMATSTPLAVVQFPTSRESRRLHNDLIDAALGRSSGTGRIQFLLEDDRVEMTETDTDPPPPSGLTGRPIAIWFLDSLRSYFRLEMYLNQLGSPYKRNGFFLVIYTGLEDQPMESLKIMFRRLLNMYVLNVNVFLQRDGTVHLYTYYPYGPHHCQSSLPVYYTAFQDLAAPANGFGLTKPLFPRKLTNMHGCEMVVATFEHRPYVIIEDDPKTPGGRSIHGIEGLIFRSLAERMNFTIKLVEQKDKNRGEILPDGNFTGILKMMVDGEVNLTFVCFMYSKARSDLMLPSTSYTSFPIVLVVPSGGSISPMGRLTRPFRYIIWSCILVSLIFGFVLICLLKITALPGLRNLVLGRRNRLPFMGMWASLLGGLALYNPQRNFARYILVMWLLQTLILRAAYTGQLYLLLQDVEMRSPIKSLSEVLAKDYEFRILPALRTIFKDSMPTTNFHAVLSLEESLYRLRDEDDPGITVALLQPTVNQFDFRSGPNKRHLTVLPDPLMTAPLTFYMRPHSYFKRRIDRLIMAMMSSGIVARYRKMYMDRIKRVSKRRNLEPKPLSIWRLSGIFVCCAGLYLVALIVFILEILTTNHRRLRRAFNVINRYAA

>DmelIR7d

MDIRCVVALLLGLCKVQAVVWPHQHLLEEQLASQISATLQKIFINGLAVYNFGVFISTSYEEMDRDRVILVHQVLNRNLYPPNFPVAVVLASKMNRKITAQVFTQLLFVQNAEQAIAIAEGVNRNGLCVIVLLTSQPERPIMTKIFTYFMQERYNINVVILVPRLHGVQAFNVRPYTPTSCSSLEPVEIDIKDGDLWDVFPRRLKNLHGCPLSVIVWDIPPYMRINWKSSDPMDGLDGLDGLLLRIVARKMNFTLKLIPNEPNGLIGGSSFMNGTFTGAYKMLRERRANITIGCAACTPERSTFLEATSPYSQMSYIIVLQARGGYSIYEVMLFPFEKYTWLLLSTILGLHWIVGSRWRMPSPILAGWMLWIFVIRASYEASVFNFIQNSPVKPSPRTLDQALSGGFRFITDHASYRMTLKIPSFQGKTLISAGQPVDVFDALLKAPWKTGAFTSRAFLADHLVRHRKHRNQLVILAEKIVDNMLCMYFPHGSYFAWEINKLLFNMRSFGIFQHHSQILAWDNLPTTTDTDTPGKRIHSSTESVATGFAESMSFVVAALNCLMGALCISIVVFGLELLSRRRHWTGLEWLFERV

>DmelIR7e

MNHINEFVARAVLHVVHHYILSVTPSLVLTLCCRSNHTCNFYNKMMSTLFREWGLAPLQIVNVLRGVPWHPVPGRRHFNVIFTDSFAAFEEIRMEYYSREYNYNEHYFIFLQARDRLLQGEMRLIFDYCWRYRLIHCSIQVQKSNGDILFYSYYPFGEHGCSDMEPQLINRYNGSMLVEPDLFPRKLRNFFGCPLRCALWDVPPFLTLDEDQEEVLRVNGGYEGRLLLALAEKMNFTIAVRKVHVNMRDEALEMLRRDEVDLTLGGIRQTVARGMVATSSHNYHQTREVFGVLASSYELSSFDILFYPYRLQIWMGILGVVALSALIQLIVGRMLRERMGSRFWLNLELVFVGMPLLECPRSHTARLYCVMLMMYTLIIRTIYQGLLYHLIRTHQLNRWPQTIESLVQKNFTVVLTPIVQEVLDEIPSVQHMRFRLLEANSELDPLYFLEANHQLRQHVTASALDIFIHFNRLSADKVHQRGEQGSGAHFEIVPEDIISMQLTMYLAKHSFLIDQLNEEIMWMRSVGLLSVWSRWELSESYLRNEQSFQVLGTMELYAIFLMVLVGLIVGLLVFILELVSMRSIYLRKLFT

>DmelIR7f

MQGEDANLYVARALRLVIENVLAQLSTTLVVTISTRHLGTAHWFEYMMNILMDSWRMVAVQLLRIRPDLVVNPVPGRKRVSLLMVDSYQGLLDTNITASNANFDDPDYYFIFLQARDHLIPKELQLILDHCLAHFWLHCNVMIQTAQVEVLVYTYYPYTADACQKAYPIPVNTFDGRKWKASQMFPDKLSQMHGCPLTVLTWHQPPFVELVWDPKHNRSRGSGFEIQLVEHLARRMNFSLELVNIALLRPNAYRLAEGSSEGPIEKLLQRNVNISMGYFRKTARRNQLLTTPMSYYSANLVAVLQLERYRIGSLALLVFPFELSVWMLLLLALLIHLGIHLPSARRGNEEDGGGGLQVVALLLGAALARLPRSWRHRFIAAHWLWASIPLRISYQSLLFHLIRLQLYNTPSFSLDQLLAEGFQGICTANTQRLLLEMPQLARDPDSIQSVDTPFDWDVLNVLTRNRNRKIFAVANQDVTLSFLHSSAHPNAFHVVKQPVNVEYAGMYMPKHSFLYEKMDDDIRRLDASGFIHAWRRASFASVHRKEQVHMTSRRYINHAKLSGIYMVMAGLYLLAGLLFAGEVLLRQRN

>DmelIR7g

MNVTSLLNFESMKYIGAQTQAASINHHVAQALRVFIEDFYQRIAPAFIVVLSCRRPSPMNFYRNIMQLLYESVDTMIVQLVLVELGRPRRIAGPRTHNLLLVDSLDALLDIEIHTYTAQSDTSEYYFIFLQQRDALIPHDMQGVFAYCWRHQLINCNVMTQSSGGQVLLHTYFPYAPGQCNDSQPTRINMFLGESWKHRDYFPSKLHNLNGCPLIVLARKVSPFLDLDEGQRELRGLEGRLLQELSRRMNFSIQFSGLQDQLKNRTTWTEKQLLQKLVQERIAHLAIGYVRKRIQYATNLTPVFPHYSNRVVGCLLLNAHNLTSLEIWSFPFQALTWICLVAGDRLALVLAVYAASLGLPIDPPERPSLQLLFASWLIFGLIVRSMYSALLFFILRYHLHQRLPGNLQDLTHGDYAAVMGRTTLQDLREVPSLQDLLGLKSVIVTSEREEEVLRTLDRCTLREGAGSHPLFFGLISQDALLHLTQRGHRAGAYHIIPQDVLEQQLAIYLQKHSHLASHLDHLVMSIRSVGLVHHWAGQMASERYFRSRFLYREKRIRQPDLWAVYILTAGLYLLSLVVFICELLASRRAGL

>DmelIR10

MAVLGTVFLLFMLDLKTLNLTRLNGLLVEPTRDLPQLELWLRAGSDHQDAENPYVQWFLLRTEIPLSIVTYQENRYWMDDPFGRRNLVLVMSLDQLLTNRGAAAPIQKASTFFYILADQDKDLSADEQLRLEGSCRQLWTQHKVYNRFFLTRDGVWIYDPFKRRDSAFGRLVRYYGSETLDKLLFRDMAGYPLRIQMFRSVYTRPEFDKETGLLTRVTGVDFLVAQMLRERLNFTMLLQQPEKKYFGERSANGSYNGAIGSIIKDGLDICLTGFFVKDYLVQQYMDFTVAVYDDELCIYVPKASRIPQSILPIFAVGYDIWLGFVLTAFACALIWLTLRVINLKLRIVSLGNQHIVGQALGIMVDTWVVWVRLNLSHLPASYAERMFIGTLCLVSVIFGAIFESSLATVYIHPLYYKDINTMQELDESGLKVVYKYSSMADDLFFSETSPXWNRDLRADVIDEVARFRNKAGVSRYTSLILESSHFTLLRKIWVVPECPKYYTISYVMPRDSPWEDAVNALLLRFLNAGLIVKWIQDEKSWVDIKMRSNILEADAESELVRVLTIGDLQLAFYVVIGGNLLAFLGFLAEHFRWKLQKKGV

>DmelIR11a

MRFAILWLFSGCLLPGIQVGIWVVVRAQPTGRDVLLSRLGNQQNELNTRRLANASSYLTRNYIANRINTLVVREICVECPYELSERQRQLVDQILASLAPELSVLLHKGTAEETTWEYTLFVVNDHTAFTGQVFIFPDELLEREFFCIVVVSEIQSRQFVRQTVGSIVKSNLQMHFVNVVVVAQLEDGTVGTYSYKLFKANCTPGITVRQINHFDRITGKPQQSMPDLYPVRNGHLGDCPFNVGAAHMPPHLIYKRHKDPPPASNVSIPAEDLAGIDWDLLQLLAKALKFRIQLYMPQEPSQIFGEGNVSGCFRQLADGTVSIAIGGLSGSDKRRSLFSKSTVYHQSNFVMVVRRDRYLGRLGPLILPFRGKLWGVIIVILLLAVLSTCWLRSRLGLSHPIEDLLTVIVGNPIPDHRLPGKGFLRYLLASWMLLTLVLRCAYQARLFDVLRLSRHRPLPKDLSGLIKDNYTMVANGYHDFYPLELTCRQPLDFSARFERVQRAAPDERLTTIALISNLAYWNHKHPNISRLTFVRQPIYMYHLVIYFPRRFFLRPAIDRKIKQLLSAGVMAHIERRYMQYENKRKVASNDPVLLRRITKSIMNGAYRIHGLVIVLATGMFILELLAGRSNGRLRRWMEWVHQ

>DmelIR20a

MLASLNRSTGLSAELLDLYGLVVHFLLSGEHTTLVYFNPAGLDCSWGVLWQRNLTAHPQIVWQRNYSYPDLYYQFNAKLLVLACLPMDSRAAIQLEILANSLSHLRTVVRLLIEVAGPDQVTLARQYLSFCLRRSMLHVELYFRDYHHSLILYSFRAFPSFELVMRWISVGQGVKLFLHKLDDLRGHRLRVIPDLSPPNTFFYRDARGDNQVTGYLWDFLATFAGRLNAGLEVVRPSWRAGSASDSSYMLEYSAKGLIDVGLTTTLITKWNLWAIHQYTYPLLVSSWCTMLPVEKPLATPDLFGRIVCPTLAMTLLLIILVTWLVFRQLRCLTRLKNSRPARIVPHLLTLLLLTTCSAQLLSLLIFPPYHVRIASFEDLLRGDQKILGMRNEFYNFDGAFRARYAGVFYLIDDPNELYDLRNHFNTTWAYTMPYIKWLVIKTQQRHFSKPLFRWSKDLCFFDFMPTSVIVAPDSIYWESIKDFTFRIHQAGLMKHWIRKSFYDMIKAGKMSIKDYSDLETLKPLNIGDLEIVWRVCGAAIAVASAIFIMELLYFYINVFFNSL

>DmelIR41a

MFIDLSWSLVLSAIVGKYLNESTICIFWNDKFEFQLLHKSDYISFVGINIKSFDDNGGHYIIDTGLKKKELQNKHLFLDELVIKIIISIEVTHCETFVVFDKDIDRFVNAFNKASVYSIWRSLHNKFVFAHIANESPESRNHFFEDQPNILFVVRDHSSASSFDIKTNKFVGRKAENPSQMILVDRYLASEQRFQFGKSLFADKLNNLQGREVIIAGFDYPPYTVIKHNMSTNAQDMGVSGESDFKNVYIDGTETRIVLNFCEQFNCTIQIDSSAANDWGKVYPNMSGDGALGMLINRKADICIGAMYSWYEDYTYLDLSMYLVRSGITCLVPAPLRLTSWYLPLEPFKETLWAAILLCLCAEATGLVLAYKSEQALYVLPGYREGWWTCTSFGVCTTFKLFISQSGNSKAYSLTVRVLLFACFLNDLIITSIYGGGLASILTIPSMDEAADTVTRLRFHRLQWAANSEAWVSAIRASDEALVKDILYNFHIYSDDELLRLAQDQHMRIGFTVERLPFGHFAIGNYLGPQAIDQLVIMKDDIYFQYTVAFVPRLWPLLDKLNTLIYSWHSSGFDKYWEYRVVADNLNLKIQQQVQETMTGTKDIGPVPLGMSNFAGFIIVWILGSAIATLTFLLELSLTYILKQSNLK

>DmelIR47a

MRQIKLLVWLLVVGVVSSTEQLQFLKNFLEAVHKERSISTILLIQRKVHKNDFLHGLYPIFWPIICLDETKRVELVNNFNKDFLALVYMESEADTLLLSALAADLNHIRDARIMIWLQMSPSENFLDRIVFQASKQKFLNLVVIENTLKTRRFYPFPQPKVQVIDKPFEEKEIYPALWRNFMGKNAIAVPDLVPPRSFNSFDPKTGHRRESGSIYNVFKAFTQRYNITMLLKWPLIRNTTQEEIIGKSVRGEIDLPITGQLISFRHPNGSRSQPLLGMTALSIAVPCGPELPMFDRFFLFYGLATPITITGYYVLLNTIEIILGTLSDRIKRHPRRKKILNLVLNLRVFSCILSLPTPQGNRLRSVKGQLTMVMSITGLILSCIVAAQTSTILTMKPQYRHIKNFQELSDSNITVVCNHLNYLTIKQQMDPKFMAKFMQNIWIVNSIEQMKMIFDLNTSYAYQTFSYKKDPFTLLQMHTTRKAFCRTPGLDLVSGLAYTAVLEKNSIYALALQDYTLKAFSAGLVYYWAEESIRDLISTVGRTQFEKLPIVIGYQSLKLQDYNVCWKILLIGGALAFCVFIVEVVVGLINRRI

>DmelIR47b

MREAQIIIFLLTSAAAVTLKQYEFLXSFLKAGEQEQTITTLLMMQKHVHTKNLLQGLYPXPWPIIHFVETQRIKFIALLYMSSEKDIFLSSLAANLKFERLDKPFGKSNIFPVLWRNYMGXIALTLDHLVEPRSFYWTDPRTNIKRRTGYIYMLITNFAEQHNITLQLXSPPNEDMSQMVIIERTHKGPRSTHNWADDQLETFERXQDSLLPWHGSMAIVVPCGQEMSAYERFHAAHAFRAPIIFFGFHIFLSLIDFLLRTISDRIRCNPRRIQLLQTVLSLCVLRCILSTSLPNSNXLRSRLRDNSPXXXVLQAXSYSALWXLTGTAXQXHNRDFQSHKLHDYXTTDGSXHSIEVPGLLKARNXXIXLFHIFSSLGTKFDLRIGSAGSHTSGVEFRYYELLDRXSSLENNIVSQVFTILKLPYSRFRVLKLEDCRGCWQTLFVGFSIATFVFIVNVLMGFFRNINQKK

>DmelIR48a

MHLLITETYMIIGKTLHDILNELNERLIISTNIIFCKQFDNLIHFEAQTSRFVYSSLEAFNITSLWNHVGNDNKLFVIVGNVPPYELFAKLELSSPENCTQFILNNTVDMCADALVKNSKAFSVSRELRIAPANVIVPHGKPLLSYRYLAAPFNTKVWIALGTYVFLISGFLCLIHWLRSGKWDFSQNLLEVYSSLLFTVFHLKATNGIERYILFGVLFISGFVYSTSYLRLLKSMLIAETFEKQIQTFEELAESNIPLLINPYDRMIFQHHHIPKSLWTAVRTVSSETLLNHRSHGYVRLCPAILTASKIPSHTHRHLFSVCRFSHEQEVVPKGSSXXSLVPCIRKRNREXNHLGCLSGVSWPGISXFFHYGALGGEAFGSILLHDANYFPSPRLFRRLAELHYGSY

>DmelIR48b

MILQQSSNLLKLLLLLAISSVRTQGLNDIIIELNQRLLISNNFLYCNQSDKLNEYEIKYLQHMPPISLMIFTSIESMNFTQVEYNLGADNKLFLIMGNEEPPYDFLHALNLHFQFAEYIIVIDEPVDLKKSTKWLDFVNHLWQQGYVQLLIYTSYDEKLYHKIIFPETVIEETLVEQYISIRGSFNNLYGYPVRVAAYNNAPRSMLYVNRWGKHIFAGFYMRFLRAFIDARNGSFVPVLTPSNSPGNCTLNLVNETVDVCADALAANPAAFSLTHGFRIASANVLVTHAKPLHSYRYLTAPFQWSVWACLVIYVLLVVNFLSFIGWLRSGKWEFSKYLLEVFSSLLFSGFYLKEIRGRERYILFGVLFIAGFVYSTEYLGLLKSMLISEVFEKQIDTFEALVESNITLMVDPYDKILFAKYNMPEILSPIMELVSFETLLKHRNRFDQDYAYILFSDRMALYDYAQQFLKHPKLLRIPIDFSFLYTGIPMRKRWFLKHHLGRAWYWAFESGLTRKLALDADFEAVRVGYLSFLITEHVEAQPLNVDYFVMPAIALAIGYILALLSFVIEMTAWRIREFLGCRKATMTSTGCSEGGHVDVD

>DmelIR48c

MSLLRIILIIIFLRIVSSIPDTIISHLSAELQIKIQIYFGLGNDLYDFSRLDGNYQKIIISHNISEEFKTYHDEPVLIIIRLERDLNLNLATLDVLRSYLTDRQYNDILLIDNDEENLNSYVDIRKAYWNAGFSQVLIYNSQQRTWSIKPYPYLQIRPTSLKEYIENRNTRNLMGYPLRVLVTNDPPHCFVDKDELPGSPNRYKGSIVTMLKIFADQLNATFQANPFREFRRYSTADCVQMVSDDEIDACGSIFIRTYTYATSQPVRLNRVVIMAPFGNPIEKFYYFFRPFDLYVWIGTGIIVVYIAVMGSLLHRWHFKEWNVGQYLLLAVQTLLNRELSLPQSSSGSKFMLLLLLFAIGFILSNLYVALLSMMLTTKLYQRPIENLADLKAANVNILLQTHNIRPNSVYGSSEELRERFLLVEESQHLEKRNGLDPSYAYVDSEDRMDFYLYQQKFLRRRRMKKLSNPVGYTWAVQVIKQNWVLEKHYNDHVQRFFETGLQNKLVDDVHELAVKAGFLHFFPTQTQTIEPLRLEDIVMAAMVLGGGHALAVICFLVELFA

>DmelIR51a

MYNVLVLFLLLFTRAQMEPHRRGHNMTLLRSVLTVIRGRENWKNTPIFLGGHCNSDDLNNLMSWLQNTMEVTCHTVDTSTSAKNENALGHFNINADNSLGLLFCQSSHELIWFNMDKRLRRLRGIRLIVILSDKRSSSSKAIMSTFKRLWHFQFQXNFQGYVVSTPVENDIPRVFFVKDKKTGRKQIRGFGYRTFVEYLHRYNASLHVSNSQQEHAINSSVNMGRIINQIVDGQLEISLHPYVDVPENMGDNSYPLLIASNCLIVPVRNEISRYMYLLLPLNQSSWILLLGSVIYISGVLYYIQPGLLHRTWDQRIGLNILDSISRIINICSPSRIYNPSLRYFIVSVHLSILGFVVTNLYSIMLGSFFTTLVVGEQVDSMQQLIQXQQKVLVKYYEVSTFLRHVEPDLVDGVAQLLVGVNASEQVSALLGFNRSYAYPFTLERWEFFSLQQQYAFKPIFRFSSACLGSPIIGYPMKSDCHLQSSLNMFIMRIQAAGLLRHWVVSDFNDAMRAGYVRLLENFLGFHSLDVDSLRLRWAVLLCGWLLSTLIFLCER

>DmelIR51b

MCKVLTLLVVILLLALTNAAYNVTLLKSVLSLISTREPWINTPIFVGHNTQGGDLNDLIIWLHQTMGVTSLTMNLFLQPEHIRPLGHFKITRYNGIALFFCHDKHDIMWLTLDRNLRKLRRIRLIIILRNQRSGSQGAIKSIFNALWQYQFLNVLVLQRDQLYSYTPYPAMRFFKLDIHTEPLFPHAARNFHGYVVSTPAENDIPRVFHVHDPLTKSRKVLGYAYRTFVEYLDHYNASLRLTNPDENLDPTTSVNMNHIVQLIIDGQLEISLHPYVFTPPTATKSYPLLIYPNCLIVPMRNEIPRHMYLLRPFQLYSWYILLFAVFYITGILYCISPKLNKSSWPQRLGLNFLDAISKILFISPPITIYRPTWRHLIIFLQLSVLGFMSTSWYNIELDSFFTTIVVGEQVNSMDQLVHQQQRVLVKEYEINTFLRHVEPRLVEKVSRLLVPVNASEQVSALLSFNRSFAYPFTEERWQFFAMQQQYAFKPIFRFSSACLGSPHIGYPMRVDSHLETSLNHFILKIQDTGLLNHWVVSDFNDAMRAGYVRFVDNVLGYQSIDVDTLRLGWCVLGIGWILSALVFSCEYWHLYPWRFIA

>DmelIR52a

MALGWSVIILGFIGQLSAQILNYTQSRDLELLEGSLFRVLSRLNLEEEYNTLLIYGKECVFHSLLRKLEISAVTVPSGSTDYDWSFSTAILILSCGYDAENEENSYTLMKLQRTRRLIYLEDNSEPESVCMRYSLKEQHNIAMVKSDFDQSDTFYSCRLFQTPNYVEGHFFKDQPIYIENFQNMRGATIRTVADSLVPRTILYRDEKSGETKMMGYLGHMINTYAQKLNAKLHFIDTSKLGAKKPSVLDIMNWVNEDIVDIGTALASSLQFKNMDSVWYPYLLTGYCLMVPVPAKMPYNLVYSMIVDPLVLSIIFVMLCLFSVLIIYTQHLSWKNLTLANILLNDKSLRGLLGQSFPFPPNPSKHLKLIIFVLCFASVMITTMYEAYLQSYFTQPPSEPYIRSFRDIGNSSLKMAISRLEVNVLTSLNNSHFREISEDHLLIFDDLSEYLVLRDSFNTSFIFPVSVDRWNGYEEQQKLFAEPAFYLATNLCFNQFMLFSPPLRRYLPHRHLFEDHMMRQHEFGLVTFWKSQSFIEMVRLGLASMEDLSRKRNEEVSLLLDDISWILKLYLGAMFISSFCFILEILRCGERCKRLWRCRW

>DmelIR52b

MTWLVILLCFLGYMAAHIADISVQNQSLMDNELINLLLKLRNEEFYDTLLVYGKDCEFHSVIKNVDVAVVLVSDSMNFEWNFSSLTLILSCGPDIDNGGPNSTSIKLQRNRRLVLLKEDFQPSNICNIYTQKEQYNIALVRENFTKSKSIYTCRYFQDPNVDEVNLSGTKPIFIEQFQNMKGKAIRIVPDLLPPRVMLYQDANDGELKMIGYVANLITNFAQKVNATLQLDFLKPSTSITEISRMAKDDELDMGITLEASLNTSNLETSSYPYLLTSYCLMVQVPAKFPYNLVYALIVDPLVLGIIFVLFLLLSVLLIYSQKMSWQDLSVANILLNDKSLRGLLGQSFPFPLNASKKLRLIFTILCFASIMLTTMYEAYLQSFFTNPPSEPEICSFQDVGSYNRRIAMSALEVNGLIKTNNSHFREIRMDDLEIFDNMPECYELRDAFNLSYNYVVTGDRWRSYAEQQTLFKEPVFYFARDLCFSRLIFLSVPLRRHLPYRHLFDEHMMQQHEFGFVNYWMSHSFFDMVRLGLTSLKDLSRPLAYTPSLLMDDISWIMKIYLAAIVLCVFCFLLEIGVDKWKRWMKFRNLQILNTC

>DmelIR52c

MVWLIIILFCLGNSSSQILDVTNNSHLDFDYRLFGLLQRLQVEKSYDTLLVYGEDCAIPSLFERLQVPAVLVSSGSTNFDWNFSSLTLILSCNFQDEREENYRTLMKLQTSRRLILLKGHIKPESVCDFYSKKEQHNVAMVKENFYQLEVVYSCRLFQDQNYEKLNLFDGKSIYKDQFRNMHGAPIRTLSDKEPPRTIPYIDSKTGEEKFKGYVGMLISQFVKKVNATMQIREDLIKDDEEVSFVDITNFTSNDILDIGICEARTLEMSNYDAISYPYLMSSYCFMAPLPDSLPFSDVYMAIVAPSILIMFLIIFCICSVLIIYIQERSYRSLTIRSVLMNDICLRGFLAQPFPFPRQYNRKLKLIFMLVCFSSLISTTMYTAYLQAFLWGPPIEPRLTSFDDVKKSRYTMAINIYEREFLEALNVSLEDVEIYDYGKFSKLRSTFNTNYLFPVTALQWFTINEEQKLFKYKIFYYCDAFCLNQFDILSIPLRRHLPYRDIFEEHMLLQKEFGLTKYWIDQSYRDMIRANLTTFKDFSPLLENDYIEVHNLYWVFTMYFVGMGMGLCFFILEILRPLRYWRNCKIKCEYCYAFLKNFAK

>DmelIR52d

MVRIIIILLCLGYTKARILDATNTNHTDLEERLLSLLLRLQQEQFFNTLLIYGEDCAFSSLSRRLQVPTILVSSGSTSFEWNYSSLALILTCEFKAEREENYQTLKKLQMNRRLILLNGNIKPDSVCDFYSKKDQYNIAMVNNNFHQVGIIYACRLFQERNYEKVYLSEGNPIYVDQFRNMQGALLKSITFNLIPGSMAYRDPKTGQEKHIGYVANLLNNFVEKVNATLDMQVKLHKAGKKTSFYNITKWASEDLVDIGMSYAAYFEMTNFDTISYPYLMTSTCFMVPLPDMMPNSEIYMGIVDPPVLVVLIAIFCIFSVMLNYIKQRSWRSLSLVNVLLNDICLRGFLAQPFPFPRQSNRKLKLISMLVCFFSVITTTMYTSYLQSFMWGPPIDPKMCSFADLENSRYKLAIRRYDIEMLRPFNVSMDHVVVFDESSQLEYLRDSFDDNYMYPMSALSWSAFKEQQKLFAFPLFYYSEKLCLKPISFFSFPIRRHLPYRDLFEEHMLQQNEFGLSTYWIDRSFSDMVRLKLATMNDFSPPRLEDYIEVSDLSWVFGMYFTGLGISCCCFGLELLGLPSWTRRLRLTNWLRVRN

>DmelIR54a

MWTVITGIVLWAPVLVAGSAVDFIFRAAAEHSLSVIMIRIDYCPYNWAKDIFENQTIPVVVLSDSETFINIRMFSRPLHVACLPGHELQKDLALLENFTSSLMDFPSQKKIVYISNNFSDPTRMDYIFETCYHRRIWNIVGLLASDEHRYFYRYHLYPSFRTEYRSLESSTIFDKDFPNMHGHPLTVMPDQWLPRSVLYVDRRTGKQILAGSVGRFFHVLSWKLNATLQLSKKVTTGRFLNATALKELSESFSVDVPASLTIMERVEQLASTSYPMEVTHVCLMVPVARRIPIKDIYFILSSASNMFLAIVIVSSYGLALNLLRNMTHRDVRLVDFVLNDKALRGILGQSFNLPLSRSFSTRLIFLMLGIVGLNVSSIFGAGLDTLMAHPPRQFQARSFAGLRRTKIPLVTTEEDFPTWMKLRVPMLVVNVSEYNHLRNGRNTSNAYFASRLYWNLFSEQQKRFTRELFIYSTDDCLWSLALLSFQWPQNSLFTEPVSQLILEVNANGLYDFWVGMHYYDMTAAGLSGLEDPSLQLKEREHPTSLRIVDFQWMWQAYGTFMVIAILVFLLEVSWHRITSLFVSLVY

>DmelIR56a

MGSRFFIRNLILFGLLASSNMQIPFGELEKKFELDVDFLLGVTELVGHIQGLYSITVYADCIDIHPSIQQRIMDKFMVPVNTIGSNLSRPNYHKLDNSRIRIVLFTGLNDTILVNLNKTDVPYSDNFYMLAYASAIKNKCIELDFIEEVFTLLWKMSIQNAILLIRGEFMMEMWSYLYMGKIHKIKLTKPNSYLESLRKYNYRFSLEVINDPPAIFWYNSSEQADVTGGGNLSVSGPLGLIIINFLRHLNVTIDIVPIPGKQTSQYELFQQPDNLRAENGVNMVGSALLKYSPMVTQSRMCLLVSNRRMIPFSRFLDRLVSPGVHKLTFVSSIGIFVIKYFSHRPRSFVDAIFCTIRFFFAIPLPSIILNRLPVVDRFIEVFIIIFVQILLSSNISITTSALTTGFWEPPIINVETMRASGLHILTEDPTILQAFKENILPSSLADLVILVDEDTYFHHVTTLNNSYVYVVQAHNWQIFRLYQQQMTNEPFEIASEELCSKWRILGIPLNPKSPLRFMFKDYFYRILESGLREQWVHSGFKKFCEFNNLKKLPVDSVDSWQPLSIEFYSNVIRAYIIGLVIATLAFVAELLHNGYRRKNVKKT

>DmelIR56b

MLLDTDLASGVIRSPYSFDIPHAFIFNETQFVVPKFCGPYMEIVKHFAEVYHYQLFLDSLESLPKKSVVEQDIISGKYNLSLHGVIIRPEETSDFFNATQHSYPLELMTNCVMVPLAPELPKWMYMVWPLGKYIWTCLFLGTFYVALLLRYVHWREPGNATRSYTRNVLHAMALLMFSANMNMSVKLKHASIRVIIFYTLLYIFGFILTNYHLSHMTAFDMKPVFLRPIDTWSDLIHSRLRIVIHDSLLEELRWLPVEYQALLASPSRSYAYVVTQDAWLFFNRQQKVLIQPYFHLSKVCFGGLFNALPMASNASFADSLNKFILNVWQAGLWNYWEELAFRYAEQAGYAKVFLDTYPVEPLNLEFFTTAWIVLSAGIPISSLAFCLELFIHRRKQRRPQYERFECYDY

>DmelIR56c

MRSSFRICLFLLTTYHPSHGWNMQHLLNLLAPFGRMNVFQEIVWFVSPHQRLDQLDEFIMRIDEAFGKSATQTVVNNNTEMRMIYSSARRNHMSFVFTTGAEDPIMKVFSKVLLGRHFYVSMVIYVDKVGDMHPIYDLLTFAYNQQFFNSMVHFESMEGVNQLFGVSKFPVMSFENRTDFLKYMGKIWKQVQNARSDVGGFGFTTPLRQDLPHLFQSQGHYDGSTYRIIETFVRFINGSFKELIMPPDSLGGQVINMKDALQLIRERKMEFCAHAYALFMSDEELEKSYPLLVVQWCLMVPLYNSVSTYFYPLQPFDWNVWFFALGALLALVLLELMWLRMFGGWSGYRGAVLNSFCYIINVPIEGQLQQPCLLRFLLLATVFFHGFFLSAYYTSNLGSILTVNLFHAQINTMNDIVSAQLPVMIIDYEMEFLLNLNKELPQEFLELLRPVDSAVFSEHQTSFNSSFAYFVTEDHWEFLDEQQKHLKQRLFKLSSICFGSYHLAFPLQMDSSLWRDIEYFTFRIHSSGLLNFYARSSFGSALHAGLVQRMPDTQEYTSAGLQHLAIAFILLLVMSFLAGIVFVLETLSR

>DmelIR56d

MDNRAAELILRERNIFPTNGSDNITLLNNMFVLEMFYRITQLYHFKNFIFYISERLDLNNKDSQEFFHNFWTYFPMAPNLIITREHHLGIPMMQFISTPSLVMVFTTGKDDPIMELASHNQQGIHWLKTIFVLFPSLQSRDFETNPESLAQFTAEIKDVYDWVWRKQFINTFLITIKDNVFILDPYPTPSIVNKTGVWQAEEFFHKYAKNMKGYLVRTPILYDMPRVFKSDRPTNRYEKNFIHGTSGNLFLGFLEFVNATLMDTSANVTADYLNMTNLLDLVSQGVYETLIHSFTEITTKFVVSYSYPIGINDCCIMVPYRNQSPADQYMHEALQENVWVLISLFTLYITVAIYLCSPLRPRDLSAAFLQSICTLTYSVPTFIIRTPTLRMRYLYILLAIWGIVTSNLYISRMTSYFTTAPPVRQINTVQDVVEANLRIKMLAIEYERMAKSPLQYPESYLNQVDLVDKHMLDLHRDPFNTSFGYTVSSDRWRFLNLQQLHLRKPIFRLTEICEGPFYHVFPLHKDSHMRSVMTEYIMIAQQAGLMNHWERETFWEAVHLHRIHVHLFDDEPMALSLDFFSSLLRTWTLGLILAGLAFAAEMKWHEHVTFKRRPVIRITRKPRSFLRRFMKL

>DmelIR56e

ERXAFRNQWAFCFPRTXAIEVVLSAWSPXCPGQRSKPQPISXPHHXGSCWRKRKWKXKPRLLVVDKRTLVEHLNSLNDGYAYCIIAGHWQVGMM

>DmelIR60a

MWCNNPGLIIIIFLGQILNLCQGIVNLSNETANTVIFMLPEKDLGPDVWKAGVGCLDSFAQIFFFRNPKERFTRAYNLMLVHAFHLSSPADQIQEGFSKLINEAVTNPGPPDREELFQMRVASDYNITNGTEDKGELILADNYVIVVDSVDRLKELMKKKIVEMRSWNPGARFLVLFHNATCRNRPLGVASNIFKDLMEMFYVHRVALLYANSTMNYNLLVNDYYSNVNCRILNVQSVGQCHDGKLYPNNAVVKASMQDYVSGFSPRNCTFFACSSISAPFVEADCILGLEMRILGFMKNRLKFDVNQTCSLESRGEMDGPANWTGLLGKVQNNECDFVFGGYYPDNEVADHFWGSDTYLQDAHTWYIKMADRRPAWQALVGIFEAYTWIGFILILIISWLFWFTLVMILPEPKYYQQLSLTAINALAVTISIAVQERPICETTRLFFMALTLYGLNVVATYTSKMIATFQDPGYLHQLDELTEVVAAGIPFGGHEESRDWFENDDDMWIFNGYNISPEFIPQSKNLEAVKWGQRCILSNRMYTMQSPLADVIYAFPNNVFSSPVQMIMKAGFPFLFEMNSIIRLMRDVGIFQKIDADFRYNNTYLNRINKMRPQFPETAIVLTTEHLKGPFFILVVGSCWAALTFIGELIIHRWRTQLVSTSEQQDRRSDKRRRRRRRRKPEKDNRWQRQVQVAPVVRFTPVKRRKVFQGQTSQK

>DmelIR60b

MRRSLYLIIAIGLVDVHCVSLRYILNALENELQYRAILLVESASEIESCWEQKYIQGAVPILNFNANQSLYLKDALNTNILALVCLNENVESTMQALYENLEDMRDTPTILFVLSDSKVQDVFLECLRRKMLNVLAFKGLDRGFVYSFRAFPTFRVIERNVMDILQYFEQQLEDLGGHTLTTLPDNIIPRTVVYKSPDGSRQLAGYLYPFLRNYVSTINATLKVCWHLVPEDGMIQLGEVVRLSEIHDVDFPLGMHGIEHGSTSQNVPLEVSSWFLMLPMEPSLSRAQFFIMLGFEKVTPVLLLLTILLSTAHRIEMGLRPSWRCYVLGDRVLQGTLGQAFFLPRRLSVKLMLVYSLILLNGFTFSNYSITSLETWLVHPPSGHPIHSWEQMRTLNLKVLIVPSELDSMTKALGKQFTESNSDLFELSKSGNFQDKRLAMDQSYAYPVTCTLWPLLEHAQIRLPKPEFRRSREMVLIPLLIMAMPLPKNSMFHKSLNRYRALTHQSGLYEFWFKRSFNELVALRKIHYKVNGDHQIYRDFEWQDFSYVWLGFVGGTIASILVLLAEIGYHRWQLNQN

>DmelIR60d

MRLAIYVAFLSSIGNRSGFLSSLLMSLGKELHYKTILLVGGSSTCWSLEPFETGVPILNLRGENNAYPQDTFNSQMLALACLQTESEDAVKLLYRSLKDMRDTPTLLFASSEEHIHDTLFLGCFRENMLNVLALTASSKEFIYSYQAFPTFRVIKRKLVEIHRYFEPQLKDLGGHIVSALPGNIMPRTMCYRNAEGERQLAGYLNTFIRNYVESINGTLRISWGLVPEDDMRHLTISRLSKIQHVDFPLGIIPLYNKTDKQHVYMEISSWFLMLPMETSVPRAHLFVKLGLERLLPIIVVVGAVLGNAHRIEVGLGPSWRCYYLADKVLRGALAQPIVLPRRLSPKLMLIYSLLLLSGFFLSNYYMASLTTWLVHPPASDRILEWDQLRYLHLKVLTIPEEFKYMSLILGTDFMTAYGSIFQLTNSTDFQRRRISMDPSYAYPVTTSLWPFLELSQVRLRRPLFRRSYDMVLQPFQVMSLPLPRNSIFHKSLLRYAALTRETGLYYYWFRRSYYELVALGKISYKEEEGNPYCDLKWNDFRIVWLAFLGGTIISCLALLLEVAHYRWHLGNSSL

>DmelIR67b

MELLYLNTLQSLSLLEGNRLVQTVQELNNIYQTELNVFLEFGNGADILESAQGTFVPTLWIKNPQNQKVMKGNFTSCTLTILYLEDEHLDRGLYYLANWLWEYHHLEVLIFFNGGSYDKLIQIFSRCFNEGFVNVLVMLPGSDELYTFMPYQDLKILNLKSIKEFYSLSRKKMDLNGYNITSGLVIAGAPRWFSFRDRQNRLILTGYMLRMIVDFTNHFNGSVRLMNVLTVNDGLELLANRTIDFFPFLIRPLKSFSMSNILYLENCGLIVPTSRPLPNWVYLLRPYAFDTWIAWLIMLIYCSLALRILSKGQISISAAFLKVLRLVMYLSGSRDMGTRPTTRRLFLFVILTTSGFILTNLYVAQLSSNSAAGLYEKQINTWEDLDKSDSIWPLIDVDIKTMEKLIPDRTKLLKKIVPTLEADVDTYRRNLNTSCIHSGFFDRIDFALYQQKFLRFPIFRKFPHLLYQQPLQISAAFGRPYLQLFNWFVRKIFESGIYLKMKDDAYRHGIQSGLLNLAFRDRHLEVKSNDVEYYYLIAGLWFGGLTLATVCFLLELLIGYAKIKVTISCKMNIM

>DmelIR67c

MFCWLIFLNIILLSDRSESWSAREVIHQFNHDQQLQLNIYLDCNDVELQIGQEVSNLFVNSTADKMKILGRFSSHSLIIACFKDSTRNRTLNGVKELLWGLQYLPILFVVDSNMDFYFQQALRHGFIHVLALNFMNGSLYTYKPYPKVEVHQIKDMQKFYKLTKLRNLQGQAVRTTVETMTPRCFRYRNRHGQLVYAGYMYRMVKEFISTYNGTEEHVFGNVDTVPYKEGLAALKNGEIDMMPRIIHALEWYYFYRSHILYNIKTYIMVPWAEPLPKSLYFIQPFRGTVWITIMVSFVYASIVIWWIRYRQQGNSSLTQSFMDVLQLLFQLPLSKIWHFNMGTHQVVSFIVLFVFGFMLTNLYTAQLSSYLTTGLFKSQINTFDDLFREKRTLLVESFDAEVLHNMTKEKIIQKEFESIILITSIEEVFKHRKSLNTSYAYEAYEDRIAFELSQQRYLRVPIFKILKEVYDQRPVFVALRHGLPYVELFNNYLRRIFESGIWIKLQEDSFLEGIASGEISFRKSKSREIKIFDKDFYFFAYILLGMGWCVSTIALFLELWSFKYSVTNVLHEG

>DmelIR68a

MRCLWILIVAFISLAMATSIPIPIANPAPLSGYEMQLKILLQKILWVANVKRCFAVITDDLHYPIYDRIFFESVGRRVIPFFVMRTNESDDLQRPSRQVELFVKAIKSSDCELNVITILNGWQVQRFLGYIYDNRSLNMQKKFVLLHDLRLFESDMIHLWSVFIDAIFLKRQLDNKYTISTIAFPGILSGVLVMKNIANWELGKGLNGRILFADKTSNLFGTSLPVAISEHVPMVLWANATKSFQGVEVEIMNALGKALNFKPVYYKPNQTENMDWTELDGGASVAYGSGNPDGYAQNGTHIDSMLVDEVAAHSARFAIGDLHLFQVYLKLVELSAPHNFECLTFLTPESSTDNSWQTFILPFSAGMWVGVLLSLFVVGTVFYAISFLNAIINGNVSSEFFRCLRPNRNVPMDPKIYRRISFRIAISRYRSSKGDRMPRDLFDGYTNCILLTYSMLLYVALPRMPRNWPLRVLTGWYWIYCILLVATYRASFTAILANPAARVTIDTLEDLLRSHIPPSTGATENRQFFLEANDEVARKVGEKMEVFGYSDDLTSRIAKGQCAYYDNEFYLRYLRVADESGSALHIMKECVLYMPVVLAMEKNSALKPRVDASIQHLAEGGLIAKWLKDAIEHLPAEALAQQEALMNIQKFWSSFVALLIGYVISMLTLLAERWHFKHIVMKHPMYDVYNPSLYYNFKRIYPQH

>DmelIR68b

MKFLVGLLLQWYLPGIYALAEIACRIAVEQNVQVTYLYRCASCPASFDADYSALELDLYRCVGSRLPVITRNMEAHELEPFRRTDSLSIFQIPAAEKGDSLVRRILDMLNPHQRRKHMHKYLFVWPNAGRHQLLRLFRGSWAKKLLYGLAITGRENGTFDFDPFAWGGLQVIQRLDGEVPYARKVKDLRGYPLRFSMFTDPLMAMPRSPVETAGYQAVDGVAARVVGEMLNASVTYVFPEDNESYGRCLPNGNYTGVVSDIVGGHTHFAPNSRFVLDCIWPAVEVLYPYTRRNLHLVVPASAIQPEYLIFVRVFRRTVWYLLLVTLLVVVLVFWVMQRLQRRIPRRGVIQFQATWYEILEMFGKTHVGEPAGRLSSFSSMRTFLMGWILFSYVLSTIYFAKLESGFVRPSYEEQVDRVDDLVHLDVHIYAVTTMYDAVRSALTEHQYGLLENRSRQLPLGIATSYYQPVVRRRDRRAAFIMRDFHARDFLAITYDSQAERPAYHIAREYLRSMICTYILPRGSPFLHRLESLYSGFLEHGFFEHWRQMDLITRVGASPDAEEFLEDLGDQTDTDSGSNELAIRNKKVVLTLDILQGAFYLWSVGIGISCLGFAVEHAHWFWRRQTLRNAVEARTS

>DmelIR85a

MSIQWLKHILLLAILVNLAGTRENHIPLDLKKSSIVMVKMSQILCKARIKVLFVYFENQTSHEHTGQILKEVTKCDISNQNTPLEAVKDDGILMYMVMITTNISQPLELSLIRKKSAAKHRSHVFLLVRDADTVSDAWMRASFRQFWKIWLLNIVILYWRDGRLNAYRYNPFMDNYLIPVDNKPNEVPTLEQLFPKTIPNMQRKPLRMCIYKDDVRAIFWRQGTILGTDGLLAAYVAERLNATMMITRPHSYNNHNLSSDICFLEVAKEYVDVAMNIRFLVPDTFRKQAESTVSHTRDDLCVIVPKAKTAPTFWNIFRSFGSLVWALILVSVLVANVFCYILKSEVGRVPMQLFAGALTMPMTQIPPNHSIRLFLIFWLYFGLLICSAFKGNLTSMMVFQPYLPDINQLGALARSHYHIIIRPRHVKHIQHFLTLGHKHESRIREQMLEVSDTQMYEMMRNNDIRFAYLEKYHIARFQVNSRVHMHLGRPLFHLMNSCLVPFHAVYIVPYGSPYLGFLDSLIRSSHEFGFERYWDRIMNSAFIKSGVKVVNRRRGSGNDEPVVLKLQHFHAVFALWLVGIGMACIVLAWEHLTHNYNLAVTKRRD

>DmelIR87a

MSTPEQRFWLAALLFLLSQHSEVRGFGINLMKVQTEDKGQEACILALLRKYFDSGDGLSGSVLCINRNYQLPNIEEQLLRGVNNYENYPWSLLITNSREGPSPAKFLMNEKPQCYFLIVDNLEDEDLDEVFEHWKGMVNWNPLAQFVVYLASLEETDEEMNDLMVELLLTFINKKIFNVNVIGQSEENQFYYGKTVFPYHPDNNCGNRVISVELLDACDYPSEETDSEDENDEDEGDGAQEEDDGPQEEGDGEQEEEDGPQEQEDGDQAKGDEGQENDDGGLENKVENEFRIGASDDDELENDLSSNSSEPEAIIEEFFRAKFEDKFPRDLSGCPLTASFRPWEPYIFRNSEEQPVDDYYYGLQGDEDDYNDTSPNYGESDDESYADPGEDGDGAIPDTETQSGGKLKLSGIEYEMVQTIAERLHVSIEMQGENSNLYHLFQQLIDGEIEMIVGGIDEDPSISQFVSSSIPYHQDELTWCVARAKRRHGFFNFVATFNADAGFLIGIFVVTCSLVVWLAQRVSGFQLRNLNGYFPTCLRVLGILLNQAIPAQDFPITLRQLFALSFLMGFFFSNTYQSFLISTLTTPRSSYQIHTLQEIYSNKMTVMGTSEHVRHLNKDGEIFKYIREKFQMCYNLVDCLNDAAQNEHIAVAVSRQHSFYNPRIQRDRLYCFDRRESLYVYLVTMLLPKKYHLLHQINPVIQHIIESGHMQKWARDLDMRRMIHEEITRVREDPFKALTFDQFRGAIAFSGGLLLVASCVFAFELCYVKYVYRTEKRERKTKKITKKVHNIKIQHD

>DmelIR94a

MALPKQLKFINIFLVLLIIYGSSDGTENQHEIFLNRLLQAVHNERSVETLFLLHHSNLANCSLQDWNPPRIPTIRSNELTVFNVEKTFNHNALALVCLMKNSYREILNTLAKSFDCMRQERIILMIHRKSDSKFIEDITHEVKNLQFLHLIVLIVQEKYNGQVFASTLRLQSFPEPHFKRIRNVFAIQRIFYRPINFHGKVLNAIPNDIPILFVALNEMFTEYARRYNSTLRIQNRTIKEDIEITEDNYDIDMKIQLHNSQNFLHHMNIAMDIGSNSLIILVPCATELRGLDIFKELGVRTLTWLALLFYIIFVLVEMLFVFISNRFNGRNFTMRYTNPLINLRAVRAILGQTSPISNRYSLSIQHFFVFMSLFGTLFGGFFDCKLRSFLTKRPYYSQIENFSELRKSGVTVVVDHTTRQFIEQEINANFFRDEVPNVRTTTIQELINHVYSYDRKFAFVANSIPWRTFREEMKSINQKILCDSKNLTILENVPLTFSIRRNAIFSHHLRNFIINAADSGMITCWFKMAGKVIRKHIKTTLRESEQQPSHLPLSFDHFKWLWAVLCIAYVMSFMVFVMEILWSKYQRRTRSVSIV

>DmelIR94b

MSLIFNLLFILILSQAVSQETEFLQLKYLNNIVRSMIKLHKMETLVIVKHHLDNNCSLQNWNAHGMGIIRTNDQGKLIMKDTFNSRTLAIICIGQNSHITLLRNVFETFGKVQQKKIILWTQMELKEKFFQEISKKSRDLKLLNLLVLKAVTKDKLLIYRLNPFPSPHFKRIENIWTPNDTLFMDTKFNFHGMTAVVKHDYNWTIQMGNIRKFPISRIEDKEVIEFALKYNLTLQFFNDVERFDIELRKRIILKSNSTQPIDSGIPMVFSSLLIVVPCGNYLSIQDVIKVSGIEKWIFYIILVYVIFVLIEITFLGVTILISRQSRHQMIPNTLVNLCAFRAILGLPFPETRRTSLSLRQLFLAIALFGMIFSIFINCKLSSMLTNPCPRPQVNNFEELKTSGLTVVMDHDAENFIEKEIGVDFFNQYMPRKVTLTFTERAKLLFSLKGNHAFTLFSESFAIIESYQRSKGLRAHCTSEDLIVAERVPRIYILENNSILDRPLRRFIRQMQESGITNHWLKNIPSSLEKNLMQITIPYDRERVHPLSIEHLTWLWCILILGYSISMIVFFVEMSLKRRKKNLENRAPNICIC

>DmelIR94c

MSKVFKLLVLPLIYLSLTKGSKNPQLKFLRELINVIEEGREIRTIMVIKHSRDEYCHLDQWNPRGSPILRTNEMGSIRISGYFNDQAVILACMGENSDYGLLKSLANAMDNMRQERIILWSEREPTKMLMDYISQQADRYNFAQIIIVTMNEDVDAVPSLHQLNPYPTPRFRQITNISNIRRTSFFGCGLSFQGKTAILKESVVSNIRFKVWSPSGPIPLSELKDYEIVQFAVKYNLSLKLYDQNESKSDHFDIQLGPLFITKDFPTQMAFVSPNTACSLIVIVPCSPKWRFMDVLHKLGVLKLIGCLLIAYAVFVLIETLILWLTHRISGREVRLTSLNQLLNPRAFRGILGLPFPEFRRSSISLRQLFLVISVFGLVYSNFVSCTLSALLTKPAQNPQVRNFKELRDSGLITIMDKYTHSFIEKHIDPEFFDHVLPHYLILQKKEALRMIWNFNDSYSYVMYTTTWKSLNTVQKSFDERVFCESESLTIAWNLPRMYVLGNNSVLKWMLSRYITYMPQTGIPDSWTEQLPKVLKLLYNVTSPRRIKEGAVPLSIQHLSWIWHLLFIGESIATLVFIVEILLQKSNQHTSNMRERSSEDDDFV

>DmelIR94d

MGQLHLLLVALVLLSPGGDSFYHSLIHHLNRELKIEYVLLLGNFDTTWLDILWQLPVSVLQIKEHSRETYSLLENPSHNVLTIAFVNDSPEDILEILYRNLRMLNTQPVLLVIRKSTIRVNSLLEWCWHHQLLKVVAIAQDFMESLIVYSYNPFPVLQFIERRLDNSTVIFEKRLENLHGYEVPIALGGSSPRLIVYRDLEGKLIFSGPVGNFMKSFEQRYNCRLVQPYPFDESAISPARDLIASVQNGSVQIALGAIYPQVPYTGYSYPIELMSWCLMMPVPEEVPHSQLYSMVFSPMAFGITIVAMVLISLTLSMALRLHGYRVSFSEYFLHDSCLRGVLSQSFYEVLRAPALIKAMYLVICLLGLLITSWYNSYFSTFVTSAPRFPQLTSYESIRHSNIKIVIWKPEYEMLLFFSENMEKYSSIFQLQEDYKEFLHLRDSFDTRYGYMMPMEKWSLMKEQQRVFSSPLFSLQDDLCVFHTVPIVFPMVKNSIFKEPFDRLILDVTATGLLSRWRDMSFTEMIKAGQLGLEDRGHPKEFRAMKVGDLIQIWRFVGWMLGLATIVFLLELICFWRHKMWQNMKYMFCRNKNI

>DmelIR94e

MDCPKWILSGLCLISLVSGATVIELLGTLKLELDFEYVLLMKNRNFSLSDQVWNGTSLTKDVMDEVQVPVLQFNENVSYFLHNSISRRLVTLGFMSDANLDEHRGLLTALVANLRHMTTSRVIFLVQSKASTDFLYELFRNCWRKKLLNVIVIFQDFETTSTFYSYSNFPILQIEERIYETSLQTLPIFPDRLRNLHGYEMPVILGGTAPRMIAYRNKKGNVVYDGTVGHFMTAFQQKYNVKFVQPLQAKNPLDFAPSMQTVGAVRNETVEISISLTFPTIPPFGFSYPYEQMNWCVMLPVEADVPPFEYYTRVFELAAFLLTLGTLVLISCLLASALSLHGYATNISEFLLHDSCLRGVLGQSFVEVFRAPTLVRGIYLEICVLGILITAWYNSYFSSYVTSAPKQPPFRTYDDILASKLKVVAWKPEYAELVGRLLEFRKYETMFLVEPDFNRYLALRDTLDTRYGYMITTNRWVLINEQQKVFSRPLFQKRDDFCFFNNIPFGFPLHENSVFMEPVQKLIMELAETGLYYHWITTGFSELIDAGEMHFVDLSPHREFRAMQIQDLQYVWYGYAFMVVLSSLVWLLENLAYTVKSKTIFPTHFMQRNKK

>DmelIR94f

MSGMWQQVLLAETSNWFRSDVLQRFWTHLRVEIRFRTMLNYRLESCDCWFDNVLGSDNSTALLWNDQTYPHYLRRRQDTDILVVSCLRFHQYQEVLLALSLMLDQMRSMPVVLQLCGDEDSMQELNSARLLLKHSQDLKMPNVVLLSSTFFTSATLYSYEMFPEFNVQKLVYQAYLTLFPYKLGNLKGHPIRTVPDNSEPLTIVRKTLNGSIAIDGLVWQFMIEFAKHINATLQLPIEPHPEKSIKLVQILDLVRNQTVDIAASLRPYSLNVQRSSTHIYGSPMMVGNWCMMLPTERVIGSHEALTRLMKSPWTWLILLLFYSVHRFLAQKTRLRSSLIHLIKLLINLSLICFLQAQLSAYFIGPQKVNHISNMQQVEESGLKIRGMRGEFMEYPIDMRSRYASSFLLHDLFFDLAQYRNSLNTSYGYTVTSVKWELYKEAQRHFRRPLFRYSEEICVQKLSLFSLIQQSNCIYCYRSRIFILRMHEAGLIRLWYRRSYYVMVTAGRFPIGDLSTVHRAQPIRWTEWQNVVLLHGVGLLFSVVVFVIELTVHYANVCLNNL

>DmelIR94g

MSTAVNSVHSKLVSLISRGQELTSIFFYAPAKEKCHLEDTISSATWGLPLVIWRTDRTVILNGFIGEGLLVLACLPGFHWRALLGSLARSLKYLRQARILIELMQDRDEFLVSEVLQFCLSQDMINVNAIFDDFPETENLSSFEAYPSFEVVNQTFTPDTQVSDLYPNKMLNLRGGVIRTMPDYSEPNTILYQDKEGNKEILGYLWDLLEAYAHKHNAQLQVVNKYADDRPLNFIELLDAAQSGIIDVGASIQPMSMGSLSRMHEMSYPVNQASWCTMLPVERQLHVSELLTRVIPYPTLALLLLLWIFYEVLRGRWRRHSRLQSIGWLVLATLVSSNYVGKLLNLFTDPPSLPPVNSLAALMESPVRIISIRSEYSAIEFTQRTKYSAAFHLALHASILIGLRNAFNTSYGYTITSEKWKIYEEQQKRSSKPVFRYSKDLCFYEMIPFGLVIPENSPHRAPLHSYTLLLRQAGLHDFWVNRGFSYMVKAGKINFTAVGERYEAKTLTITDLRNVFIIYVSVLLISLILFTCELFVSWVNYWLGF

>DmelIR94h

MLSNISFSSAPELVDLYGLVLKFLVSSETTLFYFNPTGQKCSWETLPRTILSNHPQIIWFREETYPGLYKRHSSNLFVMACLSSTSYDGQLQLLAESLTRYRSVRVLIEVQDKEGSFLASQILLLCQQHSMLNVVLYFSRWTRTLNVFSYLAFPYFKLLKQRLSGSLRPKIFINQLKDLQGYKIRVQPDLSPPNSFSYRDRHGECQVGGFLWRIVENFSKSLKGDTQVLYPTWAKAKVSAAEYMIQFTRNGSSDIGVTTTMITFKHEERYRDYSYPMYDISWCTMLPVEKPLSVEILFSHVLSPGSALLLILAFILFFLIVPQLIKCLGITFRGRLIGMASRIFALVMLCSSSAQLLSLLMSPPLHTRIKSFDDLLTSGLKIFGIRSELYFLDGGFRAKYASAFHLTENPNELYDNRNYFNTSWAYTITSVKWNVIEAQQRHFAHPVFRYSTDLCFSSETPWGLLIAPESFYREPLQHFTLKINQAGLITQWMTQSFHEMVRAGRMTIKDYSRTNLMKPLRIQDLRKCWVIFAVGLGTSTVVFTIELLLIYTNVFLNSL

>DmelIR100a

MATTLQLIMLALVGGTLGQANNTDHKQVLTSIVKQLEGGLELHLRTSEDGGNDLVQFLMQEKSSIIISAKQEEVPSRAKIMRHHFFIFDGVHQMQEIRTSLFNTDGFYILALENNTIEDDVLLMEFAADVWLQHGHSRIYYVQLSKKSVLLFNPFLQRLVVVQDSKTYSRIYKDLEGYHLRIYIFDSVYSSVIGDGENKVLSVTGADAKLAKTVARQLNFTADFVWPDDEFFGGRLANGEYSGGVGRAHRGEVDIIFAGFFIKDYLTTHIQFSAAVYMDELCLYVKKAQRIPQSILPLFAVHMDVWLCFLLVGLLGALVWLILRAVNLILGIEGVPDGSRATRISYFGAARRIFVDTWVIWVRVNVGRFPPFHSERIFVASLCLVSVIFGALLESSLATVYIRPLYYRDVNTLRELDESGQPIYIKHPAFKDDLFYGHNSEVYRRLDAKMMLVAEGEERLIEMVSKRGGFAGVTRSASLQLSDIRYVMTKKVHKIPECPKNYHIAYVLPRPSPYLEEVNRIVLRLVAGGIVGLWTGEAKERAKWSIQRFPEYLAELDVGRWKVLTLSDVQLAFYALTIGCLLSAIVCMAEILLGRQRRLHSPK

>AgamIR100a

MRWIWAAVVVVVLAAANLSASDPSTTLSFESLSSQQQQQESDQSDALIATIFATIDHLDIVNGAAGAAETQLLQHRTVRWFDGEPATLCGAPPASEHSECFAPPERPTDRHQWDGALAGAGLILYGSVAKLDRYACLFEPAGTYLLVDEPSRSPLEQAHLRRLLGTLWTTRGAYRVYVRARGQLYGYDPFRRPAGAAEYGALVQLEDGRPLPTVPLTDFGGYPLRIEVFRSVYSNPDERASASKADQITYSGPDVMARDVFCRALNVTVIPVPADRDLFGDRLPNGSFTGALGRLVRREVDIVFTGFFIKDYLARDVEFTAGLYSDAVCCLVRKASRIPEALLPLYIFPGDIWALLGALGLACASVWALLRWCVRHVKPPAGGLWSRRHRLAVLFNLPRSLRAAGPVRRTVQLYVDSFILLVSAPYQRFTRSGVERLFLTGLLLVSLIFVSLYTSGLAAVFVNPLYYPDIGTLQQLDESGMAIPVKYRGFLDDVFAVNYSRLMDSLRARMQHLPAKESMLARVARLGNIATVTRKTTLALDNAIYLTTRQLHMVPECPRTYNLAYVMPHRSAFGEQFNKVLLRLVGGGLVDHWIEQARYGWTLRDWRVARRMMESSFKVLTVLDMQFAFYVLAIGLAVSVAVIGAELVHFHRAHRGDGHGVNVLLRK

>AgamIR93a

MVLRLVGLWSILLLLLLLLVLRPDPAVGDDFPSLLSTNASMGKLNITPLLSIILDREYLGADYERTLDETKNVVEKLIREHLKNGGLIVKYYSWTSINLKRDFSAVLSVSSCKNTWDIYQEAVRERLVMLSITDPDCPRLPTNNAIMIPRSDGSGSNAFDEVSQIILDMKSSRAINWHTATLLYDQVYDAEISRCILSLLEDREGIKPLTLTEFKINAPTHSWEKRKEIRRTLLGIPTAYTGRNFIAIVNIATLTLLMEISKDLKLVNPFAQWLYLIPNTEKANSNFTTRSTLINEGDNVAFVYNSGSKAQNCTVSVLCYIESYLLHFIRSLSKLIREEQVVFGQISDEEWEIIRPSKQERKTKFLQMIKAAITSKDECNKCSQWKIQSAETWGYVYRTDFLTDGADLQERRKYTMLDIGYWSPQDGFMLTDALFPHTQYGFRGVQLIFYSYHNPPWQFVAYNDSGSPVISSGVVYDILNELSRKLNFTYTMVISQPAEINGSLVEGNTSSVYDLKTISSDIPQEIFSTLVNNKILLAAVGATVNEKQKKFVSFTDPISIQTYSFVISRPRELSRVLLFLSPFGSDTWLCLAAAVALMGPILCAINKLSPYYEVHNKPTDTGLGKVNNCFWYIYGALLQQGLYLPYADSGRIIIGTWWLVVLVIVTTYCGNLVAFLTFPKIDIPVNRVMQLLRNDRGMTWSIRRGTFLEEMLMDSTEPKYMQLYKGSQIIGELTDELVERIEAGQHVHIDWRNNLRYLMKRQFLRTDRCDFALSTDEFLDEQIALVMPKDSPYLELVNEEIKRMHQFGFIQRWVAQYLPAKDKCSGTGRVMDVQNHTVNSSDMAGSYWILLLGFVSGLFVFVCEFAVAWYRKHRAARAATVAYRD

>AgamIR100h

MPEAVVPCCLLLLLLTLPLSAGSLPAGGVDLIARTPYSLDAGSTVPVRERLIALERRLVQQQQPCTRLLIVFVQLSDGRPPHLAGDTLSLAIDAFAPGHRTPRLVLRGDDEAAADALSSAATLADRDSYCVVLILLLPDLGNSLPGTLLQTTARLFPLALKLLLLGSAAPTDEELSAARTLLAALWQTERALRALTIWYRPATGLLIGAYDPFEPPAGRFYTLAGPNRTDLPTDLAAFGRRMNLRGSAITVYGFEAAMAYRRQDIDMLPRTFPARRPGSTHASRDALLDALFGADVEAVRELARRMNFTPVVHHMRANFGFKMANGTFTGVLGRLVARDAWLSMNVYFLKDYETRDLQFGAAVYQDSLCVFVQAAGMLPDWLLIFRCFTPTLWIGVWSTVAVVSLCYLALTLLIAIYRPAWEHGRQHTGNVSPDQPQQDLTGLVGQIVGALLTAPTNGFHRTTTHQKLFVAFGLIWGLTITGAFQGSLVDVYTTPTSMKNLDTLAELDASGLSITVTAPALIVDVFGTERPGSTLGNLKRRLVIETATNRSAAYGVLGGRTAGLIRNQDFVRLSTKYLGRDGTPRLHRMRQCPRSYTLAYLYPRGSPLYRAANDHTLHFLQHGLYAKWQREAAHIVRANRALKVQRYAGQAGRTAGQAVTLRLDHLLLPFMVLGAGFALGVVCFAWECRALLTAAVAVRN

>AgamIR7n

MRLSWTCTGQLLVLLLSVLRFSAAAANLANNKTLAKLTGQLQSASFTHTELLIGATAEILLQHYTTLTSKQLYVRWEDGASGPLVDEILRRTATHLSVLVERDPPVTPADGLDQSHHPQLRLLNLLLVTDRAEFDHVVAGLTDELYDYSGLYTVLLVGASSKRQHLETAGIILRTLWALYIVNVVVLIGDGRQQQADGDDDDDDVRLYTYFPYGEDYCERALPVVWNVYERGVGFVHREHSPFPPKLDNFYHCPLAVATFPVYPFIIPSAMMADLWPGHPERTEQEEEEDLKGIEAMVLRTLRQRLNFRLQLMNVDPPDWGTAGPRDQATGASAYIRHLRANLTIGYWATTLHRNRYMANSFAYYTSQLVLAVPPGAPYTSLELLRCPLAKPIWTFLLCSLAAGLAVIGLLRWAGSPTARHFVLGHNAPNRAWPA

MIAVLLGAGLSRAPRGSFARALLLCWLAGTLVLRSAYTGSMIRFLQSDRNHTVPANLPALLDAGYQPYMYRNYSFVFDAYPHIGRRVQLVTAHQFRTAIVRRLQRPGARLCVLLPLETVTFLNRNLTRTGQLLRIARERVTVAKLAIYTQRTSALLGPLNKLLERFVASGLLHRWAAQYHQLRFLANPYQYRGRQQLVLADMDGPFEILLVGLGLGAGTFLIELVVGRWEGGNAKNAKAASNSRTKNRTAVCLSSCVTRSIMTNDASTGNQRAGEHYYYTVHEH

>AgamGLURIIc

MSTIGRRRWKVLFALLLITQLAAVIGYHHGDLDETVEHIKIGGLFDGATEDVELAFQYAVEAVNNEKLSYSNYQLEAQAVQVKYGDQFDASKKLCRLLKTGVAGIFGPSSPKSALHVQSVCDEKEMPHIETRWDAYTKLPTLNLHPHPHIMGRVFLDLVVAFEWKSFTVLYESGPWLPGIADLLKMYDPGYTVTVRQLDLGLNGNYRAVLRRVKLSEDKRIILACSIDSMPEVLRQAQQVGLLTDHHQIIVTSLDLHTIDLEPYQYSGTNITGVRLIDPEEDKIRQVADFLNASQIAKTLELQDGLNPAKMRVETALMYDAVLLFAEALKHLIGSEPAHLLEPIALRCDDTATWKNGYSVINYMKSSTIHGLTRSIRFDHQGHRSDFLLDIIELGPAGLEKVGVWNSTEGLNFTRKKEQTLLAFDDGTLQNRTFLVLTAISPPYGMLKDSPVKLTGNERFEGFGIDLIHELSLMLGFNYTFILQEDGVYGSLNRDTKKWNGMVLELLEWRADLAITDLTITSDRESAVDFTMPFMNLGISILYRKPTKEPPSLFSFMSPFSKQVWLYLGGAYMMVSMSLFILGRISPKEWDNPYPCIEEPEELENQFSFSNSMWFTIGALLQQGSEIAPKAPATRAVASIWWFFTLIMVSSYTANLAAFLTVEQVVSPISNAEDLAAAGGTIKYGAKRDGSTISFFKDAEYGTYAKMYQYMMANQELLTSSNPEGLQRVKTENYAFLMESTSIEYIVERECDVTQIGGLLDDKGYGIAMRKNSPYRSALSEAVLRLQEQGVLTSLKRKWWKEKRGGGACSQSTSDDGAEELDMDNVGGVFFVLFVGCSFASLFGCCELFFVIAHRARRHKVPFREELMAELRFVAKCHGNTKPVRHRKSSSASGENSLELDVEDSTSSSKQDISGNGMGDRLPKGNQRRQNGGKLALNGNTRKMLSED

>AgamGLURIIb

SVPGGLFHPDDDHQEIAFRYAVEKINSDRTILPRSKLLAQIERISPQDSFLASKRVCHLLGVGVAAIFGPQSSHTASHVQSICDTMEIPHLETRWDYRLRRESCLVNLYPHPSTLSKAYVDLVAAWGWKSFTIIYETNEGLVRMQELLKAHGLSDYPITVRQLSDSGDYRPLLKQIKNSAESHIVLDCSTERIYEVLKQAQQIGMMSDYHSYLITSLDLHTINLDEFKYGGTNITAFRLVDPENPEVAQAIHNWTIGEARLGKKVDFKTAVSAETALMYDAVHLFAKALHDLDTSQQIDIHPLSCDTQDTWPHGYSLINYMKIVEMRGLTDVIKFDHQGFRSDFVLDIVELGPQGLRKSGTWNSTSGVNFTRTYGEQQKEIVEILQNKTLIVTTILSAPYCMRKDSAEKLTGNSQFEGYAIDLIHEISKILGFNYTIRLAPDGRYGSHNKETGEWDGMIKELLEQRADLAIADLTITFDREQVVDFTMPFMNLGISVLYRKPVKQPPNLFSFLSPLSLDVWIYMATAYLGVSVLLFILARFTPYEWPTPNPCDPHPEKLQTQFTLMNCMWFAIGSLMQQGCDFLPKAVSTRMVAGMWWFFTLIMISSYTANLAAFLTVERMDSPIESAEDLAKQTKIKYGALRGGSTAAFFRDSNFSTYQRMWSFMESARPSVFTASNIEGVERVVKGKGSYAFLMESTSIEYVIERNCELTQVGGMLDSKGYGIAMPPNSPFRTAISGAVLKLQEEGKLHILKTRWWKEKRGGGSCRDDTSKSSSTANELGLANVGGVFVVLMGGMGVACVIAVCEFVWKSRKVAVEERVSLCSEMASELRFALKRNRAQPKHAGSGGTLRTASSRASPKDSASEPSSEHGRFHPLGPPYAQYEFDPKAVP

>AgamGLURIIa

MQGTRRGVSVHYLPAILLLVHALCGSVDGKREIPIGAIFHQVPDHSYESEIAFRYAVERVNMHEKHFELVPIVRYVSPEDSFRTERKVCELAAEGVTAVFGPSSLLTAGIVGSICKTLEIPHIITHWDPEPLGGIEAELRAMTINLYPEADVLSRALADLIVDYSWKSFTIIYDSDEGLMRLKDILQIHGPADAPITVRQIDDDPDYRPLLKDIQSSGESHIILEIRPDRILELLRQAKEVKMLEEYQSYIITSLDAHTIDFEELRYSRSNITALRLMDTKSFDIKNAVHDWEQGEARMKRPFRVSPEHVHTESALYNDAVKIYATAIRELDATEEITPARLSCGSKNLRQWPFGLRIVNYMKVKTEHGITGPIIFDDFGRRAHFHLDIIELSKDEGFKKIATWDPTHGVNYTRSQGEVYSQIVESLQNKTFIVASRIGAPFLMFKEKKDGEFLEGNNRFEGYSLELIDGISKILGFQYRMELVPDGKYGSYNKVTKKWDGLVKYLLDRKADLAVCDLTITYERRTAVDFTMPFMTLGISILYAKPVQQPKDLFSFLSPLSLDVWIYMATAYLGVSVLLFVLSRMAPADWENPHPCKQDNDEVENIWDMCNALWLTMGSIMGQGCDILPKAISTRLVAGMWWFFALIMLSSYTANLAAFLTMERMDATIESAEDLAKQSKIKYGVVMGGSTMAFFQTSNFSTYQRMWASMESARPSVFTKSNDEGRDRVIKGKRMYAFLMESTSLEYITERYCDLTQIGGLLDSKGYGIAMPVNSPYRTAISGAVLKMQEEGKLYQLKTRWWKEMRGGGQCTEVPNSSQENELGIGNVGGVFVVLALGCFCAFLIGILEFLWNVRKVAVEEKVTPWDALKAELKFALNIAVTSKPVHNTLSESTASGKSSLRSKSESRRGSELAGRGNSVRTAASLTNLDKSGFAFDKH

>AgamNMDAR1

MKLTRMLPASCMLLSLMIPQALLAQKPTGSESPSYYNIGGVLSNNESESHFATVIAHLNFDQQYVPRGTTYYDKTIRIDKNPIKTALNVCKHLISRRVYAVVVSHEPTGDLSPAAVSYTSGFYQIPVIGISSREAAFSDKNIHVSFLRTVPPYYHQADVWLEILSHFGYTKVIIIHSSDTDGRAVLGRFQTTSQTNYDDIDVRATVESIVEFEPKLDSFSSYLMDMKTAQSRVYLLYASQEDAYVIFRDAAIHNMTEYGHIWIVTEQALSANNTPTGIIGLKLNNAENETDHIKDAIYILASAIKEMTVNETITEAPKDCDDSGVIWESGKRLFGYLKTRNIRGETGQVAFDDNGDRMYAEYDVINVHEKHSFVKVGSFHYDSEKRKMRLKINDSSITWPGNTGKKPEGIMIPTHLKVLTIEEKPFVYARKLLDDEIDCADDEVVCPHFNITNGNEQEYCCKGYCIDLLKALAQRINFTYDLALSPDGQFGHYQLKNHTTGIGTTVKKEWNGLIGELVAERADLIVAPLTINPERAEFIEFSKPFKYQGITILEKKPSRSSTLVSFLQPFSNTLWILVMVSVHVVALVLYLLDRFSPFGRFKLSTTDGTEEDALNLSSAIWFAWGVLLNSGIGEGTPRSFSARVLGMVWAGFAMIIVASYTANLAAFLVLERPKTKLTGINDARLRNTMENLTCATVKGSSVDMYFRRQVELSNMYRTMEANNYDTAEQAIQDVKDGKLMAFIWDSSRLEYEASKDCELVTAGELFGRSGYGVGLQKGSPWTDAVTLAILDFHESGFMESLDKEWIFHGNVQQCEQFEKTPNTLGLKNMAGVFILVGAGIVGGIGLIIIEVVYKKHQIKKQKKMEIARHAADKWRGTIEKRKTLRASLAMQRQYNVGLNSVSKSVQSQNAEKSRYPILPPLPRTPERAWPKDKEYIVNRKNNSSGAGKPPPRYMPTYATDVSHLIV

>AgamIR75h.1

MKVVVCALFLLGLLTPTRCSSRTQLIKEFLEQRSITSLLLLHCVQELVDQDVIRIASALRQSFHGSVYYLDVTGAHFRRHFQWHMFYERYKTAITLNLECSGMESVLSHLSDNAYFNDTFHWLMFGGRNFEQVTCLLSAQNINYDASIMLVFDRGDRADVPRIFEVYDVRGTVKRRGGRVSFDLLGTVSSLNQLPKRRARSQDLEGIELWTALTTISKHQPRPLIEYLNTVKRQTSYTATIHSYQLVKLLEMKLNFKLIVILTEDWRFDLIGKNSSRGVVGQIQTKQVDFATTPFAITPERIAIFEYTIEIAHGTFYTVFRHPKSLNNSNIFMLPFTNIVWLAISLIFGAVALLIALLIVCIHRTGRSRSTLDWLVEQSLLGTLGMVCQQGIHHRIIRWSSNRVLVLVAMCSSMILFQFYCSFIVGYQLITPPKTINTLEKLVDSEIQMTVENLSYQHDFFRRTNNPTALKLYETKILPNRYGGFVNLSFGMQLVRQGGYAFHCETSYGNALIIETFTEREICELQQVQLYPQRPVHLPLIKGSPLRELFKVNLQLIKESGLLAYHHARYYTPRPKCNKQSSTHTEQIHLADVRFAFVLLAAGMAASAALLGSELVFLHLRTWWHEQQIRAIPAGFRWLN

>AgamIR75h.2

MKVVVCALFLLGLLTPTRCSSRTQLIEEFLEKRSITSLMLLHCVQEPVDQDVIRIASALRQSFHGSVYYLDVTGAHFRRHFQWHMFYERYKTAITLNLECSGMESVLSHLSDNAYFNDTFHWLMFGGRNFEQVTCLLSAQNINYDASIMLVFDRGDRADVPRIFEVYDVRGTVKRRGGRVSFELLGTVSSLNQLPKSRARSHDLEGIELWTALATIDKLQSQSFIEYLNPVKRQTSYTATIHSYQLVKLLEMKLNFKLIVILTEDWRFDLIGKNSSRGVVGQLQSKQVDFVTTPFGLTTERIAIFETTIEIAHGTFYTVFRHPKSLNNSNIFMLPFTNIVWLAISLIFGAVALLIALLIVCIHRTGRSRSTLDWLVEQSLLGTLGMVCQQGIHHRIIWWSSNQVLVLVAMCSSMILFQFYCSFIVGYQLITPPKTINTLQKLVDSEIKMTVENLSYHRDFFLVTLRGARRTNNPAALKLYETKVLPNRYGGFVNLSFGMQLVRQGGYAFHCETSYGNALIIETFTEREICDLQQVQLYPQRPVHLPLIKGSPLRELFKINLQLIKESGLLAYHHARYYTPRPKCNKQSSTHTEQIHLADVRFAFVLLAAGMAASAALLASELLFLRLRTWWHERQIRAIPAGFRWLN

>AgamIR7t

MGSAIIIAIMHLLVLSVPEVAAHLLNAKPSGNPATVTQQASLLTPIVQTHYRLPEGFVAVRVQNGGHSNPSHQQQDLIDGLMRTGHDWLAVSFDDLPAAERRPAYYGVFLVADYRSLCTLLDGMTPGAYQFDGLYTIVIEQRRPKLHDVMERLWSYRLLNVVVIVSEKRANEDEERYVAYTYDPYREHRCGSVEPYAVGQYANGTWTELVRWYAKRTDNFNGCPLVIGTIHIIPCSIIERDGPGGSTTHKGIEVSQVDDLSRRFNFTPEYRISNGSTRWGFARAVNSTGLMGWIQRGEVDFGLGSIGISLSRVQHLRPGIASRFGQLAMAIPPKRPDSSVEKLIKPFSRQTWLCVVLGLAGISTLAWALFGIGWRLVADRLRHPCYTVWVLTMGGPCGALRMDSTRLFVVSLVLNMLVVRTLYHAAMFERLQASASLASELDTLEQINRAGKTYIMHKTITLFFNDNPLVGPRRIRRTLHDNENWEELLYQLSQPGSDFVVTLPLDCIKYYVQQYGNRGLVYVGKHNGITYNTAFFYPQTTALQAPFSARVLAYHSAGLVDQWARAFEDGRYWSNAKADPEPASLAWSHLSGAFYLCGTMHLLAVCVFVAELGWARKWRKASEQPTPRQYHPAH

>AgamGLURIId

MINVMENSESRRKMRLFPMFLHRLLVVAFAFLLIGPTDGYNGDFDEPVEHIKIGGLFDGDTDDAELAFQYAVEAVNNEKLSYSNYQLEAQAVQVKYGDQFDVSKKLCRLLKTGVAGIFGPSLPKSALHVQSVCDEKEMPHIETRWDAYTKLPTLNLHPHPHIMGRVFLDLVVAFEWKDFTILYESGPWLPGISDLLKMYDPKGYTVTVRQLDLGLNGNYRAVLRRVKLSEDKRIILACSIESMPEVLKQAQQVGLLTDHHQIIITSLDLHTIDLEPYQYSGTNITGVRLIDPEEEKIKQVADFLNASQIAKTLELHDGLNPAKMRVKTALMYDAVLLFAEALKHLIGSEPPRLLEPISLKCDDPTTWKNGYSVINYMKSSTIHGLTRSIKFDHQGHRSDFLLDLIELGPAGLEKVGVWNSTEGLNFTRKTEQAAHAMDDGTLQNRTFLVLTAISPPYGMLKDSPIKLSGNERFEGFGIDLIHELSLMLGFNYTFILQEDGVYGSLNRDTKKWNGMVQELLEWRADLAITDLTITSDRESAVDFTMPFMNLGISILFRKPTKEPPSLFSFMSPFSKQVWLYLGGAYMMVSMSLFVLGRISPKEWDNPYPCIEEPEELENQFSFSNSMWFTIGALLQQGSEIAPKAPATRAVASIWWFFTLIMVSSYTANLAAFLTVEQVVSPINNAEDLAAAGGAVKYGAKRDGSTISFFKDAEYGTYAKMYQFMMANQDLLTSSNPEGLQRVKTENYAFLMESTSIEYIVERECDVTQIGGLLDDKGYGIAMRKNSPYRSALSEAVLRLQEQGVLTSLKRKWWKEKRGGGACENTMEEGGALALELANVGGVFVLLIVGCVAALFVSFCEMLCDVHRRTRELKQSTSEDGAEELDMDNVGGVFFVLFVGCSFASLFGCCELFFVIAHRARRHKVPFREELMAELRFVAKCHGNTKPVRHRKSSSASGPNSLESGMESAEQSATSSKQDISGSPTGAAGADEADRDAELENRQRRQNGGGKSALNGSARKIKSDE

>AgamIR41a

MNYSVELSELTQGELFHFELNRLLKWIFLQHLVSFFCTCIVVRTGSSGHWTAATEQFPHPVMVVALSRNDELDVLLDAIENGCQTFIVAQSTAIEFLDAFRYVHDRATVRYPHKRVIILTDDADVQWRRTVFEHGAFRDVMDWLLVHPLPDGKRVDLLTTGYGYDEWIPLASHDTSVPGVLHLTVELFPNKRTNLWGRYVRLAIFNYEPYTLWSAVDDSNDGNAFYQHNRTLFIDGTEARLFVEFCAKLNCSLEISLDEAGEWGQIFDNRTGDGIIGAVVERRADIGVGALYSWYHESLYLALSKPISRTGVTCIAPKPLPLSSWMTALLPFSTEMWLAVLGTIAVSTVCEMVVSFVTQKFNPNKVHRVDACESIMAIISIFILQSVLIRTTRNPYCSQMILIGSILFVGLMIGNAYSGGLASVMTVPRFEKSIDTVQDLADRNLRWGSTHDAWIFSIQLATQPTIVKLLESFVTYPKDVLHEHAKQRNLAYSIERLPYGHYAVGDYITDVVSSNFEIMLEDIYWENCVAMATKTWPLMDELDELTLRIFQSGIQRYWELEVVTKFADNKVQHAISTSRHFGNPGPIPLQPSHLVGAFFLLAVGLGLGTVCLLLELLWHRLTATNDGAGMARRSETP

>AgamIR41n

MENQNPTHSLLHQSIIASDDIVGLNSLVNYLVLTYFAHFFGICLIIARNDSFHHSAPLPTIVLVIQDNDDFEKALGVAVDMGCQVGMEISFIVTEHAAGAFFDAFLPVHEAALQRSMEKRVLMLLESSDSPFLSTISQLLVVEVQATGAVQLYTLDISDGNPVTISPRVIDVIDTRNDSFPNTKSVNHFPDKFANMNKRRLRLGTVPYLPNAFIEDKPLGEGNARYILPAKPNVSAMISGTELWLVVLFCEIANCTTEIMIASEWGNVLDNGTKFGLLGAPAKREVDITLAGLYTWYSSFQHLAFSAVHSRSGCTCIVAKPRIIANWRTPFLSFTGSLWGAVLAAFVAGAFAVLVMSRSRQRILQLGEATRYTFSDSVLIMIGFFMEQGVPMPNELVASCLLFATLMFAGFMIGSSYNGGLASTMIVPQYEKSINTVHDLAETRTTWVGVTVNWLFSIQLAYQPDMLTLLTTFREWEEGEISRRAHERTVAIIVERMEYGHVAHPQMELDAMKGRKMMAEDIYWESVVGMCSKTWPARARFDRLVLDLKAFGILAHWELIGVARYLSFKSQQILRYSRETGGDEFTPLRMANITGALLILLAGLSLSLVVFVVELAWYWCGPRIKRCVADCLIKCSMRFARKRARPTGREV

>AgamIR40a

MGVGSNSKYILALVLLRVALVWGAFPTQRNLIALYERSNQSGMIRGISEMVNLLAPKSLVILVQNETKIDRLDKLTVMIHHHNIPTCVYYDLEAYFSLIEENLKKSLEITSLIFCHPEDMLQDITDRRLAHRLSLFIFYWGAAQLPPTLNPNLLMEPFRVAIITNPRRNIFRIFYNQAKPNNRGDMLSVNWFDGNDMTFKRVPLLPSPTEVYKNFEGRIFTIPVIHKPPWHFIVYGNGSASVGDNQNSSSSDAAGGFELELDENVTVESDDTYFTVKGGRDHNLMQLIAERMNFTFQYVEPPEKIQGIALGSEDNASFSGALGMLQRREVELYLGDVAVTWERMKAVEFSFFTLADSAAFVTHAPRKLNEALALVRPFQITVWPPVIITILISGPILYIIISTPYRWRSAQTVHARNARWRPTRSRLRKPAFYNLRYIEEMSYTRFRAERTSLINNHHHSRGQDYPSLDRCIWYTINVYLRQSANIPFDGHLARFFSILLWLCATYVLGDVYSAQLTSQLARPARESPINTLGRLENRMNREGYQLLVERQSAFHAALVNSTGVLQRLYRLTRQRSVNDSFLVKSVEEGIRVLQADPKYAVFGGRETLYFNTKRYGANRFQLSEKLYTRYSAVAVQIGCPFLDSLNEVIMRLFEAGIVEKITIAEYEQMFGRQKGGVSHAEETVRTVKSTNSECDTDGTGSGKRKTDSNDKLQPMNLRMLQGAFLVLACGHLLGGICLFIERHMGMINPCGDTLRQGWRHLNRVVRKLGRGGSFKTQSN

>AgamIR41t.1

MEATVAQLGLNSGVPIPTMSVLMRFLILKYYSQYYSFCTVGNDFLQDVPLSRIVLSLYDQLNTSMLHAIDNGCQAFVVDEQGAERFLDLYLPVHDLARQRSLDKSLILLIAKRNQTHLFNRMRVHEAFRELANVLIVVYDEQERPTDLYNTVVRISDVLERSVNVAMELVAVKWTSWKNHSFFPDKARDLNGYVLRTSMCNYLPFTGYERLDQETGNAFDSTTGKRSIWLDGTELQLLVSYCERRNCNIMVFPEDEDEWGDVYPNGTGIGLMGSVAERRTDIAFGAFYLWFKPYNFSSYTATISRSGVTVLVPKPKLLPHWRTPFLSFSWPLWIAVMVTFLVGIIATWLAGTVRLRLLLLSAPTDQPWTGRSDLISEQLTLSDAALMMVGFFVAQSSPIRTDLWLCVCLFASLLLAGFMVSNLYSGGLASVMMVPKYEKSIDTVVDFAATGMKWFGPTPYCLEEIRNASEPHLQQIQKTFRSVGPEEMKQYAHMGGSGFFVEQAQHGNFAPSNFLDRQVSTTLQPLKDYVYTQNCAALITNTNPLRSNLNEYILRVQQSGLLYHLGIRTAIRYLPTDVMRNIERSRMHQHDDGAVRLELGHFLGAFFILGYGLLCASLVFVGELWGTVMMRRWNETFVSKATVVTAY

>AgamIR100i

MFTKGFIVAITLVHMWLGTSCSLERMLPEDTLIGPLVEQVMRSLLLRYFRSARLLLLGCYRFSLLEIPLLTFTHELADDGFMDWIQPTVEDNDQPIQRMNSTGFLSARDNLFVMPDHAEKLHFFNPFHAEETEQLLTFYVGEDFAMSPTADNKLNIFNGIPIRCTVFPRNPTLLPWDSLPASFREVHYVQQSVRASNGSGGLDGMLLGNLAVALNFTAETMDASDGQEYGYRLKNHTFVGSLGDLLQYRTDVSFNVRFMKYYDTHGIEFLHPIYSDQLCILSPKSLEIPQWLAIFLCFHPYVWTSFVVVGFAGGYCWYLLKRWTLRKVSRYRQHLLKGDRAQYTVLSIEMWLVLLGASSTYLPVRMIERTLLVAFLIANVIISGTFQGTLTTAFSTKSYYKDLNTLAALDKSELPIATSSRSLLDIFGNDSLSPLYQSLKGKLQILNESARHRAAFQRDVCCIERHSDVHLIINTEYIRPNGQPMLHVVDECPRVYSLAYIVRKGWPFAPLFNAAIYRFVESGLCMKWYEDTETALILQKRIRQLREQEEEPALRKLTMIDMQTSFYIMGLGMLLSFSVFIMETFVGRGLKCSQL

>AgamIR64a

MHRVRSAMRLMGHKRMMLSSWIAVPAILLAAVGLACGYAVTSNDYSETVATFVFRFIRDHKGLKLGIVFGCDQKLLLIYNVLSQLATIGVRARMVHIGPGPTPDRNEQEHERFSSTLRQSLEFHQFVLLDMACENAPDVLKQVSRLFIHPNGAGSGLDRLPAHACHAHVKPSVVGTMWRVCNTIRYCILPIKASRYELFNASFHWLIIDNHYNSVEVEGNTGENGLFTCPWTMITNSTENGGTAKPIQSFTNGTEPDGAMDSDWTASSNGSEATASGHTFDLLATMNVSINAEITVASPTDPSYRGFTLYDLWNPGFISGGKLKIEPLGCYSPAEGLQIPKRESTVVRRRNMDGLRLKIMTVVTQKPHQPFELYLTTPQNTHLDSVHRYNYGLMGMLKEFYNFTFVNRRTKSWGYLRNGKFDGMIGALSRREVDLGGSPMFFRQERHRVVSYTTRTFVERPCFIFRHPPRNDAVKNPFLLPFEIIVWYLMVGCGSILVTVLCFSFFVEDAGLAGRNVSPETCLNESLLNRPKLSPMRRSYWHRETTGISYNVDEDDDVQGSKSASANVQPDIRSPNAAGSSRQRWKICHWLYPVDCRQLVPCNSDAAAAAAVGGGDGTLTARNCHNKTVPANISSTHRFHPGDGNAGAKCNYKAEKVSKSILLFLGGVCQQGNLANGVFLPRLSEIPHLSSGRCTSFFILLFGYLMYQYYSASIVGTLLMEQPKSIKTLRNLIDSRLTLGIEDIPYSRDYFVDCFISMDPAKIVLQKHTRTNQKNRLPHTPNYQRTTDADSLELHRTRIEYYEPKTGRNESNFLAAADGLQLVREGGYAFHVAISAAYRIIRLTFSEREICELSEIDMFPVWSQWMVAIVQKNSPYRDVITYGLRRLNEAGLMQRQRHVWQEAKPKCVRQIAPTDLIVGLDAVVSAFVLLCGGICLSVCVLFVEILCHRIERHRHGCHRRELWRNRFVMMTKQPSLPAQPRRDGTELFYVE

>AgamIR75d

MHSYYDETISHQQEKRDDREQNRLAVGVYSVLGTLLCYVGHVEPKSKHVKSVTVIRCWEAEERYDFFLAATRKGLLVKFVDATNIEALIAIHPNRVSQAGLLIDASCEIEPISKLWQMDQILNKLLYGNLHWLIVEKQLPVKRSIDSGPYSSDQLELLYNERLRPLDVMPYTNVVLAFLNVSDSWELFELYKPYRKARLSVVNIASNYLPAVDRYGSQLIVRRKLVDRRRNLQGFAMPCGTATTSPEYFTGMDDRNDVHDLFTKANFPFIRELMYDLNFTLNMVQLDKVGYKQNGTFSGVMGKFQNRSIELGCLGTLMRTERLEVADFMIVTLIIKSSIIFRQPPLSIVANIFELPFSVGVWACCFALMAIYWMTMIAIRQLTSGERFGAIESLVYIIGTMCQRGCDIVPQFNGTRLLMFSLQLTSFFILTSYSASIVALLQSPSRAIASVGDLVRSPLKAGVMDTSYGRVYYQETQDPDVQELYRKKIKPHGEKAFLEPNEGIGRVKQEMYAYEGELNAAYKLIKETFAPEEVCKLQELEAIKLPPFGIPIVKGSKYRELIRQRLMWQREVGIIKRFNLIWIHQKPQCENLNAGFSSVGVVEMRYLYLFLAVGFLAALATLLAERSWWSGMAKKLKSRTAIIRRKNEA

>AgamIR75l

MGGRDKQDLILPLMIAPLLLSGYSVAAAVDASMVGAVGALLKLIGTPLKVTAFVECWNEGEKLMFARIGLSGGRHLMQFVEPRRASPDARTDVKDMDNYRNATLDEDAESHQTLVVVDLRCNGSERLLAEAGQRLYLNYRWLLMDFSEGGAPLGIEHYLAVLQDLPALVSSEIFVMLEEDGQSIRFMQVYRVSQNSELLTEHYALWNATVPGDTEGEMIDLRTHKVTSVRRKDLHGHYLRASMVITNPDTLNHLTDYKDKHIDTITKVNYILTNCLVAYLGAEVNYTRVATWGYYNTTTGMWDGMIGELVHNTADLGASPLFFTTDRIAVIEYLAMTSETRSKFIFRSPKLSYTENVFVLPFDDKVWICVIAVIIVSSVLLLVTLWAEWRITNGDLDMPPAPPDSSTMAASLRDTLLMMYGASCQQGSAVLPRSCSARTITMLTFTVLMFLYASYSANIVALLQSPSTKIQTLEDLLASRLKFGVHDTVFNRHYFTHATEPTRRALYEQKIRRPYGPDAFIALEQGIDRIRHGLYAFHVEQGVGYKVISETYQEDEKCGLQEIQYLQVIDPYYAIQKNSSYKEMVKIGLFRLNEHGIQYRENDKLYTKKPTCSGGGGKFVPVSLVDVEPAVWIILWGSGLASGFFLTERLYFRFLRRKVRQLVRRWQHN

>AgamNMDAR3

MFALPWKRSSTLLVVELLLSLLALAGLTVPSSASSSEETLDADATVEFAGGPVAAEMLKAHPAGGAFTVDDGSQPVFPERPDAVYFAVAVTGGAKLWGRTLARTLIDMGPPFGHPQGPPLRPIYINLPDSGRFSSKLMTTACDSIDGMPLSGMVIVGDSPAAKSLALAGNAMKVPVLWAKGGIATLHNSYDEVHWHSYHILCDVDTYVLISGKKGAPLRQKPLNPIIVTLPTNFDLIYKKLAFISRSTKGVVLVLCNLKVARLIMAEAQRMKMLNGHFVWLWMDTTSATEFYDSGQPSYDPKQQQPLDYPTAEGSEMKLKRSSEYLKPHNSSGHVLYHHNQDFPVGLLALRPIKMKIDRHFIRAAVRLFAATWAKVERDDTPAAGGGKGRGGPSGRDGLAGKAKTMFRVVVSVAPPFVMESSVNEEGQCLRGLICYQIYTTGRHNLTLMFNEIERRNRLREIQPASSLHLQEEPRQKYPLYRTRCCYGLSMDLLQKLATEINFDFHLYIVHDGLFGRRVPPPPEPTEPPTNVRKPAVATHVESQSKFDAQSGRSLKRGSKLKLSQAGINATARDEGPQYPFEVPAAPPVRTRQLWNGVIGDLISGTADLSFAPLSVSKARSEVIDFTIPYFHGGVSLLAAPEAATDIPLLAFLLPFSPELWIAIFTSLNVTAVAVAIYEWLSPFGLNPWGRQRSKNFSMSSALWVMWGLLCGHLVAFKAPKSWPNKFLINVWGGFSVIFIASYTANIAALIAGLLFHNEAKYYEMSMLTQRVGSPIATAAESYVQQNDKRLWEHMKKYQLQSIDEGIERLKNRTIDLLMCDTPILDYYRGTDQSCSLQRIGDNYIEDSYAIGMSKGFPLKKTMSALISKYSHDGYLDILTAKWYGDLPCFKLDREMAQPKPLGVTAVAGVFLLLGVGMVLGVLILIIEHVFYKYTLPILRHQPKDTIWKSRNIMFFSQKLYRFINCVELVSPHHAAKELVHTIRQGQITSLFQKSVKR

>AgamIR133

MCLFEIDCKSPIQRGGGRGGGGRISTKSNITMTGPPRSTGPLAAAQSAHLETLVNITKGIHKIETPSAKYELCLVTHATDPYWSSVVVGYLSHFSSYPRVFLNAVQRNVQLHRCSVYLIVAPLIEGKQLYGLLQNVSAGRNWNPAAHYIVAINDRPTLTSLVNVFRAFPPLGIRNGAVVRYRRGIIDTLVCDYNTSRVYVVTGTDAKHRSWLTHDRNRNLDRLPVRIKTSMEFPFLIVSQKGIAGVYRWFFVAFAKHINALALFDRQPNTTRYEIALKIHDTYRDTLKPVAFGSFSGDCVVLPEVSKRGLFYFLLLPFTRPVWLMCGLLATGSFLLNWLQAQRFPNALLLTLLFGDQAQRYTLAERRLLTVGSVLLFTLTEAYWGKLHALFMVSLNEPHLRTVEQFLRTSVPLEVVHRDAASYYHELHRHRQLIVPETDAQHMDNVQNGRCALLMPCSNARLLLHLLLNVNPDVYRVPYYPLEQPVRQHLDGFSVFRYSPIADQLVQFVGTAQQAGLAHYWRTIYVHQLEGIARRRILVQETLGWGELASLLYLLFAGYAIALVAFVLELWSDRFRRVAPNA

>AgamIR134

MALQAQSDDTLSQSVLITLRHVTTRAEFLERPSAGQELCISTSATHPHWNDVLQAYLHEFPLYPRVLTSIEIMSDVKLPKCSLYLLFEPNASARQLFLLLKAISSNSNWNTPAHVILLTGWIERPDQFKTLFKVFYTMGMHNVLHLVANREGNDSAIVACIERGFIQSFRPGSPHYRQLLADRTSNLHQLPLVIEKKVVYPYLMYTRDTVSGVYRQFFDAFAAHINVRVTFDTTDNQILLGIKAPNALSVPIAADGAFTGNCLVVPEKPKAGLIHYLLFPFTAPLWYLCCCLAGATIVLNCRCPARFGHSILLTILFGDQDTRNSYGRTERRLIFFAVLVMFFLSEAYTARLLSEFIRSLNEPHLTTIRQFAASGITLELPSVQRDRATLLTEELRHNLLVSDKRTYLANVRRGQHALLLDCGNAQHLVHEILNESEDEFPVRYYILQEFVGWRINGWSVSKFSPARVQLTQFAGRIAQAGLWNCWHGLYVKRIHSLRSRSTRYRKTLTMRDLISLHYLLLAGHGVSAGVFVMEIVTHFALIACGRLRKSLIKAKSQLQRMRH

>AgamIR135

MGNMGGVLGTVSAQFTHSKLRTFAMVPPPFDWALLLVRFGLLAADSSPSADAALASLAYLTAHPGTALERPAAGQELCIAPPPDNDPYWNDVLERYLHHFPLLPRVLTRTKLGDNIPLHRCSLYLLFEPASHARQVFLRIQFLSTNGNWNPSARFAVLINARSHTSEIFNQFENFDLMGVRHGLVLMSVPEHNRTFTMLANCRQRTLDYVMDVSDVGELLANHSSLGLQQSEPIVVARRAEFPFFIHDRSRIEGVYCKFFTAFARKYKARVVFGDRNVQIVMTIHNRETLTQPYAIGSFTGNCVIVPERPKAGLVHFMLSPFSSPVWYLCCCFLAAIFLLNWRWSHRFQHNILLTVLFGDQAADERYSLPERRLIFLAIVLMFFFSEAYSAKLLSTFIESLNQPRIRTLRALGASDVPIGVLHYEDVEGYEQLHRNLEVLDEPEYYRNMRAGRNAFLLQCGNAEYMLHKEMRDTRVFRVPYYILGEMVGWRMNGFSVSKFSSIAGELVEFIGRIGQAGLWEYWREQYVQKLRNVARKELSQRETLLMGDLISLQYVLTMGYGSAAIVFGVEVMMGCVRRCKRTRRTVVPLRAVGGH

>AgamGLURI

LTGAIFEQGTDEIQSAFKFAMLNHNLNVTARRFELQAYVDVINTADAFKLSKLICNQFSRGVFAMLGAVSPDSFDTLHSYSNTFQMPFVTPWFPEKVLTPSSGFLDFAISMRPDYYQAIIDTVRYYGWDRIIYMYDSHDGLLRLQQIYQGLRPGNETFHVETVKRIANVSDAIEFLRTIEELNRWSRKHIVLDCSTELAKDIVVSHVRDITLGKRTYHYLLSGLVMDDRWESEVIEYGAINITGFRIVDTSKKYVKEFLDGWKRLDPTTSQGAGKELISAQAALMYDAVFVLVEAFSKIMRKKPDQFRAYTMRNRGQPFNLPANGTRTLDCNTSKGWVTPWEHGDKISRYLRKVEISGLTGDIRFNEDGKRQNYTLHVVEMTVNSAMVKVAEWSDEGGLAPVVAKYTRLKTDMHYERNKTYIVTTIIEEPYIMLRQPEPGETLETNERFEGYCKDLAELVAKKLGINYELRIVKDGQYGSENPDVKGGWDGMVGELVRKEADFAIAPMTITSERERVIDFSKPFMSLGISIMIKRPVKQKPSVFSFLNPLSKEIWVCVLFSYVGVSIVLYIVSRFSPFEWRLVNYNVVNALSGGKHTAHLPCKSTDKSRNLSHMFCFLPLLPGTTPSVPTLTRCFVTHFMANADPREQPDAVPQATVNEFSILNSFWFALGAFMQQGCDISPRSISGRIVGSVWWFFTLILISSYTANLAAFLTVERMVTPINSPEDLASQTEVQYGTLIHGSTWDFFRKSQISLYSRMWEYMNSRKHLFVKSYDEGIRRVRTSKGKYALLIESPKNEYTNEREPCDTMKVGRNLDAKGFGIATPLGSPLRDPINLAVLSLKENGELTKLVNKWWFDRTECKHYDKQDASRNELSLSNVAGIFYILIGGLLVALAVALVEFCMKSSNRSSNRIPLSDTMTSNSNSKNRLTMPPTAREYDNGRIGVSKAHPLHHPYHHTRTATGPY

>AgamIR142

MLPTMNDFSSVRSTTMNTTVLFSVLLAGLLQSVSNKHSSLYATVGRLTKEAYRSRFHSVKQVYAVGIPDEAFGEWNAYVPMISLQEPYTIRWKLENAFVIVKISKFLTLNVLSAILSHARDVEKVFLAVIIDSPPSHHTLKQLTGMLLKRGLLNYCIIHTNTHGIEPSLLKYDTIANHFQSHDLNTSIELLFPPFLRTLEGHKLNALLSDNYPYAYLLSSVWEGVDAYILTLARESFHFEVQLINMAINRQNALHRMDLIRQRLENGTIDIHMPRAHVSRSYLNVDVAAAYEWEALVLVVPKSDQLNLINIMLQPFTIEVWTIVLAYLLVRQMIKLFSFFKRRCNRFTLKKTLTCRWSFGSFGSLTTVGVELVSFLLIEAYLAKITEFLLYCRFRSDPQTLDEFFRSTIPVLVPEYMDPLVEALGPTVAANFHAKLIRPDEYAKRAASCCARIHTLPRAEYVVRMGKYFDATLGRKQLYILPEQLTIIPMSYLVGRNFAFKNSFELFLLSVHESGLIGRYVTAHRKDMAMMERNFFTKGWLTLADLLPLFVLVGAGWCCSFAAFLSEVFGVRFNRYWARKRVRPFVAE

>AgamIR136

MSSPNSTFVQMFANERSTTAALLISVLVRIINKTDRFGSNAVALFNFNTHCMDHVPDQLLKQISTITLLNYDSSPLIKDTFERPNFFLHIHGYKVAANVTTNDIITQQVKMFYLYRLFGITERVVVVFDQTVPEPNPLHKVATLYHRHGVINVIYIVMFRAQLLVFRLDRQYKAFIEVPITASLQTLFPDRLSNLSGQPYMVATYENPPKSYADSSRRIVGADIELIAIITHHQSTFAFFNYSTKPGPIFVPWDDRTFDFATYRIIYKGAVQYPFSSLLFPDRQSSCIAVPRWFNRVLQEQVIWPFALDLWALSGGLAAFYVWYLLVLAPHLRQHRRDMYDLVNTPLHIFRIVLLFLLTEYYTALLTSSLGLSQVPFYPTTIQEFVKTPTPLLVLRRDLISILQENKDFARKMIFYENIEQYQHGQYALTQLCDMFMYTIGSITKYLGKEMSYRHYHLIDEPFTTTVTMVPFGKLNPRLKRFQMYVNRLNEAGIWTYLVKKWNLKASGTQVVYEPDDVDALFLSLEHFVPVFIASGYAYLITIFVFLLERIVYSIKLARSKKLFKRNKKIVKLRIGKV

>AgamIR137novel1

MTLIGGILLEPATADSLLVSFLIRVIFELHQDHPNPSNVGLVNFDSQNLSYIPTELMKHVTDVTFLNIDINEQRTETKAIHYAPILVHAFGISKPNGPSEESPIQSEVLNILEYFKLIEKTKNIIVLLDAYGVKPTELDLLAKTYHHFGAIDIIYVLLKMKEPIVIRLNDMSTEFVKLSTFARIEQLFPDRLANMSGRPYKVACLENPPLSFRSPATNRTIGIDVEFIDMIAKHQHTVADYRYTAQPIELFEPWHSTEIDFATYRIILTEQAYKFALLFLPNQNLWCLAVPKTYNRILHQQILWPYTTDMWLLIGTIVICFLLYRLILKHTLQRRHPNAFPIINTPLHILRILLLFLLSEYYTALLSSNLGLSKLPAYPRTFEQFKKSTIPLIVHRPESYEFLRNNEDLMSRTIRWNFSQRYDPTRLAVVQLCDLFPYTIRATTRLVGKELSHHHYHLIEKPVKCAICMSPFRQTSPLLSRFQMYVRRLYEAGVWDFVVRKWTSFTMQPMGFEALDSSMLKLEHFAPVYIVGGYIYVLCVGVFLLEIITHRILRRLNGR

>AgamIR138novel2

MDSSLIYTQEGSATSGLLISFLVRIINELHCDRCSQSTVGFVNFNTPHSFSYIPSQLMQQLSTVTFASIDLRQDGCQLQSLNTPIVIHAVTSFQWMKNKLYILKDVLDTLQHFGLYGKSKKLIVLIDMNRVTMAELKVLKESYQHAGAIDILYVLEHSNFNQLNIMIPTRHNRTTLVHRSAESSIEQLFPDRLSNQSGQPYKVACIENRPLTYRDASSGRIIGIDVDFIDIIAKHQRTVAQFKHTADPIKQFKSWYDVEFDMATYRVPDGGLAYPFAPLYFPNQFRWCLAVPKTYDRVIHDQVIWPYQPPLWAMILSLAAFLIVYRLFLRQPIQHQYPDVFPAIDTPLQLLRMLLLFLMTEYYTAKLTAILGLSEVPIYPRTLAEFSSSPIPLLASHRSGYQYLIDNPQVHAKTIEWNFSAQYDPTGMALLQLCDLFPFTIGDTTQIMGKRLSHHHYHLIDEPISTSICISPFRKTSPRLVRFQQYVTRLNEAGIWDQLVSKWMLKDGRVSVAHGADKRTSFRSSILELFHFVPVYVIGGYLYTSALIIFILEHLVYRLQQRF

>AgamIR139

MSSRKIVLVSLILTFYSVHTLPKRQAGIVDYLAHMAAALQMQHFGVFNCWLLQFSNGTNLSPLLTSIVQRLSNEHISLVQADHSGRHIPTHQEPNMVIMLWGNEQQMLDQFYINQWLWEIPSDCRTIVLFELDASGASSLPWQIGEYFESLMLYYVACIAINKNALFSFHYQPLRIVSHSCFPDLSELFFDRLQTIQNKQFAAGYMKDHYTAMYCGQMYGEDTALFLLFVDRLGLTLQLQEVLCDGFESLVSCVARYNGIHFLLNRLYFTQYNKHVISASAMEQFTIATPKGRLLTVWEILLKPFHHSAWGLILGILVTLQLINQLKPTLFSNNLLALALFGFEKHQLRLTKRVEKATAFALIVLFFQLKCAYEAKLVSYIAEPPRLPDAASIEDLRERNIIVHSRKINIMEDDKLNGIVEFYDGVHFKFDGLTLLENRVALVLEKLFADSVEGHGMLYTILQENVYETLPFYALGAKSLLRRRFQTFQQHVFEAGMQQHLRQKQASCLIWYIVKSKKIPGAFDGSSAVIRFDHLKPLMLFFLGQWVLEVMVFMIEMLVERYKRSRRV

>AgamIR75k

MDKSRYLQFLVQSLIVLKVLTVSNGTPQATAVNPLSPQTQTDQRIAAVRDLLVHLDRAHQLLVLTCWSPSVRYALWQSVRESTATSHRGTASVRFAPIDQRHLPWHDPNQHQIVIVLDLSCPGTDRLLESARQLLYYRVRWIVFRSSIEESGGSVRGWSNCSEYSVLDRLPLLVSNELFYFCTETSTGQHLVRQQYRLAARSASSSVPIYETFGTWNAALGVRVVVDSKVRPPVTSIRRQNFHKFQLRASLVILHNETLNHLDDLHDKHIDTLSRVNYLLTKSVAHALNATVKFSIVDTWGYRDRETDRYNGMIGELQRDLADLGGTSMFFTQDRIKSVDFLAMTASTRASFIFRAPKLSFTSNVFVLPFDQYVWYCTVSFIVLSGVLLLVMLRTEQRYTAGGRGGRGSAIGSNSPAAVTGLSDTLLNVFGTTCQQGSFIEPQTAPSRCLILLCLVVLMFLYASYSANIVALIQAPSTKIQTLEDLLASRLKTGAEDTVYNQYYFRTETEPSRKALYERKMRNKDGTENFLPLAQGVELIRQGHYAFHVERGVCYKLISETFQEEEKCGLQEVEYLKVIEPYYAIQKNSSFREPVRINLFKLREFGIQGREHTLLYTKKPRCIGGSSFIPVSIVDVWPALVTLGWGYLLTVAVLIAELLWFRLRSRILPTAGYFQ

>AgamIR68a

MCKTSRLVWILTAFVLILGVKHCANKQLNSATKGNDRKSSQSIHHEEYSTELHLEMLLLELAAKMDYGHCYVVLFDEVYESVLNAAFFRQIHRAARYIVKIEQDEDTFNPRPSLKCILESTRKAGCGGYILLMANGIQMALYELSTAPFPMQIKGVFFSKILNFWQGGKFRLANSTFFDDKTKDLRRQEMRVVVLEHTPAVFKSATTSNYYGLEIELLKAISKAMHFQMVFYETSDADKERWGRLGGNGTLTGIIKEMQEGKADFALADLHHTEYNLGFMDLSVPYNTECLTFLTPEALSDNSWKTLILPFNGEMWAGVLLSLFAVGFVFYAFSNTLMLKWLRHKKPKTNMSKSSAYDRNKLKKLRMIPFKRQPEPWHDPLPANDMFDTFSDCIIYTYSMLLLVSLPRIPEKWPLRMLTGWYWVYCVLVVVAYRASFTAILANPIPRVTIDTLQDLAESSVRCGAWGEQNRLFFQMAQDQYSQTIGAKLEHAPNQNEAVEKVSEGLYAYYENIYSLRQLRSTRKSEKARQTLHIMQECAVHMPISIGLGKNSPLKHQVDLYVRALIEGGLTRKWLSDAIEQFQSNVEIPPQEAIIDLKKMYAGIVALCFGYVIALFAFVVEKIYWRYYIENNPAFDKYLHGIVFRGRG

>AgamIR21a

MFKRIVLAVINLVFLIVSTTAFASLHYPESFNQQLIRYKGRDSSNGYFELASEAYYTGTYDLVEELNETATCLRADCREQSNSSPVVRDRRRVDPTFYGHPKTREQIWERNFAHVIEDNRQTLSLVTLLNKIILKYLHSCIPIVLFDTYVATTENYMLEALFSDFPITYITGRIGPNYTLDNPGILEPTGPQCRSYIIFLADVMMTRKVIGPQMNSYVVLIPRSSQWKLQEFLAAKQSRDIINLLVIGESYSVDKRINNEQPYVLYTHELYIDGLGANRPQVLTSWIGNKFSRNNVNLFPRKLRKGFSGHRFTVKAAHQPPFMIKRLSTDGVGNVNIRWEGLEMRLLRVMAQYLNFTYDIIEPGRTELGPGDAVVEEIKRGQGDMGLAGIYVTIERNLATEMSVSHSTDCAAFLTLMSSALPRYRAILGPFQWPVWVAVILIYLLAIFPLAFSDKMTLRHLLGNWSEIENMFWYVFGTFTNSLTFQGENSWSNTRKTSTRMLIGIYWVFTIIITACYTGSIIAFITLPVEPERIDGIEQLSRGFFRVGTLDRGGWERWFLNSSHKQTNKLLKDLRFVSSVDEGIRNVTEAFLISYAFIGSKGELEFLIKSNLSHQFENKRYGLHVSRECFALYGVSMVFPPNSVHRDPINNAILYMQEAGLIGKLNRDVTWETMKTKDGRRKEASVGEVLRSTAPSERGLTLADTEGMFLLMLFGYVVALGVLISEWVGGCTNKCREVLKERAERLKAAAAEIAAAATAGSDNGSLPVSSPTSTNRNSPHKRTGPNGVENSLPASGNGSATVRRIRLTGEDSENEPNEYDVPPDAASDGGSSLQRHSLSECLSEVSAHTMQDLYNGPDRRHSTIVFLDGQLMSEEEAQRKVARSKSRHRHSLSSVLEREVSQLFRFLGKESPHSARADESSDAGGLVRRGAGRERKEMKVAVEINARATEEGQQGPGAAVGRRSIEATFGEKLLH

>AgamIR41b

MELSTDLLHFTSAFQLFLQTLALTHLSGHYSVCVVRSITDDVRLEFPRAIVTVTLASPANTTDTTFGDRLVRSINAGCEGFMVLEGALFPFLDHFRSAHEAAYFRAHNKRIIATVRLGPVARERLLQHDTMEVTPNMLLVEVNESEGMVGLYTTKLSPQEPRGIVAELMLLERLRLGEELLLGLPESIRKFPNKLTDMEGSRVRLSTLPYPPCSVANEVPLGEGNARSTVPANYSLQADGTEILMVLELCRRHNCTLEIELVANSEWGQVYPNGSADGLIGSLIDRRSDVAVAAIYRWYNWYQYMTMSAYTGRSGVSALVPRPRLLPYWQTPFLSFPPSLWLMVAVSFCVGTVAVFLTEHARHHIRPLSGSTSHNNRLIDSIFFMVSLYVEQSVPLPNSLLAGSMLLTFLLFGGFMIGNSYAGALACVMTIPRYEKSIDTRADFAASGMKWSGPTVAWMNSLLMAEQPELVTIRDRYEVHGGDTLARYSHTRRDMGYVHERLQYGSYALESFINLNATRLLQPLKEDVFWEQIVTACSKTWPLMGYYDDLILRVQQNGILRYWELGSVIRNMGLEIQRNLANARVQDSDHEPVKLRMAHFLGVFFILFVGLSLASVIFVAELVVYRANSNLTTKQKVQVISVD

>AgamIR31a

MNNTVSWLLLSRNTTADLLPRVLWNTAGIQMNSDLVTAIETTDHRGVFNLFDIYSKGRHLCKDIFQTLLGTWSADGGFRLTPNYSPYKIRQNFNFLQLRGVTVIDRENVTSGKVDQLLGEPGLTKGIVAFVKYHYALLVVLRDFHNFTIKFRPTRGWAGRLRSGYRLGLLGVVQRHETDVAATGIIMRLSRQPELDSIHYSWAFETGFIYKITPDIGSKSEGNGFVAPFSLPVWVALLLSLALSVLVLQYLARLSADERNTRATMAYVLDVMACVAQQGVPNVSRLVPTRVAVIVLLVANLVLYNYYTSTVVSGLLSSQMIGPETIAQVIDSPLLLSLTDTGYHRILLREQTLPYSTRMHERKALPPRSPNDLPLFTDVEHAVPYLRRGGHVLHCELTEVYPAIANQFTANEICELRTVEGLYRYDIRVMAFVLPKHSMYSELFKITLMRAQETGIVKRIYRIHKIAKPICQGSATVYSVELTEVSLAFIIL

>AgamIR25a

MDPKNGRRWLVLIPIQLASYAIIAIMGQTTQNINILFVNEVDNNLANVAVEVALNYVKKNPQLGLSVDMMYVEGNRTDSKDLLQALCSKYGQSLSENRPPHLLLDTTLTGVSSETVKSFSLALGIPTVSASFGQEGDLRQWRDLTPTKRGYLLQVMPPADMIPQVIRSIIIYMNITNAAILYDNTFVMDHKYKALLQNIPTRHVVTTIADDRDRASQIEKLRNLDINNFFILGSLASIKQVLESAKNEYFERNFAWHVITQEQKDLTCNVENATIMFLRPMSDSSSKDRLGSIRTTYNLKQEPQITGFFYFDLTLRALIAIKNILQSGSWPSNMKYITCEDYDGTNTPNHTIDLKTAFIEVTEPTTFGPFEIPKGGKMQFNGNTYMKFDMDINAVSIRSGASVNTRSLGTWEASLNAPINVANEAEIKNLTADVVYRVYTVVQAPFIMRDPTAPKGFKGYCIDLLNKIAEIVEFDYEIREVEDGKFGNMNENGEWNGIVRKLIDKQADIGLGSMSVMAERETVIDFTVPYYDLVGISIMMQLPSTPSSLFKFLTVLETNVWLCILAAYFFTSFLMWIFDRYSPYSYQNNREKYKNDDEKREFNIKECLWFCMTSLTPQGGGEAPKNLSGRLVAATWWLFGFIIIASYTANLAAFLTVSRLDTPVESLDDLSKQYKILYAPLNGSSAMTYFQRMADIEAKFYEIWKEMSLNDSLTAVERSKLAVWDYPVSDKYTKMWQAMLEAGLPNSLEEAVQRIRNSTSASGFAFLGDATDIRYQVLTNCDLQMVGEEFSRKPYAIAVQQGSPLKDQFNNAILMLLNRRELEKLKEQWWKNDDVQNKCEKPDDQSDGISIQNIGGVFIVIFVGIGMACITLLFEFWYYKYRNNSKVIDVAESTDQQHGGTIVKNVRPAGKLMKQDSLKDSTKGHNYQNLRTRTLMPNLSKFQPRF

>AgamIR8a

MVGNVMKKVMANANGISIKEYYIQYDHEFAPFGEDEQFCAAMFEGVSVLLDTTWIDSRYLASAAEEFGIPYFHIDLSVQTYVKLLESFLLARGGNDVVYILPNYQDADATIYLLITDSYLRAIVFGELDGDTVDRIKDLRPYPSFYAVIAGTMEMNTILQKAIDGGLVRKPEKWNLLFTDLQTDKYTLGDDFPQMNRLVLDSNTCCILLQQRFPCICPDPFDPMDAHLQNVLERIVQWFIKDPIPLVRASNCTPESQQQELLEHAIAAQTKLMNEIAAMAEFWITLESGMLRPNLNLSIISQPANQNTTSAAAQPPPATTIGNVAKGQIVLVKNESLKPSKRFFRVGTTESIPWAYRKRDPNGNILRNSTTGEPIWEGYCIDFLHQLSVVMNFDYDLVSPRNGTFGLRDADGKWDGLVGDLVVGEIDFAIASLKMTAEREEVVDFVAPYFEQTGILIAMRKPVRETSLFKFMTVLRLEVWLSILLAIVATAVMLWLLDKFSPYSAKNNKDAYPYECRKFTLKESFWFALTSFTPQGGGEAPKALSGRTLVAAYWLFVVLMLATFTANLAAFLTVERMQTPVQSLEQLSRQSRIKYTVVKDSDTHDYFRNMKNAEDVLYQMWRNLTLSSGNDQAQYRVWDYPIKEQYINILSAIESANPVATAAEGFRRVNERLDADFAFIHDSAEIRYEISRNCNFTEVGEVFAEQPYGIAVQQGSHLQDELSYFILELQKERYFESLTAKFWNNSARSQCPNTDDSEGITLESLGGVFIATLVGLALAMITLLGEVIYYRRKETSRNFIKVAPFGNADKPMARDGKRIALKQKSIAAVKHLLDLDTPAKALRGATTTVKINTKKEKPIPKEITIGNKFVSAAEKQQKLSYIAIMPRNAIH

>AgamIR60a

MLLVQLVGAMLLALLPSSVNGIVYVRIRNERGPTAARAGIQCLELLSHTYFNMRETTQIENIVIFYLQNLSSPAREILLGYLQLHHTGYDIHEDDDDDEDGEPESQFQLKLMSDSVSSDDLRRAQFMDHKQIDYYVIVIDSFEGLQSALDRISATAAFNPRGKFVVLYNNPNDRDSNARLANRALGHLFVGHHSVNVLFAFAIDATSYHVYTGDPYHGATDCGQMKALKVATVVNGSFASRALSTAMINIPKVPPELESCTFLLCTRVAPPFIDLDCSRGLELQIMDLLRESMKFKVNVSCSTMDRGELEPDGSWSDLLGLMRADECDIIAGGFSPDFDVHDAFGSTTLYLQDYYTWFVPAAGPDARWKLLVYIFEPTTWEAFAGVLFVSALVWRLVAHYLPELAAHRELSMCFLNTWSVFLCISANNRPECNALRLLFMGLTLYALNVTTIYTSILITMLTNPPLAYQMDTIEEILASGVPIGGRLDSEDWFINDFADDRLVSEQYNATSEFQPSLDNLQAVVEGKRSLLMSRLYVRNTKYNGLVHGLSRDVLVTQIEMIMEKGFPLLPKFNRILSNLIDMGIMQKLWNDFLYNVTILDRIRAHRALSEADIIAASPEVVLTLDHLQSAFALYGIGMCLCVVVFLLEVLSKTRWIGKGRVVLEKRWDELLVRLQLKERTAREMTIRPRKMVRFRKVLK

>AgamIR76b

MDRFPVMMMMMVRFVKPCARSYALCRAERWPDKDHGDPQRRRYGMTATVAVALAMMMMIVVATTQLGVDAQGIAPVEWDGNETTEYYQLEMDASAEVKEREMQELRRRLAGTTLRVTTLQDWPLSYTVKINGTYIGAGVAFELLEFLMEKFNFTYELVMPEQNIVGSSNDMAGSVLQLLTNGTADMAVSFLPILADARQHIRYSTGLDEGEWIMIMVRPMESASGSGLLAPFNRDVWILILVSLLAVGPIIYGLLILRHRLTKDKEQIIYTLPHCVWFVYGALMKQGSTLSPTGDSTRILFASWWIFITILTSFYTANLTAFLTLSKFTLPINNAEDVRRKEKQFVTIRGGAIEYAIKNRDEALNALSVLVDKRLVDFTTNVNDSDTLADKVAKQNYVFVRDRPAIDHMIYADYLVRRKINPKNERVHCPYATATTPFLKRNRAFGYPPNTEWNRIFDPELLKMVEGGIVKYKLHDRLPKAEICPQNLGGTERQLKNRDLVMTYFVMVTGFVTSIVVFASELGFRYLNQRKLNEQLAQQQQQQQQQDQQPTSKKLATERISYLGKQFTTGDSPPPPYAEVFSRHQQQQQLGVLGDSERTGKLFDDGSGGLFGAGAGGANRQMINGRDYMVVREKNGLGSRLIPMRAPSAAIFHYTYAN

>AgamNMDAR2

MNETYSLCIIILFLSLSCNVALVVSKSYKVNSGRSGSGISIGNSHNNAANYGTHGSVSTGSANVNTVGFSSSGASGRLVINSGITSVGGGSTSSRLSTNGAPIRNPAEHQLNIGLLVPHTNFGRREYLRSINSAVQGLQKGRGAKLTFLKDHEFQTSNIHFDMMSLTPSPTAILNTLCKEFLHANVTAILYMMNYESYGRSTASAQYFLQLAGYLGIPVISWNADNSGLERRASQSALQLQLAPSIEHQSAAMLSILERYKWHQFSVVTSQIAGHDDFVQAVREQVGAMDHFKFTILNSIIVTRPSDLLELVNSEARVMLLYCTKSEAIEILHAAEELQITGENYVWVVTQSVIENTQTHPQFPIGMLGVHFDTSSGALVNEIATAIRVYAYGVEYYLNDVKNTGRRLDTHQLSCEDQGRGRWDSGEVFFKYLRNVSLESESNKPNIEFTADGDLKSAELKIMNLRPSVNSKGLVWEEIGIWKSWQQQKLDIRDIAWPGNSHTPPQGVPEKFHLKITFLEEAPYINLSPADPVSGKCLMDRGVLCRVAADHEMTDIDMGQAHKNGSFYQCCSGFCIDLLEKFAEELGFTYELVRVEDGKWGTLENGKWNGLIHELVNRKTDMVLTSLMINAEREAVVDFSEPFMETGIAIVVAKRTGIISPTAFLEPFDTASWMLVGIFAIQAATFMIFLFEWLSPSGYDMKTVLQNGQTTPYRFSLFRTYWLVWAVLFQAAVHVDSPRGFTSRFMTNVWAMFAVVFLAIYTANLAAFMITREEFHEFTGLDDSRLSHPFSHKPTIKFGTIPWSHTDSTISKYFKEMHYYMKQYNRSSVADGVTAVLSGQLDAFIYDGTVLDYLVQQDEDCRLLTVGQWYAMTGYGLAFSRNSKYVGMFNKRLLEFRANGDLERLRRFWMTGTCRPGKQEHKSSDPLALEQFLSAFLLLMAGILLAALLLLLEHLYFKYFRKRLAKKDRGGCCALISLSMGKSLTFRGAVYEATEILRRHRCNDPICDTHLWKVKHELDMSRLRNQHLEKTMQIHGIKPPQPKLGSTNKIIRIAGTTGARDLIDDGYQQPNLFGNLSIGGSSQDLYRWSYKTEIAEMETVL

>AgamGLURIIe

MQCLGRVMGFNFLSVILLLVHVLCGYVDGKREIPVGAIFHQVPDHSYESEIAFRYAIERVNMHEKHFELVPIVRYVSPDDSYKTERKVCELAAEGVTAVFGPSSILTSGIVGSICKTLEIPHIITHWDPEPFGGLDPDLQAMTINLYPEADVLSRALADLIVDYSWKSFTIIYDSDEGLMRLKDILQIHGPSDSPITVRQIDDNPDYRPLLKDIQTSGESHIILEIRPDRILELLRQAKEVKMLEEYQSYIITSLDAHTIDFEELRYSRSNITALRLMDTKSFDIKNAVHDWEQGEARMKRQFRVSPEHVHTESALYNDAVKIYATAIRELDATEEITPSKLSCGSKNLRQWPFGLRIINYMKVKTEHGITGPIIFDDFGRRAHFHLDIIELSKDEGFKKIATWDPTHGVNYTRSQGEVYSQIVESLQNKTFIVASRIGAPFLMFKEKKDGEFLEGNNRFEGYSLELIDGISKILGFQYRMELVPDGKYGSYNKVTKKWDGLVKHLLDRKADLAVCDLTITYERRTAVDFTMPFMTLGISILYAKPVQQPKDLFSFLSPLSLDVWIYTATAYLGVSVLLFVLSRMAPADWENPHPCKQDNDEVENIWDMLNALWLTMGSIMGQGCDILPKAISTRLVAGMWWFFALIMLSSYTANLAAFLTMERMDATIESAEDLAKQSKIKYGVLMGGSTMAFFQTSNFSTYQRMWASMESVRPSVFTKSNDEGRDRVIKGKRMYAFLMESTSLEYITERYCDLTQIGGLLDSKGYGIAMPVNSPYRTAISGAVLKMQEEGKLHQLKTRWWKEMRGGGQCTEVPNSAGENELGIGNVGGVFVVLALGCFCAFIIGILEFLWNVRKVAVEEKITPWDALKAEMKFALNISVTSKPVHNTLSESTASDKSSLRSKSESRRGSELTGRGNNVRTATSLNNLNKIGFVFDKH

>AgamIR41c

MDYKMVNFLPLDNFNTSLQFLLTHLLRLYYSQHYTVCFIRSFHDNIPFTTGEPPLPLVHIVLEDEVLTAAAPPNGTAWATKKEEFCQKLLQAVDTNCGGFVVTESTLFPFLEHFYEVHKRAKLRPSPKHLIAMTTSATFDRSRLLQYNQTLEALVNLLLIVRSSSDKGNHHRKDAFELHTTQLLPNYPLGVRIELVQIGKIVLKQDANGVVEFEPKHDTEIDFFPDKVRDMKGRRVRVSTLEYVPCSVYKKPIGQGNAKCADDAAHEFWLDGFELILTMEFCHRHNCTVELSMINQTDWGEVFENGTTTAILHDLVEQRADVGLGALLAWHAWFQLTTITQMIGISMISALVPKPRLLPFWQTPFLSFPPSLWLVVGITFTAGTLTVFMVSVARLRILPPNVDDRQRQSRSQHLLDALFFMFSLYVEQSARLRKDLLAAAILLAALLFGGFMIGNSYAGALASTLTLPRFEASIDTVDDFLARGMRWTGGSAVWIYTLYLATTPAMIRMRESFVVIPDSAERERLPFTDPRMGYVIEGMMHGSYGLGTPLALNASLLLQPLKQPIYLDHTNGFCSKVWPLRDAYNAFILEVHQCGFADLLIYYPLQSLIRHYGLTVQRNIVTARSQDNGHEPVALSIDHFLGVFFIYFFGISLACIVFVVELVLHRWNARF

>AgamIR41t.2

MEATVAQLGLNSGVPIPTMSVLMRFLILKYYSQYYSFCTVGNDFLQDVPLSRIVLSLYDQLNTSMLHAIDNGCQAFVVDEQGAERFLDLYMPVHDLARQRSLDKSLILLIAKRNQTHLFDRIRGHEAFRELANVLIVVYDEQERPTDLYNTVVDMSDVLERSVNVAMELVAVKWTSWKNHSFFPDKARDLNGYVLRTSMCNYLPFTGYERLDQETGNAFDSATGKRSIWLDGTELQLLVSYCERRNCNIMVFPEDEDEWGDVYPNGTGIGLMGSVAERRTDIAFGAFYLWFKPYNFSSYTATISRSGVTVLVPKPKLLPHWRTPFLSFSWPLWIAVMVTFLVGIIATWLAGTVRLRLLLLSAPTDQPWTGRSDLISEQLTLSDAVLMMVGFFVAQSSPIRTDLWSCVCLFASLLLAGFMVSNLYSGGLASVMTVPKYEKSIDTVVDFAATGMKWFGPTPYCLEEIWNASEPHLQQIQKTYIAAGPEEMNRYAHMGGSGFIVEQAQHGNFAPSNFLDRQVSTTLQPLKDYVYTQNCAALITNTNPLRSNLNEYILRVQQSGLLYHLGIRTAIRYLPTDVMRNIERSRMHQIDDGAVRLELGHFLGAFFILGYGLLCASLVFVGELWGTVMMRRWNETFVSKATVVTAY

>AgamIR75g

MTKEAVESTRKQTRIILHKVLIHQLHSAMKNHPASMSFLNVASFSGSKFEASLQSLHVGVGLMADLNCDGLAGVLKALSVRGFFNGARFRWLLGGLGTLQQSRMFLEKLNITASSMVLLTLPMRTSNNDTNFAILDVCGHRVGTRWELSFLQIGAWSTSAGLTMWDKRSVYERRQNLLGMQLTGIAKATAIGASTVTGELQSGRAYGLRLWNVLSTMHNLSITENPVNEGNQQPFDLIINPVEIKQQDISKLHYTAAVHDTQYVSLTILLFLHPNVDWTRNLFLRPFTVLSWLGITALFLAFLLLMCCILHLDRLEGQDRENGILVLVLGILCQQGFIETFHSYASRITVFAMILFSMLVYQFYLTYIVSFLLVVPPKTIHTLHQLVENGFAVALENVPKNVEYLNATDDEHLLALVQKQLTQADTLYYDMAEGLELILRGRVAFLCDAHHAYQMMQTHFTDEQRCALQEVVLISKKSTHLALAKDNPLRELFRVTVHRIAGNGVMQYERSRCYADKPRCAENEVKMPEVNLDQVSSVMVLLLGAIVGSIAVLLLELTHSRVWPRRHRVVPSRGINRKL

>AgamIR7h.1

MCNEHLRFVMLLLLLLLVTLQQHTAGSAIADERPNHIDRWVSCIGEIVLANADTFRHELNIFTLGGGETEEESLHFEHDLLDRLLARLVYRHADKKPFTFNLAPYDRTVSYNQRNLVLILLHDLRQLEQHLLPQLAAKNVEQRGYFLLLLVPSNATTTTTAEKHVAATFRLLWSVRLHNVVLALERRRPTTRIDLLAYDPYGPGCCGCSRPRLLAHCNAPPPPLYDRFERNLHGCQLRIASFERAPFLEFVHRPGRNGTATATPKLTGIEGNLVELLAAHLNFRVAIVQPADGRTWGRIYPNGTANGALGLLLNGTVHMTVGGYFPYPALLAATTQTHHYYTAELVLAVPEELATLSPLEQLLKPFQPAIWTVVAVELAIGALGALHRRHRLLHFWRTVIGESLPGRTVPRRTVARLALLLWVVHAALLRESYKGSLVGFLTEPNPLADIHSLEALVQAGYRFVMTETVYHTVFDRSSTNRRPLAPHQVLLIQSADRQALLERTIRSRDRLAFTSTREEIVKFNSRNRTDINYRTSDETLLTFHFAMYFKRSSPIAAACDWYIRRVVATGFVARWHHQHLDLRFERPTLAGSGQAEVLQLHHLHGSYLLLAAGLLLATAVFTLELLWGRWCERRRRPNYPGPYPYLHELSRQYPPVGRRKRTRRIA

>AgamIR7y

MNRWTILLLAAGLRECRPEDSNGGMKRLVVRETVGNHFSEVIVNALTRYYVQNHSSTQVMRLSTVSDGTYDLQSDIMDEVMQRTSRSIAYEFRSALGPSRRPRIFNIAFIDGYGAFEQLFRSLDPAENDFAGYYLIVLTAYGRRIAPDTLARIFALLWTMNVINVNIVSADLEDQSRWQSVLMHTYYPYRAGGNCERIVPHLLHRFRKGQRLDRSVELFPSKLSNLHGCPVTVGTFHLPPYMLLTPAGRYGGLEGDLLRALSRKLNFTVRLVVPDDGELWGRTPSPSVVVADANRTRGGASSASGCVRLVLTERVNLTLGRFAIRGDRNLVMKSSRSYYTVRMVLAVPAGREYTPFEKLFRPFSRSVWVLVTLYLLAGMVVIGTVQLLHRPAVRDFVYGRGVSTPVLNLLSVLFGGAVVQLPMRNFARTLLFLWMYYCLVVRTCYQGSLYEYLQERKNFSPLQTVDALVRNDYRFYSLHGTSLYLEQMPQILSRITWLPDDDASVDRLLTELATRQALTSALFIDLERVAYHNRFYVRGGLVYIANVVLVRLPIGIYYTKKSCLANRFDHELDRLRTSGYVSYRLGHYLNYDAFKGPADYQPLPTPLTTDQLVGCYETLFGLLLVATGLLLLELASRWFVSLRQLLTFLQAE

>AgamIR140.1

MVMSQAIVVAVFTYLALLAPDGNAFDTFPLLTPLESNISFILKAFVELQSQWCPPWRYKKFYILNYNSDQLSEDILTGILQLNGPPWIISTEASGLTYWTYENNRCAVIMVSGYERMRPARPLYYGSRYFIFHPNLVEGNVFMDQINSGMSCILDKAYFIVYNRTTIEIQHKNFFTNRTIQLDPANIQVTDDLKDLYGRKLHIALPEGNEEVATFERYLAETICQQRNASYEISSLYDASVDYGVVNMGVPFPTTDKTVALGSTFVSVMVPRSKPKPIIAVLIDPFDYYTWITLFVLIFLLALVLSLFGKVLSRWNIAENALEMIMCILGGPTRQYGGWFENQIITNYCLLAIVIVYSYQSLIISYLTYTRYSPEINTMDEIRNNCIFPSGSSWAAYFDFHTDGEYDDERDNMCFLFASRDDKQITTLLMENMRDINPAAGQAYKRNLRVADTVLFKYYLIYYFLNKSIIRELFPFYIGAFRESGLFDQHYRNKSIHTAIHSREMFGVRSFNVADLSIIWYLYAGGVMVSILWFGVEIAFFHCLRCGRMVRKRCQTYFLRRKCEIRNTCSMEKQVQTLNERSKQQSLPVFKQQHWKCSVL

>AgamIR7u

MKLFAALLALAMVAQHCYCSAGKLIPLVDAVSATEHLQAPLKQHFKYPTFPVNFRAESSENGTTTTTRELFEMNELMRENSGWLIGTVSGRLPIVTNPYASFYNVFFADGYDAMGKILQTLNYTDYDPTGRCLLVINAAYETDHMVQLVAILWQLRIVNVALIVQEAATDTDSYRAYSYDPYREGKCELLEPLLLDQFVAGRWQSLHRWYRNKMENFHGCSLSVGTFAAKPYSMVRREGNATIRYGMEVSIVENVARWFNFTIDYRSPAGTVKWGIIRAANSTGMMGMIQRNEVAFGFGCMGYNEYRNRYLTVGLPSFITQLSMAAPPARPFTWLEKLFAPFTLEAWLCIALCYAGYLLLTVLVFDSRLVTTVEHFRNPAYNVWVMLMGGPSRPVRQTSIRLFLAGFVLNALVIRTMYHSAMFERLQATTTLGSDLNTFQQINAAHMLYYMYITTSFYYKDNPLVHDRIRILWDETKDWDEVMYNISHYRLNGVFVIPLDAIEYYVKNVGQRGLVYVSSHTSINYNPGLVYPKASPLTEPFSAIIGRYQAAGLVPIWREQFRDTRYWNNAKQHPEPISLQWSHLSGGFYLWACLLALSSLVLVGERIMSRRK

>AgamIR7i

MKVLLMVSLCCIATSLCAMVPVSDHHRNKYHHLTVPIAHHFKDTNIPVVFWLDSPVYVTNQSTQLDALHAIVLAHSDWMVAVFRSSLRCMRCRTRLQNVFIAATVRSLQTFLASLEYDCFYPSGRYIFIVTEQLAREATDVRDVFEIVWKNRIVHVVLIVSRSNDTARFRAYGYEPYAYGKCGKVRVKLIDRYTDAGWRRLAGGWFNCGLPNFNQCPLKVATFESKPFVMVRTVGNVTRYSGLEVKIFNHIAAKLNVSIVYTPPPNNTRWGVLLPHNSTGQMGMLQRNEADVGFGSVGRSIERDLYLRSSVPSIVSQLSMTIPPRLPYTALEKLFLPLRPSAWLLVAAGYTTILCLYVVLFRGKHRPRRERIPGLYYTFWTILMGGPGREVHRHSTRLYVISLVLNALIVRNLYQSALFQRLKSNDLMAANLHTYQDINKAGLSYYMFRATVRFYADNPEVNGSIRTIANENIDWDEVMYNISQHRLKGVIPLSLESIAYYVKHRGQQQKGAMVYVSEHTAISYYVAFHFPRRTALQQPFDRLLHRLQAGGFILHWRAEYRNNPNGATNYEQQDGVVPTPLQLQQVAGGFYLWALGLLAATVAFVGEFAVSKVNRASKHI

>AgamIR7s

MWWRRRVQLLIAPLWFHCCCWAQPPLPTQPLVNLADIVGVVLHTHFRTPFATTLVTVRSATQRGAWLQQDLLERLLVRHGQGQLVVQLEGVPSPVQLLHRHPPWSRTLLLAESYDALRTVFGQLTPDRFDFTGRYLIALSEAPATLATVDRIFHELWLRQIVNVVVALRPLDDDHSSAAAAAGPVQLWTYFPFSPGLCRIPKPHLLFTWPNDTLLYGVDFYPRKSDQFHGCPLRVGSFETRPFTILAGGDGANPPAVGGFEGDLLHSLSARLNFREDVRVPPRAQQWGEAAFENSTGLMRMLYTEEVDFGLSCLGVSVERSAMLKAGKVHFTTDLVVVVPPGKPYTAFEKLFQPFQPPVWLAVGFCTGVGLGVLATLRLLPLDGRQRATVRRYVAGDARLQAPALNLVRVLLSSPLPFTPTGTFPRTLLAQWMLVSLLLSLLYQGSLFQYLQRASTHPPMRTLAEIDRAGALYHIASSARRFFLPYPQRLSRVRYFPPVPDSIAAWLRWMGSHPGEPHVAMCTRDHVAYHNAQHGRTDGRQLLIARESMALYAITILYPKRSMLTASFDQHIERIGSSGLLKYWSARYGDYHFGESRASSSSTTDQRKTATAGPRPVSVEQLAGALQLLLGMLLAATLLFLVELGWARHTRRWRHNC

>AgamIR7w

MLLFCLMLWCTFADGCTVQRREEEMPPEFLQSESTNTRFLAPILRAHYRALALEVHFRSWNGNDTQRQLTPWQGELIDDALCRNADWMIVSFADLLAPAVQRRSAHYSVLLMLDYDALCSWLQHGLDSGAYHFDGLYTIVIEQLADRGQLHAVMRELWNREIINVVVVVVLASGEADNRGELVAYSYDPYGEGQCGNAAPYEIGRYANNNGTWDRLAGWFPNRLTNLHGCSLTVGTVEVSPFSMTRTVDNRTVQYGLEVHIVDTLAARLNFTFRYVRPTDGVKWGILYAANSTGLVGLLQRRGADFGFGSLGFSLNRHTYLRMGVPNHMTQMIMGIPPKRPYTSFEKLFQPFAADAWLCIALGYAAFALVALALVTVNRRLAREPALQHPLYQLWVLLMGGAVGWLRLDSTRLFLIGFVLNALVIRTLFQAGLFQRLQSSASLASDLNTLEAINRAGLFYNMFRASLQFYRDNPSIPASRIKLVPNDQRDWDDLFYELSQDRLGGVMVSPLDCIAYYVKRRGKNGVVYVGKDTGFMYNLGFHYPKSTVLQRPFDGWILRMHAAGLVHHWSEEYRDNRYWTNAKEDPEPASLRWNQISGGFYLCSALMLLATVVFLGEIVYFRLRTRRLLQRCCGRKTRTRRKKSV

>AgamIR101

MKQACSCVLLVLLWQRVAAVLDPLRHNDCHSRAVSMETRILDDYLADQHTVTVIESCTEIPIMSPYCMHNPVPLNAFTLHRFQAFVHQSLEHTTQELEYRTNECFVGSGHMDDLLTELLPYLSEYNPRAKVMLITQEMADQELAELFHAAWYRYRLLQLIVLNHRANDTIESCLFNPFRKALSPELRYLPTGKSDLHCRLLTDGQQLDAYNRELNLFIDDRVYNLNGYPLNIAMHVSNGSTSAYDCILGTVSFTDIDQEILSIMQKQMNFSLILHKNELELSIGYIHQNGTPVGTLGLIEQNAIDLAANSRIIHNYDTRNLLYLHYISTEKLVFITPRNYFRNRDKTQVFINPFSVAYMLTNVLLSFGVPMIIFLLEYAACRLDVPREPSSHTFGTKVLNLVGIIYNVSVKLPRADRKRWIIVGLLVYNIVSYPIWQGVTIRYLHPSNQQVNNINSLEELIETELELKVSHYHEHIVRHEGPHFQSPVYSALSGRLSTRNTSSLRDSIEQIIMHGNSALLIAEEYVPLVLAGNYQWIPGKPDGIWPIQKPIYEFYKSMAVPKTSPFAGTFNAIVLHCVEAGMNDRFKHQLETAVQLMQIRRVREHPSVPDYIVFNMEHLLPLFIFYFAMLLLSGGIFLIELAVHAFQASRARSQQRAPLRVPVEEYVPFEFVH

>AgamIR140.2

MKAFVELQSQWCPPWKYKKFYFLNYNSHPLAEDIFTAILKLNEPPRLIFNEASAFHYWTYEHNRCAPVLKPVYLGSRYLIFHPNLEEGNVFLEQLHSGISCILDRAYYLVYDYTTIQIQHKNFFSNRTIQLDPANIRVPDDLKDLHGRKLHISLPEGNEDVVTFDLYLAETICQQRNATYELQSINSTLLDYGVVHIGIPLLGTDKTVALGSTFVSVMVPHSKPKPIIAVLIDPFDYYTWITLFVLIFLLALVLSLFGKVLSQWNIIENALEMIMCILGGPTRQYGGWFENQIITNYCLLAIVIVSSYQSLIISYLTYTRYSPEINTEDEIRSSCIFPFESNWARHYDFELDGEYDDERDNMCFLLLSRDDPHITTLLMENTRDIDPAAEQAYKRNLRVADTILFKYYLIYYFFNKSIIRELFPFYIGAFRESGLFDQHYRNKSIHTAIHSREMFGVRSFNVADLSIIWYLYAGGVMVSILWFGVEIAFFHCLRYGRMVRKRWITFFQKYHGQAKFMKKKLKSFNMT

>AgamIR141

MMRKALAFIVPLLLPMLIFTPHTQSGPSRPGGDRDGRHIHRLSIASHETRVEFWTQLIAYMDCSHVVLLDSFEFFANRPTFARQLFARLNDAGIRVSVQRRLREVRRLPPVTHRLAIIVPIVMNPSSPFNAELDTFIGQIAQESNFVEFYRWLFVFRASHKKSVWRALQQLPIRADSHVYAVIVVPTAKTVPFDDRIHLERKMMPLERFVRRMQMLPPHHPEAATRTSAILTLPGHTTAAGWVAVWQLYRLHTRMKTIDVAIELLETSTEGRMILYRGVAPEQRNDFFGQATITTMVHRSAPHHINSTYVAQSIPMFRNASTRIRTYRIGSGRHKVDLHFQLITTNRFNQRLQGWYSYSLPLFHEHLTMYIHYTHLSYHEQSSQILSIVLLPFALLGLLLCLSLLLALRTGNSYRSDRNQSRPVSFIDTLIWMVGVLAQQGSIIRPNSSSSMVIILVALYMSAIIYCSYLTKIASLLSVDASVNLDLQTVLSAGQYQIGFVGNNTADETVIQKRYDPVMNEIMRRMVQNSSLYSLSHEHALHRVLTTKYVLIGNVGSVRKAMQTLDEQQNCNITEIELTGIEQMIALQMPSFYAYRKIIHYE

>AgamIR7x

MAPLPVWWTSRLMCCWLLWSTTAARAQIPPQPDTDQQLLPHIVTHLLQRYLRHPFQPVQFFLAANTAPHLLAQRDLLSQVLRHTSGQAAVTFGSSTNNPVGLRAAVQQQRSRAILFGEDFVAFERLVANFSTTLNDYSGRYLCVLTGASGSVHQLETKHLPRLFDALWTRHIVHVNVLIAAANGTVRAYTYQPYTPERCGKPAVKLAAVFAPGSSVDAQPHLYPRLTTFWNCTLQVGSFEAKPYTLLRPKVDGYTELGGFEGDLLHLLASRLQFRVNVTESPHQVQWGVIGAPGNSTGTMQLVQDELVDLVIACMALDVTRGLYLKAGWAHYTSRILFAVPQGRPYTAFEKLFRPFGTAIWAALAGTLLGVAGVVGGLSCGRRARSWRRFVYGPAVRMPLLGALYLLWGGSVVAVPGRNFARSLLALWLGFTFVVRTLYQGSLYLYLQRSATFPPLATIEQVHRSTLHYHMVNIAMRFFVDRPEIKPRVRFIPPGLDTLGEQVAGMAARYTDRVVVCPQDMVAYNNRASRGRQPAAAPIQVTRESITLFPLTIYYPKKSCLTQPFDRLVRHVVESGLVSFWVRSYGDYDFEANRREPAGGGEPRKLTLAHLVGAYQLLAAAHLLAFVIFLLELVSLRLAALRRVLEFCMD
