# Supplementary material for: Transcriptomics reveal the molecular underpinnings of chemosensory proteins in Chlorops oryzae
Source: BMC Genomics. 2018 Dec 7;19:890. doi: 10.1186/s12864-018-5315-4 (PMC6286535; doi:10.1186/s12864-018-5315-4)
Supplement: Supplementary file 7 — Protein sequences of OBPs used to construct phylogenetic tree. (DOCX 28 kb) [file 12864_2018_5315_MOESM7_ESM.docx]

>Co-Cluster-17022.1

MLRYNSPIVVVFFSFVWNVIIFEAAADPVTPSTSTSTYDTKFDNIDIDEVLHQERLLNHYVKCLEGKGPCTPDGKMLKETLPDAIETDCSKCSEKQKYGSDKVTRFLIDNRPEDWDRLEKIYDVEGKYRKKYLAEKAEMAAAAEKERSN

>Co-Cluster-13523.0

MNSAVQRFQEFLNNCKAQCVEKMGLAPNEVDKSLLFEENPTPKEKCLMACVLERTKIMNHENKVSVHTIGMMAGMMANENELVKAFAMAAAENCNRHINTSNRCEAAAQINRCVANGLKAHRIKLDY

>Co-Cluster-17517.0

MKPFTLWLFCCVIVVAVEKCYASVSPNELDVSNGVCQRANNVTTEELSALVSAIKQLDFDESSEDSNEDMLSEAVNELQENFDAGMKCFALCMFEYLEILDEKDKIDVERVREKEDVSHQQVEEVKVCNELFDDEENACDYGFKMFVCFLIMEIKHENSPAQK

>Co-Cluster-17667.0

MKSFLIVFNLLSVLWLILMSHSTIGNGFEMPENLKNQARKLHERCQKQTGVPEGLIRQSHDGNIPDNSVLKCYIHCLLDMIGLIDSNDIVHLEYLMEVVPSEGHHIINNLLEKCGTKKGTDGCDTAYRTVKCYFEVDTPSLMAAFGFIFD

>Co-Cluster-3781.101742

MKALLLMSFISCSVYTAWCADDLNTVSSACAAEVKVTEKEVADFYTMDGKIKTPTPNLQCHVKCVMEKFDQLKNGVFNAEKAAKGFKEIPEFSENGDRFDIKFFESCKNEKGANDCATAYKIFECLRNKIPKKA

>Co-Cluster-3781.110134

MKECGFSEKRMLPKGSRQRQAVFLALIIAVSIALGGAQQPRRDDQYPPPAILKMSKPFHDTCVEKTGVTEAAIKEFSDGEIHDDENLKCYMNCLFHEIDVVDDNGDVHLEKLFNTVPGSVRDLLMNMAKDCIHPEGDTLCHKAWWFHQCWKKADPVHYFLP

>Co-Cluster-3781.117642

MNIIPQYLDKPPNAYIKVVYDGNMIAENDAEIAPLKTKEPPTVEWHASVETYYTLVMVSPDAPGRGNPYLKEWLHWLLVNIPGNDIQQGEVICPHISSLAPKGGGLLRYAFLLFQQPDYIVFDEPKLNDTSTEGHDHFDTMKFANKYGLGTPVAVTTFQAQFDDFVPIMHAQLHIKPV

>Co-Cluster-3781.140399

MKFFTLCFAVLVAGAFADELTLQQKKRLHEVVAQCITETGTSAKVVKEMKNGDWKNVGANEKCFSDCSLTAIDFIRNGVANEPVIKATLEPHFGWQKVNAAIQACKGVTGVDKCEVGYNFYKCYYDNGLYVPL

>Co-Cluster-3781.152546

MKFFALCLVVLVAGAHAQGLTDEQKAKLRGIVVECVAETGASDAAVQGLRNGVWDAVDNEGKCFATCGLEKLEFISGGAVNAPTVETKMGPIFGAAAVKDALDACSPTSGADRCEKGFNIVQCFYGKGLTFPL

>Co-Cluster-3781.159183

MESLNLKMQKFLSVLLFCILCAAIVKCAINKEGIMKTLHEHANECMTKEGASKSDIDDLMQKKPAAGKPGKCLRACMMSKVKLMDANGKFDKSVAMEHGKMFADGDAEKMKIGTEIIDKCAATSVPADRCDAAELYGKCFKEETLARGIDIGV

>Co-Cluster-3781.162639

MKLLLDSIIVLILISKSCSDRKADSLAAWASCQTVHNVPSGLAKEVIERRTKPEDAPENIKCLFSCWFEKLGVLVDGIYDSKIALKIVQERPDWRGMFVEFERSIRDCKKERSTDICDMGYRLILCLRNVKN

>Co-Cluster-3781.17418

MKAISLIFLLFALSYCNGQFAAMSSTCAAELKLSEKDTKDFFKMINTMDVVPPQMQCFVKCVMEKAGEFKNGVLDMEKTMKDWKEIPELAEFRVTDPKVFTKCKEEKGANECETAFKIFKCLRQNKAMK

>Co-Cluster-3781.178841

MTANDRPKYAQLSLLLTVLCSGVTGDPSFGNGYKYYATKKSKCLNPPRTARRVEIFIGECQDEVKNKLVNEAYQILKEEVAKEHPKLVDPDDYTFDLIETTEEKPTNTQNWADQLKIPPTASSYWNVKDVANYDEDSYTDYIDITTTETPEKARRKKRVARLLHRIRRRIGDIGHGAGIYHPTLVPYEDKRIAGCLLHCVYAKNNAIDRHGWPTLDGLVDFYSEGVNEHGFFMAALRSVNLCLRAVTNKYHIDRRKLPEQGESCDLAFDVFDCISDQLTGYCLDQYD

>Co-Cluster-3781.178870

MTANDRPKYAQLSLLLTVLCSGVTGDPSFGNGYKYYATKKSKCLNPPRTARRVEIFIGECQDEVKNKLVNEAYQILKEEVAKEHPKLVDPDDYTFDLIETTEEKPTNTQNWADQLKIPPTASSYWNVKDVANYDEDSYTDYIDITTTETPEKARRKKRVARLLHRIRRRIGDIGHGAGIYHPTLVPYEDKRIAGVNIILKFSAHHTSTCPCCQFKRASSKLRGISSP

>Co-Cluster-3781.18626

MDGNGIVPEIIDEAPKELAKITYPSGAVVDLGKELTPTKVKDQPEVKWNADSNALYTLLMTDPDVPSRENPTLGEVCHWLVINIPGDRVS

>Co-Cluster-3781.30341

MFNARENLFNFGIVIFQSYGQQDLDLMSKACQEETHVTDEELTKHYSNRLRESDAKENIKCHIKCMMEKGGYFKNGILVETAVFNTLKVKPKYTEFQAQVEQAVGSCKNVKGVNDCDTAYKITKCFNEFKSHTKQ

>Co-Cluster-3781.30619

MSIILIKILLSFAFASIFCHSVTVAVEIDCQRPPQLVDPALCCQDGGRDAVTENCAKRFGLTGKPTDPPPTVQTATCLAECILMQSGYLEPQEKVNFNSIQSDLTRKFTNDTIYVQSMVEAFKKCEPTAKTKMQAFRQLPLSKLALQRGCSPFSGMLLGCAYMEYFKNCPAHRWTQNEQCALAKQFVTQCALGA

>Co-Cluster-3781.35578

MKGIFCYFLFAALCATALGSKGLAENMKRFSDECVKEHGIEASDIEIAQSADFKPEDIKNNVKCALHCILMKGDYFDDKGKIKPDSVRATLDENQKKIFDQAVEDCNDIQGKDVCDTTFLKFGCMMKHKP

>Co-Cluster-3781.36358

MTEEDFQKHKVVPDMIKKPPPSILKITYKDVEVCYGNELTPTQVKAQPNIEWEPEDKAFYTIVMSDPDAPSRKDPKFREYLHWAVGNIPGKNIDQGEVICAYVG

>Co-Cluster-3781.38930

MTDPDAPSRENPIQREFNHWLVGNIPGHDLSKGENLAEYIGSGPAEDTGLHRYVFLLFKQSKKFVFDEEHLPNNSIRGRPKFSTQKFAKKYKLGVPLAGNFFQAQFDSSVPELHKIIMN

>Co-Cluster-3781.66842

MKNLNFPDEPNVREYLLCTAIKMDIFCTHEGYHADRIAKQIKMGMDEAEVIEIAQGCIDKNEQGSPNDVWAYRGHKSLMGSK

>Co-Cluster-3781.66844

MKVIAAICILFSVASAEYLVKTQEDLINFREQCVKNLNVDAEHVQQIQKWQYPNDAITHCYLKCVFEKFGLFTEANGFLVEDIHHQLLGQLPSADHAKDTLHGQIESCVNNIDKSLGICEQAYVGITCFFKNNLQLVQKSVAPAVTA

>Co-Cluster-3781.78579

MWAAGKLMRDVCLPKFPKVTNEIADKIKAGNVPDDKDAKCYINCVMEMMQSMKKGKFLYESSMKQIDIIMPDSYKDDYRNGVQKCKDSPIGIKNHCDAATAVFQCMKREITKFVFP

>Co-Cluster-3781.90561

MKHMIAILFALLALANANEYRFRNQEDLVAARKACVESKKVPEAHIEKYKKFEFPDDETTRCYIQCILEKFELFNPKTGFNKGNLLHQFTHAKPAGNSE

>Co-Cluster-8053.0

MQQFRQSLDMMRNGCAPKYNVPVEVLDKLRNGEFTEGIPDLKCYTKCVATLAGTITKKGDLSIQKAQAQIPIILPPELQQGARDALEACKDIQKKLQGVMRSSLLFDQMCTRLQSRRFRVPLASERDGSISRILKHKVNGHQLKFEFHKFVNNNYFIYTYTVQ

>Co-Cluster-3781.76321

MKFQLIIFLFACVALALAKFQIRTREDALKAHEECREDNYVPDDIYEKFLNYEFPEHKRTNCYVKCLVEKLGLFAERKGFNEKNIIAQFTHDNTKNLDSVQHGLEKCIDHNEWESDVCTWANRVFSCWIKINRHIVRKQFGEQ

>DmelOBP18a

MKVVCSIAVLWICLITMWQSAGRVNAEGCLKHHNLTSAQVQAVAPSTPVADVPVAVKCYSRCLIQDYFGDDGKIDLQKVGKRGSQEDHVILSQCKQQFDGVTNLDTCDYPYLILQCYFKGKQSGTIAS

>DmelOBP8a

MMRRSQIGLLSRLLLLLLVVELTPPAIPVPMRSSPQSLALLRARDQCGRELTAAQRLQLDRMQFEDAAHVRHYLHCFWSRLQLWLDETGFQAQRIVQSFGGERRLNVEQALPAINGCNAKTSSRGSGAQTVVDWCFRAFVCVLATPVGEWYKRHMSDVINGNA

>DmelOBP19a

MKFHLLLVCVAISLGPIPQSEAGVTEEQMWSAGKLMRDVCLPKYPKVSVEVADNIRNGDIPNSKDTNCYINCILEMMQAIKKGKFQLESTLKQMDIMLPDSYKDEYRKGINLCKDSTVGLKNAPNCDPAHALLSCLKNNIKVFVFP

>DmelOBP19b

MMQCSRMTTTLKMTNLLLAVACAAVLMGSATADEEEGSMTVDEVVELIEPFGDACTPKPSRENIVEMVLNKEDAKHETKCFRHCMLEQFELMPEDQLQYNEDKTVDMINMMFPDREDDGRRIVKTCNEELKAEQDKCEAAHGIAMCMLREMRSSGFKIPEIKE

>DmelOBP19c-C

MKPSTPVAAIPLMTIVVAVLLQTHCVRGQTQAFDLAKLLPKTGTEPIWAVIDRNLPQVQELVTAARMECIQKLQLPRDQRPLGKVTNPSEKEKCLVECVLKKIKLMDADNKLNVGQVEKLTSLVTQDNKMAIAVSSSMAQACSRGISSKNPCEVAHLFNQCISRQLERNNVKLVW

>DmelOBP19c-A

MKPSTPVAAIPLMTIVVAVLLQTHCVRGQTQAFDLAKLLPKTGTEPIWAVIDRNLPQVQELVTAARMECIQKLQLPRDQRPLGKVTNPSEKEKCLVECVLKKIKLMDADNKLNVGQVEKLTSLVTQDNKMAIAVSSSMAQACSRGISSKNPCEVAHLFNQCISRQLERNNVKLVW

>DmelOBP19d-A

MSHLVHLTVLLLVGILCLGATSAKPHEEINRDHAAELANECKAETGATDEDVEQLMSHDLPERHEAKCLRACVMKKLQIMDESGKLNKEHAIELVKVMSKHDAEKEDAPAEVVAKCEAIETPEDHCDAAFAYEECIYEQMKEHGLELEEH

>DmelOBP19d-B

MSHLVHLTVLLLVGILCLGATSAKPHEEINRDHAAELANECKAETGATDEDVEQLMSHDLPERHEAKCLRACVMKKLQIMDESGKLNKEHAIELVKVMSKHDAEKEDAPAEVVAKCEAIETPEDHCDAAFAYEECIYEQMKEHGLELEEH

>DmelOBP22a-C

MRVLLAFVLLLGLSVLATKEPEEVKIVSECAKENNVHRKKALDLLMSYRLKKKTHNVMCFINCIFERTNILQKVKEKVVKENHNCDSIKDADKCAESFQKFQCLVKIEMKVRGIDRG

>DmelOBP22a-B

MRVLLAFVLLLGLSVLATKEPEEVKIVSECAKENNVHRKKALDLLMSYRLKKKTHNVMCFINCIFERTNILQKVKEKVVKENHNCDSIKDADKCAESFQKFQCLVKIEMKVRGIDRG

>DmelOBP28a

MQSTPIILVAIVLLGAALVRAFDEKEALAKLMESAESCMPEVGATDADLQEMVKKQPASTYAGKCLRACVMKNIGILDANGKLDTEAGHEKAKQYTGNDPAKLKIALEIGDTCAAITVPDDHCEAAEAYGTCFRGEAKKHGLL

>DmelOBP44a-A

MKNAVAILLCALLGLASASDYKLRTAEDLQSARKECAASSKVTEALIAKYKTFDYPDDDITRNYIQCIFVKFDLFDEAKGFKVENLVAQLGQGKEDKAALKADIEKCADKNEQKSPANEWAFRGFKCFLGKNLPLVQAAVQKN

>DmelOBP44a-B

MKNAVAILLCALLGLASASDYKLRTAEDLQSARKECAASSKVTEALIAKYKTFDYPDDDITRNYIQCIFVKFDLFDEAKGFKVENLVAQLGQGKEDKAALKADIEKCADKNEQKSPANEWAFRGFKCFLGKNLPLVQAAVQKN

>DmelOBP46a

MCSQLFAFLLLLLTAFVTGRSTPPALDEDCELNSVDTMHDFCCDLHDESPQFSDCQMEWHEKIPYETDEEEQTYMFCTAECSFNSTNFLGRDRRSLNLNEVKEHLESDLVNDADIKLLYDTYVKCDKHALSLMPHKGVKQLSKRLSRLGCHPYPGLVLECVANEMILHCPTKRFRQTAQCEETRNHLKQCMQYLKYKS

>DmelOBP47a-A

MNRVLVLLLVLKMFALSESRFAKININLGLTVADESPKTITEEMIRLCGDQTDISLRELNKLQREDFSDPSESVQCFTHCLYEQMGLMHDGVFVERDLFGLLSDVSNTDYWPERQCHAIRGNNKCETAYRIHQCQQQLKQQQQNLLATKEVEVTTTPAGSDETKP

>DmelOBP47a-B

MNRVLVLLLVLKMFALSEININLGLTVADESPKTITEEMIRLCGDQTDISLRELNKLQREDFSDPSESVQCFTHCLYEQMGLMHDGVFVERDLFGLLSDVSNTDYWPERQCHAIRGNNKCETAYRIHQCQQQLKQQQQNLLATKEVEVTTTPAGSDETKP

>DmelOBP47b

MSPSQLLVIFASLALNTRLVFGQATIDCQRPPQLVDPALCCKDGGRDQVAEQCAQRILGTANGQKAGGPPSLDTAACLAECILTSSKYIDEPQKLNLANIRSDLSAKFSNDTLYVETMTMAFSKCEPQSQRRLAMIMQQQQQVQQQKTQQQQPRCSPFSAIVLGCTYMEYFKNCPDHRWTPNAQCTLAKAYVTQCGLGA

>DmelOBP50a-A

MRTGRILVALIFLGLIIPFRAAKCRAAPKSVQNVHVCCSAPLPNWGVFNRECHKSAIQASCRLDCDFNASSVLQGNRLIQAKVRPMLERAFSNEPTIDAYESNFAKCSTVVRSKYQELSPLSRQSDACDRHALFYSLCAYARLIFTCPDKMWQRNNRMCQEAKAYAKKCPWPALKMFMRNT

>DmelOBP49a

MLSKSQLLLLVVGFCLNAAVSADVDCSKRPSFVNPKTCCPMPDFVTAELKQKCIKFDMTPPPPPDGEASGSFESKRRHHHPHPPPCFFSCIFNETGIYQNRKLDEAKLNAYLQEVFEDSSDLQTTATQAFTTCATKVADFEANLPPRPAPSPPPGFPMCPHDAGHLMGCVFRNMMKNCPDSIRNDSQQCTDMKEFFTKCKPPRGPPPSAEDM

>DmelOBP50a-B

MRTGRILVALIFLGLIIPFRAAKCRAAPKSVQNVHVCCSAPLPNWGVFNRECHKSAIQASVSINRISKSKVNLANFLIKCRLDCDFNASSVLQGNRLIQAKVRPMLERAFSNEPTIDAYESNFAKCSTVVRSKYQELSPLSRQSDACDRHALFYSLCAYARLIFTCPDKMWQRNNRMCQEAKAYAKKCPWPALKMFMRNT

>DmelOBP50b

MSSVLHLLGFLWLPLLVYSVSNDMGGLQKCTELLNTHKLVYCCGKSFLDKFPFVGSNCTPFWDDYGPCRYECLYRHWDLLDQDNKIKKPELYLMITSLYSPLNGYDKYGAAFKAAHETCEALGSRHADFLLLYSNQVADKMGMASSTCLPYAMLHAQCTMVYLTANCPRENWIDDPKCNSLQKLLSSCTKKLDEKTNALKGKDEELTDNGCGHIDSEGSNLLMACFLTLMIAKFISDH

>DmelOBP50c

MARHIALLICSLLAMAGCDPIDVDCTRRQDFNIVKDCCVYPTFRFDQFKSQCGKYMPVGAPRISPCLYECIFNKTNTVVDGAIHPDNARLMLEKLFGNQDFEEAYFNGLMGCSDSVQEMISNRRSRPQRKTEQCSPFSLFYGICAQRYVFNHCPSSSWSGTESCEMARLQNMNCSKPSRGSSHRL

>DmelOBP51a

MKVFIGLVLLLAVTTLSSALFESEANECAKKLGITPDYFENFPHSSRVKCFYHCQMEKLEIIANGVVTPFDLKVLNISPESYDKYGVKVKPCLKLSHRDKCELGYLVFQCLKREFNL

>DmelOBP50e

MHKYIICFGFLLIILECSLASFNCSAPPNFNNFDINTCCRTPELDMGDVPQKCHKYVSGLKSANSKYPSYAHLCYPDCIYRETGAMVNGKIKVNRVKQYLEEHVHRRDQEIVSHIVQSFESCLSNVKGHMKSLNIESYKVLPHGCSPFAGIIYSCVNAETFLNCPQQMWKNEKPCNLAKQFAEQCNPLPHVPLPSS

>DmelOBP50d

MLHKLTWVLIFIPAFRAADPICSQRPDVTALRNCCKLPNLDFSSFNSKCSQYLVNGVHISPCSFECIFRAANALNGTHLVMENIEKMMKTILGSDEFVHVYLDGFRSCGNQEKVLIKAMKRRRVPITGKCGSMAIMYGLCAHRYVYRNCPESVWSKSATCNEAREYSIRCDDM

>DmelOBP56c-C

MYFRASLMALLCLTLSEFVSKAWTRSLSVSLNMSMTRTLVPDPPNGTENKLSQEMLRACMRRTEISMSQLKLFHMSLMNSDYNNDNDIAPTPVQSIGDVNNLGDLDFNGNSQMPYLDLKHNEPLQCFVSCLYETLDLDRYNVLLEEAFKNQVQTIIQHEKAEIKECSDLQGKTRCEAAYKLHLCYNHLKTLEAEQRIREILERTEAENEGFGPEGSDFIDGIQHSGEAMTTAKSE

>DmelOBP56b

MKLIYLLVVFLIFALSELVAGQSAAELAAYKQIQQACIKELNIAASDANLLTTDKEVANPSESVKCYHSCVYKKLGLLGDDGKPNTDKIVKLAQIRFSSLPVDKLKSLLTSCGTTKSAATCDFVYNYEKCVVKGISA

>DmelOBP56a

MNSYFVIALSALFVTLAVGSSLNLSDEQKDLAKQHREQCAEEVKLTEEEKAKVNAKDFNNPTENIKCFANCFFEKVGTLKDGELQESVVLEKLGALIGEEKTKAALEKCRTIKGENKCDTASKLYDCFESFKPAPEAKA

>DmelOBP56d-B

MKFLIVLSVILAISAAELQLSDEQKAVAHANGALCAQQEGITKDQAIALRNGNFDDSDPKVKCFANCFLEKIGFLINGEVQPDVVLAKLGPLAGEDAVKAVQAKCDATKGADKCDTAYQLFECYYKNRAHI

>DmelOBP56d-A

MKFLIVLSVILAISAAELQLSDEQKAVAHANGALCAQQEGITKDQAIALRNGNFDDSDPKVKCFANCFLEKIGFLINGEVQPDVVLAKLGPLAGEDAVKAVQAKCDATKGADKCDTAYQLFECYYKNRAHI

>DmelOBP56c-B

MYFRASLMALLCLTLSEFVSKAWVMFFIFYISFTRSLSVSLNMSMTRTLVPDPPNGTENKLSQEMLRACMRRTEISMSQLKLFHMSLMNSDYNNDNDIAPTPVQSIGDVNNLGDLDFNGNSQMPYLDLKHNEPLQCFVSCLYETLDLDRYNVLLEEAFKNQVQTIIQHEKAEIKECSDLQGKTRCEAAYKLHLCYNHLKTLEAEQRIREILERTEAENEGFGPEGSDFIDGIQHSGEAMTTAKSE

>DmelOBP56f

MKVFLLFIFISAIWLQAFCMKSSEKIKACLKRQLGYTITENTKFDAKEDSLQSKCFYHCLLEVKGVIANDAISSEQPRKVLEKKYGITDTDELEKAEEKCHSIKASGKCELGYEILKCYQSITKH

>DmelOBP56e-B

MKVFFVFAALAALSLASAGLTDSQKAEAKQRAKACVKQEGITKEQAIALRSGNFADSDPKVKCFANCFLEQTGLVANGQIKPDVVLAKLGPIAGEANVKEVQAKCDSTKGADKCDTSYLLYKCYYENHAQF

>DmelOBP56e-A

MKVFFVFAALAALSLASAVGLTDSQKAEAKQRAKACVKQEGITKEQAIALRSGNFADSDPKVKCFANCFLEQTGLVANGQIKPDVVLAKLGPIAGEANVKEVQAKCDSTKGADKCDTSYLLYKCYYENHAQF

>DmelOBP56h-A

MKFTLFCIALAAFLSMGQCNPDFRQIMQQCMETNQVTEADLKEFMASGMQSSAKENLKCYTKCLMEKQGHLTNGQFNAQAMLDTLKNVPQIKDKMDEISSGVNACKDIKGTNDCDTAFKVTMCLKEHKAIPGHH

>DmelOBP56g-B

MRATFALTLLLGCLSGILAQQANIDSSVSKELVTDCLKENGVTPQDLADLQSGKVKAEDAKDNVKCSSQCILVKSGFMDSTGKLLTDKIKSYYANSNFKDVIEKDLDRCSAVKGANACDTAFKILSCFQAAN

>DmelOBP56g-A

MRATFALTLLLGCLSGILAQANIDSSVSKELVTDCLKENGVTPQDLADLQSGKVKAEDAKDNVKCSSQCILVKSGFMDSTGKLLTDKIKSYYANSNFKDVIEKDLDRCSAVKGANACDTAFKILSCFQAAN

>DmelOBP56i-A

MHFFTCCALLLVVVTLPTCFVQAGPIKDQCMAAAGITAQDVANRHETDDPGHSVKCFFRCFLENIGIIADNQIIPGAFDRVLGHIVTAEAVERMEATCNMIKSETSHDESCEFAWQISECYEGVRLSDVKKGQRTRNHRG

>DmelOBP56i-B

MVVCVQRTQVQAGPIKDQCMAAAGITAQDVANRHETDDPGHSVKCFFRCFLENIGIIADNQIIPGAFDRVLGHIVTAEAVERMEATCNMIKSETSHDESCEFAWQISECYEGVRLSDVKKGQRTRNHRG

>DmelOBP56h-B

MKFTLFCIALAAFLSMGQCNPDFRQIMQQCMETNQVTEADLKEFMASGMQSSAKENLKCYTKCLMEKQGHLTNGQFNAQAMLDTLKNVPQIKDKMDEISSGVNACKDIKGTNDCDTAFKVTMCLKEHKAIPGHH

>DmelOBP57b-B

MFIYRLVFIAPLILLLFSLAKARHPFDIFHWNWQDFQECLQVNNITIGEYEKYARHETLDYLLNEKVDLRYKCNIKCQLERDSTKWLNAQGRMDLDLMNTTDKASKSITKCMEKAPEELCAYSFRLVMCAFKAGHPVIDSE

>DmelOBP57b-A

MFIYRLVFIAPLILLLFSLAKARHPFDIFHWNWQDFQECLQVNNITIGEYEKYARHETLDYLLNEKVDLRYKCNIKCQLERDSTKWLNAQGRMDLDLMNTTDKASKSITKCMEKAPEELCAYSFRLVMCAFKAGHPVIDSE

>DmelOBP57c

MLKLWLICILTVSVVSIQSLSLLEETNYVSDCLASNNISQAEFQELIDRNSSEEDDLENTDRRYKCFIHCLAEKGNLLDTNGYLDVDKIDQIEPVSDELREILYDCKKIYDEEEDHCEYAFKMVTCLTESFEQSDEVTEAGKNTNKLNE

>DmelOBP57a

MFNTRLAIFLLLIVVSLSQAKESQPFDFFEGTYDDFIDCLRINNITIEEYEKFDDTDNLDNVLKENVELKHKCNIKCQLEREPTKWLNARGEVDLKSMKATSETAVSISKCMEKAPQETCAYVYKLVICAFKSGHSVIKFDSYEQIQEETAGLIAEQQADLFDYDTIDL

>DmelOBP57e

MLDQLTLCLLLNFLCANVLANTSVFNPCVSQNELSEYEAHQVMENWPVPPIDRAYKCFLTCVLLDLGLIDERGNVQIDKYMKSGVVDWQWVAIELVTCRIEFSDERDLCELSYGIFNCFKDVKLAAEKYVSISNAK

>DmelOBP57d

MPEKMSLRLVPHLACIIFILEIQFRIADSNDPCPHNQGIDEDIAESILGDWPANVDLTSVKRSHKCYVTCILQYYNIVTASGEIFLDKYYDTGVIDELAVAPKINRCRYEFRMETDYCSRIFAIFNCLRQEILTKS

>DmelOBP58c

MKCTILLSFFSLIWFAGGIKIDCENTEAINEDHIHYCCKHPDGHNDLIEGCARETNFTLPNQNEEALVDITADRAIRGTCFGKCVFSKLNLMKDNNLDMDAVRSLFTERFPDDPEYAKEMINAFDHCHGKSEENTSMFLSKPLFKQMSKQFCDPKSSVVLACVIRQFFHNCPADRWSKTKECEDTLAFSKKCQDSLATL

>DmelOBP58b

MLRIGFVICVIISLRLNGLVAVRVHCRHMERIHEENIHHCCKHQDGHDDVTESCAKQTNFRLPSPNEEAIVDVTVDQAMVGTCWAKCVFDHYNLMENNTLDMDKVRSYYKRYHQTDPEYATEMLNAYEKCHTQSEEATEKFLSLPIVRAFSTAKFCKPTSSIIMSCVIYNFFHNCPASRWSNTTECVETLAFARKCKDVLTTM

>DmelOBP59a

MKQLIFLLICLSCGTCSIYALKCRSQEGLSEAELKRTVRNCMHRQDEDEDRGRGGQGRQGNGYEYGYGMDHDQEEQDRNPGNRGGYGNRRQRGLRQSDGRNHTSNDGGQCVAQCFFEEMNMVDGNGMPDRRKVSYLLTKDLRDRELRNFFTDTVQQCFRYLESNGRGRHHKCSAARELVKCMSEYAKAQCEDWEEHGNMLFN

>DmelOBP73a

MRITQLLCISCMVITSIDAVEYLIRFETKKAKCLNPPRTARKVESVIRECQDEVRNKLVNEAYEILKEQVSQNQPPIDPNDDSIDFIWPSVPEAPSLDHSPNISQYEYIVYDEPEPQRHVARLMRNIRRLDVASSGIYHPTLVPLEDKRIAGCLLHCVYAKNNAIDQRGWPTLDGLVHFYSEGVHEHGFFMATLRSVNLCLRTMTARYGVNRKELPKKGESCDLAFDVCSHMNTNIFKDLQFWAPFISYVNESRAINYI

>DmelOBP69a

MVARHFSFFLALLILYDLIPSNQGVEINPTIIKQVRKLRMRCLNQTGASVDVIDKSVKNRILPTDPEIKCFLYCMFDMFGLIDSQNIMHLEALLEVLPEEIHKTINGLVSSCGTQKGKDGCDTAYETVKCYIAVNGKFIWEEIIVLLG

>DmelOBP58d

MVNIVCYWTFLILVAVSKAQDNEETTAVAISSGDLTEDKCNTSRAGCCSELYIGEEEDLVKCFVIHSPKLPVDGDADIGKTLRFLSCFVECLYKQKKYIGKSDTINMKMVKLDAEKTFVDRPKEKDYHIAMFEFCRKDAVGVYNLLKASPGAKVLLKGACRPYLLMVFMCISDYHQKHECPYFRWEGTAKAGTKDMCENAKAECYQIDGITLPTKSPA

>DmelOBP83a-C

MDQEGPRSSGKERNGKSHIKMALNGFGRRVSASVLLIALSLLSGALILPPAAAQRDENYPPPGILKMAKPFHDACVEKTGVTEAAIKEFSDGEIHEDEKLKCYMNCFFHEIEVVDDNGDVHLEKLFATVPLSMRDKLMEMSKGCVHPEGDTLCHKAWWFHQCWKKADPKHYFLP

>DmelOBP83a-A

MALNGFGRRVSASVLLIALSLLSGALILPPAAAQRDENYPPPGILKMAKPFHDACVEKTGVTEAAIKEFSDGEIHEDEKLKCYMNCFFHEIEVVDDNGDVHLEKLFATVPLSMRDKLMEMSKGCVHPEGDTLCHKAWWFHQCWKKADPKHYFLP

>DmelOBP83a-B

MALNGFGRRVSASVLLIALSLLSGALILPPAAAQRDENYPPPGILKMAKPFHDACVEKTGVTEAAIKEFSDGEIHEDEKLKCYMNCFFHEIEVVDDNGDVHLEKLFATVPLSMRDKLMEMSKGCVHPEGDTLCHKAWWFHQCWKKADPKHYFLP

>DmelOBP83cd

MQMKSGILIALCLCLSLNEGLALLEHEGETINRCIQNYGGLTAENAERLERFKEWSDSYEEIPCFTRCYLSEMFDFYNNLTGFNKDGIVGVFGRPVYEACRKKLELPFESGESSCKHAYEGFHCITNMESHPFTVIDNMPNISPSAKDAMKDCLQDVHQDEWKSFDAFAYYPVNEPIPCFTRCFVDKLHIFEEKTRLWKLEAMKQNLGIPAKGARIRTCHRHRGRDRCATYYKQFTCYAMAV

>DmelOBP83b

MVKYPLILLLIGCAAAQEPRRDGEWPPPAILKLGKHFHDICAPKTGVTDEAIKEFSDGQIHEDEALKCYMNCLFHEFEVVDDNGDVHMEKVLNAIPGEKLRNIMMEASKGCIHPEGDTLCHKAWWFHQCWKKADPVHYFLV

>DmelOBP83ef

MSSPRAVLVSLFLICSQALADLSGDAQTLEKCLRQLSSPESIAGDLRKLERYSSWTREEVPCLMRCLAREKGWFDVEENKWRLKQLTEDLGADVYNYCRFELRRMGSDGCSFAYRGLRCLKQAEMHAGTSLSTLLQCSRQLNATNVELLQYSKLKSKEPIPCLFQCFADAMGFYDPDGNWRLENWKQAFGPSGNEDQSSGADYSGCRLSGTQREVALSKCSWMYHEYKCWERVNGNKLVEDNEEQ

>DmelOBP83g

MQSQSLLLIVAAVATFLVAQTTAKFLLKDHADAEKAFEECREDYYVPDDIYEKYLNYEFPAHRRTSCFVKCFLEKLELFSEKKGFDERAMIAQFTSKSSKDLSTVQHGLEKCIDHNEAESDVCTWANRVFSCWLPINRHVVRKVFA

>DmelOBP84a-C

MFHSLYLIGILSLIWVAAQDIVPDDPEVQMQMHAMFYTARVACADENLIPYVRACAVIAFLILSPNCARALQDHAKDNGDIFIINYDSFDGDVDDISTTTSAPREADYVDFDEVNRNCNASFITSMTNVLQFNNTGDLPDDKDKVTSMCYFHCFFEKSGLMTDYKLNTDLVRKYVWPATGDSVEACEAEGKDETNACMRGYAIVKCVFTRALTDARNKPTV

>DmelOBP84a-A

MYSALVRACAVIAFLILSPNCARALQDHAKDNGDIFIINYDSFDGDVDDISTTTSAPREADYVDFDEVNRNCNASFITSMTNVLQFNNTGDLPDDKDKVTSMCYFHCFFEKSGLMTDYKLNTDLVRKYVWPATGDSVEACEAEGKDETNACMRGYAIVKCVFTRALTDARNKPTV

>DmelOBP93a

MYVYNLLFVVIVFSYCAKSFNYTSCDHAKQPKFLSSCCDVQKNDKAINSCRKSLLGNNSTNSNGEVRNLKSDKVALHACIAECSFRTNGFLLSNGTVNTQALQKSYQQRYKNDPNMSQLMLKSLNSCTDYARKRVQEFQWMPKKGDCDFYPATLLACVMEKVYINCPTSKWKNTSDCTAMWKYLVACDDVASNKKK

>DmelOBP85a

MSPGSVVFSMFLTRPSLDKGNSECRKSLNLPAHRKFNFAELYTINMCIEECNFIGCGYIEIDPPFRLDLANIRTNLQTIAPQPQNESIPFLVDAYRKCELFRSSHGRRFTLHLPDIEFIEEPCNPFALQITICVRIHAMQKCPSEFYVDSDECRLAREYFTQCVGDIETNLA

>DmelOBP99a-A

MKVFVAICVLIGLASADYVVKNRHDMLAYRDECVKELAVPVDLVEKYQKWEYPNDAKTQCYIKCVFTKWGLFDVQSGFNVENIHQQLVGNHADHNEAFHASLAACVDKNEQGSNACEWAYRGATCLLKENLAQIQKSLAPKA

>DmelOBP99c-B

MDLSEEEALQIAQSCVDDNAQKNPTDVWAFRGHQCMMASKIGDKVRAFVKAKAEEAKKKAA

>DmelOBP99c-A

MLKYLIVALALCAVAHADDWTPKTGEEIRKIRVDCLKENPLSNDQISQLKNLIFPNEPDVRQYLTCSAIKLGIFCDQQGYHADRLAKQFKMDLSEEEALQIAQSCVDDNAQKNPTDVWAFRGHQCMMASKIGDKVRAFVKAKAEEAKKKAA

>DmelOBP99a-B

MKVFVAICVLIGLASADYVVKNRHDMLAYRDECVKELAVPVDLVEKYQKWEYPNDAKTQCYIKCVFTKWGLFDVQSGFNVENIHQQLVGNHADHNEAFHASLAACVDKNEQGSNACEWAYRGATCLLKENLAQIQKSLAPKA

>DmelOBP99d

MNHLRLEIICWSCLLIAMAVSTEAASVWKLPTAQMVYEDLEKCRQESQEEDAATLRCLVKKLGLWTDESGYNARRIAKIFAGHNQMEELMLVVEHCNRMEQDTSHLDDWAFLAYRCATSGQFGHWVKDFMSQKEVER

>DmelOBP99b-B

MKVLIVLLLGLAFVLADHHHHHHDYVVKTHEDLTNYRTQCVEKVHASEELVEKYKKWQYPDDAVTHCYLECIFQKFGFYDTEHGFDVHKIHIQLAGPGVEVHESDEVHQKIAHCAETHSKEGDSCSKAYHAGMCFMNSNLQLVQHSVKV

>DmelOBP99b-A

MKVLIVLLLGLAFVLADHHHHHHDYVVKTHEDLTNYRTQCVEKVHASEELVEKYKKWQYPDDAVTHCYLECIFQKFGFYDTEHGFDVHKIHIQLAGPGVEVHESDEVHQKIAHCAETHSKEGDSCSKAYHAGMCFMNSNLQLVQHSVKV

>DmelOBP-lush

MKHWKRRSSAVFAIVLQVLVLLLPDPAVAMTMEQFLTSLDMIRSGCAPKFKLKTEDLDRLRVGDFNFPPSQDLMCYTKCVSLMAGTVNKKGEFNAPKALAQLPHLVPPEMMEMSRKSVEACRDTHKQFKESCERVYQTAKCFSENADGQFMWP

>BdorOBP19d-1

MKYFVVFLAICSFAISFSEADEFGEKVKKIAEECKGQVGASDDDVARLFKYEPAANDKAKCLTACTMKKLGTMDENNKVVEAGAIAYIKQLSGGDAEFEKLSLETYNECKSTPESSNECEYAEAFRQCVLESAKSKGLKILPQV

>BdorOBP19d-2

MKILNICLIVCVALISNAKCNYEEAKAVANECKEEVGATDDELETILKMEAAESTTEKCLGACVMKRFGAMNGDGKFDREKAMEILAIIADGNEEQHALGVEVLDACADIDVNEDHCEAAEEYRTCMHAKAKEIGFVVGRV

>BdorOBP19d-3

MEILIVLPILAVSSVFTITNAAAESEPPHYSSLRVMAEAAIEDCYEDSAQSVKVQITDESFDEILKGSRTNLSHNAKCLRYCIMRKNGLLSMDNSIDEENILQIFGIIHPQIKKDSLLDVLHKCALETDKQTDNCERAFVATSCILRELQADGVTDI

>BdorOBP56d-1

MKSYILLVALISHSAVATHELKSNKSTQVHQFYEDCLKESGASAAQLDALKKGDFNAVDDKAKCFLKCLQNKKGILENGVPNEAAIHKVMTPAIGNSPPKNTLAKCNGLKGANECDTAFQIYKCYRQEHVGLI

>BdorOBP56a

MKSSIVCCILATVVLSLCVFNAEAGLRKPKKLTPELEAKFEVLTAWIAYRLNLKHAKEACVGEYGFSDELATNLVKIKVANPSDREKCYVNCLYNKLVFYKDDAINKQAMKESLYEIVGEQRLLNIVDGCLNAGGTNACDKVYKFHACASPEFDKVRSDIFLPDE

>BdorOBP56d-2

MNFFAVAVLIVFVAVAAAQEGMLTPEQIQKVHTLSNECLKETGASEDAIRALIKGDDSQVDGKVKCYAQCMLVKLGYVENGKVNEEKVQNILGKLIGEEKAKATQAKCNGLKGTDECDTAYQIRQCYSAGYNGFAF

>BdorOBP56d-4

MKFFAVAVLLAFVAVAVAQEGVGKLTEEQKQKVHAAAAECFKETGASEDAVRALLKGDDSQVDGKVKCFAKCTLGKLDLLQNGKVNEEKVQKILGKLIGEEKAKAAQAKCNGLKGTDECDTAYQIRQCYAAGHESFVF

>BdorOBP56d-3

MKFFAVAVLLAFVAVAAAQEGVNKLTEEQKQKARALGTECLKETGASEEAIRALIKGDDSQVDGKVKCFSKCMQEKLGFVENGKVNEEKVQNFLGKLIGEENAKATQAKCNDLKGTDECDTAFQIRQCYAAGHEGLDF

>BdorOBP56h-1

MQKFYILTIIAALVTLAVCQLPADLEKFHKACMDEAKVTDEQMRQFFQNGMKASDATENIKCQMKCMMQKQGIWKDGVFDADAKIKELVQNPKFKGKEADLTKAINNCKNEKGANECDTVFKISMCIKEFMTQNNL

>BdorOBP84a-1

MSNGNVFVMLPLTIILLYCGIMVSAQANGSVSSEGMDVAHICNNSFSIPSDYIVQFNRNGDLPEIVDKTGMCFIRCYFEKAGLLKNWQLNKGLIMQTMWPIKADSIAICEPEAKQEMNACVRSYAIAKCLMKRGFQDTCNDTVA

>BdorOBP56h-2

MKSFVTIALLVVGSAVVLCNPHDPEMRGYIEDCNKEHNVSPKDFHDFIEGKLTTVPENMKCSSQCIMVKQGIMDESGNFKPDAAKAKMKEDKLVAAVDECKDLSGSTPCDTAFKITSCMLSKK

>BdorOBP57c

MYQFGAHEKRATTTATMSATVLAAGGGKGKSIPGLTWLVLLAVIVVFALPPGAVALTPTAPTRSFVEACQVKHNITLQELDEFPTDPSPEDIDMKFKCYADCLLNGMGFMDSNGKLDAEGLHEWGILNDESYENMLECKAANDMEDDPCEYSFGMMLCARMLNSEEENYYSDEVDEAAEERRRK

>BdorOBP19d-4

MVKSSSALLIAGFICLMSLQCLTALSEDADKLSEKRKPLMTREDPSTLEDYKRTKRQLPQPLQEFQDFVTTSKTQCAKEMNINPNELQKSLLYEDQPTSIEKCMMECVLKRMEVMSKDDTLSTTTIGHIADIIGDNNALITSIAMASAENCKKFITAEDSCERAFQINKCIAAEMKMRKIKLIY

>BdorOBP19a-1

MYLLDRYMLFPEAKLITEEQMWATAKLMRDVCLPRFPKISIELANQLRDGNIPDNNKDVKCYINCVLEMMQTMKKGKFLYEASLKQVDLVLPDSYKDDYRAGLLKCKDASAGIKKDNCEAAYTILKCLRGEIKKFIFP

>BdorOBP19a-2

MLNKINSFVLATVFIALVLHSDPVSGGATEEQMISAGKLMRDVCLPKFSKISPEVADGIKEGNVPDTKDVKCYINCIMEMMQTMKKGKFLYESALKQIDLLMPDDYKDDYRNGLAKCKDVTSGIKNNCDASYALLICMRDNISKFLFP

>BdorOBP28a-1

MAKFILFAALCILSAAVSNAAFNKEEAIKNFMTRAEECRGEVGAADSDIQDIVAKVPASSKEGKCLRSCLMKKYGAMDSNGKFVKSVADQHAQDFTDGDADKLKTAREIIDACADIAVPDDHCEATEVYGKCFMDQAKAHGIQKFDF

>BdorOBP99a-1

MKYIVAVLLAALVAMAAAEEYKIRNQDDLLKARKECMEAKKVPTEHIEKFKKFEFPDDEVTRCYIECIFNKFQLFSPTEGFKTQNLIAQLGQNKENKDAVKADIEKCADKNEQKSDSCTWAYRGFKCFISKNLPLVQESLKKN

>BdorOBP69a

MNTKQFVFLVIIYQYTFYTGVTTLEVPKHMVSGVKKLTNICIKESGASEELFKDIRATGELPNNQNLKCFMHCVLDKIGLIDDDNIVHLDNLIEIMPPDFVPIIEQLHTTCGTKSGADGCETAFLTIECYIKKEPIISKMLFSTFAD

>BdorOBP99a-2

MKTIIALCLLLAVTSAEYVVKNEENLQQYRRECATELKVPAEHIEQFRKWQFPNDAVTQCYLKCVFEKFGLFDAETGFNVEHIHQQLQGAEVAPPGDADHDDVVHDKIAACVDTNEQGSNACEWAYRGGVCFIKENLQLVKHSVKPQA

>BdorOBP84a-2

MINHRLLSLALSLVLFGFLAGTRANPETDATNNKLDKQQSMEMTTPTAGAANETGFDFEEVVRTCNASYTIPLEYIQQFNETAELPNITDKTGMCFLKCYMEKTGLLRDWQLNPTLIRQTMWPATGDSLPVCQNEGSRETCPCKRTYAIAKCLTLRALVDARNKPLV

>BdorOBP-lush

MCLKINALKYFLTLLACTAVSAVTMQQFETSLDMMRNGCAPKFKIATEILDNLRAGEFIENNGDLKCYTRCIAQLAGTVTKKGDFSVQKALAQIPIILPPEMQDPAKEALNACKDVQKNYKESCDKVFYTTKCVRDFDPATFKFP

>BdorOBP-A10-X2

MLRFVAASVLICAIYHVTITSAAPHPPTTAAPLVANQAAYDTKFDNIDLDEVLNQERLLRNYIKCLENTGPCTPDSKMLKEILPDAISTDCAKCSEKQRLGSAKVTHFLIDNRPEDWARLEQIYDPQGNYRLNYLAAKDKGDGMEKTTEAVTKTQA

>BdorOBP71

MSKRSLAGLSFGPLRITVLFFYCSMCYALKCRTDDGPSESELKRITRNCMRKIGESVHPIGGGSMNSNNHQNHPYGPLHQSNFGPQHGGNSRYDYNYDYGDNADQYYGNANNPNNNNYNNNNYNNNNGNHDRDRNVLQQRNRDRQQNNRSDNNRSASSGSSNNNGGRYDNNDGNGNRGGGGGNGDGGNRNGNGSANSGGRNSGGNGGSGNSQRGFNQNNSNNNNNGNNRNGKNDTADVACVVHCFFDELNMLNSDDYPDRYKVQYGLTRDLRDRELRNFYTDTIQDCFQYLESQRRRDKCHYSRDLINCMTEYAKVNCDDWQEFNVVFN

>BdorOBP68

MKNLALIIFSIILVLKLNNSLKIDCDNPESINEDHIHYCCKHPDGYQEVIDSCAKETGFKYIKHDEEAMVDITVDHAITGTCFGKCVFNKLQFMKGADLDMAAVRTHFESKFKTDPEYAKEMINAFDHCHGKSVENTAKFLSNPIFRQPSAEFCDPKPGVILACVIREFFHNCPADRWAKTEECNTVLEFSKKCKDALTTI

>BdorOBP-A5-1

MWRRILCFFLAARCAVAVDNDVEKLFRDMEVVSDILDEPPKEMLKIEYNDGLDVGNGEEFTPTQTKDEPKLYWTSEPDAYYTVIMVNPDIPTRQNPLLREWLHWLVVNVPGVDIAKGDIIDPYIGPMAPKMSGVLRYVFLIYKQPGKQVFDEAKITNTDVTGHEKFSSMGFAGKYNMELVAGNLFQARWDELVPSLHKQFGISL

>BdorOBP56h-4

MTPLQLMDACNKESGITKEELQQYFDSQMDPAKATNAIKCHMKCVSEKLGFYKNNMLDDTLTIKYLNENNMAPKASVNNVKQSIQKCNQMKGANTCDTAYQIMTCFKSQPIFT

>BdorOBP56h-3

MMKDLFILGILSTLYSMAVCKEMMADEKFELPCLIEANVTEADLKKFRSNGLKANEANANIKCMAKCLMEKREVLKKGVFDPEKVYADLIRMPELKGLEDQIKEAINICKTEKGANDCDTAFKITMCLREFKSRNI

>BdorOBP99a-4

MKFFIVILAVVALAYADEEWVPKNVAQIKAIRQECIKDFPLSEEYIQKMKNFEYPDEEPVRKYLLCTAKKLGVFCEHEGYHADRVAKQFKMDLDEAEVIAIAEGCADKNVEGSSADVWAYRGHKCVMASKIGERVKAYIQKSVEEAKKH

>BdorOBP99a-3

MKFCLALLSLLMVVVFAVADHAGHTDYVVKTNEDLIRYRDECVSKLSIPSDLVDKYKEWSFPDDEKTRCYLKCVLEKFELFDAAKGFDVHNIHHQLVGANADHSDATHGAIENCAKEAAGDDACVRAYNGFTCFLKNNAQLVQAGVEKSSK

>BdorOBP-A5-2

MCIHIEYDGGLVVGRGEEFTPTQTKDEPKVDWTAEPDAFYTIIMTNPDIPTRQNPATREWLHWLVVNIPGTDLAKGYVLDPYIGPLNPKESGLVRNVFLIFKQLGKQEFDEPILNNTNVAGHERFSSKGFAKKYDMELVAGNIFTSRWDEYVTLLHKQFGIIK

>BdorOBP99b

MKFFIVILAVVALAYAKDEWEPKTEAELKVIANECIKDFPLSNEQVQKYTAYQHPDKESIRNRMLCAIKKXDFFSEHEGYHADRIAKQFQIDFHEAEVAAIAERCADKIVKGSSVDVWAYRSRKCVMTSKIGERLKARNQKI

>BdorOBP99a-5

MKFFIVILAVVALVYAKDEWVPKTEAELKVIVKECLKDFPLNNEQLQKYTTFQQPDEEPIRKYMLCTAKGVGFFSEHEGYHVDRVAKQFKLDLDEAEVAVITEGCADKNAEGSSVDEWAYRGHKCVMASKIGERLRVYIENLKKEAKKH

>BdorOBP99a-6

MKFFIVMLAVVTLAYAEDEWMPKNDAEVSVIRQECIKDFPLSEEQLQKFRIFEYPEEEALRKYLLCVTKAVGIFTEHEGYHADRVAKQFNINLDEAEVTIIAEGCADKNVEGSSADVWAYRIHKCVMASKLGERVKAYIQNLKKEAKKH

>BdorOBP99a-7

MKFFLVILAVVTQTYAEDEWRPKNMAELNAIRQECFKEYPLSEEQLQKIKNFEYTDEEPARKVLLCTVKKLGVFCEREGYNADRVAKQFKMDLDEAEALAIVEGCLDKNLEGSSADVWAYRGHECVVASKIGDRVKAYFLKSKK

>BdorOBP99a-8

MKFFIVILAHVALAYAEDEWMPKNMAELNVIRQECLKDFPLNDEYIEKMKNFEYPDEEPVRKYLLCTVKRFGIFREGQGYNIDRVAKQFKMDLDEAEVLAIVEGCVDKNTEGSSDDVWVYRCRKCVMASKIGDRVKAKSRE

>BdorOBP99a-9

MKYFMLIVILAVVALVQADDWSPKTVDDIKKIREECMKQVPSSDEEFQKRKENDYPDVESVRKYALCNSKGWGLYKESKGFYPDRVAEQFKDDMPEDEIKAIVNDCDEKTKEETDDERCYHLLKCVMSTKLGDHIKDLVKRLE

>BdorOBP99a-10

MKFFIVILVVIALTYAEDEWIPKNDTELEVIAQECLRDFPLSKEQLQKFSSFEYPDEEPIRKYMLCTAKRVGFFTEQEGYHADRVAKQLKMDLDEAEIVAIAEGCADKNVEGSSADVWAYRVHKCVMASKIGEHGKAYFQK

>BdorOBP83a

MHSRKTLLGTLLWIGFLLNLIWAQKELRRDETYPPPELLKELRPVHDSCVAKTGVTEEAIKEFSDGDVHEDELLKCYMYCVFEETDVLHEDGEVHLEKILDKLPESMHVIALHMGKKCLYPKGDNKCERAFWLHRCWKEADPKHYFLI

>BdorOBP99a-11

MNTQLILLLACVALVAGKFQIRTAQDALDAHEACHEEYRVPEDIYQKFLNYEFPAHKRTNCYVKCFVERMGLFTEEKGFDEKAIIAQFTAKSSKNLAKVSHGLEKCLDHNEHDSDTCTWANRVFSCWISVNRPIVRRTYIEN

>BdorOBP70

MLSNVVYRAYIALILLDKSGILAKDSHSFTKNRCLNPPRTARRVESFIQECQEDVKNKLISEAYYILKSQIKNENTALDITVDNISEINSAPAPVSVPSIIFHTIEENSKLASQHDAYQFQPERQQVTSLMNHIRRISYNPRSALYYPTLVPAEEKRLAGCLLHCVYAKNNAIDKLGWPTLDGLVNFYSEGVNEHGFFMATLRSVNLCLHAITVKYNIDRRKLPKRGESCDLAFDVFDCISDHLTGYCLNQYE
