# Supplementary material for: Transcriptomics reveal the molecular underpinnings of chemosensory proteins in Chlorops oryzae
Source: BMC Genomics. 2018 Dec 7;19:890. doi: 10.1186/s12864-018-5315-4 (PMC6286535; doi:10.1186/s12864-018-5315-4)
Supplement: Supplementary file 8 — Protein sequences of SNMPs used to construct phylogenetic tree. (DOCX 17 kb) [file 12864_2018_5315_MOESM8_ESM.docx]

>Co-Cluster-3781.108647

MKLNNSLTGKIETNEFAWLLNRNGSKEYEGSFNIDTGQNDLHQMGELKLWKGAPHTGFYEGECGRINGSTGDLFVPNRSPDEFITIFVTDTCRIINLEPTYKEVTIEGIKTWRYETTPNTFDNGQNNDDMKCYCPTEKQPDNCPKPGVTDLGPCGGGAPMYLSHANFMYADESYASTITGLTPNYDEDAFYLLIERKLGIPLEVRANVQANLFVRSDPDITLLKDIPEFYAPLFITASRARVDKKLAKQIRLVLYLPDLGRNIAIALIVIGGVMIIVGITLTFTRRWHGQRIKDPFEMD

>Co-Cluster-3781.67127

MLIPGIIKGPRSFVRSLLSPIVNHKIKVQSKENVILADVIKCHTCAETFVVFSNIDQNRGRLKTHSNNRLAVIIIGIIILILGIILSSTPWLDYFILKNLRLWNDTLSFHYWQRPGVVRLTKVYIYNVTNPDGFLNGEKPRLQEVGPFVYREDMEKVNINFHDNFTVSYQHKKILKFVPELSIDKNTPIVTPNIPLLTLTSLSRKLGYLLSKTISVVLTAAKFEPFINVTADQLVFGYDDALVSLAHRFYPKHLRPMERMGLLLGRNGTLTEVSTIKTGHTGMHEFGYIDRLNGMDHLSHWNEAPCTNIGGSEGSFFPPRDITKSDVVYVYDKDLCRIIPLRYMKTVEKDGIDADLYYLPEDTYGDSASNPANQCFDTNDYAAVKGLQNISPCQYGAPVYISNPHFYQADPKLLDAVEGLKPNKTEHETFFKIQPKLGVPLEGKVRIQLNLKVTQARDVYPVRNFRDFMFPVMWLEEGISELTPSIRRWVYLATVFAPNIIPVASYLMIASGALAIVFVFVRVYQNFVFARDPTLEILEMGRRSLRRGSSFIAQHQHKFIHRESYTLLKTVPSTLLDDDREDSVPIINNEL

>Co-Cluster-3781.122983

MVDVGLTIYGQKISVIKTADELLFTGYNDIMIDMARAMPIFGKDVDVPFDKFGWFYTRNSSADLTGVFNVNTGADDITRLGRLEAWNYSNRTKFFDSYCGYTNGSAGEFYPSNLEKNGSVTLFTPDLCRSIPLDYVKPVEVEGILGYKYTGGPRSVDNGSVYPENSCFCG

>Co-Cluster-3781.137336

MTEKVNIIWHDANSSVSYRKRSHYYFDAEGSRGSLSDHIIIINSIAVVSKEEAYQTQKKNYRVFMKYFYSLKTAAAQAK

>Co-Cluster-3781.141790

MYINWIDIFTRLRGKELALRPTSPTYGGWKVSPIPLFFDVYLFNWTNPEDFYAGSKKKPRFQQLGPYRFREIPDKVDMVWHNHNHSVSFRRKSTFYFDAAGSNGSLQDLVTSVNTVAHVAALRAKNGNAFEKGMLNRAFDLYHQKITTTRTADEWIFKGYSHPFVTLGSFLSKFSSKIEVPFSRVGWLFSRNGSSTYDGHFNVYTGADDVYKMGQIHAWNGLEHNGRFPGECGRVKGAMGEFFPPNLSTNDTISIYIPNLCRSIPLDYVETMEIHGVKTYRFAGGQRSVDNGTQFPEAKCFCVNDKCEASGIINIAPCKYNSSVYMSYPHFYKADSSYLEAIEGLEPDRDKHEFFITLEPNSGVPMDVGGGFQANYLLESIDGFPLYSKIKRTFIPIMWAEERVRVTEEIAKGIAMVPLIILVGQIITGILLASGVIFICWYPTKSLSRLFKDPKRKKSLLQPLSTINRTITITPQQSKGTKPRNGFIEGSVVPLIDFKNGSFIRSSDVMREELLGNTFRKPLSDVINR

>Co-Cluster-3781.160102

MKKMFLHWTLIVSTVGIVISVLGAYCGWYLFPKMVHKKVEESVIIADGSEQYKRFVQLPQPLTFKVYIFNVTNPDNIQQGAIPIVEEIGPYIYKQYRRKKVKHFSHDGSKITYVQDQVFEFDERASAPYTQSDNIVVLNMHMNAFLQVFEREITDIFQGFANRLNHRLNRTPGVRILKRLMERIRGKRKSVLQIAENDPGLALLLVHLNANLKAVFNDPKSMFVSTTVREYLFEGVRFCINTNGIAKAICNQIKESGSKTIRELSDGSLAFSFFNHKNGTGRDVYEVHTGKGDPRRVLEIQKLDDSHNLQVWLNSSDGETSVCNQINGTDASSFPPFRKRGDSMYIFSADICRSVQLFYQKDIQYHGIPGFRYSIGENFINDIGPEHDNFCYCVDKLTNVIKRKNGCLYAGALDLTTCLDAPVILTLPHMLGASNEYTRMIRGLKPDAKKHQTYVDVQYLTGTPLQGGKRVQFNMFLKSINRITITENLTTVLMPAIWLEEGIELNDEMVSFFKKKLINTLRTLNIIHCVALIGGVSVAVISLIYYVVQRRKPEEEAPLK

>Co-Cluster-3781.49122

MQLNRNKLLAYSSGAFVFAILFGWIGFPQILNLMLKKQVNLKPGSEVRDLWANTPFPLHFYIYVFNITNPEDVLNGAKPNLHEIGPFVFDEWKDKYNLEDDPVEDSITYNMRNTFYFNEKESLKHGLTGEEMITLPHPLLMPIAIFGQRERAAMMELISKAISIVFEDQSAMMTAKFMDMFFRGFYVDCSSEEFASKALCTVFYTGDVKQATQVNSTHFLFSFLGKANHSDAGRFTVCRGVKNVHKLGKVIRFDGEPEIDNWPRDECNQFIGTDSTIFPPFLKREEGLWAFTPDLCRSLGAKFFSKTTYHGLPATRYYMDFGDIKSDPSLHCFCDDPEDPSTCPPKGTMNLANCVGGPIIASLPHFFNGDPSLYENVNGLEPNDKKHGIWIDFETISGTPFQAAKRLQFNLDVEPVETVDKVSKVRKMILPMFWVEEGVAINKTFTNMLKYTLFLGLKFNAGIRWTLITVSLIGFMSAGYLFYRKSDTLDITVPPKIVESNKISDVKAEVTKVPIELPNAEAMRQRDLLNQRRIDGEERF

>Co-Cluster-3781.38760

MLLHKGHCFTAFGISLGLFGICFGLTWKYVFYKIIQKLLVIRPNSLIFDLWQIPSRSIEIDFYMFNWTNPEDFRKPNVKPKFQELGPYHFFERQDKFDFAWHANNASVSYRRRSIYKFDGKGSVGSLNDTLVTLNSLAVGVSTLAKGFNVVKRRILEFSTLIYPRDLTIRKTIGELLFDGYSDPLLIANRFIPTGIDVPFHKYGYCYGRNVTNTYFGAYNVLTGANDYNNMGKVHTWNGKSFGRSSLNKCSKIRGSAGEFYGPNLEVNRTIKMFIRELCRDVPLEFDRETYLKGIRAFKYIAGHQAIDNGTLNAENACFCDGECLPSGVMNTTECWYGSPIFTSYPHFYKADPFYLTQVDGLRPDKKKHEFSYTIEPNTGMGLSIMARTQINQLVRPIEHSRMLHDVRQIMFPLFWAEARLELSWDLLDYFRLIELVILFGQLFGAACFFVGILITLRNYYGTKKAFKKQNYDLLVLKTTQNGKVHSWCENSMEKKIVLPETLLL

>Co-Cluster-3781.47316

MKIHVSSFNKKMYKRIGIGSVILMVTGIVIGFVGFPALLNYLIVKEVTLKPGSETRQLWEKLPFPLNFSFYVFNVTNPDGVQNGEKPVVEEIGPFVFEEWKDKYNIEDNDTEDTVTFNMRNTFIFRPDLGLSGEEIITMPHPVLQVMALAVKRDRAAMIGIVSRGLTEMFHPTSPFISAPFMDIFYRGIDVDCSSDDFPVKAVCMGFFTGTVKGAVQVDDNSFKFSLVGANNHSDAGEFKVSRGIKQPGTVGKVLEFNGDEELSYWNGEECNKIRGTDSTIFPPLMNRDEALVVFSADLCRSLAPKFEQYTEYDGIPALRYTMDFGDIKNETENHCYCKSYPDECPPRGTLDLSPCNDAPMFASQPHFLNADPQLLDNVDGLCPSNWRHGIYIDYEIISGTPLSIARRLQFNLDVQPIEELDVMKNMRRLIMPLFWVEETVILGPEYTQLLKNKVFLIKNINNAFRWVAVVFGALGCGFSLIMIYVTKKTRVTMVETPMPAK

>Co-Cluster-3781.63294

MNPEEDAHEFYMVLEPRTGIALEVAARFQVNMLLEPIAGINLFNNTPRTFLPLIWFEQKVRISPELAKDLKLLPIILLSGQIFAAFCFAVGLLLLSWYPIEAIWHRCSSKDFKTPLPDNGRYKNTMVNLDATAKRPASPNVQKEKIQDVSPLLDKSKKSSVIEPTKGDTQNAKVPSSNDNKK

>Co-Cluster-3781.75288

MLPKGLSAFLVLAIGVLCITSAVLVKIFRPYDAIFNWKLNMEGGGEIFNLWAEPPVDLYIKVYLFNITNAEEFLAGREKMKVEQVGPYVYKELMTHKNITFNANYTMSTNPSHPLVWQEHLSEGHKEDDEVVMLNIAMLAIAHLTTNKPYFVRLALNTLFATSNSNPIVRMTAKEFMFGYESSLTTLGNTFLPNWISFEKVGLIDRMYDFSTDFETFYTGETNRNIGGLYATYRGDANLVQWNGDHCSNIELASDGTKFKSKLQPNETVKFFRKSMCRPVNLHRDGDLEKYGSLSGYKYVFEENALDNGAINEKNKCFCRNGKCQPRGLIDVTDCYYGFPISLSYPHFMDGDEKLIKKIDGMVPNKELHSSAFVIQPVSGHDFYQVQCLFVFFFIFLFCFVLFCF

>DmelSNMP1（NP_650953）

MQVPRVKLLMGSGAMFVFAIIYGWVIFPKILKFMISKQVTLKPGSDVRELWSNTPFPLHFYIYVFNVTNPDEVSEGAKPRLQEVGPFVFDEWKDKYDLEDDVVEDTVSFTMRNTFIFNPKESLPLTGEEEIILPHPIMLPGGISVQREKAAMMELVSKGLSIVFPDAKAFLKAKFMDLFFRGINVDCSSEEFSAKALCTVFYTGEIKQAKQVNQTHFLFSFMGQANHSDSGRFTVCRGVKNNKKLGKVVKFADEPEQDIWPDGECNTFVGTDSTVFAPGLKKEDGLWAFTPDLCRSLGAYYQHKSSYHGMPSMRYTLDLGDIRADEKLHCFCEDPEDLDTCPPKGTMNLAACVGGPLMASMPHFYLGDPKLVADVDGLNPNEKDHAVYIDFELMSGTPFQAAKRLQFNLDMEPVEGIEPMKNLPKLILPMFWVEEGVQLNKTYTNLVKYTLFLGLKINSVLRWSLITFSLVGLMFSAYLFYHKSDSLDINSILKDNNKVDDVASTKEPLPSANPKQSSTVHPVQLPNTLIPGTNPATNPATHHKMEHRERY

>AgamSNMP1（Q7QC49.3）

MELKERNFKKIGLICVAVLLCGMVFSYGIFPSILRFMIKQNVLLKPGTQIRDMFEKIPFPLDFKLHIFNVTNPDEIMRGGKPRVNDIGPLYFEEWKEKYDTVDNVEEDTLTFTLRNTWIFRPDLSALTGEEIVTIPHPLIMGVLLMVQRDREAMMPLVKKGVNILFDPLESAFLKVRIMDLLFDGIYVDCSSQDFAAKALCSGMDSEGAVMPHNETHYKFSFFGMRNHTEAGRWVVYRGVKNIRDLGRVVSYNEETEMDIWDGDECNQYIGTDSTIFPPFLTAQDRLWAWSPEICRSLGAHYVHKSKYAGLPMSYFELDFGDLKNEPHNHCFCRDAPDDCPPKGTMDLSPCLGGPIIGSKPHFYGADPKLVEAVDGLAPNKAAHDVYIHFELASICWFTGSPVSAAKRLQFSMELGPIRDHELFGQLPDVILPMFWAEEGASLNKTWTNQLKYQLFLGLKFNATVKWLTIIIGTVGAVGSAYMYFRKETK

TTDVAPVDVSTPDTNPSSAKDGVVNVSLGRNLPPVIDGLDKPPKLRATELQQERY

>DmelSNMP2（ABW70129.1）

MIHWSLIVSALGVCVAVLGGYCGWILFPNMVHKKVEQSVVIQDGSEQFKRFVNLPQPLNFKVYIFNVTNSDRIQQGAIPIVEEIGPYVYKQFRQKKVKHFSRDGSKISYVQNVHFDFDAAASAPYTQDDRIVALNMHMNAFLQVFEREITDIFQGFANRLNSRLNQTPGVRVLKRLMERIRGKRKSVLQISENDPGLALLLVHLNANLKAVFNDPRSMSVSTSVREYLFDGVRFCINPQGIAKAICNQIKESGSKTIREKSDGSLAFSFFGHKNGSGHEVYEVHTGKGDPMRVLEIQKLDDSHNLQVWLNASSEGETSVCNQINGTDASAYPPFRQRGDSMYIFSADICRSVQLFYQTDIQYQGIPGYRYSIGENFINDIGPEHDNECFCVDKLANVIKRKNGCLYAGALDLTTCLDAPVILTLPHMLGASNEYRKMIRGLKPDAKKHQTFVDVQSLTGTPLQGGKRVQFNMFLKSINRIGITENLPTVL

MPAIWVEEGIQLNGEMVAFFKKKLISTLKTLNIVHWATLCGGIGVAVACLIYYIYQRGRVVEPPVK

>AgamSNMP2（Q7Q6R1.5）

MVQCTLIWAGIGAMMAVSGALLGWVVFPRAVHEKVIEATELRQGTDQYKRWEALPQPLDFKVYIFNVTNPYEVMQGRRPKVVEVGPYVYFQYRQKDNVRFSRDRSKVHFSQQQMYVFDAESSYPLTENDELTVLNMHMNSILQIIDNQAKETITNFRSDVNNTLEKIPVVRVIKRIIERTTPIQSILQLAEDETYDSLRLINVELNRIFGRPDTMFLRTTPKQFLFDGVPFCVNVIGIAKAICKEIEKRNTKTIRTMPDGSLRFSFFSHKNMTDDGMFTINTGIKDPSRTQMIELWNGRTTLDVWNNRSSGLSSSCNKIHGTDGSGYPPFRTGVERMTIFSTDICRTVDIKLTGSSSYEGIPALRYEIDNNFLHEIGPEYGNDCYCVNKIPKSIVKSNGCLYKGALDLSNCFDAPVVLTLPHMLGVAEEYTALIDGMDPEPERHQIFVDVEPYTGTPLNGGKRVQFNMFLRRIDAIKLTDRLQPTLFPVIWIDEGIALNEDMVKLIDDSLMKVLSLLDVVQWVLIGVGLLLAVLMPTVYFVKRCRGEGSRTVSPAVTATTSAASLSTVAGVTGDRSK
